# Exploration of the Molecular Mechanisms of *Hyssopus cuspidatus* Boriss Treatment of Asthma in an mRNA-miRNA Network via Bioinformatics Analysis

**DOI:** 10.1155/2022/7111901

**Published:** 2022-05-05

**Authors:** Zhongdi Cai, Mimin Liu, Fengjuan Yuan, Li Zeng, Kaiyue Zhao, Ting Sun, Zhuorong Li, Rui Liu

**Affiliations:** ^1^Institute of Medicinal Biotechnology, Chinese Academy of Medical Sciences and Peking Union Medical College, Beijing 100050, China; ^2^The First People's Hospital of Urumqi (Urumqi Children's Hospital), Urumqi 830002, China

## Abstract

*Hyssopus cuspidatus* Boriss (*H. cuspidatus*) is a traditional Chinese medicine commonly used in the treatment of asthma. In the present study, we applied bioinformatics techniques for mRNA-miRNA profiling to elucidate the potential mechanisms of *H. cuspidatus* in asthma treatment. Bioactive compounds from *H. cuspidatus*, potential therapeutic targets of *H. cuspidatus*, and asthma-related targets were identified from the literature and databases. The intersection of *H. cuspidatus*-related targets and asthma-related targets was identified using the STRING platform. Gene Ontology and Kyoto Encyclopedia of Genes and Genomes pathway enrichment analyses were performed using the Metascape platform. Networks were constructed from these nodes using Cytoscape. The results showed that 23 active compounds were identified in *H. cuspidatus*, sharing 122 common asthma-related targets. Moreover, 43 miRNAs regulating 19 key targets involved in the antiasthmatic effects of *H. cuspidatus* were identified. Further analysis of biological pathways, active compound-key target-pathway network, and active compound-key target-miRNA network indicated that the antiasthmatic effects of *H. cuspidatus* mainly occurred through caffeic acid, methyl rosmarinate, luteolin, esculetin, and 8-hydroxycirsimaritin. These compounds interacted with multiple miRNAs, including miR-99a, miR-498, miR-33b, and miR-18a, regulating multiple genes, including JAK, STAT3, EGFR, LYN, and IL-6, in multiple pathways, including those involved in the regulation of JAK-STAT signaling, EGFR tyrosine kinase inhibitor resistance, PI3K-Akt signaling, and inflammation. In summary, we have elucidated the potential mechanisms of *H. cuspidatu*s treatment of asthma from a systemic and holistic perspective through analysis of compound-mRNA-miRNA interaction. Our study should provide new insights for further research on *H. cuspidatus* treatment of asthma.

## 1. Introduction

Asthma is a chronic respiratory disease affecting over 300 million people worldwide. Its pathogenesis involves repeated cycles of inflammation eliciting progressive airway obstruction and exacerbation of respiratory function [1]. While the prevalence of asthma is currently 1–18% of the total population (depending on the country), this is increasing year by year. Because of its high prevalence, approximately 250000 people die from asthma each year [2]. While Western medicines (e.g., corticosteroids, bronchodilators, and *β*_2_-agonists) effectively manage asthma symptoms, there are significant problems associated with existing drugs, including their high financial burden, poor tolerance, and side effects due to long-term use [3–5].

Asthma is known to be highly heterogeneous at both the clinical and mechanistic levels [6]. Moreover, asthma is generally accepted to be influenced by genetic factors, especially those involving gene expression and regulation. MicroRNAs (miRNAs) are short, endogenous, and evolutionarily conserved RNA molecules that are composed of 18–25 single-stranded non-coding RNAs. They are a category of regulators that are supposed to act as fundamental regulators to revolutionize the profile of gene expression. Several miRNAs have been demonstrated to contribute to pathological processes in asthma, including miR-192-5p, miR-124, and miR-34a [7–9]. miR-192-5p has been reported to inhibit autophagy by separately targeting matrix metalloproteinase (MMP) 16 and autophagy-related 7 (ATG7), which allow airway remodeling and exert antifibrotic effects, thereby relieving asthma [7]. miR-124 has been well-studied to be involved in the pathogenesis of asthma and has shown a negative correlation with inflammation, acute exacerbation risk, and acute exacerbation severity in asthma patients, along with a positive correlation with lung function [8]. miR-34a is upregulated in the lung-infiltrating cells of mice with ovalbumin and impairs Treg function. Further, it has been evidenced to be a target of resveratrol in the treatment of allergic asthma and works by increasing FOXP3 expression [9]. Therefore, miRNAs may be novel candidates for noninvasive biomarkers and potential regulators of target genes in the mechanism-directed prevention and therapy of asthma.

*Hyssopus cuspidatus* Boriss (*H. cuspidatus*) is a traditional herb used in Uyghur medicine and folk medicine with the functions of relieving asthma, dissipating phlegm, and benefiting the lungs. *H. cuspidatus* contains a variety of chemical components, including volatile oils, flavonoids, organic acids, and lipids [10, 11]. In recent years, *H. cuspidatus* and its extractions have played an important role in the treatment of asthma [12, 13]. Pharmacological studies have demonstrated that *H. cuspidatus* can treat asthma through its anti-inflammatory and antibacterial effects [14]. Our previous research revealed that JAX2, an ethanolic extract of *H. cuspidatus*, prevented bronchial asthma by inhibiting inflammation via the MAPK/NF-*κ*B signaling pathways [15]. We also found that *H. cuspidatus* extracts had an immunoregulatory effect on the balance of helper T cells (Th) and regulatory T cells (Tregs) in ovalbumin- (OVA-) induced asthmatic mice [16]. Moreover, *H. cuspidatus* extracts relieved the acetylcholine- or histamine-induced contraction of isolated tracheal rings from guinea pigs [17]. However, due to the complex composition and diverse activities of *H. cuspidatus*, this research has so far been confined to extracts. Thus, the active compounds, regulated genes, and role of miRNAs in *H. cuspidatus* extract treatment of asthma have not been fully elucidated.

**Figure 1:**
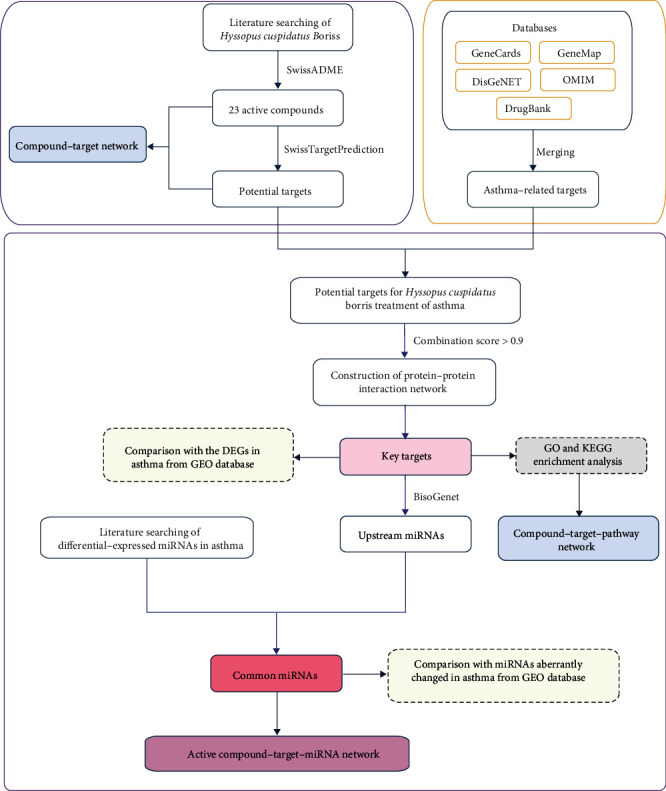
Workflow of the present study. DEGs: differentially expressed genes; GO: Gene Ontology; GEO: Gene Expression Omnibus; KEGG: Kyoto Encyclopedia of Genes and Genomes; miRNA: microRNA.

Because traditional Chinese medicine (TCM) has a synergistic effect based on multi-targets and multi-pathways, developments in network pharmacology provide possibilities for the exploration of the integral mechanisms underlying TCM [18]. In the present study, we aim to identify the key genes, upstream miRNAs, and signaling pathways regulated by the bioactive components of *H. cuspidatus* responsible for the treatment of asthma through bioinformatics analysis. In doing so, we hope to provide a systematic understanding of the underlying molecular mechanisms involved in *H. cuspidatus*-based intervention on asthma. The workflow of the present study is illustrated in Figure 1.

## 2. Materials and Methods

### 2.1. Identification of Active Components of *H. cuspidatus*

The Traditional Chinese Medicine Systematic Pharmacology (TCMSP, https://www.tcmsp-e.com/) database is one of the largest platforms, collects a large number of entries on chemical components of herbs, and contains comprehensive information on pharmacokinetic properties [including oral bioavailability (OB), drug-likeness (DL), intestinal epithelial permeability, blood-brain barrier, aqueous solubility, among others] of chemical components to help users in further screening. However, we did not find any information on *H. cuspidatus* in the TCMSP database. Therefore, we compiled the chemical components by searching all published literature related to *H. cuspidatus*. The molecular structures of these compounds were validated and exported using the PubChem (https://pubchem.ncbi.nlm.nih.gov/) platform. The SwissADME (http://www.swissadme.ch/) platform is a tool that allows the evaluation of pharmacokinetics, DL, and medicinal chemistry friendliness based on molecular structures [19]. We used the pharmacokinetics and DL of the resulting compounds in the SwissADME platform as indicators to screen for potential active ingredients. The “high” gastrointestinal absorption (GI absorption) in pharmacokinetics was one screening criterion. In addition, the DL included five different analyses from large pharmaceutical companies as filters for the rule, including Lipinski (Pfizer), Ghose (Amgen), Veber (GSK), Egan (Pharmacia), and Muegge (Bayer). The results of DL were indicated by “yes” or “no,” suggesting whether the molecule was defined as a similar drug. Compounds with at least two filter results of “yes” could be considered potentially active.

### 2.2. Construction of the Active Compound-Target (C-T) Network of *H. cuspidatus*

The potential targets of identified active components of *H. cuspidatus* were analyzed using the SwissTargetPrediction platform (http://www.swisstargetprediction.ch/), a tool for target prediction of bioactive compounds based on a combination of 2D and 3D similarity measures with given ligands. The chemical structures of the compounds obtained from the PubChem platform were imported into the SwissTargetPrediction platform, and the computational process was initiated through the JChem Web Service (version 18.29.0) and Open Babel (version 2.4.1), which performs the chemical processing of the input structures. On the prediction result page, a green bar indicated the estimated probability of a gene to be a true target based on its score. Therefore, we included all genes with probability values. Finally, these selected predicted targets were input into the Cytoscape software to construct a C-T network for *H. cuspidatus*.

### 2.3. Prediction of Asthma-Related Targets

Asthma-related targets were also searched using computer target technology. The GeneCards database (https://www.genecards.org) is a platform that aggregates 150 web sources to search for lists of disease-related genes. The OMIM database (http://www.omim.org) is an online catalog of human genes and genetic disorders that classifies the relationship between phenotypes and associated genes. Moreover, the GeneMap database (https://omim.org/search/advanced/geneMap) is an advanced search mode in the OMIM database. The DisGeNET database (https://www.disgenet.org/) is a discovery platform of gene–disease associations. The DrugBank database (https://go.drugbank.com/) collects information on investigational drugs and their therapeutic targets. Therefore, corresponding information of disease phenotype can be searched using these databases. Using “asthma” as a keyword, potential targets related to asthma were collated from the GeneCards, GeneMap, OMIM, DisGeNET, and DrugBank databases. The targets identified in the five different disease databases were then merged, and duplicate values were removed to obtain the asthma-related targets.

### 2.4. Construction of Protein–Protein Interaction (PPI) Network for the Active Compounds in *H. cuspidatus* Treatment of Asthma

The PPI network was constructed to identify the essential proteins and possible functional modules for the action focused on the mechanisms of active compounds in *H. cuspidatus* treatment of asthma. First, the intersection of potential targets of bioactive compounds of *H. cuspidatus* and asthma-related targets was obtained using the Venny 2.1.0 software (http://bioinfogp.cnb.csic.es/tools/venny/index.html), identifying key asthma-related targets involved in the antiasthmatic effects of *H. cuspidatus*. Second, these key intersecting targets were submitted to the STRING 11.0 database (https://string-db.org) utilizing a combination score threshold of “highest confidence” (>0.9). The PPI network was further analyzed using the molecular complex detection (MCODE) tool in Cytoscape (3.8.2) software to obtain the core submodules. The functions of the core submodules were subsequently described by analyzing the biological processes in which they were involved.

### 2.5. Gene Ontology (GO) and Kyoto Encyclopedia of Genes and Genomes (KEGG) Enrichment Analyses

To discover the molecular mechanisms of gene functions and enriched signaling pathways for the targets of *H. cuspidatus* involved in asthma, GO and KEGG analyses were performed using the Metascape (https://metascape.org/) platform. The targets of *H. cuspidatus* related to asthma treatment were imported into the Metascape platform, and the results were screened using *P*-value < 0.01. This process yielded the main biological processes and signaling pathways in which the key targets involved were enriched.

### 2.6. Construction of the Active Component-Target-Pathway (C-T-P) Network

Based on the targets mapped by the KEGG results, an active C-T-P network was constructed using Cytoscape (3.8.2) software. The network topology parameters (including degree, betweenness, and closeness) of active compounds and targets were analyzed using the tools in Cytoscape. Therefore, the key targets and the main active compounds that exert drug effects were determined based on the network topology parameters.

### 2.7. Reverse Prediction of miRNAs for Key Targets during *H. cuspidatus* Treatment of Asthma

To obtain miRNAs for these identified key targets associated with *H. cuspidatus* treatment of asthma, the backward prediction was conducted using the BisoGenet (3.0.0) tool in the Cytoscape (3.8.2) software. In brief, key targets were put into the “Input Identifiers.” Subsequently, we clicked on the “Gene identifiers only” and only chose “micro-RNA silencing Interaction” in the data settings. Consequently, the key target-miRNA interaction network was constructed.

### 2.8. Construction of the Active Compound-Target-miRNA (C-T-M) Network

Before revealing the interaction between these active compounds of *H. cuspidatus* and their miRNAs along with target genes, the miRNAs known to be aberrantly expressed in asthma were collated from the published literature, and an intersection of predicted miRNAs derived from key targets and collated miRNAs from literature was obtained using Venny 2.1.0 to plot a Venn diagram. The intersecting miRNAs were then mapped to the key targets and the active compounds to construct an active C-T-M network. Finally, the active compounds, key targets, and miRNAs involved in the *H. cuspidatus* treatment of asthma were obtained.

### 2.9. Gene Expression and Tissue-Specific Analyses for the Key mRNAs and Identified miRNAs Involved in *H. cuspidatus* Treatment of Asthma

To verify that the screened key mRNAs and miRNAs were related to the pathogenesis of asthma, we downloaded raw data of RNA sequencing and microarrays from the Gene Expression Omnibus (GEO) database (https://www.ncbi.nlm.nih.gov/geo/) and analyzed them using GEO2R to identify the differentially expressed genes (DEGs, |logFoldChange| > 0.5) in asthma. Following this, we separately compared the key mRNAs and identified miRNAs with DEGs and DEMs acquired from the datasets. With regard to key mRNAs, six datasets of experiments on gene expression profile (GSE104471, GSE89809, GSE74986, GSE74075, GSE37853, and GSE27335) for asthma were selected. With regard to miRNAs, the original gene expression profiles were obtained from the GSE142237, GSE146306, and GSE25230 datasets. In addition, we used the TISSUES platform (https://tissues.jensenlab.org) to identify correlations of key genes with lesion sites of asthma. The TISSUES platform contains multiple tissue expression data, which is integrated from a database based on manual collation of the literature, proteomic and transcriptomic screening, and automated text mining.

## 3. Results

### 3.1. Screening Results of Active Compounds of *H. cuspidatus*

**Figure 2:**
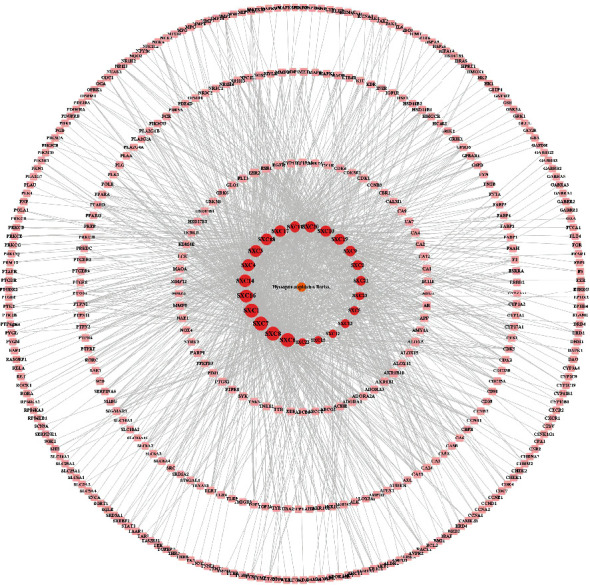
Compound-target network of *H. cuspidatus*. The orange octagonal node represents *H. cuspidatus*, the red-orange oval node represents the active compounds, and the pale pink round-rectangular node represents the potential targets. The degree of the active compound is shown by the size of nodes and the font size.

A total of 45 compounds were identified from *H. cuspidatus* in the published literature (Supplementary Table 1), and 23 were selected as active compounds based on the dual criteria of “high” GI absorption and DL using the SwissADME platform. These active compounds were named SXC1~SXC23 and are shown in Table 1. Using the SwissTargetPrediction platform, 385 potential targets of these active compounds were identified. Moreover, 23 active compounds and 385 potential targets of *H. cuspidatus* were uploaded into Cytoscape (3.8.2) software to yield the compound-target network with 409 nodes and 1388 edges. The visualization network diagram is shown in Figure 2, and the relationship between each component and target is shown in Supplementary Table 2.

### 3.2. Analysis of Asthma-Related Targets

Overall, 6692 target genes related to asthma were identified in GeneCards, 42 in GeneMap, 1198 in DisGeNET, 101 in OMIM, and 127 in DrugBank (Supplementary Table 3). As the number of asthma-related genes obtained in GeneCards was large, a score value greater than twice the median was set as a rule of thumb for the GeneCards data results. The final target list was collated by merging the GeneMap, OMIM, DisGeNET, and DrugBank results and then removing duplicate values. In total, 1616 targets related to asthma were included (Supplementary Table 4).

### 3.3. PPI Network and Topological Analysis

**Figure 3:**
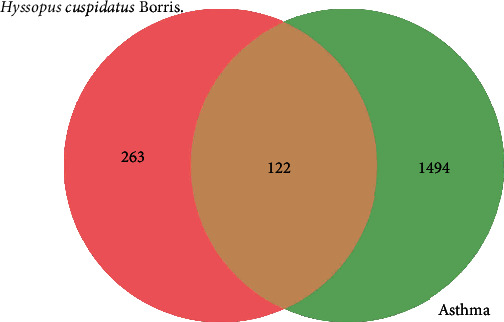
Venn diagram showing the number of *H. cuspidatus*-asthma common targets. Red and yellow areas show the number of potential targets of *H. cuspidatus*. Green and yellow areas show the number of asthma-related targets. The intersections (yellow areas) indicate the number of *H. cuspidatus*-asthma common targets.

**Figure 4:**
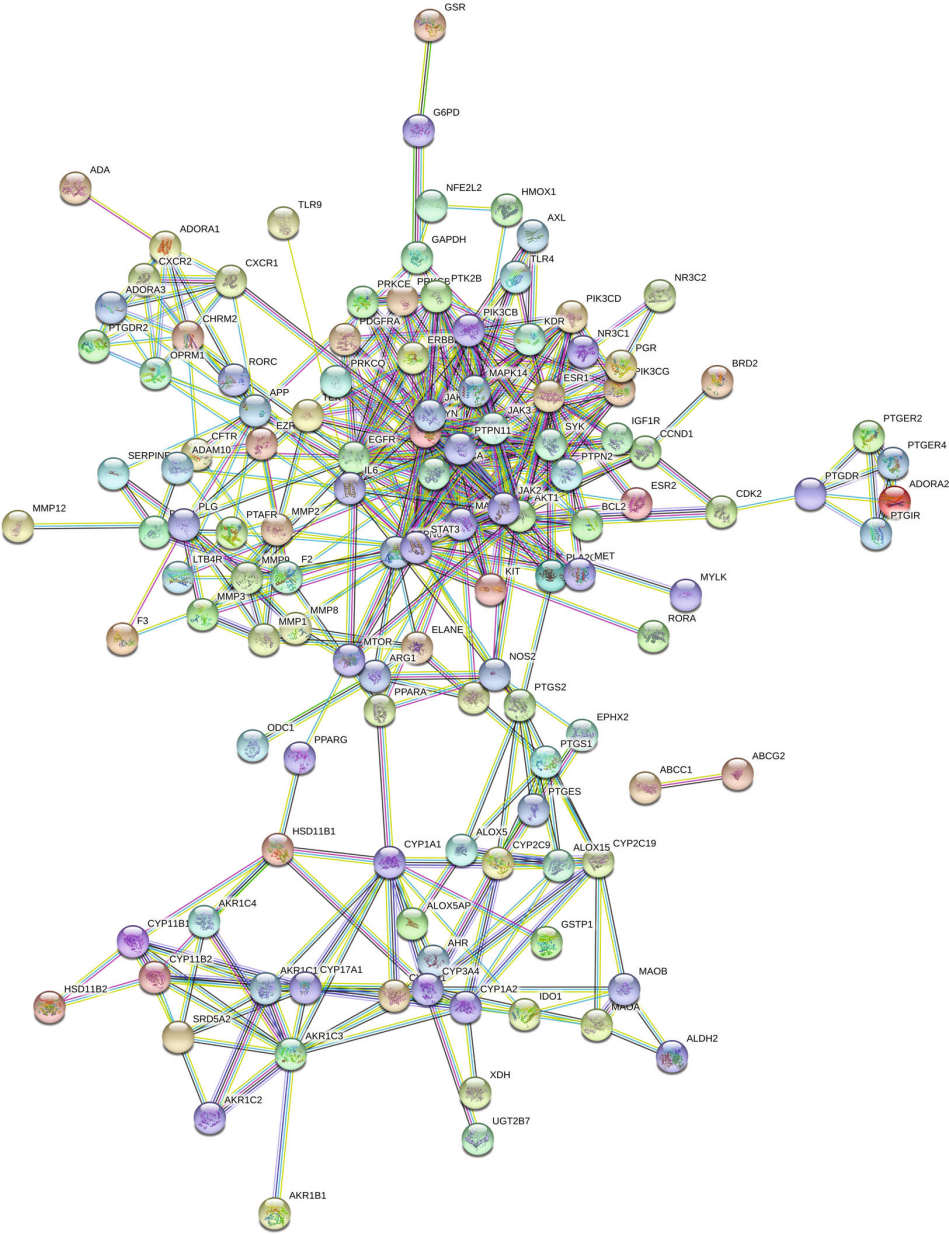
PPI network of *H. cuspidatus* in the treatment of asthma. This PPI network was directly obtained from STRING 11.0. Each node represents all the proteins produced by a single, protein-coding gene locus. The colored nodes are query proteins and the first shell of interactors, and the nodes are filled with some known or predicted 3D structures. The edges represent associations between the two proteins, including known and predicted intersections, with the known intersections derived from different databases and experimental data.

**Figure 5:**
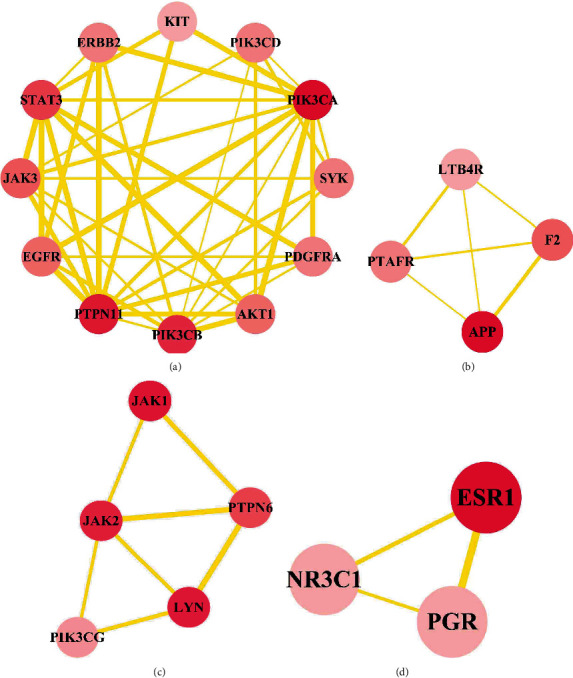
Closely connected submodules in the PPI network of *H. cuspidatus*. Set parameters in MOCDE: degree cutoff is 2, node score cutoff is 5, *K*-score is 2, and max depth is 100. Four closely connected submodules are obtained. The degree of the targets in the submodules is indicated by the color shade of the node.

To further obtain the potential targets of *H. cuspidatus* treatment of asthma, we obtained the intersection of the predicted compound targets and the disease targets. As a result, 122 common targets were found and identified as potential therapeutic targets of *H. cuspidatus* treatment of asthma (Figure 3). These 122 targets were used to construct the PPI network, which included 122 nodes and 428 edges (Figure 4). Moreover, the coexpression, experimentally determined interaction, and combined score among the targets of PPI network are presented in Supplementary Table 5. Among these targets, PIK3CA, STAT3, MAPK1, PTPN11, and EGFR demonstrated more interactions, revealing the need for further analysis of the interplay between these targets. To more precisely analyze the mechanism of action of *H. cuspidatus* treatment of asthma, we first identified the intrinsic core of the PPI network and then analyzed the characteristics of the core network. The closely connected network relationships were analyzed by the MCODE tool, and four key submodules were obtained with an MCODE score over three. There were 12 nodes in submodule 1 with the highest MCODE score of 7.273, where PIK3CA interacted with 11 other nodes (Figure 5(a)); submodule 2 had a total of four nodes, scoring 4 (Figure 5(b)); submodule 3 had a total of five nodes, with a score of 3.5 (Figure 5(c)); and submodule 4 had a total of three nodes and a score of 3 (Figure 5(d)). According to the *P*-value, the top three biological processes in the four submodules were respectively retained, and their functions are listed in Table 2. The genes in these core networks were mostly enriched in pathways associated with inflammation and kinase activities, including inflammatory response, immune system development, positive regulation of cell migration, and regulation of T cell activation, suggesting potential molecular mechanisms underlying *H. cuspidatus* treatment of asthma.

### 3.4. GO Function Enrichment and KEGG Pathway Analyses

**Figure 6:**
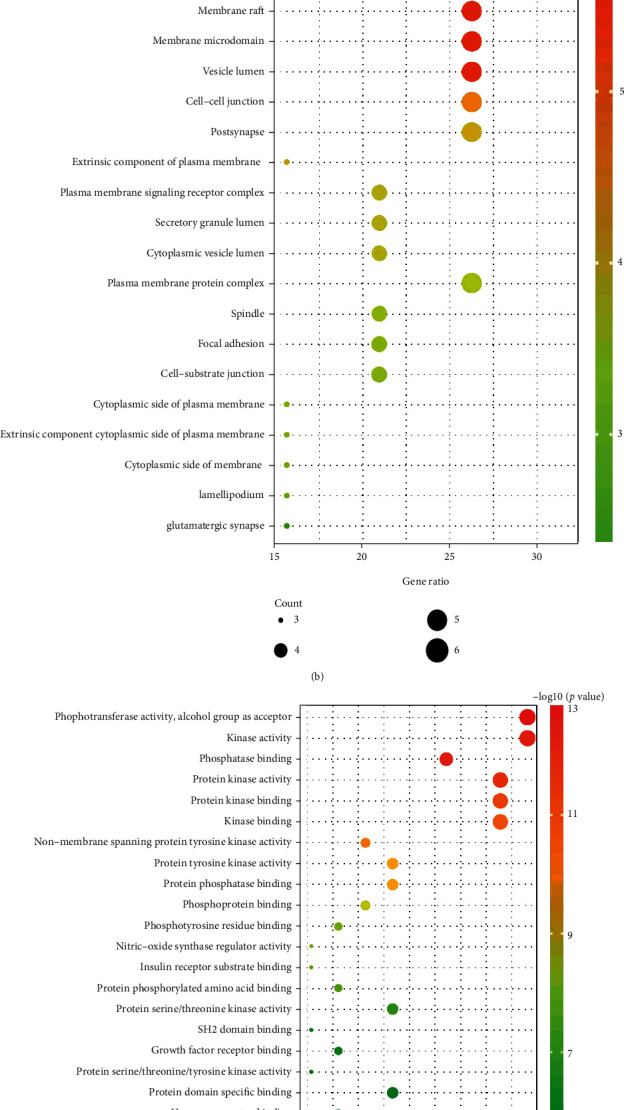
GO function enrichment and KEGG pathway analysis of key targets predicted for *H. cuspidatus* treatment of asthma. GO and KEGG pathway enrichment analyses were used to investigate the potential functions of key targets. (a) Biological processes (BP); (b) Cellular component (CC); (c) Molecular function (MF); (d) KEGG pathways. The bubble size represents the target number enriched in each entry, and the color represents the enrichment significance based on the corrected *P* value.

Among the 122 potential targets within the PPI network, 19 key targets were obtained after the degree was empirically set to be greater than twice the median. After searching and analyzing the raw data from the GEO database, the result showed that all 19 key targets were differentially expressed in asthma (Supplementary Table 6). Moreover, these key targets had different expression levels in peripheral blood mononuclear cells, nasal epithelia, bronchial epithelia, bronchoalveolar lavage, sputum, airway epithelia, airway fibroblasts, and distal lung fibroblasts of asthmatic patients compared with healthy participants. Overall, 19 key targets also showed high tissue distribution in the lung using the TISSUES (https://tissues.jensenlab.org) database (Supplementary Table 7). These key targets were further used in GO and KEGG enrichment analyses on the Metascape platform. The results yielded a total of 710 GO terms, including 655 biological processes, 24 cellular components, and 31 molecular functions. In addition, 93 signaling pathways were enriched by KEGG analysis. The top 20 pathways were selected and used to draw a bubble chart according to *P*-value (Figure 6). The main biological processes identified were peptidyl-tyrosine phosphorylation, peptidyl-tyrosine modification, phosphatidylinositol 3-kinase signaling, platelet activation, regulation of MAPK cascade, and activation of protein kinase activity (Figure 6(a)). GO annotations enriched in the CC category included “receptor complex,” “membrane components,” “vessel lumen,” “cell-cell junction,” and “postsynapse” (Figure 6(b)). GO annotations enriched in the MF category included “phosphotransferase activity,” “phosphatase binding,” “protein kinase activity,” and “protein tyrosine kinase activity” (Figure 6(c)). The signaling pathways enriched for asthma in the KEGG pathway analysis included the EGFR tyrosine kinase inhibitor resistance, Jak-STAT, PI3K-Akt, and Fc epsilon RI signaling pathways (Figure 6(d)).

### 3.5. Construction of the Active C-T-P Network

**Figure 7:**
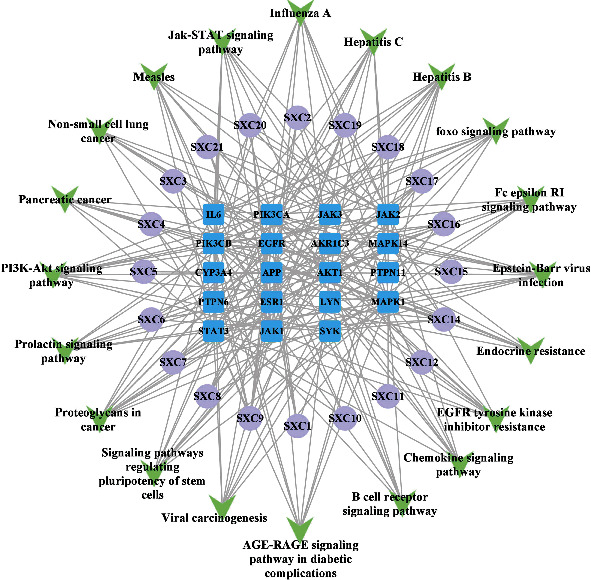
The “active compound-target-pathway” network. The blue round-rectangular node represents the potential target, the purple oval node represents the active component, and the green V-shaped node represents the twenty most enriched pathways.

To understand the relationship between the signaling pathways, candidate targets, and active components, an active C-T-P network was constructed using Cytoscape (3.8.2) software. In total, 59 nodes (20 compounds, 19 targets, and 20 pathways) and 250 edges were obtained (Figure 7). The topological parameters of *H. cuspidatus* in this C-T-P network describing the treatment of asthma were then analyzed by Network Analyzer in Cytoscape (3.8.2) software. The core components, targets, and pathways are illustrated in Table 3. For the active compounds, the degree, betweenness centrality, and closeness centrality values of caffeic acid, methyl rosmarinate, 8-hydroxycirsi-maritin, luteolin, and esculetin were higher than the average values of the other active compounds. For the targets, the degree, betweenness centrality, and closeness centrality values of 19 targets (including PIK3CA, AKT1, PIK3CB, MAPK1, STAT3, EGFR, ESR1, JAK1/2/3, and SYK) were closely related to the antiasthmatic effects of *H. cuspidatus*. For the pathways, caffeic acid was predicted to be the main active component in *H. cuspidatus* treatment of asthma (degree, 10; betweenness centrality, 0.08955; closeness centrality, 0.5043). The pathways affected included the Jak-STAT, EGFR tyrosine kinase inhibitor resistance, Chemokine, foxo, Fc epsilon RI, and PI3K-Akt signaling pathways. Methyl rosmarinate (degree, 8; betweenness centrality, 0.02733; closeness centrality, 0.4715) also shared the same targets (STAT3, PIK3CB, MAPK1, ESR1, SYK, and EGFR) and identical asthma-related signaling pathways with caffeic acid. 8-Hydroxycirsimaritin and luteolin demonstrated similar values of degree, betweenness centrality, and closeness centrality, and also similar potential targets. Esculetin demonstrated a degree of 5, a betweenness centrality of 0.01679, and a closeness centrality of 0.4427. The asthma-related pathways of the top five compounds were all identical.

### 3.6. Construction of Key Target-miRNA Interaction Network

**Figure 8:**
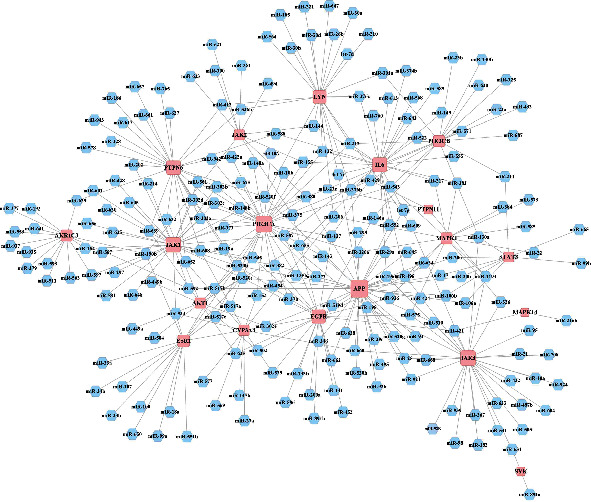
“Key target-miRNA” interaction network for the main components of *Hyssopus cuspidatus* Boriss. The pink round-rectangular node represents the key targets, and the blue hexagonal node represents the miRNAs. The degree of the key target is shown by the size of nodes.

To further explore the upstream miRNAs regulating these key targets, we obtained 212 miRNAs interacting with 19 key targets through the BisoGenet plug-in in Cytoscape. Using the 19 key targets within the PPI network and 212 associated miRNAs, a key target-miRNA interaction network was constructed with 231 nodes and 340 edges (Figure 8 and Supplementary Table 8). From this network, the top mRNA-miRNA pairs were identified. For example, EGFR, PIK3CA, JAK1, ESR1, PTPN6, and APP were regulated by miR-520b; AKT1, PIK3CA, JAK1, PTPN6, APP, EGFR, and ESR1 were regulated by miR-520e; and MAPK1, IL-6, APP, EGFR, and JAK1 were regulated by miR-498.

### 3.7. Construction of the Active C-T-M Network

**Figure 9:**
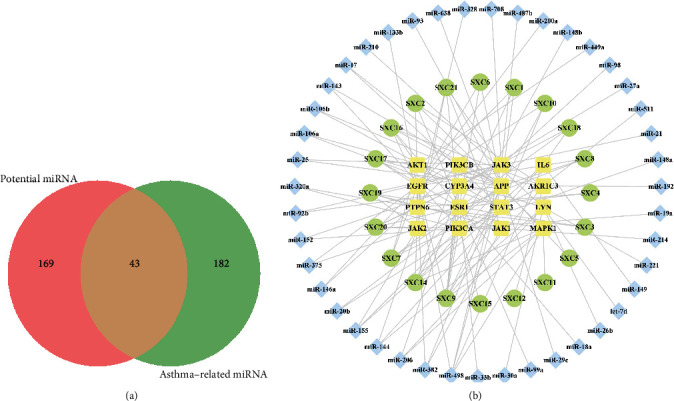
Construction of the “active compound-key target-miRNA” network. (a) Venn diagram of the common miRNAs. Red and yellow areas show the number of potential miRNAs predicted by key targets. Green and yellow areas show the number of asthma-related miRNAs. The intersections (yellow areas) indicate the number of common miRNAs. (b) “Active compound-key target-miRNA” network. The yellow round-rectangular node represents the key targets, the green oval node represents the active ingredients, and the blue rhombic node represents the miRNAs.

In total, 225 miRNAs were found to be differentially expressed in asthma following a review of the relevant literature over the last three years (Supplementary Table 9). The intersection between these 225 miRNAs and the 212 miRNAs predicted using the key targets was then evaluated. As a result, 43 intersection miRNAs were obtained by Venny 2.1.0 (Figure 9(a)). Subsequently, we identified whether these 43 miRNAs were altered in asthma in the GEO database. The results showed that all 43 miRNAs were aberrantly altered in both bronchial epithelia and sputum of asthmatic patients (Supplementary Table 10). To better reveal the relationship between miRNAs, key targets, and active components, an active C-T-M network was built using Cytoscape (3.8.2). The C-T-M network revealed that 16 key targets and 20 active compounds interacted with 43 miRNAs that contributed to the antiasthmatic effects of *H. cuspidatus* (Figure 9(b)). According to the degree, betweenness centrality, and closeness centrality values of this network (in descending order), caffeic acid was the most active compound (degree, 9; betweenness centrality, 0.2136; closeness centrality, 0.4286). Furthermore, caffeic acid interacted with the APP, ESR1, EGFR, PIK3CA, AKR1C3, MAPK1, STAT3, CYP3A4, and PIK3CB mRNAs. The miRNAs potentially affected by the 16 targets connected the 20 active compounds to miR-498, miR-155, miR-206, miR-382, miR-320a, miR-146a, miR-375, miR-152, miR-92b, miR-25, and miR-106a. Information on the active compounds interacting with miRNAs and key targets of *H. cuspidatus* in the treatment of asthma are summarized in Table 4.

## 4. Discussion

*H. cuspidatus* is a Chinese herbal medicine with significant antiasthmatic effects. In clinical patients with asthma, the Chinese patent medicines, Hanchuanzupa granules and Luoou Kezupa, both principally composed of *H. cuspidatus*, relieve the symptoms of asthma and are considered an effective treatment [20, 21]. Modern pharmacological research has revealed that *H. cuspidatus* suppresses airway inflammation, eliminates phlegm, and reduces cough (in addition to its antioxidative, antitumor, and antibacterial properties) [22, 23]. In our previous studies, ethanol extracts of *H. cuspidatus* were effective against bronchial asthma in various *in vivo* and *ex vivo* models [15]. Moreover, we revealed potential underlying mechanisms involved in alleviating airway hyperreactivity and inhibiting immune-inflammatory reactions. However, the multi-compound, multi-target, and multi-pathway effects of *H. cuspidatus* remain elusive. To investigate these unknowns, we systematically explored the potential mechanisms of *H. cuspidatus* action against asthma via the network pharmacology method.

The whole *H. cuspidatus* herb contains many bioactive compounds with considerable molecular diversity. A total of 23 active compounds met the ADME pharmacokinetic parameters. These included five flavonoids, eight terpenoids, four esters, three phenolic acids, one coumarin, one lignan, and one glycoside [11, 24–26]. Caffeic acid, methyl rosmarinate, luteolin, and esculetin were identified as active components with favorable drug-likeness and were discovered in both the C-T-P and C-T-M networks. Among these components, caffeic acid might be recognized as an essential active compound in *H. cuspidatus* containing multiple targets, including STAT3, EGFR, and PIK3CA, that contributed to treating asthma. In agreement with our findings, caffeic acid has previously been reported to be a potentially effective therapy for asthma, possessing various immunomodulatory, anti-inflammatory, and antioxidative effects via the MAPK, cyclooxygenase-2, NF-*κ*B, and AMPK pathways [27–29]. Methyl rosmarinate is also known to be present in several Chinese herbal medicines used for asthma treatment [30, 31]. Luteolin has high medical value for its antioxidant, anti-inflammatory, antitumor, and enhancement of immunity functions [32, 33]. Recently, luteolin has been demonstrated to protect lung tissues against ovalbumin-induced inflammation in mice by activation of the PI3K/Akt/mTOR pathway and inhibition of the Beclin-1-PI3KC3 complex [34]. Esculetin alleviates the progression of asthma in several experimental asthmatic models, where its antiasthmatic effect corrects mitochondrial dysfunction and attenuates Th2/Th17 responses [35, 36]. Although these different compounds may be associated with multiple biological functions, they were identified to act in common signaling pathways. For example, the targets of both caffeic acid and methyl rosmarinate shared the same pathways, including the Jak-STAT, EGFR tyrosine kinase inhibitor resistance, and PI3K-Akt signaling pathways. Therefore, the active components of *H. cuspidatus* likely exert their therapeutic effects through synergistic action against multiple common targets.

After analyzing the raw data acquired from the GEO database, all 19 key targets were differentially expressed in different samples (including peripheral blood mononuclear cells, nasal epithelia, bronchial epithelia, bronchoalveolar lavage, sputum, airway epithelia, airway fibroblasts, and distal lung fibroblasts) of asthma patients based on the comparison with healthy participants. To identify the targets of active compounds of *H. cuspidatus* in treating asthma, the involved gene function and signaling pathways were the first to be predicted. The results indicated that 93 signaling pathways were influenced by the active compounds of *H. cuspidatus*. The most significantly enriched signaling pathways in the *H. cuspidatus* treatment of asthma were associated with mechanisms of inflammatory reactions, immune pathways, oxidative stress, and various kinase activities and receptor binding activities. GO results revealed that *H. cuspidatus* may ameliorate asthma by regulating 655 categories of BPs, including regulation of phosphatidylinositol 3-kinase signaling, MAPK cascade, and activation of protein kinase activity; 31 MFs, including phosphotransferase activity, phosphatase binding, and protein kinase activity; and 24 CCs, including receptor complex, membrane components, and cell-cell junction.

To further accurately explore the potential mechanisms of *H. cuspidatus* treatment of asthma, the C-T-P and C-T-M network analyses were conducted by importing predicted targets into the Cytoscape software to construct relationships between proteins and obtain key targets of *H. cuspidatus* in the context of antiasthma. In the C-T-P and C-T-M networks, EGFR, JAK3, STAT3, IL-6, and LYN were the core targets. EGFR has been recognized as a key pathogenic factor and potential therapeutic target in asthma, and its expression increases in response to sustained airway inflammation, leading to epithelial damage in asthmatic airway epithelium. Furthermore, inhibition of EGFR activation may improve airflow limitation and abnormal mucus production [37]. Therefore, the EGFR tyrosine kinase inhibitory resistance pathway may provide a useful biomarker for *H. cuspidatus* asthma treatment efficacy. In clinical research, the JAK3 inhibitor tofacitinib has been widely used to treat a variety of diseases, and tofacitinib demonstrates potential as an alternative therapy for asthma [38]. JAK3 is an upstream signaling pathway of STATs, whose activation promotes the expression of STATs, an airway inflammatory target involved in the pathogenesis and development of asthma [39]. Our pathway analysis and the C-T-P network both reveal that the JAK/STAT signaling pathway may be an important pathway in the *H. cuspidatus* treatment of asthma. The JAK/STAT pathway is also involved in the signal transduction of cytokines (e.g., IL-2 and IL-15) that affect the differentiation of Th cells. A JAK inhibitor (LAS194046) administered via inhalation reduces allergen-induced late asthmatic response by inhibition of STAT1, STAT3, and STAT6 phosphorylation [40]. Thus, targeting the JAK/STAT pathway by *H. cuspidatus* is a plausible explanation for its therapeutic action against asthma.

With regard to other core targets involved in the networks for *H. cuspidatus*, IL-6 is a pleiotropic cytokine produced by various cell types and acts as a promoter of cell stress response or cell injury [41]. Clinical studies have revealed that the presence of IL-6 in the pulmonary airways is associated with impaired lung function in asthmatics of various types. As the JAK2/STAT3 pathway is preferentially activated by IL-6 [42], prevention of IL-6/JAK2/STAT3 signaling may be associated with the anti-inflammatory effects of *H. cuspidatus* treatment of asthma. In addition, LYN is a central effector of mucus hypersecretion and endoplasmic reticular stress in asthma [43] and participates in the regulation of airway inflammation, airway remodeling, and airway hyperresponsiveness through multiple mechanisms, thereby exacerbating asthma [44]. Therefore, suppression of LYN could be a possible target for asthma treatment by *H. cuspidatus*.

To identify potential miRNAs involved in the antiasthmatic activities of *H. cuspidatus* and to understand their regulation of antiasthmatic genes by *H. cuspidatus*, the C-T-M network was constructed. A total of 43 miRNAs that interacted with 16 target mRNAs were identified and correlated with the antiasthmatic effects of *H. cuspidatus*. Based on the comparison of RNA sequencing and microarray datasets from GEO, all 43 miRNAs were aberrantly changed in the bronchial epithelium and sputum of patients with asthma, implicating these in the occurrence and development of asthma. Of these 43 miRNAs, the regulation mechanisms of 15 miRNAs involved in asthma have previously been studied in depth using *in vivo* and *in vitro* models [45–48]. These are miR-155, miR-146a, miR-206, miR-375, miR-106a, miR-20b, miR-19a, miR-221, miR-30a, miR-98, miR-21, miR-149, miR-33b, miR-200a, and miR-27a. Among them, miR-155 overexpression is known to be involved in the development of allergic asthma [49] and was identified as a noninvasive biomarker in serology for the diagnosis of asthma and its severity [50]. Clinical trials revealed that both miR-155 and miR-498 levels increased in the nasal mucosa of asthmatics. Moreover, there was a correlation between the expression of miR-155 and miR-498, suggesting that these two miRNAs have similar regulation or similar function [51]. miR-206 expression is significantly increased in the peripheral blood of asthmatics, is potentially involved in airway smooth muscle innervation, and was identified as a molecular marker for asthma attacks [52, 53]. The remaining 28 miRNAs were aberrantly expressed in the serum, plasma, and cells of asthmatics (compared with the control group) [54, 55], suggesting that additional exploration of the potential regulatory roles of miRNAs in the pathology of this disease may be required. Therefore, the initial identification of miRNA profiling of *H. cuspidatus* in the treatment of asthma may help us investigate the upstream target of *H. cuspidatus* and provide potential biomarkers in response to the therapeutic effects of this herbal medicine.

From the perspective of mRNA targets, 16 mRNAs (including EGFR, JAK1/2/3, STAT3, MAPK1, IL-6, AKT1, PIK3CA, and PIK3CB) are controlled by more than one miRNA or share a common miRNA within the C-T-M network. For example, MAPK1 is a common gene in the inflammatory pathway, EGFR pathway, and PI3K/Akt pathway, and is controlled by miR-206, miR-320a, and miR-498. Hence, multi-pathway cooperativity involving MAPK1 may be important in asthma pathogenesis. Interestingly, MAPK1, EGFR, JAK3, and IL-6 all share the same binding miRNA, miR-498. Therefore, interactions involving these genes may play a synergetic role in multiple cascade events in asthma.

From the perspective of the active compounds, individual compounds affected multiple miRNAs in the C-T-M network, reflecting the multiple actions of *H. cuspidatus*. Caffeic acid interacted with the largest number of miRNAs (29 in total). These 29 miRNAs participate in oxidation, inflammation, T-cell responses, autophagic pathways, and airway hyperreactivity in asthma [45, 49, 56–58] via their PI3K/Akt, JAK/STAT, MAPK1, EGFR, and ESR1 targets. 8-Hydroxycirsimaritin and methyl rosmarinate interacted with 21 miRNAs, regulating inflammation and apoptosis by targeting PIK3CB, MAPK1, STAT3, EGFR, and AKT (according to the topological parameters). In total, 15 compounds, including caffeic acid, luteolin, esculetin, chrysin, and methyl rosmarinate, cooperatively interacted with miR-99a, contributing to NF-*κ*B-induced inflammatory cytokine production, T-cell activation, and allergen-driven exacerbations of airway disease [59–61]. Notably, maslinic acid and 20*β*-hydroxyursolic acid interact with the same binding miRNAs (miR-33b, miR-18a, miR-99a, and miR-328) and mRNA targets (ESR1 and PTPN6). These targets are associated with airway hyperresponsiveness, lung function decline, and inflammation of asthmatic airway epithelial cells [62, 63]. Therefore, the establishment of complex compound-miRNA-target axes may provide an explanation of the multiple targeting therapy of *H. cuspidatus* against asthma, reflecting the disease specificity and drug cooperativity.

Furthermore, there are several limitations in this study. First, the bioactive ingredients were identified from existing databases and literature and not by liquid chromatography, mass spectrometry, or other methods for detecting drug ingredients. Second, the potential miRNAs, key targets, and pathways identified by network topology analysis should be validated, and this will be our future research direction.

## 5. Conclusions

In conclusion, a total of 23 active compounds, 19 key targets, and 43 miRNAs associated with the *H. cuspidatus* treatment of asthma were identified by network analysis. The pathway, C-T-P, and C-T-M network analyses suggest that the principal active compounds of *H. cuspidatus*, including caffeic acid, luteolin, esculetin, methyl rosmarinate, and 8-hydroxycirsimaritin, play a critical role in the treatment of asthma by regulating multiple miRNAs, including miR-99a, miR-498, miR-33b, and miR-18a, that target mRNAs, including PI3K, JAK, MAPK1, EGFR, and ESR1, in the immune-inflammatory, oxidative stress, and various kinase activities and receptor-binding pathways. These findings provide evidence to support further studies of *H. cuspidatus* treatment of asthma.

## Figures and Tables

**Table 1:** Active compounds of *H. cuspidatus* and corresponding drug-likeness information.

Number	PubChem CID	Compound	Drug-likeness
SXC1	5281607	Chrysin	Five “yes”
SXC2	689075	Methyl caffeate	Five “yes”
SXC3	426384660	20*β*-Hydroxyursolic acid	Two “yes”
SXC4	73659	Maslinic acid	Two “yes”
SXC5	N/A	3′-Methoxy-vittarilide-B	Five “yes”
SXC6	188323	Cirsimaritin	Five “yes”
SXC7	N/A	8-Hydroxycirsimaritin	Five “yes”
SXC8	161271	Salvigenin	Five “yes”
SXC9	689043	Caffeic acid	Four “yes”
SXC10	445858	Ferulic acid	Four “yes”
SXC11	3084296	Eugenyl glucoside	Five “yes”
SXC12	10393	2-(4-Hydroxyphenyl)ethanol	Three “yes”
SXC13	637520	Methyl cinnamate	Five “yes”
SXC14	6479915	Methyl rosmarinate	Four “yes”
SXC15	181681	Medioresinol	Five “yes”
SXC16	5280445	Luteolin	Five “yes”
SXC17	5281416	Esculetin	Three “yes”
SXC18	N/A	2*α*,3*β*,24-Trihydroxy-12-en-28-ursolic acid	Three “yes”
SXC19	101928774	3-Eudesmene-1*β*-11-diol	Five “yes”
SXC20	11356850	4-Eudesmene-1*β*,11-diol	Five “yes”
SXC21	44566705	Caryolane-1,9*β*-diol	Five “yes”
SXC22	92231	Spathulenol	Four “yes”
SXC23	14312736	4*β*,10*α*-Aromadendranediol	Five “yes”

**Table 2:** Functional description of closely connected submodules in the PPI network.

Submodule	GO	Description	Log_10_*P*
Submodule 1	GO:0007169	Transmembrane receptor protein tyrosine kinase signaling pathway	-22.5692
Submodule 1	GO:0007167	Enzyme-linked receptor protein signaling pathway	-20.4625
Submodule 1	GO:0048015	Phosphatidylinositol-mediated signaling	-12.8317
Submodule 2	GO:0034765	Regulation of ion transmembrane transport	-4.7945
Submodule 2	GO:0006954	Inflammatory response	-4.7391
Submodule 2	GO:0034762	Regulation of transmembrane transport	-4.5566
Submodule 3	GO:0018108	Peptidyl-tyrosine phosphorylation	-8.5838
Submodule 3	GO:0018212	Peptidyl-tyrosine modification	-8.5482
Submodule 3	GO:0006468	Protein phosphorylation	-8.1814
Submodule 4	GO:0030518	Intracellular steroid hormone receptor signaling pathway	-8.2926
Submodule 4	GO:0043401	Steroid hormone-mediated signaling pathway	-7.8685
Submodule 4	GO:0009755	Hormone-mediated signaling pathway	-7.1708

**Table 3:** Characteristic parameters in the active C-T-P network.

Number	Compound	Degree	Betweenness centrality	Closeness centrality	Target	Asthma-related pathway
SXC9	Caffeic acid	10	0.08955	0.5043	STAT3, PIK3CB, MAPK1, PIK3CA, ESR1, SYK, AKR1C3, EGFR, CYP3A4, APP	Jak-STAT signaling pathway, EGFR tyrosine kinase inhibitor resistance, chemokine signaling pathway, foxo signaling pathway, Fc epsilon RI signaling pathway, PI3K-Akt signaling pathway
SXC14	Methyl rosmarinate	8	0.02733	0.4715	STAT3, PIK3CB, MAPK1, ESR1, SYK, MAPK14, EGFR, APP	
SXC7	8-Hydroxycirsimaritin	6	0.02058	0.4567	AKT1, ESR1, SYK, AKR1C3, EGFR, APP	
SXC16	Luteolin	6	0.02058	0.4567	AKT1, ESR1, SYK, AKR1C3, EGFR, APP	
SXC17	Esculetin	5	0.01679	0.4427	AKT1, ESR1, AKR1C3, EGFR, LYN	
SXC19	3-Eudesmene-1*β*-11-diol	5	0.08109	0.4173	JAK3, JAK2, ESR1, JAK1, PTPN6	PI3K-Akt signaling pathway, Jak-STAT signaling pathway, chemokine signaling pathway, EGFR tyrosine kinase inhibitor resistance
SXC20	4-Eudesmene-1*β*,11-diol	5	0.08109	0.4173		
SXC2	Methyl caffeate	4	0.01056	0.4234	MAPK1, ESR1, AKR1C3, EGFR	Fc epsilon RI signaling pathway, PI3K-Akt signaling pathway, EGFR tyrosine kinase inhibitor resistance, chemokine signaling pathway, foxo signaling pathway
SXC18	2*α*,3*β*,24-Trihydroxy-12-en-28-ursolic acid	4	0.006048	0.3841	IL-6, ESR1, PTPN11, PTPN6	PI3K-Akt signaling pathway, Jak-STAT signaling pathway, EGFR tyrosine kinase inhibitor resistance, foxo signaling pathway
SXC1	Chrysin	4	0.005162	0.3946	ESR1, SYK, EGFR, APP	Fc epsilon RI signaling pathway, PI3K-Akt signaling pathway, EGFR tyrosine kinase inhibitor resistance, foxo signaling pathway
SXC6	Cirsimaritin	4	0.005162	0.3946		
SXC8	Salvigenin	4	0.005162	0.3946		

**Table 4:** Information on the active C-T-M network of *H. cuspidatus* in the treatment of asthma.

Active compound	Number	miRNA	Key target
Chrysin	SXC1	miR-17, miR-93, miR-638, miR-25, miR-106b, miR-92b, miR-29c, miR-106a, miR-382, miR-143, miR-20b, miR-498, miR-33b, miR-18a, miR-99a, miR-375, miR-133b, miR-146a, miR-200a	APP, ESR1, EGFR
Methyl caffeate	SXC2	miR-33b, miR-18a, miR-99a, miR-375, miR-133b, miR-146a, miR-200a, miR-382, miR-498, miR-511, miR-192, miR-206, miR-320a	ESR1, EGFR, AKR1C3, MAPK1
20*β*-Hydroxyursolic acid	SXC3	miR-33b, miR-18a, miR-99a, miR-328	ESR1, PTPN6
Maslinic acid	SXC4	miR-33b, miR-18a, miR-99a, miR-328	ESR1, PTPN6
3′-Methoxy-vittarilide-B	SXC5	miR-17, miR-93, miR-638, miR-25, miR-106b, miR-92b, miR-29c, miR-106a, miR-382, miR-143, miR-20b, miR-498	APP
Cirsimaritin	SXC6	miR-17, miR-93, miR-638, miR-25, miR-106b, miR-92b, miR-29c, miR-106a, miR-382, miR-143, miR-20b, miR-498, miR-33b, miR-18a, miR-99a, miR-375, miR-133b, miR-146a, miR-200a	APP, ESR1, EGFR
8-Hydroxycirsimaritin	SXC7	miR-17, miR-93, miR-638, miR-25, miR-106b, miR-92b, miR-29c, miR-106a, miR-382, miR-143, miR-20b, miR-498, miR-33b, miR-18a, miR-99a, miR-375, miR-133b, miR-146a, miR-200a, miR-511, miR-192	APP, ESR1, EGFR, AKR1C3, AKT1
Salvigenin	SXC8	miR-17, miR-93, miR-638, miR-25, miR-106b, miR-92b, miR-29c, miR-106a, miR-382, miR-143, miR-20b, miR-498, miR-33b, miR-18a, miR-99a, miR-375, miR-133b, miR-146a, miR-200a, miR-382	APP, ESR1, EGFR
Caffeic acid	SXC9	miR-17, miR-93, miR-638, miR-25, miR-106b, miR-92b, miR-29c, miR-106a, miR-382, miR-143, miR-20b, miR-498, miR-33b, miR-18a, miR-99a, miR-375, miR-133b, miR-146a, miR-200a, miR-511, miR-192, miR-148a, miR-320a, miR-152, miR-155, miR-19a, miR-148b, miR-206, miR-27a	APP, ESR1, EGFR, PIK3CA, AKR1C3, MAPK1, STAT3, CYP3A4, PIK3CB
Ferulic acid	SXC10	miR-17, miR-93, miR-638, miR-25, miR-106b, miR-92b, miR-29c, miR-106a, miR-382, miR-143, miR-20b, miR-498, miR-375, miR-133b, miR-146a, miR-200a	APP, EGFR, STAT3
Eugenyl glucoside	SXC11	miR-206, miR-320a, miR-498	MAPK1
2-(4-Hydroxyphenyl)ethanol	SXC12	miR-511, miR-192	AKR1C3
Methyl rosmarinate	SXC14	miR-17, miR-93, miR-638, miR-25, miR-106b, miR-92b, miR-29c, miR-106a, miR-382, miR-143, miR-20b, miR-498, miR-33b, miR-18a, miR-99a, miR-375, miR-133b, miR-146a, miR-200a, miR-206, miR-320a	APP, ESR1, EGFR, MAPK1, STAT3, PIK3CB
Medioresinol	SXC15	miR-155, miR-19a, miR-143, miR-148b	PIK3CA
Luteolin	SXC16	miR-33b, miR-18a, miR-99a, miR-375, miR-133b, miR-146a, miR-200a, miR-382, miR-498	ESR1, EGFR, AKR1C3, AKT1
Esculetin	SXC17	miR-33b, miR-18a, miR-99a, miR-375, miR-133b, miR-146a, miR-200a, miR-382, miR-498, miR-511, miR-192, miR-206, miR-26b, miR-221, miR-144, miR-30a, miR-210, let-7d, miR-449a	ESR1, EGFR, AKR1C3, LYN, AKT1
2*α*,3*β*,24-Trihydroxy-12-en-28-ursolic acid	SXC18	miR-33b, miR-18a, miR-99a, miR-328, miR-149, miR-144, miR-155, miR-498	ESR1, PTPN6, IL6
3-Eudesmene-1*β*-11-diol	SXC19	miR-33b, miR-18a, miR-99a, miR-487b, miR-92b, miR-21, miR-498, miR-328, miR-375, miR-144, miR-155, miR-214, miR-382	ESR1, JAK3, PTPN6, JAK2, JAK1
4-Eudesmene-1*β*,11-diol	SXC20	miR-33b, miR-18a, miR-99a, miR-487b, miR-92b, miR-21, miR-498, miR-328, miR-375, miR-144, miR-155, miR-214, miR-382	ESR1, JAK3, PTPN6, JAK2, JAK1
Caryolane-1,9*β*-diol	SXC21	miR-33b, miR-18a, miR-99a, miR-487b, miR-92b, miR-21, miR-498, miR-375, miR-144, miR-155, miR-214, miR-382	ESR1, JAK3, JAK2, JAK1

## Data Availability

The data in this study that support the findings are available from the corresponding authors upon request.

## Supplementary Materials

Supplementary Materials Supplementary Table 1: Compounds of *H. cuspidatus* in the published literature. Supplementary Table 2: Information of “compound-target” network. Supplementary Table 3: Asthma-related genes obtained by searching the GeneCards, GeneMap, DisGeNET, OMIM, and DrugBank databases. Supplementary Table 4: One thousand six hundred and sixteen targets related to asthma after removing duplicate items. Supplementary Table 5: Node interactions in the PPI network. Supplementary Table 6: Information of key genes in *H. cuspidatus* treatment of asthma in the GEO database. Supplementary Table 7: Searching results of 19 key targets in the TISSUES database. Supplementary Table 8: Interactions in the “key target-miRNA” network. Supplementary Table 9: Differentially expressed miRNAs in asthma in published literature over the last three years. Supplementary Table 10: Information of the common miRNAs in the GEO database.
