# Supplementary material for: Exploration of the Molecular Mechanisms of Hyssopus cuspidatus Boriss Treatment of Asthma in an mRNA-miRNA Network via Bioinformatics Analysis
Source: Biomed Res Int. 2022 May 5;2022:7111901. doi: 10.1155/2022/7111901 (PMC9098316; doi:10.1155/2022/7111901)
Supplement: Supplementary Materials — Supplementary Table 1: Compounds of H. cuspidatus in the published literature. Supplementary Table 2: Information of “compound-target” network. Supplementary Table 3: Asthma-related genes obtained by searching the GeneCards, GeneMap, DisGeNET, OMIM, and DrugBank databases. Supplementary Table 4: One thousand six hundred and sixteen targets related to asthma after removing duplicate items. Supplementary Table 5: Node interactions in the PPI network. Supplementary Table 6: Information of key genes in H. cuspidatus treatment of asthma in the GEO database. Supplementary Table 7: Searching results of 19 key targets in the TISSUES database. Supplementary Table 8: Interactions in the “key target-miRNA” network. Supplementary Table 9: Differentially expressed miRNAs in asthma in published literature over the last three years. Supplementary Table 10: Information of the common miRNAs in the GEO database. [file 7111901.f1.doc]

Supplementary Table 1: Compounds of H. cuspidatus in the published literature.

| Compound | References |
| --- | --- |
| 20*β*-hydroxyursolic acid | [1] |
| 2*α*,3*β,*24-trihydroxy-12-en-28-ursolic acid | [1] |
| 3′-methoxy-vittarilide-B | [3] |
| 3-eudesmene-1*β*-11-diol | [4] |
| 3-*O*-Caffeoylquinic acid methyl ester | [2] |
| 4-eudesmene-1*β*,11-diol | [4] |
| 4-*O*-Caffeoylquinic acid methyl ester | [2] |
| 4*β*,10*α*-aromadendranediol | [2] |
| 4-(2-Hydroxyethyl)phenol | [3] |
| 8-hydroxycirsimaritin | [3] |
| Apigenin-4′-O-*β*-d-glucopyranoside | [3] |
| Caryolane-1,9*β*-diol | [4] |
| Glochidioboside [(7S,8R)-dihydrodehydrodiconiferyl alcohol 9′-O-*β*-d-glucopyranoside] | [2] |
| Oresbiusin A | [2] |
| Spathulenol | [4] |
| *β*-Sitosterol | [1, 5] |
| Ferulic acid | [3, 6] |
| Chrysin | [5] |
| Epmedin C | [5] |
| Eugenyl glucoside | [3] |
| 24-Methylenecycloartanol | [5] |
| Daucosterol | [2, 5] |
| Quercetin-3-*O*-*β*-d-glucopyranoside | [2, 3] |
| Cirsimaritin | [3] |
| Caffeic acid | [2, 3, 6] |
| Methyl caffeate | [7] |
| Cerotin | [5] |
| Cryptomeridiol | [4] |
| Linarin | [3] |
| Rosmarinic acid | [2, 3, 6-8] |
| Methyl rosmarinate | [3] |
| Luteolin | [2] |
| Luteolin 7-glucuronide | [7] |
| Medioresinol | [3] |
| Esculetin | [3] |
| Oleanic acid | [1, 5] |
| Methyl cinnamate | [3] |
| Salvigenin | [2, 3] |
| Maslinic acid | [1] |
| Diosmin | [7, 8] |
| Ursolic Acid | [1, 2] |
| Icariin | [2] |
| Epimedoside A | [2] |
| Hexadecane | [9] |
| 1-Hexadecanol | [9] |

**References**

[1] Y. Zhang, Y. Kang, J. He, X. Cai, and X. Wang, “Triterpenoids constituents of *Hyssopus cuspidatus*,” *Journal of Chinese Medicinal Materials*, vol. 43, no. 2, pp. 347-349, 2020.

[2] O. Shomirzoeva, J. Li, S. Numonov, S. Atolikshoeva, and H. A. Aisa, “Chemical components of *Hyssopus cuspidatus* Boriss.: isolation and identification, characterization by HPLC-DAD-ESI-HRMS/MS, antioxidant activity and antimicrobial activity,” *Nat Prod Res*, vol. 34, no. 4, 534-540, 2020.

[3] X. Cai, A. Maimaiti, X. Wang, and J. He, “Chemical Constituents of Anti-asthmatic Active Site from Overground Part of Hyssopus cuspidatus,” *Journal of Chinese Medicinal Materials*, no. 4, pp.848-852, 2021.

[4] D. Liu, X. Zhu, J. Xiang et al., “Sesquiterpenes from aerial parts of *Hyssopus cuspidatus*,” *Chinese Traditional and Herbal Drugs*, vol. 50, no. 5, pp. 1049-1054, 2019.

[5] P. Abulizi, Y. Cong, Y. Zhu, and R. Kasimu, “Studies on chemical constituents from *Hyssopus cuspidatus* Boriss,” *Chinese Journal of Modern Applied Pharmacy*, vol. 28, no. 8, pp. 735-737, 2011.

[6] L. Zhao, Z. Ji, K. Li, B. Wang, Y. Zeng, and S. Tian, “HPLC-DAD analysis of Hyssopus Cuspidatus Boriss extract and mensuration of its antioxygenation property,” *BMC Complement Med Ther*, vol. 20, no. 1, pp. 228, 2020.

[7] J. Zhao, F. Xu, J. He, W. Tan, Z. Gu, and L. Ma, “Study on Chemical constituents of Hyssopus cuspidatus,” *Journal of Chinese Medicinal Materials*, vol. 36, no. 1, pp. 54-57, 2013.

[8] F. Yuan, R. Liu, M. Hu et al., “JAX2, an ethanol extract of Hyssopus cuspidatus Boriss, can prevent bronchial asthma by inhibiting MAPK/NF-κB inflammatory signaling,” *Phytomedicine*, vol. 57, pp. 305-314, 2019.

[9] Y. Zhu, “Studies of chemical constituents and pharmacodynamics of *Hyssopus offcinalis*,” Xinjiang Medical University, 2009.

Supplementary Table 2: Information of “compound-target” network.

| Compound | Target |
| --- | --- |
| SXC1 | AKR1B1 |
| SXC1 | CDK5R1 |
| SXC1 | XDH |
| SXC1 | CYP19A1 |
| SXC1 | CA2 |
| SXC1 | CCNB3 |
| SXC1 | CA7 |
| SXC1 | CDK6 |
| SXC1 | CA1 |
| SXC1 | CA12 |
| SXC1 | CA9 |
| SXC1 | CA4 |
| SXC1 | ABCB1 |
| SXC1 | CYP1B1 |
| SXC1 | ABCG2 |
| SXC1 | CBR1 |
| SXC1 | TNKS2 |
| SXC1 | TNKS |
| SXC1 | ALOX15 |
| SXC1 | ALOX12 |
| SXC1 | ESR1 |
| SXC1 | ESR2 |
| SXC1 | HSD17B1 |
| SXC1 | ACHE |
| SXC1 | ADORA1 |
| SXC1 | ADORA2A |
| SXC1 | PARP1 |
| SXC1 | KDM4E |
| SXC1 | CDK1 |
| SXC1 | GRK6 |
| SXC1 | CSNK2A1 |
| SXC1 | NOX4 |
| SXC1 | MAOA |
| SXC1 | FLT3 |
| SXC1 | PTGS2 |
| SXC1 | SYK |
| SXC1 | GSK3B |
| SXC1 | ABCC1 |
| SXC1 | TTR |
| SXC1 | CFTR |
| SXC1 | AKR1B10 |
| SXC1 | LCK |
| SXC1 | IKBKB |
| SXC1 | NTRK2 |
| SXC1 | BCHE |
| SXC1 | ADORA3 |
| SXC1 | ALOX5 |
| SXC1 | PIM1 |
| SXC1 | PTPRS |
| SXC1 | EGFR |
| SXC1 | HSD17B2 |
| SXC1 | AR |
| SXC1 | AMY1A |
| SXC1 | APP |
| SXC1 | NAE1 |
| SXC1 | ARG1 |
| SXC1 | PFKFB3 |
| SXC1 | CALM1 |
| SXC1 | GLO1 |
| SXC1 | MMP9 |
| SXC1 | MMP2 |
| SXC1 | MMP12 |
| SXC1 | CD38 |
| SXC1 | TOP1 |
| SXC1 | TERT |
| SXC1 | AURKB |
| SXC1 | CYP1A1 |
| SXC1 | ESRRA |
| SXC1 | TYR |
| SXC1 | AHR |
| SXC1 | SLC22A12 |
| SXC1 | PRKDC |
| SXC1 | ST6GAL1 |
| SXC1 | PIK3CG |
| SXC1 | MAPT |
| SXC1 | TOP2A |
| SXC1 | INSR |
| SXC1 | MYLK |
| SXC1 | APEX1 |
| SXC1 | MET |
| SXC1 | OPRD1 |
| SXC1 | TBXAS1 |
| SXC1 | KIT |
| SXC1 | MAOB |
| SXC1 | PTPN1 |
| SXC1 | NOS2 |
| SXC1 | PLA2G2A |
| SXC1 | PLA2G4A |
| SXC1 | IGF1R |
| SXC1 | KDR |
| SXC1 | PLK1 |
| SXC1 | ALK |
| SXC1 | AXL |
| SXC1 | PDE5A |
| SXC1 | SRC |
| SXC1 | PLG |
| SXC1 | F2 |
| SXC1 | SIGMAR1 |
| SXC1 | CYP1A2 |
| SXC1 | GPR35 |
| SXC1 | CDK5 |
| SXC1 | CDK1 |
| SXC1 | CCNB1 |
| SXC1 | CCNB2 |
| SXC2 | CA2 |
| SXC2 | MMP2 |
| SXC2 | CA7 |
| SXC2 | CA1 |
| SXC2 | CA12 |
| SXC2 | CA14 |
| SXC2 | CA9 |
| SXC2 | MMP9 |
| SXC2 | CA6 |
| SXC2 | CA5B |
| SXC2 | CA5A |
| SXC2 | ALOX5 |
| SXC2 | AKR1B10 |
| SXC2 | MAOB |
| SXC2 | MMP1 |
| SXC2 | AKR1B1 |
| SXC2 | LCK |
| SXC2 | EGFR |
| SXC2 | PTPN1 |
| SXC2 | ERBB2 |
| SXC2 | CA4 |
| SXC2 | ESR2 |
| SXC2 | FYN |
| SXC2 | SLC6A2 |
| SXC2 | AKR1C3 |
| SXC2 | AKR1C4 |
| SXC2 | AKR1C2 |
| SXC2 | ESR1 |
| SXC2 | ADORA1 |
| SXC2 | TMIGD3 |
| SXC2 | CA3 |
| SXC2 | ADORA2A |
| SXC2 | PLAA |
| SXC2 | PTGS1 |
| SXC2 | HCAR2 |
| SXC2 | MAPK1 |
| SXC2 | CA13 |
| SXC2 | TTR |
| SXC2 | TLR4 |
| SXC2 | CBFB |
| SXC2 | CDK2 |
| SXC2 | ST6GAL1 |
| SXC3 | PTPN1 |
| SXC3 | POLB |
| SXC3 | CD81 |
| SXC3 | HSD11B1 |
| SXC3 | AKR1B10 |
| SXC3 | PTPN2 |
| SXC3 | PLA2G1B |
| SXC3 | CDC25B |
| SXC3 | RORC |
| SXC3 | PTPRF |
| SXC3 | ACP1 |
| SXC3 | PDE4D |
| SXC3 | PTGES |
| SXC3 | PTPN6 |
| SXC3 | TERT |
| SXC3 | NOS2 |
| SXC3 | FABP1 |
| SXC3 | NR3C2 |
| SXC3 | SCD |
| SXC3 | HSD11B2 |
| SXC3 | AR |
| SXC3 | PPARG |
| SXC3 | FNTA |
| SXC3 | ESR2 |
| SXC3 | CDC25A |
| SXC3 | CYP19A1 |
| SXC3 | PPARA |
| SXC3 | CES2 |
| SXC3 | PTPN11 |
| SXC3 | PTGS2 |
| SXC3 | FABP4 |
| SXC3 | FABP3 |
| SXC3 | FABP5 |
| SXC3 | PREP |
| SXC3 | PPARD |
| SXC3 | G6PD |
| SXC3 | CYP51A1 |
| SXC3 | PTGS1 |
| SXC3 | GRIK2 |
| SXC3 | NR1H4 |
| SXC3 | LTB4R |
| SXC3 | FAAH |
| SXC3 | NPC1L1 |
| SXC3 | SIGMAR1 |
| SXC3 | CYP17A1 |
| SXC3 | PRKCH |
| SXC3 | NR3C1 |
| SXC3 | SLC6A3 |
| SXC3 | ADORA3 |
| SXC3 | PTGER2 |
| SXC3 | ALOX5 |
| SXC3 | PGR |
| SXC3 | GPBAR1 |
| SXC3 | SERPINA6 |
| SXC3 | SHBG |
| SXC3 | HMGCR |
| SXC3 | TOP1 |
| SXC3 | NR1H3 |
| SXC3 | PLA2G4A |
| SXC3 | ALOX5AP |
| SXC3 | PTGER4 |
| SXC3 | MMP3 |
| SXC3 | MMP1 |
| SXC3 | MMP2 |
| SXC3 | ESR1 |
| SXC3 | SLC6A4 |
| SXC3 | AMPD2 |
| SXC3 | HSF1 |
| SXC3 | SAE1 |
| SXC3 | FNTB |
| SXC3 | UBA2 |
| SXC4 | PTPN1 |
| SXC4 | HSD11B1 |
| SXC4 | AKR1B10 |
| SXC4 | POLB |
| SXC4 | PTPN2 |
| SXC4 | PLA2G1B |
| SXC4 | CDC25B |
| SXC4 | PDE4D |
| SXC4 | RORC |
| SXC4 | PTPRF |
| SXC4 | ACP1 |
| SXC4 | CD81 |
| SXC4 | NOS2 |
| SXC4 | CES2 |
| SXC4 | PTGES |
| SXC4 | CDC25A |
| SXC4 | PTPN6 |
| SXC4 | FNTA |
| SXC4 | FABP1 |
| SXC4 | AR |
| SXC4 | SCD |
| SXC4 | PTGS2 |
| SXC4 | PPARG |
| SXC4 | HSD11B2 |
| SXC4 | PRKCH |
| SXC4 | PTPN11 |
| SXC4 | CYP19A1 |
| SXC4 | ESR2 |
| SXC4 | PPARA |
| SXC4 | PPARD |
| SXC4 | NR3C2 |
| SXC4 | FAAH |
| SXC4 | TERT |
| SXC4 | PREP |
| SXC4 | SERPINA6 |
| SXC4 | SHBG |
| SXC4 | G6PD |
| SXC4 | CYP51A1 |
| SXC4 | FABP4 |
| SXC4 | FABP3 |
| SXC4 | FABP5 |
| SXC4 | PTGS1 |
| SXC4 | LTB4R |
| SXC4 | SIGMAR1 |
| SXC4 | CYP17A1 |
| SXC4 | SLC6A4 |
| SXC4 | HMGCR |
| SXC4 | ADORA3 |
| SXC4 | PTGER2 |
| SXC4 | TLR9 |
| SXC4 | NR1H3 |
| SXC4 | TOP1 |
| SXC4 | TOP2A |
| SXC4 | NR3C1 |
| SXC4 | SRD5A2 |
| SXC4 | ALOX5AP |
| SXC4 | ALOX5 |
| SXC4 | SLC10A1 |
| SXC4 | PGR |
| SXC4 | SLC10A2 |
| SXC4 | BCHE |
| SXC4 | PTGER4 |
| SXC4 | GRIK1 |
| SXC4 | CHRM2 |
| SXC4 | SLC6A2 |
| SXC4 | GRIK2 |
| SXC4 | PTGDR2 |
| SXC4 | NR1H4 |
| SXC4 | ESR1 |
| SXC4 | FFAR1 |
| SXC4 | BACE1 |
| SXC4 | RORA |
| SXC4 | GPBAR1 |
| SXC4 | PTGIR |
| SXC4 | FNTB |
| SXC5 | APP |
| SXC5 | PYGL |
| SXC5 | MMP13 |
| SXC5 | MMP2 |
| SXC5 | MMP12 |
| SXC5 | AKR1B1 |
| SXC5 | SCN9A |
| SXC5 | HPRT1 |
| SXC5 | TLR4 |
| SXC5 | TTR |
| SXC5 | CXCR2 |
| SXC5 | PLA2G7 |
| SXC5 | SGK1 |
| SXC5 | ADAM17 |
| SXC5 | MMP14 |
| SXC5 | MMP7 |
| SXC5 | MMP8 |
| SXC5 | ADAM10 |
| SXC5 | AKR1B10 |
| SXC5 | TARS |
| SXC5 | KCNA3 |
| SXC5 | BMP1 |
| SXC5 | HTR6 |
| SXC5 | MMP9 |
| SXC5 | CTSV |
| SXC6 | AKR1B1 |
| SXC6 | ADORA1 |
| SXC6 | ADORA2A |
| SXC6 | ADORA3 |
| SXC6 | OPRD1 |
| SXC6 | ABCG2 |
| SXC6 | OPRM1 |
| SXC6 | KIT |
| SXC6 | PIM1 |
| SXC6 | ALOX5 |
| SXC6 | PTPRS |
| SXC6 | FLT3 |
| SXC6 | CYP1B1 |
| SXC6 | SYK |
| SXC6 | HSD17B1 |
| SXC6 | AMY1A |
| SXC6 | GRK6 |
| SXC6 | PLG |
| SXC6 | ESR2 |
| SXC6 | GSK3B |
| SXC6 | PTGS2 |
| SXC6 | ABCC1 |
| SXC6 | KDM4E |
| SXC6 | CDK6 |
| SXC6 | ESR1 |
| SXC6 | CDK1 |
| SXC6 | AKR1B10 |
| SXC6 | NOS2 |
| SXC6 | NOX4 |
| SXC6 | MMP9 |
| SXC6 | ABCB1 |
| SXC6 | HSD17B2 |
| SXC6 | CBR1 |
| SXC6 | MAOB |
| SXC6 | GLO1 |
| SXC6 | ARG1 |
| SXC6 | CDK5R1 |
| SXC6 | MAOA |
| SXC6 | CYP19A1 |
| SXC6 | TERT |
| SXC6 | MMP2 |
| SXC6 | MMP12 |
| SXC6 | ACHE |
| SXC6 | APP |
| SXC6 | CA2 |
| SXC6 | CA1 |
| SXC6 | CA9 |
| SXC6 | CSNK2A1 |
| SXC6 | CA7 |
| SXC6 | PARP1 |
| SXC6 | BACE1 |
| SXC6 | CCNB3 |
| SXC6 | ALOX15 |
| SXC6 | CA12 |
| SXC6 | AHR |
| SXC6 | AR |
| SXC6 | KDR |
| SXC6 | NAE1 |
| SXC6 | LCK |
| SXC6 | MET |
| SXC6 | TNKS2 |
| SXC6 | TNKS |
| SXC6 | CA4 |
| SXC6 | MMP13 |
| SXC6 | EGFR |
| SXC6 | PFKFB3 |
| SXC6 | ALOX12 |
| SXC6 | PTPN1 |
| SXC6 | GPR35 |
| SXC6 | TOP2A |
| SXC6 | PIK3R1 |
| SXC6 | CA3 |
| SXC6 | CAMK2B |
| SXC6 | NEK6 |
| SXC6 | PLA2G1B |
| SXC6 | APEX1 |
| SXC6 | NUAK1 |
| SXC6 | AKR1C1 |
| SXC6 | AKR1A1 |
| SXC6 | TAS2R31 |
| SXC6 | MPG |
| SXC6 | SLC22A12 |
| SXC6 | ODC1 |
| SXC6 | XDH |
| SXC6 | CDK2 |
| SXC6 | MMP3 |
| SXC6 | TOP1 |
| SXC6 | MCL1 |
| SXC6 | CALM1 |
| SXC6 | TTR |
| SXC6 | CFTR |
| SXC6 | ST6GAL1 |
| SXC6 | PLA2G2A |
| SXC6 | ALK |
| SXC6 | AURKB |
| SXC6 | PLK1 |
| SXC6 | CYP1A2 |
| SXC6 | BCHE |
| SXC6 | CA6 |
| SXC6 | CA14 |
| SXC6 | CDK5 |
| SXC6 | CDK1 |
| SXC6 | CCNB1 |
| SXC6 | CCNB2 |
| SXC7 | AKR1B1 |
| SXC7 | ADORA1 |
| SXC7 | ADORA2A |
| SXC7 | ADORA3 |
| SXC7 | OPRM1 |
| SXC7 | OPRD1 |
| SXC7 | KIT |
| SXC7 | ABCG2 |
| SXC7 | PTGS2 |
| SXC7 | ALOX5 |
| SXC7 | PTPRS |
| SXC7 | TERT |
| SXC7 | FLT3 |
| SXC7 | PIM1 |
| SXC7 | HSD17B2 |
| SXC7 | HSD17B1 |
| SXC7 | CYP1B1 |
| SXC7 | ABCC1 |
| SXC7 | NOS2 |
| SXC7 | AMY1A |
| SXC7 | GSK3B |
| SXC7 | ESR2 |
| SXC7 | PFKFB3 |
| SXC7 | BACE1 |
| SXC7 | AHR |
| SXC7 | ESRRA |
| SXC7 | SYK |
| SXC7 | NOX4 |
| SXC7 | TYR |
| SXC7 | CA2 |
| SXC7 | KDR |
| SXC7 | KDM4E |
| SXC7 | CDK6 |
| SXC7 | TTR |
| SXC7 | ABCB1 |
| SXC7 | MET |
| SXC7 | PLG |
| SXC7 | EGFR |
| SXC7 | PIK3CG |
| SXC7 | GPR35 |
| SXC7 | GLO1 |
| SXC7 | CA3 |
| SXC7 | CAMK2B |
| SXC7 | NEK6 |
| SXC7 | APEX1 |
| SXC7 | NUAK1 |
| SXC7 | AKR1C2 |
| SXC7 | AKR1C1 |
| SXC7 | AKR1C3 |
| SXC7 | AKR1C4 |
| SXC7 | AKR1A1 |
| SXC7 | MMP13 |
| SXC7 | MMP9 |
| SXC7 | ESR1 |
| SXC7 | MPG |
| SXC7 | AKR1B10 |
| SXC7 | MMP3 |
| SXC7 | MMP2 |
| SXC7 | CDK2 |
| SXC7 | CYP1A2 |
| SXC7 | MCL1 |
| SXC7 | ARG1 |
| SXC7 | CA1 |
| SXC7 | PLK1 |
| SXC7 | CBR1 |
| SXC7 | APP |
| SXC7 | BCHE |
| SXC7 | SRC |
| SXC7 | MAOA |
| SXC7 | CA12 |
| SXC7 | MYLK |
| SXC7 | MAOB |
| SXC7 | MMP12 |
| SXC7 | F2 |
| SXC7 | CCNB3 |
| SXC7 | CA7 |
| SXC7 | ALK |
| SXC7 | AR |
| SXC7 | CDK5R1 |
| SXC7 | ACHE |
| SXC7 | CDK1 |
| SXC7 | AURKB |
| SXC7 | CYP19A1 |
| SXC7 | CA9 |
| SXC7 | CA4 |
| SXC7 | XDH |
| SXC7 | PTK2 |
| SXC7 | AKT1 |
| SXC7 | ODC1 |
| SXC7 | MAPT |
| SXC7 | NAE1 |
| SXC7 | PIK3R1 |
| SXC7 | IKBKB |
| SXC7 | CA13 |
| SXC7 | ALOX15 |
| SXC7 | GRK6 |
| SXC7 | PARP1 |
| SXC7 | TAS2R31 |
| SXC7 | CA14 |
| SXC7 | CA5A |
| SXC7 | CDK1 |
| SXC7 | CDK5 |
| SXC7 | CCNB1 |
| SXC7 | CCNB2 |
| SXC8 | AKR1B1 |
| SXC8 | OPRD1 |
| SXC8 | ADORA1 |
| SXC8 | ADORA2A |
| SXC8 | ADORA3 |
| SXC8 | KIT |
| SXC8 | ABCG2 |
| SXC8 | PIM1 |
| SXC8 | PTPRS |
| SXC8 | CDK1 |
| SXC8 | ALOX5 |
| SXC8 | FLT3 |
| SXC8 | MAOA |
| SXC8 | PTGS2 |
| SXC8 | AMY1A |
| SXC8 | GRK6 |
| SXC8 | ALOX15 |
| SXC8 | OPRM1 |
| SXC8 | ABCB1 |
| SXC8 | SYK |
| SXC8 | MMP9 |
| SXC8 | MMP2 |
| SXC8 | ABCC1 |
| SXC8 | HSD17B1 |
| SXC8 | KDM4E |
| SXC8 | AKR1B10 |
| SXC8 | ACHE |
| SXC8 | ESR1 |
| SXC8 | ESR2 |
| SXC8 | APP |
| SXC8 | CYP1B1 |
| SXC8 | GSK3B |
| SXC8 | NOX4 |
| SXC8 | LCK |
| SXC8 | MMP12 |
| SXC8 | PLG |
| SXC8 | CBR1 |
| SXC8 | GLO1 |
| SXC8 | ARG1 |
| SXC8 | CCNB3 |
| SXC8 | MAOB |
| SXC8 | CA2 |
| SXC8 | CA1 |
| SXC8 | CA9 |
| SXC8 | NOS2 |
| SXC8 | XDH |
| SXC8 | CDK2 |
| SXC8 | PARP1 |
| SXC8 | CDK5R1 |
| SXC8 | CA12 |
| SXC8 | HSD17B2 |
| SXC8 | CA7 |
| SXC8 | MCL1 |
| SXC8 | TERT |
| SXC8 | AHR |
| SXC8 | ESRRA |
| SXC8 | NAE1 |
| SXC8 | MMP13 |
| SXC8 | MMP3 |
| SXC8 | AURKB |
| SXC8 | CSNK2A1 |
| SXC8 | MET |
| SXC8 | AR |
| SXC8 | TNKS |
| SXC8 | CYP19A1 |
| SXC8 | SRC |
| SXC8 | PFKFB3 |
| SXC8 | BACE1 |
| SXC8 | CA4 |
| SXC8 | KDR |
| SXC8 | TTR |
| SXC8 | GPR35 |
| SXC8 | MPO |
| SXC8 | CA3 |
| SXC8 | PKN1 |
| SXC8 | NEK6 |
| SXC8 | APEX1 |
| SXC8 | AKR1C2 |
| SXC8 | AKR1C1 |
| SXC8 | AKR1C4 |
| SXC8 | AKR1A1 |
| SXC8 | BCHE |
| SXC8 | TNKS2 |
| SXC8 | MPG |
| SXC8 | SLC22A12 |
| SXC8 | ALOX12 |
| SXC8 | CALM1 |
| SXC8 | EGFR |
| SXC8 | ST6GAL1 |
| SXC8 | CDK6 |
| SXC8 | CFTR |
| SXC8 | PIK3CG |
| SXC8 | SIGMAR1 |
| SXC8 | ODC1 |
| SXC8 | PTPN1 |
| SXC8 | IKBKB |
| SXC8 | PLK1 |
| SXC8 | CA13 |
| SXC8 | CA6 |
| SXC8 | CA14 |
| SXC8 | CDK1 |
| SXC8 | CDK5 |
| SXC8 | CCNB1 |
| SXC8 | CCNB2 |
| SXC9 | CA2 |
| SXC9 | ALOX5 |
| SXC9 | CA7 |
| SXC9 | CA1 |
| SXC9 | CA6 |
| SXC9 | MMP9 |
| SXC9 | CA12 |
| SXC9 | MMP1 |
| SXC9 | MMP2 |
| SXC9 | PTPN1 |
| SXC9 | CA14 |
| SXC9 | CA9 |
| SXC9 | CA5B |
| SXC9 | CA5A |
| SXC9 | CA3 |
| SXC9 | AKR1B1 |
| SXC9 | ESR2 |
| SXC9 | CA4 |
| SXC9 | AKR1B10 |
| SXC9 | HCAR2 |
| SXC9 | MIF |
| SXC9 | CA13 |
| SXC9 | NQO2 |
| SXC9 | TLR4 |
| SXC9 | ERBB2 |
| SXC9 | ESR1 |
| SXC9 | SLC6A2 |
| SXC9 | TTR |
| SXC9 | MAPK1 |
| SXC9 | AKR1C3 |
| SXC9 | AKR1C4 |
| SXC9 | AKR1C2 |
| SXC9 | SYK |
| SXC9 | APP |
| SXC9 | EGFR |
| SXC9 | FYN |
| SXC9 | LCK |
| SXC9 | PTGS1 |
| SXC9 | PIK3CB |
| SXC9 | CYP1A2 |
| SXC9 | CYP2C9 |
| SXC9 | CYP3A4 |
| SXC9 | CYP2C19 |
| SXC9 | PIK3CA |
| SXC9 | ELANE |
| SXC9 | F3 |
| SXC9 | HSD11B1 |
| SXC9 | NFE2L2 |
| SXC9 | STAT3 |
| SXC10 | CA2 |
| SXC10 | CA7 |
| SXC10 | CA1 |
| SXC10 | CA6 |
| SXC10 | CA12 |
| SXC10 | CA14 |
| SXC10 | CA9 |
| SXC10 | CA5A |
| SXC10 | CA5B |
| SXC10 | ALOX5 |
| SXC10 | MMP9 |
| SXC10 | MMP1 |
| SXC10 | MMP2 |
| SXC10 | PTPN1 |
| SXC10 | MAOB |
| SXC10 | CA13 |
| SXC10 | AKR1B1 |
| SXC10 | CA3 |
| SXC10 | APP |
| SXC10 | NFE2L2 |
| SXC10 | STAT3 |
| SXC10 | HSD11B1 |
| SXC10 | ESR2 |
| SXC10 | CA4 |
| SXC10 | TLR4 |
| SXC10 | PTGS1 |
| SXC10 | MET |
| SXC10 | CYP1A1 |
| SXC10 | CYP1A2 |
| SXC10 | NQO2 |
| SXC10 | CYP1B1 |
| SXC10 | EGFR |
| SXC10 | PTGS2 |
| SXC10 | TTR |
| SXC10 | TUBB1 |
| SXC10 | RELA |
| SXC10 | FYN |
| SXC10 | LCK |
| SXC10 | ADORA1 |
| SXC10 | ADORA2A |
| SXC10 | ADORA2B |
| SXC10 | TLR9 |
| SXC10 | AKR1B10 |
| SXC10 | ALOX15 |
| SXC10 | PRKCE |
| SXC10 | F3 |
| SXC10 | NOS2 |
| SXC10 | SLC16A1 |
| SXC10 | CCND1 |
| SXC10 | TUBB3 |
| SXC10 | ABCB1 |
| SXC10 | CPA1 |
| SXC10 | FBP1 |
| SXC10 | TOP2A |
| SXC10 | GLO1 |
| SXC10 | BACE1 |
| SXC10 | PARP1 |
| SXC10 | KDM4C |
| SXC10 | AHR |
| SXC10 | AMPD3 |
| SXC10 | CDK4 |
| SXC11 | SLC5A2 |
| SXC11 | ADORA1 |
| SXC11 | SLC5A1 |
| SXC11 | TYR |
| SXC11 | ADK |
| SXC11 | ADORA2A |
| SXC11 | CA14 |
| SXC11 | GBA |
| SXC11 | MANBA |
| SXC11 | SLC29A1 |
| SXC11 | SLC5A4 |
| SXC11 | ADORA3 |
| SXC11 | OGA |
| SXC11 | HK2 |
| SXC11 | HK1 |
| SXC11 | EGFR |
| SXC11 | CDK2 |
| SXC11 | HSPA8 |
| SXC11 | GAA |
| SXC11 | MMP3 |
| SXC11 | MMP9 |
| SXC11 | ADAM17 |
| SXC11 | PYGM |
| SXC11 | GAPDH |
| SXC11 | MMP1 |
| SXC11 | ADORA2B |
| SXC11 | TK1 |
| SXC11 | CDK1 |
| SXC11 | AKR1B1 |
| SXC11 | HSPA5 |
| SXC11 | SLC28A2 |
| SXC11 | ADA |
| SXC11 | CDK1 |
| SXC11 | AHCY |
| SXC11 | GSK3B |
| SXC11 | PYGL |
| SXC11 | GSTP1 |
| SXC11 | GSTM2 |
| SXC11 | AHCYL1 |
| SXC11 | FUCA1 |
| SXC11 | MMP13 |
| SXC11 | MMP7 |
| SXC11 | MMP8 |
| SXC11 | GRK1 |
| SXC11 | HRAS |
| SXC11 | MAP2K1 |
| SXC11 | CCND1 |
| SXC11 | CA1 |
| SXC11 | CA12 |
| SXC11 | CA9 |
| SXC11 | MME |
| SXC11 | F2 |
| SXC11 | PNP |
| SXC11 | GPR35 |
| SXC11 | SORT1 |
| SXC11 | TYMP |
| SXC11 | MAPK10 |
| SXC11 | UPP1 |
| SXC11 | DAO |
| SXC11 | CDC25A |
| SXC11 | MAPK1 |
| SXC11 | CCNA1 |
| SXC11 | CCNB1 |
| SXC11 | CDK4 |
| SXC11 | CCNA2 |
| SXC12 | CA2 |
| SXC12 | ESRRG |
| SXC12 | GABRA1 |
| SXC12 | PTGS1 |
| SXC12 | AR |
| SXC12 | GABRB3 |
| SXC12 | CA4 |
| SXC12 | AKR1B1 |
| SXC12 | SHBG |
| SXC12 | GABBR2 |
| SXC12 | PTGS2 |
| SXC12 | TYR |
| SXC12 | AKR1C3 |
| SXC12 | GABRB2 |
| SXC12 | GABRG2 |
| SXC12 | GABBR1 |
| SXC13 | HCAR2 |
| SXC13 | AKR1B10 |
| SXC13 | CA2 |
| SXC13 | TLR4 |
| SXC13 | CA12 |
| SXC13 | CA9 |
| SXC13 | ESR2 |
| SXC13 | CA1 |
| SXC13 | KLKB1 |
| SXC13 | F2 |
| SXC13 | PLAU |
| SXC13 | CA7 |
| SXC13 | CA14 |
| SXC13 | PDGFRA |
| SXC13 | GABRB3 |
| SXC13 | CYP11B1 |
| SXC13 | CYP11B2 |
| SXC13 | SLC16A1 |
| SXC13 | AKR1B1 |
| SXC13 | PDGFRB |
| SXC13 | GABRA3 |
| SXC13 | GABRG2 |
| SXC13 | GABRA5 |
| SXC14 | MMP9 |
| SXC14 | MMP2 |
| SXC14 | MMP12 |
| SXC14 | AKR1B1 |
| SXC14 | MMP1 |
| SXC14 | FYN |
| SXC14 | TTR |
| SXC14 | CA2 |
| SXC14 | CA12 |
| SXC14 | CA7 |
| SXC14 | CA4 |
| SXC14 | MMP13 |
| SXC14 | MMP3 |
| SXC14 | MET |
| SXC14 | AKR1B10 |
| SXC14 | ERBB2 |
| SXC14 | EGFR |
| SXC14 | CA1 |
| SXC14 | ESR1 |
| SXC14 | MAPK1 |
| SXC14 | CA6 |
| SXC14 | CA14 |
| SXC14 | CA9 |
| SXC14 | CA5B |
| SXC14 | CA5A |
| SXC14 | LCK |
| SXC14 | RAF1 |
| SXC14 | BRAF |
| SXC14 | SERPINE1 |
| SXC14 | TERT |
| SXC14 | PTGS1 |
| SXC14 | CA13 |
| SXC14 | PPARA |
| SXC14 | GCGR |
| SXC14 | APP |
| SXC14 | PGD |
| SXC14 | MMP14 |
| SXC14 | BCL2 |
| SXC14 | PPARD |
| SXC14 | EZR |
| SXC14 | MTOR |
| SXC14 | PDE10A |
| SXC14 | NOX4 |
| SXC14 | PDK1 |
| SXC14 | AURKA |
| SXC14 | SYK |
| SXC14 | KCNH2 |
| SXC14 | CHRNA7 |
| SXC14 | SAE1 |
| SXC14 | MAPK14 |
| SXC14 | MMP8 |
| SXC14 | TYR |
| SXC14 | RPS6KA3 |
| SXC14 | PTPsigma |
| SXC14 | MAP2K2 |
| SXC14 | DNM1 |
| SXC14 | PDGFRB |
| SXC14 | MCL1 |
| SXC14 | LTB4R |
| SXC14 | CHEK1 |
| SXC14 | WEE1 |
| SXC14 | NFE2L2 |
| SXC14 | ALOX15 |
| SXC14 | STAT3 |
| SXC14 | HSD11B1 |
| SXC14 | CYP1B1 |
| SXC14 | RET |
| SXC14 | GSK3A |
| SXC14 | TAS2R31 |
| SXC14 | CHEK2 |
| SXC14 | RPS6KA1 |
| SXC14 | PIK3CD |
| SXC14 | PIK3CB |
| SXC14 | PIK3CG |
| SXC14 | TGFBR1 |
| SXC14 | ACVR1 |
| SXC14 | SLC6A2 |
| SXC14 | AURKB |
| SXC14 | MAPKAPK2 |
| SXC14 | CSNK1G1 |
| SXC14 | ROCK1 |
| SXC14 | RPS6KB1 |
| SXC14 | UBA2 |
| SXC15 | ALOX5 |
| SXC15 | PTAFR |
| SXC15 | MCL1 |
| SXC15 | MAPK9 |
| SXC15 | SHBG |
| SXC15 | SLC5A2 |
| SXC15 | PIK3CG |
| SXC15 | PIK3CA |
| SXC15 | CNR2 |
| SXC16 | NOX4 |
| SXC16 | AKR1B1 |
| SXC16 | CDK5R1 |
| SXC16 | XDH |
| SXC16 | MAOA |
| SXC16 | FLT3 |
| SXC16 | CA2 |
| SXC16 | CCNB3 |
| SXC16 | ALOX5 |
| SXC16 | ADORA1 |
| SXC16 | CA7 |
| SXC16 | GLO1 |
| SXC16 | APP |
| SXC16 | SYK |
| SXC16 | GSK3B |
| SXC16 | PARP1 |
| SXC16 | TTR |
| SXC16 | MMP9 |
| SXC16 | CA12 |
| SXC16 | MMP2 |
| SXC16 | CA4 |
| SXC16 | MMP12 |
| SXC16 | CD38 |
| SXC16 | CYP1B1 |
| SXC16 | ABCG2 |
| SXC16 | AKR1B10 |
| SXC16 | TNKS2 |
| SXC16 | TNKS |
| SXC16 | TOP1 |
| SXC16 | ARG1 |
| SXC16 | PTPRS |
| SXC16 | ABCC1 |
| SXC16 | HSD17B1 |
| SXC16 | ACHE |
| SXC16 | CDK6 |
| SXC16 | ABCB1 |
| SXC16 | HSD17B2 |
| SXC16 | CYP19A1 |
| SXC16 | ESR2 |
| SXC16 | ADORA2A |
| SXC16 | CSNK2A1 |
| SXC16 | ALOX15 |
| SXC16 | ALOX12 |
| SXC16 | ESR1 |
| SXC16 | PTGS2 |
| SXC16 | CFTR |
| SXC16 | AMY1A |
| SXC16 | GRK6 |
| SXC16 | CA1 |
| SXC16 | CA9 |
| SXC16 | CDK2 |
| SXC16 | TERT |
| SXC16 | CDK1 |
| SXC16 | TYR |
| SXC16 | AHR |
| SXC16 | ESRRA |
| SXC16 | GPR35 |
| SXC16 | AVPR2 |
| SXC16 | IGF1R |
| SXC16 | EGFR |
| SXC16 | F2 |
| SXC16 | PIM1 |
| SXC16 | AURKB |
| SXC16 | DRD4 |
| SXC16 | MPO |
| SXC16 | PIK3R1 |
| SXC16 | DAPK1 |
| SXC16 | PYGL |
| SXC16 | SRC |
| SXC16 | PTK2 |
| SXC16 | KDR |
| SXC16 | MMP13 |
| SXC16 | MMP3 |
| SXC16 | CA3 |
| SXC16 | PLK1 |
| SXC16 | CA6 |
| SXC16 | PKN1 |
| SXC16 | CA14 |
| SXC16 | MET |
| SXC16 | NEK2 |
| SXC16 | CXCR1 |
| SXC16 | CAMK2B |
| SXC16 | ALK |
| SXC16 | AKT1 |
| SXC16 | NEK6 |
| SXC16 | PLA2G1B |
| SXC16 | CA5A |
| SXC16 | BACE1 |
| SXC16 | AXL |
| SXC16 | NUAK1 |
| SXC16 | AKR1C2 |
| SXC16 | AKR1C1 |
| SXC16 | AKR1C3 |
| SXC16 | AKR1C4 |
| SXC16 | CA13 |
| SXC16 | AKR1A1 |
| SXC16 | PFKFB3 |
| SXC16 | PLG |
| SXC16 | KDM4E |
| SXC16 | AR |
| SXC16 | CDK5 |
| SXC16 | CDK1 |
| SXC16 | CCNB1 |
| SXC16 | CCNB2 |
| SXC17 | XDH |
| SXC17 | EGFR |
| SXC17 | CA12 |
| SXC17 | CA9 |
| SXC17 | AKR1B1 |
| SXC17 | CA7 |
| SXC17 | CA1 |
| SXC17 | CA14 |
| SXC17 | CA2 |
| SXC17 | GPR35 |
| SXC17 | CCND1 |
| SXC17 | PDGFRB |
| SXC17 | FLT4 |
| SXC17 | INSR |
| SXC17 | CDK2 |
| SXC17 | GSK3B |
| SXC17 | PLK1 |
| SXC17 | CSNK2A1 |
| SXC17 | PLK4 |
| SXC17 | TEK |
| SXC17 | AKT1 |
| SXC17 | BACE1 |
| SXC17 | MAP3K8 |
| SXC17 | BRAF |
| SXC17 | EPHB4 |
| SXC17 | HSPA1A |
| SXC17 | NUAK1 |
| SXC17 | FGR |
| SXC17 | LYN |
| SXC17 | SQLE |
| SXC17 | ESR1 |
| SXC17 | ESR2 |
| SXC17 | IGF1R |
| SXC17 | AURKB |
| SXC17 | SRC |
| SXC17 | PTK2 |
| SXC17 | KDR |
| SXC17 | MET |
| SXC17 | ERBB2 |
| SXC17 | GSR |
| SXC17 | CA13 |
| SXC17 | CA5A |
| SXC17 | MAOA |
| SXC17 | CA4 |
| SXC17 | DAO |
| SXC17 | CA5B |
| SXC17 | CCNE1 |
| SXC17 | PTK2B |
| SXC17 | CA6 |
| SXC17 | AURKA |
| SXC17 | HSD17B3 |
| SXC17 | SRD5A1 |
| SXC17 | TNNC1 |
| SXC17 | CA3 |
| SXC17 | AKR1C3 |
| SXC17 | AKR1C1 |
| SXC17 | CBR1 |
| SXC17 | MIF |
| SXC17 | ALDH2 |
| SXC17 | SNCA |
| SXC17 | KCNA3 |
| SXC17 | CDK4 |
| SXC17 | CCNA1 |
| SXC17 | CDK2 |
| SXC17 | TNNT2 |
| SXC17 | CCNA2 |
| SXC17 | TNNI3 |
| SXC18 | AKR1B10 |
| SXC18 | PTPN1 |
| SXC18 | HSD11B1 |
| SXC18 | POLB |
| SXC18 | PTPN2 |
| SXC18 | PLA2G1B |
| SXC18 | CDC25B |
| SXC18 | RORC |
| SXC18 | PTPRF |
| SXC18 | ACP1 |
| SXC18 | PDE4D |
| SXC18 | CD81 |
| SXC18 | PTGES |
| SXC18 | CES2 |
| SXC18 | CDC25A |
| SXC18 | NOS2 |
| SXC18 | FNTA |
| SXC18 | TERT |
| SXC18 | FABP1 |
| SXC18 | AR |
| SXC18 | SCD |
| SXC18 | PTPN6 |
| SXC18 | LTB4R |
| SXC18 | SERPINA6 |
| SXC18 | SHBG |
| SXC18 | PPARA |
| SXC18 | PPARD |
| SXC18 | PREP |
| SXC18 | PPARG |
| SXC18 | PTGS2 |
| SXC18 | CYP19A1 |
| SXC18 | ESR2 |
| SXC18 | HMGCR |
| SXC18 | NR3C2 |
| SXC18 | HSD11B2 |
| SXC18 | FABP4 |
| SXC18 | FABP3 |
| SXC18 | FABP5 |
| SXC18 | PRKCH |
| SXC18 | PTPN11 |
| SXC18 | NR3C1 |
| SXC18 | G6PD |
| SXC18 | CYP51A1 |
| SXC18 | GPBAR1 |
| SXC18 | FAAH |
| SXC18 | PTGS1 |
| SXC18 | NPC1L1 |
| SXC18 | CYP17A1 |
| SXC18 | ALOX5 |
| SXC18 | PTGER2 |
| SXC18 | ADORA3 |
| SXC18 | SLC10A2 |
| SXC18 | SLC10A1 |
| SXC18 | NR1H4 |
| SXC18 | ESR1 |
| SXC18 | ALOX5AP |
| SXC18 | PTGDR |
| SXC18 | PTGDR2 |
| SXC18 | IL6 |
| SXC18 | GLUL |
| SXC18 | TOP2A |
| SXC18 | TOP1 |
| SXC18 | TLR9 |
| SXC18 | PTGER4 |
| SXC18 | NR1H3 |
| SXC18 | THRA |
| SXC18 | THRB |
| SXC18 | PLA2G4A |
| SXC18 | FNTB |
| SXC19 | CYP19A1 |
| SXC19 | AR |
| SXC19 | ESR1 |
| SXC19 | ESR2 |
| SXC19 | SHBG |
| SXC19 | SREBF2 |
| SXC19 | NPC1L1 |
| SXC19 | NR1H3 |
| SXC19 | HMGCR |
| SXC19 | CYP17A1 |
| SXC19 | CYP51A1 |
| SXC19 | RORC |
| SXC19 | PTPN1 |
| SXC19 | RORA |
| SXC19 | SERPINA6 |
| SXC19 | BCHE |
| SXC19 | CHRM2 |
| SXC19 | SLC6A2 |
| SXC19 | SLC6A4 |
| SXC19 | HSD11B1 |
| SXC19 | NR1I3 |
| SXC19 | CYP2C19 |
| SXC19 | ACHE |
| SXC19 | PPARA |
| SXC19 | PPARD |
| SXC19 | NR1H2 |
| SXC19 | NR3C1 |
| SXC19 | G6PD |
| SXC19 | SIGMAR1 |
| SXC19 | DRD2 |
| SXC19 | POLB |
| SXC19 | JAK1 |
| SXC19 | JAK2 |
| SXC19 | CES2 |
| SXC19 | PTPN2 |
| SXC19 | PLA2G1B |
| SXC19 | ACP1 |
| SXC19 | AKR1B10 |
| SXC19 | JAK3 |
| SXC19 | TYK2 |
| SXC19 | SQLE |
| SXC19 | PTGER2 |
| SXC19 | PTPN6 |
| SXC19 | BRD4 |
| SXC19 | BRD2 |
| SXC19 | PARP1 |
| SXC19 | TAAR1 |
| SXC19 | ADORA2A |
| SXC19 | PRMT3 |
| SXC19 | PRKCG |
| SXC19 | PRKCD |
| SXC19 | PRKCB |
| SXC19 | PRKCE |
| SXC19 | PRKCQ |
| SXC19 | CDC7 |
| SXC19 | RASGRP1 |
| SXC19 | UGT2B7 |
| SXC19 | CD81 |
| SXC20 | CYP19A1 |
| SXC20 | AR |
| SXC20 | ESR1 |
| SXC20 | ESR2 |
| SXC20 | SHBG |
| SXC20 | SREBF2 |
| SXC20 | NPC1L1 |
| SXC20 | NR1H3 |
| SXC20 | HMGCR |
| SXC20 | CYP17A1 |
| SXC20 | PTPN1 |
| SXC20 | CYP51A1 |
| SXC20 | RORC |
| SXC20 | SLC6A2 |
| SXC20 | SLC6A4 |
| SXC20 | CHRM2 |
| SXC20 | CYP2C19 |
| SXC20 | NR1I3 |
| SXC20 | RORA |
| SXC20 | ACHE |
| SXC20 | SERPINA6 |
| SXC20 | HSD11B1 |
| SXC20 | BCHE |
| SXC20 | POLB |
| SXC20 | PPARA |
| SXC20 | PPARD |
| SXC20 | PTPN2 |
| SXC20 | PLA2G1B |
| SXC20 | ACP1 |
| SXC20 | AKR1B10 |
| SXC20 | NR3C1 |
| SXC20 | NR1H2 |
| SXC20 | DRD2 |
| SXC20 | JAK3 |
| SXC20 | JAK1 |
| SXC20 | JAK2 |
| SXC20 | TYK2 |
| SXC20 | PTPN6 |
| SXC20 | G6PD |
| SXC20 | SIGMAR1 |
| SXC20 | UGT2B7 |
| SXC20 | CD81 |
| SXC20 | SQLE |
| SXC20 | BRD4 |
| SXC20 | BRD2 |
| SXC20 | ADORA2A |
| SXC20 | NR3C2 |
| SXC20 | CES2 |
| SXC20 | PRMT3 |
| SXC20 | TAAR1 |
| SXC20 | PDE4D |
| SXC20 | PGR |
| SXC20 | OPRM1 |
| SXC20 | OPRD1 |
| SXC20 | OPRK1 |
| SXC20 | PRKCG |
| SXC20 | PRKCD |
| SXC20 | PRKCB |
| SXC20 | PRKCE |
| SXC20 | PRKCQ |
| SXC20 | NPY5R |
| SXC20 | CDC7 |
| SXC21 | UGT2B7 |
| SXC21 | SHH |
| SXC21 | AR |
| SXC21 | CA2 |
| SXC21 | CA1 |
| SXC21 | CA4 |
| SXC21 | HSD11B1 |
| SXC21 | NR1I3 |
| SXC21 | TRPM8 |
| SXC21 | ESR1 |
| SXC21 | ESR2 |
| SXC21 | DRD2 |
| SXC21 | SIGMAR1 |
| SXC21 | SHBG |
| SXC21 | CDC25A |
| SXC21 | GPBAR1 |
| SXC21 | POLA1 |
| SXC21 | NPC1L1 |
| SXC21 | PTGS2 |
| SXC21 | NR3C1 |
| SXC21 | PGR |
| SXC21 | IDO1 |
| SXC21 | CDC25B |
| SXC21 | NR1H3 |
| SXC21 | JAK3 |
| SXC21 | JAK1 |
| SXC21 | JAK2 |
| SXC21 | TYK2 |
| SXC21 | OPRM1 |
| SXC21 | HMOX1 |
| SXC21 | NR3C2 |
| SXC21 | PARP1 |
| SXC21 | CYP11B1 |
| SXC21 | CYP11B2 |
| SXC22 | UGT2B7 |
| SXC22 | HSD11B1 |
| SXC22 | IDO1 |
| SXC22 | PTGS1 |
| SXC22 | NR1H3 |
| SXC22 | PGR |
| SXC22 | SLC6A3 |
| SXC22 | ICMT |
| SXC23 | UGT2B7 |
| SXC23 | NR1H3 |
| SXC23 | GPBAR1 |
| SXC23 | SHH |
| SXC23 | HSD11B1 |
| SXC23 | AR |
| SXC23 | NPC1L1 |
| SXC23 | NR1I3 |
| SXC23 | DRD2 |
| SXC23 | SIGMAR1 |
| SXC23 | SHBG |
| SXC23 | ESR1 |
| SXC23 | ESR2 |
| SXC23 | NR3C1 |
| SXC23 | PGR |
| SXC23 | PTGS2 |
| SXC23 | NR3C2 |
| SXC23 | NR1H4 |
| SXC23 | CDC25A |
| SXC23 | CA2 |
| SXC23 | CA1 |
| SXC23 | IDO1 |
| SXC23 | EPHX2 |
| SXC23 | HSD17B2 |
| SXC23 | POLA1 |
| SXC23 | OPRM1 |
| SXC23 | HMOX1 |
| SXC23 | CA4 |

Supplementary Table 3: Asthma-related genes obtained by searching the GeneCards, GeneMap, DisGeNet, OMIM, and DRUGBANK databases.

| Gene Symbol | Relevance score | Database |
| --- | --- | --- |
| IL13 | 60.17011642 | GeneCards |
| ADRB2 | 53.56035614 | GeneCards |
| TNF | 49.74095535 | GeneCards |
| ALOX5 | 48.90111923 | GeneCards |
| CCL11 | 48.69580078 | GeneCards |
| PLA2G7 | 39.51490021 | GeneCards |
| NPSR1 | 38.42402649 | GeneCards |
| HNMT | 37.95968628 | GeneCards |
| SCGB3A2 | 37.92459488 | GeneCards |
| TBX21 | 37.78071213 | GeneCards |
| MUC7 | 36.90569305 | GeneCards |
| HLA-G | 35.13184738 | GeneCards |
| PTGER2 | 31.72349548 | GeneCards |
| IL4R | 31.71601677 | GeneCards |
| MIR126 | 31.68259621 | GeneCards |
| BRCA2 | 31.12680054 | GeneCards |
| MIR152 | 30.83338928 | GeneCards |
| MIR148A | 30.80390167 | GeneCards |
| MIR148B | 30.39452934 | GeneCards |
| PTGDR | 29.56288528 | GeneCards |
| ASRT4 | 28.72235489 | GeneCards |
| CHI3L1 | 28.59185791 | GeneCards |
| IRAK3 | 27.70132446 | GeneCards |
| ASRT3 | 27.22413063 | GeneCards |
| ASRT6 | 27.22413063 | GeneCards |
| RNASE3 | 26.61893272 | GeneCards |
| IL4 | 26.49837303 | GeneCards |
| IL5 | 26.08127403 | GeneCards |
| MS4A2 | 25.93591499 | GeneCards |
| IGHE | 24.68911552 | GeneCards |
| FLG | 24.65475273 | GeneCards |
| ASRT8 | 23.27416992 | GeneCards |
| ADAM33 | 20.1482029 | GeneCards |
| CYSLTR1 | 20.03795624 | GeneCards |
| IL10 | 19.20279312 | GeneCards |
| TGFB1 | 18.62188148 | GeneCards |
| IFNG | 18.23958588 | GeneCards |
| SPINK5 | 18.09095192 | GeneCards |
| CXCL8 | 17.93487167 | GeneCards |
| CCL5 | 17.90786362 | GeneCards |
| SCGB1A1 | 17.5534687 | GeneCards |
| CD14 | 16.98764229 | GeneCards |
| LTC4S | 16.77782631 | GeneCards |
| TSLP | 16.71398163 | GeneCards |
| IL9 | 16.58073044 | GeneCards |
| ORMDL3 | 16.39738846 | GeneCards |
| IL3 | 16.32358551 | GeneCards |
| IL6 | 15.90454102 | GeneCards |
| GSTP1 | 15.90398788 | GeneCards |
| TLR4 | 15.79980564 | GeneCards |
| HAVCR1 | 15.68398571 | GeneCards |
| IGES | 15.66012383 | GeneCards |
| IL17A | 15.37931442 | GeneCards |
| CFTR | 15.30645752 | GeneCards |
| STAT6 | 15.14654064 | GeneCards |
| GSTM1 | 14.92310238 | GeneCards |
| HLA-DRB1 | 14.86612511 | GeneCards |
| IL18 | 14.74894142 | GeneCards |
| CYSLTR2 | 14.70613098 | GeneCards |
| TLR2 | 14.68383217 | GeneCards |
| EPX | 14.6246376 | GeneCards |
| MIR155 | 14.3880043 | GeneCards |
| IL12B | 14.36049843 | GeneCards |
| IL33 | 14.22581577 | GeneCards |
| HLA-DQB1 | 14.19873428 | GeneCards |
| IL2 | 14.10492992 | GeneCards |
| CTLA4 | 13.97812557 | GeneCards |
| IL1B | 13.94536877 | GeneCards |
| PHF11 | 13.9123745 | GeneCards |
| CHIA | 13.91057968 | GeneCards |
| NOD2 | 13.77928925 | GeneCards |
| DPP10 | 13.72528458 | GeneCards |
| ICAM1 | 13.64395714 | GeneCards |
| NR3C1 | 13.59873581 | GeneCards |
| NPSR1-AS1 | 13.40730953 | GeneCards |
| CSF2 | 13.3888979 | GeneCards |
| ADA | 13.28682995 | GeneCards |
| ALOX5AP | 13.23269844 | GeneCards |
| GATA3 | 13.22352409 | GeneCards |
| IL1RL1 | 13.1570034 | GeneCards |
| MMP9 | 13.14248943 | GeneCards |
| MYLK | 12.69578743 | GeneCards |
| HRH1 | 12.66615105 | GeneCards |
| MPO | 12.61865044 | GeneCards |
| CCL24 | 12.55816555 | GeneCards |
| IL6R | 12.53868389 | GeneCards |
| VCAM1 | 12.51297474 | GeneCards |
| CCL2 | 12.510355 | GeneCards |
| CCR3 | 12.38983345 | GeneCards |
| CCL26 | 12.2891531 | GeneCards |
| IL16 | 12.19419384 | GeneCards |
| IL2RA | 12.18495655 | GeneCards |
| FCGR2A | 12.14811611 | GeneCards |
| CCL17 | 12.12125683 | GeneCards |
| HLA-DPB1 | 11.91799545 | GeneCards |
| CCL3 | 11.90241051 | GeneCards |
| IL1A | 11.84346008 | GeneCards |
| TLR6 | 11.83820724 | GeneCards |
| IL1R1 | 11.707201 | GeneCards |
| TLR9 | 11.7013607 | GeneCards |
| IL1RN | 11.61829948 | GeneCards |
| LEP | 11.51038074 | GeneCards |
| CASP8 | 11.50342846 | GeneCards |
| HLA-DQA1 | 11.48640728 | GeneCards |
| TAC1 | 11.4823122 | GeneCards |
| TBXA2R | 11.46608543 | GeneCards |
| VDR | 11.43274879 | GeneCards |
| COMT | 11.42254925 | GeneCards |
| ALOX15 | 11.4096117 | GeneCards |
| PDE4A | 11.36224651 | GeneCards |
| ITGAM | 11.34609127 | GeneCards |
| CAT | 11.2925539 | GeneCards |
| IL18R1 | 11.20030785 | GeneCards |
| BDNF | 11.1716423 | GeneCards |
| EDN1 | 11.16042042 | GeneCards |
| CD69 | 11.10199642 | GeneCards |
| FCER2 | 11.10007381 | GeneCards |
| NOS2 | 11.02650166 | GeneCards |
| IRF1 | 11.01719952 | GeneCards |
| NOS1 | 11.00943756 | GeneCards |
| CCL22 | 11.00690746 | GeneCards |
| PTGDR2 | 11.00621891 | GeneCards |
| KITLG | 10.95384026 | GeneCards |
| STAT1 | 10.93946552 | GeneCards |
| MBL2 | 10.92455006 | GeneCards |
| FOXP3 | 10.63730717 | GeneCards |
| SELP | 10.62982845 | GeneCards |
| IL17F | 10.62454987 | GeneCards |
| LTA4H | 10.60939217 | GeneCards |
| IL5RA | 10.6090498 | GeneCards |
| FCER1A | 10.57509232 | GeneCards |
| ITK | 10.57308006 | GeneCards |
| DENND1B | 10.52772427 | GeneCards |
| CXCR3 | 10.51486492 | GeneCards |
| MUC5AC | 10.50243568 | GeneCards |
| NOD1 | 10.49151802 | GeneCards |
| TLR10 | 10.48087978 | GeneCards |
| CCR4 | 10.41431427 | GeneCards |
| KNG1 | 10.39070129 | GeneCards |
| CXCL10 | 10.38491058 | GeneCards |
| VIP | 10.36119843 | GeneCards |
| ARG1 | 10.30952072 | GeneCards |
| CDHR3 | 10.30226231 | GeneCards |
| SELE | 10.29613876 | GeneCards |
| POSTN | 10.2253828 | GeneCards |
| TPT1 | 10.21807766 | GeneCards |
| ASOBS | 10.21300888 | GeneCards |
| ELANE | 10.17427158 | GeneCards |
| SOD2 | 10.1710043 | GeneCards |
| SELL | 10.14168739 | GeneCards |
| IL9R | 10.03457642 | GeneCards |
| ADAM8 | 10.00677967 | GeneCards |
| HLA-DRA | 9.997135162 | GeneCards |
| NLRP3 | 9.960375786 | GeneCards |
| TLR1 | 9.944952965 | GeneCards |
| TIMP1 | 9.925845146 | GeneCards |
| CD40LG | 9.923100471 | GeneCards |
| COL26A1 | 9.918763161 | GeneCards |
| TRPV1 | 9.889282227 | GeneCards |
| CMA1 | 9.870639801 | GeneCards |
| CSF3 | 9.869169235 | GeneCards |
| CCL7 | 9.855430603 | GeneCards |
| CSF1 | 9.842237473 | GeneCards |
| HAVCR2 | 9.837450981 | GeneCards |
| CD40 | 9.832602501 | GeneCards |
| NGF | 9.829722404 | GeneCards |
| ADIPOQ | 9.81540966 | GeneCards |
| GLCCI1 | 9.804593086 | GeneCards |
| BGLAP | 9.799670219 | GeneCards |
| SFTPD | 9.795338631 | GeneCards |
| CHIT1 | 9.749047279 | GeneCards |
| BMP6 | 9.745193481 | GeneCards |
| PRG2 | 9.716785431 | GeneCards |
| JUN | 9.705171585 | GeneCards |
| NPPA | 9.699124336 | GeneCards |
| IL13RA1 | 9.670181274 | GeneCards |
| ARG2 | 9.650295258 | GeneCards |
| NPY | 9.643086433 | GeneCards |
| F2RL1 | 9.626386642 | GeneCards |
| CD28 | 9.583223343 | GeneCards |
| HLA-DQA2 | 9.569906235 | GeneCards |
| MMP2 | 9.528141975 | GeneCards |
| RASGRP4 | 9.509477615 | GeneCards |
| MMP1 | 9.455155373 | GeneCards |
| CD4 | 9.420642853 | GeneCards |
| PTGS1 | 9.418784142 | GeneCards |
| FAS | 9.393996239 | GeneCards |
| HLA-DPA1 | 9.365171432 | GeneCards |
| CD86 | 9.359546661 | GeneCards |
| PPBP | 9.350975037 | GeneCards |
| TPSAB1 | 9.34701252 | GeneCards |
| ADCY9 | 9.339775085 | GeneCards |
| CALCA | 9.317861557 | GeneCards |
| PF4 | 9.303313255 | GeneCards |
| CXCL1 | 9.261574745 | GeneCards |
| MMP12 | 9.230830193 | GeneCards |
| CCL13 | 9.222177505 | GeneCards |
| CREB1 | 9.188137054 | GeneCards |
| CLCA1 | 9.173843384 | GeneCards |
| HDAC2 | 9.138772011 | GeneCards |
| TLR3 | 9.127590179 | GeneCards |
| FKBP5 | 9.115498543 | GeneCards |
| JAK2 | 9.08556366 | GeneCards |
| PLAU | 9.081281662 | GeneCards |
| IL12RB2 | 9.074848175 | GeneCards |
| PPARG | 9.062883377 | GeneCards |
| IL31 | 9.045798302 | GeneCards |
| CD79A | 9.03143692 | GeneCards |
| TACR1 | 9.015795708 | GeneCards |
| CXCL12 | 8.992556572 | GeneCards |
| CCR6 | 8.982315063 | GeneCards |
| SLC6A4 | 8.947847366 | GeneCards |
| ADORA2A | 8.897247314 | GeneCards |
| IL13RA2 | 8.892249107 | GeneCards |
| POMC | 8.882170677 | GeneCards |
| SYK | 8.867908478 | GeneCards |
| AREG | 8.829868317 | GeneCards |
| ADRA1B | 8.829401016 | GeneCards |
| C5AR1 | 8.828278542 | GeneCards |
| ABCC1 | 8.826049805 | GeneCards |
| TACR2 | 8.824731827 | GeneCards |
| NFKBIA | 8.798202515 | GeneCards |
| ADORA2B | 8.791568756 | GeneCards |
| FASLG | 8.787089348 | GeneCards |
| CXCR1 | 8.77859211 | GeneCards |
| CPA3 | 8.755352974 | GeneCards |
| FCER1G | 8.75176239 | GeneCards |
| CYP3A4 | 8.724244118 | GeneCards |
| IL11 | 8.690877914 | GeneCards |
| SERPINA1 | 8.686271667 | GeneCards |
| CP | 8.682246208 | GeneCards |
| ITGA4 | 8.675207138 | GeneCards |
| GSR | 8.639348984 | GeneCards |
| FGFBP2 | 8.630850792 | GeneCards |
| F2R | 8.624911308 | GeneCards |
| PLA2G2A | 8.622565269 | GeneCards |
| CD80 | 8.619934082 | GeneCards |
| ABCB1 | 8.600937843 | GeneCards |
| CCL4 | 8.592821121 | GeneCards |
| ADORA1 | 8.532004356 | GeneCards |
| CXCL5 | 8.526722908 | GeneCards |
| SERPINA3 | 8.51396656 | GeneCards |
| HLA-DOA | 8.511415482 | GeneCards |
| ADCY10 | 8.487546921 | GeneCards |
| CRH | 8.485701561 | GeneCards |
| ACP1 | 8.468743324 | GeneCards |
| G6PD | 8.468504906 | GeneCards |
| MAPK3 | 8.435940742 | GeneCards |
| IL7 | 8.429148674 | GeneCards |
| RAPGEF3 | 8.412953377 | GeneCards |
| CD44 | 8.408207893 | GeneCards |
| PIK3CG | 8.399003029 | GeneCards |
| CXCR4 | 8.385243416 | GeneCards |
| HP | 8.379307747 | GeneCards |
| GHRL | 8.376504898 | GeneCards |
| CXCR2 | 8.369828224 | GeneCards |
| CCR8 | 8.368268013 | GeneCards |
| ADAMTSL1 | 8.33172226 | GeneCards |
| ADM | 8.320796013 | GeneCards |
| RNASE2 | 8.30277729 | GeneCards |
| TF | 8.299291611 | GeneCards |
| MME | 8.290068626 | GeneCards |
| LTB4R | 8.244748116 | GeneCards |
| MAPK14 | 8.236738205 | GeneCards |
| ADRB1 | 8.231435776 | GeneCards |
| VTN | 8.203193665 | GeneCards |
| JAK1 | 8.19987011 | GeneCards |
| CYP2C9 | 8.167334557 | GeneCards |
| AKR1B1 | 8.11380291 | GeneCards |
| TNFRSF10A | 8.113045692 | GeneCards |
| TNFSF10 | 8.112318993 | GeneCards |
| P2RY12 | 8.077951431 | GeneCards |
| THPO | 8.03023243 | GeneCards |
| GAL | 8.029896736 | GeneCards |
| ICOSLG | 8.008196831 | GeneCards |
| F3 | 8.006797791 | GeneCards |
| LPO | 8.005133629 | GeneCards |
| OPRM1 | 8.001186371 | GeneCards |
| IGFBP3 | 7.983510017 | GeneCards |
| ITGA2 | 7.972917557 | GeneCards |
| AGTR1 | 7.963107586 | GeneCards |
| SP1 | 7.948368549 | GeneCards |
| ACE | 7.946042061 | GeneCards |
| CLC | 7.936238289 | GeneCards |
| HRH2 | 7.924213409 | GeneCards |
| F2 | 7.918446064 | GeneCards |
| INS | 7.870684624 | GeneCards |
| F2RL3 | 7.869746208 | GeneCards |
| SST | 7.864010811 | GeneCards |
| LTA | 7.859342575 | GeneCards |
| LAT | 7.84767437 | GeneCards |
| SLC22A2 | 7.812167645 | GeneCards |
| RYR2 | 7.774240494 | GeneCards |
| FGA | 7.760986328 | GeneCards |
| GNRH1 | 7.748669147 | GeneCards |
| GRK5 | 7.732705116 | GeneCards |
| ADCYAP1 | 7.732705116 | GeneCards |
| EIF4E | 7.623798847 | GeneCards |
| PITX2 | 7.598691463 | GeneCards |
| KLK3 | 7.598691463 | GeneCards |
| SLC22A3 | 7.568910122 | GeneCards |
| ADORA3 | 7.549983978 | GeneCards |
| IL21R | 7.547827721 | GeneCards |
| MB | 7.530097961 | GeneCards |
| HBG2 | 7.530097961 | GeneCards |
| SERPINA6 | 7.513515472 | GeneCards |
| CYP3A5 | 7.478008747 | GeneCards |
| ADRB3 | 7.436397076 | GeneCards |
| PVT1 | 7.361498356 | GeneCards |
| GAPDH | 7.279420376 | GeneCards |
| SDHB | 7.236614227 | GeneCards |
| BCL11B | 7.128394604 | GeneCards |
| STAT5B | 7.089401245 | GeneCards |
| GSTT1 | 7.06162262 | GeneCards |
| ATOD1 | 7.014172554 | GeneCards |
| DDX41 | 6.848649502 | GeneCards |
| ITGB2 | 6.540567398 | GeneCards |
| CREBBP | 6.300204277 | GeneCards |
| KIT | 6.153619766 | GeneCards |
| CLEC7A | 6.093255997 | GeneCards |
| VEGFA | 6.009780884 | GeneCards |
| PTGS2 | 6.004395962 | GeneCards |
| GSDMB | 5.863120079 | GeneCards |
| CDSN | 5.770103931 | GeneCards |
| CLEC1A | 5.760959625 | GeneCards |
| TGFBR1 | 5.700873852 | GeneCards |
| CCR5 | 5.478936672 | GeneCards |
| RUNX1 | 5.279840469 | GeneCards |
| HLA-B | 5.261995316 | GeneCards |
| FLG-AS1 | 5.198022842 | GeneCards |
| EGF | 5.133525848 | GeneCards |
| NKX2-1 | 5.0782547 | GeneCards |
| CARD11 | 5.072813511 | GeneCards |
| WDR46 | 5.03286171 | GeneCards |
| ATOD3 | 4.973373413 | GeneCards |
| PLCG2 | 4.962068081 | GeneCards |
| EP300 | 4.951002598 | GeneCards |
| POLR1H | 4.899185658 | GeneCards |
| RIC1 | 4.885139942 | GeneCards |
| IL2RB | 4.840040207 | GeneCards |
| SERPINE1 | 4.826075554 | GeneCards |
| CPN1 | 4.80962944 | GeneCards |
| TGFBR2 | 4.775547028 | GeneCards |
| FGFR1 | 4.743658543 | GeneCards |
| NOS3 | 4.708145142 | GeneCards |
| STAT3 | 4.698065281 | GeneCards |
| NAT2 | 4.663385868 | GeneCards |
| SHH | 4.634970188 | GeneCards |
| NEK9 | 4.568138599 | GeneCards |
| ALB | 4.55604744 | GeneCards |
| IL15 | 4.522335529 | GeneCards |
| CX3CR1 | 4.512227058 | GeneCards |
| PDGFRA | 4.493862152 | GeneCards |
| ODC1 | 4.452606201 | GeneCards |
| CRP | 4.438743591 | GeneCards |
| LIFR | 4.421324253 | GeneCards |
| GRHL2 | 4.350292683 | GeneCards |
| CBL | 4.321454525 | GeneCards |
| BBS1 | 4.314194202 | GeneCards |
| AFF4 | 4.242847443 | GeneCards |
| ERCC2 | 4.187997818 | GeneCards |
| ELOVL4 | 4.187997818 | GeneCards |
| GP1BB | 4.187997818 | GeneCards |
| PEX5 | 4.187997818 | GeneCards |
| TRAIP | 4.187997818 | GeneCards |
| VPS51 | 4.187997818 | GeneCards |
| SOD1 | 4.140937328 | GeneCards |
| DOCK8 | 4.137752533 | GeneCards |
| TET2 | 4.096280575 | GeneCards |
| GP1BA | 4.079414845 | GeneCards |
| TGIF1 | 4.076747894 | GeneCards |
| MIR22 | 4.06956625 | GeneCards |
| LIG4 | 4.013411045 | GeneCards |
| PGM3 | 3.975457668 | GeneCards |
| ASXL1 | 3.975457668 | GeneCards |
| SRSF2 | 3.975457668 | GeneCards |
| IL12RB1 | 3.935165405 | GeneCards |
| TBCK | 3.934053421 | GeneCards |
| ALMS1 | 3.911699057 | GeneCards |
| MTOR | 3.907794952 | GeneCards |
| NFKB2 | 3.907794952 | GeneCards |
| SOCS3 | 3.860986471 | GeneCards |
| CARMIL2 | 3.847228765 | GeneCards |
| GLI2 | 3.840352535 | GeneCards |
| GAS1 | 3.840352535 | GeneCards |
| JMJD1C | 3.840352535 | GeneCards |
| CRHR1 | 3.831298828 | GeneCards |
| SMAD3 | 3.823086262 | GeneCards |
| FGF8 | 3.817998171 | GeneCards |
| GTF2H5 | 3.817998171 | GeneCards |
| TAP1 | 3.689926147 | GeneCards |
| CRLF2 | 3.655834913 | GeneCards |
| FBN1 | 3.63450098 | GeneCards |
| HMOX1 | 3.628185749 | GeneCards |
| AICDA | 3.601909161 | GeneCards |
| LRP1 | 3.58233881 | GeneCards |
| TLR7 | 3.580549717 | GeneCards |
| PSORS1C1 | 3.576266289 | GeneCards |
| CAMP | 3.533457756 | GeneCards |
| PEPD | 3.531558275 | GeneCards |
| IFNA1 | 3.52148056 | GeneCards |
| THBD | 3.493042231 | GeneCards |
| IL12A | 3.448987484 | GeneCards |
| TNFRSF8 | 3.439386368 | GeneCards |
| PTCH1 | 3.437857389 | GeneCards |
| IDS | 3.437857389 | GeneCards |
| USP7 | 3.437857389 | GeneCards |
| STXBP1 | 3.437857389 | GeneCards |
| IGF2 | 3.437857389 | GeneCards |
| GP9 | 3.437857389 | GeneCards |
| CDKN1C | 3.437857389 | GeneCards |
| TALDO1 | 3.437857389 | GeneCards |
| DLL1 | 3.437857389 | GeneCards |
| LMX1B | 3.437857389 | GeneCards |
| HMGA2 | 3.437857389 | GeneCards |
| CDON | 3.437857389 | GeneCards |
| SDHD | 3.437857389 | GeneCards |
| SEC24C | 3.437857389 | GeneCards |
| SCN4A | 3.437857389 | GeneCards |
| TBX1 | 3.437857389 | GeneCards |
| ZIC2 | 3.437857389 | GeneCards |
| SUFU | 3.437857389 | GeneCards |
| RREB1 | 3.437857389 | GeneCards |
| FOXH1 | 3.437857389 | GeneCards |
| COX4I2 | 3.437857389 | GeneCards |
| HIRA | 3.437857389 | GeneCards |
| ARL6 | 3.437857389 | GeneCards |
| NSUN2 | 3.437857389 | GeneCards |
| NODAL | 3.437857389 | GeneCards |
| SIX3 | 3.437857389 | GeneCards |
| TDGF1 | 3.437857389 | GeneCards |
| SIK3 | 3.437857389 | GeneCards |
| LRBA | 3.437857389 | GeneCards |
| WAC | 3.437857389 | GeneCards |
| PLAG1 | 3.437857389 | GeneCards |
| DISP1 | 3.437857389 | GeneCards |
| ARVCF | 3.437857389 | GeneCards |
| CCDC28B | 3.437857389 | GeneCards |
| UFD1 | 3.437857389 | GeneCards |
| ODAD3 | 3.437857389 | GeneCards |
| NQO1 | 3.42685461 | GeneCards |
| RORA | 3.414460182 | GeneCards |
| CYFIP2 | 3.395271301 | GeneCards |
| RAD50 | 3.385202408 | GeneCards |
| CCL18 | 3.379778147 | GeneCards |
| ERMP1 | 3.366129398 | GeneCards |
| PDCD1 | 3.3633461 | GeneCards |
| GSDMA | 3.356122255 | GeneCards |
| SFTPA1 | 3.35381031 | GeneCards |
| C3 | 3.334320545 | GeneCards |
| ITGB3 | 3.330754519 | GeneCards |
| NSD1 | 3.312022209 | GeneCards |
| EPHX1 | 3.306561947 | GeneCards |
| CHRM3 | 3.298671246 | GeneCards |
| GC | 3.29444313 | GeneCards |
| CXCL9 | 3.292864084 | GeneCards |
| ENPP3 | 3.277534246 | GeneCards |
| CX3CL1 | 3.263161898 | GeneCards |
| MTHFR | 3.243919373 | GeneCards |
| CHAT | 3.225129604 | GeneCards |
| GMPPA | 3.225129604 | GeneCards |
| ASIC4-AS1 | 3.225129604 | GeneCards |
| GER | 3.225129604 | GeneCards |
| GPT | 3.181508303 | GeneCards |
| IL6ST | 3.177623987 | GeneCards |
| S100A8 | 3.173127651 | GeneCards |
| HMGB1 | 3.163199425 | GeneCards |
| CTNNA3 | 3.151883364 | GeneCards |
| MIR146A | 3.130794287 | GeneCards |
| HLA-A | 3.127897024 | GeneCards |
| HLA-C | 3.114464283 | GeneCards |
| PLOD1 | 3.07002306 | GeneCards |
| FCGR3A | 3.036389828 | GeneCards |
| MUC5B | 3.032492876 | GeneCards |
| MIF | 2.995622396 | GeneCards |
| NEU1 | 2.984707117 | GeneCards |
| SOCS1 | 2.977764845 | GeneCards |
| MMP3 | 2.974119663 | GeneCards |
| PTGER4 | 2.942875862 | GeneCards |
| TGFB2 | 2.927630901 | GeneCards |
| TNFSF4 | 2.906713963 | GeneCards |
| WDR36 | 2.905128479 | GeneCards |
| PLAUR | 2.895936251 | GeneCards |
| ATP12A | 2.884880304 | GeneCards |
| IL25 | 2.884331226 | GeneCards |
| ICOS | 2.867897749 | GeneCards |
| PDE4D | 2.864671946 | GeneCards |
| CCL20 | 2.857662678 | GeneCards |
| PAFAH1B1 | 2.839166403 | GeneCards |
| CCL1 | 2.818920612 | GeneCards |
| CCR2 | 2.813297987 | GeneCards |
| KIF3A | 2.812600374 | GeneCards |
| DEFB1 | 2.812113047 | GeneCards |
| EGFR | 2.797023535 | GeneCards |
| IFNB1 | 2.78933382 | GeneCards |
| LGALS3 | 2.782435417 | GeneCards |
| NFKB1 | 2.76555872 | GeneCards |
| BBS9 | 2.760353565 | GeneCards |
| AGER | 2.746382952 | GeneCards |
| S100A9 | 2.731588125 | GeneCards |
| MAPK1 | 2.730783939 | GeneCards |
| ATP4A | 2.727420807 | GeneCards |
| SFTA3 | 2.723433971 | GeneCards |
| PRKCA | 2.720382929 | GeneCards |
| STAT5A | 2.708269358 | GeneCards |
| PLA2G4A | 2.704412699 | GeneCards |
| CYBA | 2.671512842 | GeneCards |
| USB1 | 2.660651684 | GeneCards |
| NFE2L2 | 2.65667057 | GeneCards |
| CFTR-AS1 | 2.656208277 | GeneCards |
| ELN | 2.652828455 | GeneCards |
| C3AR1 | 2.639707327 | GeneCards |
| IFNGR2 | 2.63947773 | GeneCards |
| IFNGR1 | 2.63540554 | GeneCards |
| RANBP6 | 2.629818201 | GeneCards |
| NTF3 | 2.620613575 | GeneCards |
| GZMB | 2.620187283 | GeneCards |
| ALDH2 | 2.59809804 | GeneCards |
| BBS7 | 2.593634844 | GeneCards |
| OXA1L | 2.588762999 | GeneCards |
| LEPQTL1 | 2.5803442 | GeneCards |
| PRKG1 | 2.566848993 | GeneCards |
| GSTA1 | 2.562969685 | GeneCards |
| MPLKIP | 2.561139345 | GeneCards |
| DEL18Q | 2.561139345 | GeneCards |
| CHRM2 | 2.559151173 | GeneCards |
| SLC25A46 | 2.557487488 | GeneCards |
| STAT4 | 2.553558111 | GeneCards |
| HSP90AA1 | 2.545001745 | GeneCards |
| SERPINB4 | 2.534207344 | GeneCards |
| BBS12 | 2.508140802 | GeneCards |
| ITGAL | 2.497012138 | GeneCards |
| TLE4 | 2.473114967 | GeneCards |
| IL17D | 2.470239878 | GeneCards |
| CHUK | 2.463154078 | GeneCards |
| SLC22A5 | 2.460375547 | GeneCards |
| SLPI | 2.457124949 | GeneCards |
| HRH4 | 2.451976776 | GeneCards |
| ATG5 | 2.446300268 | GeneCards |
| PTPRD | 2.409098148 | GeneCards |
| SFTPB | 2.408194304 | GeneCards |
| ANXA5 | 2.40680933 | GeneCards |
| CTSG | 2.400264263 | GeneCards |
| IKZF3 | 2.399683952 | GeneCards |
| ESR1 | 2.397011518 | GeneCards |
| LEPR | 2.39547658 | GeneCards |
| CD8A | 2.38889575 | GeneCards |
| CEP19 | 2.380212784 | GeneCards |
| MIR142 | 2.37787962 | GeneCards |
| FURIN | 2.375284672 | GeneCards |
| CCL27 | 2.367801189 | GeneCards |
| IL21 | 2.367383957 | GeneCards |
| LTF | 2.364520311 | GeneCards |
| C5 | 2.362326145 | GeneCards |
| SPP1 | 2.358914375 | GeneCards |
| MYB | 2.353810549 | GeneCards |
| PTX3 | 2.353180647 | GeneCards |
| ZPBP2 | 2.346790552 | GeneCards |
| IRF4 | 2.345210075 | GeneCards |
| CRB1 | 2.336436033 | GeneCards |
| C4A | 2.335425138 | GeneCards |
| NFKBIL1 | 2.335425138 | GeneCards |
| MIR221 | 2.335407257 | GeneCards |
| CTCF | 2.331115723 | GeneCards |
| MIR21 | 2.32384634 | GeneCards |
| PTGER3 | 2.318345785 | GeneCards |
| DPP4 | 2.317158937 | GeneCards |
| IVL | 2.305313826 | GeneCards |
| PRTN3 | 2.303936005 | GeneCards |
| SIGLEC8 | 2.299316883 | GeneCards |
| NOTCH4 | 2.292372227 | GeneCards |
| BBS2 | 2.284840584 | GeneCards |
| BBS4 | 2.284840584 | GeneCards |
| HARS1 | 2.280510902 | GeneCards |
| C19orf12 | 2.280510902 | GeneCards |
| EOE1 | 2.280510902 | GeneCards |
| SERPINB1 | 2.276894331 | GeneCards |
| SFTPA2 | 2.276071787 | GeneCards |
| CDH1 | 2.272651672 | GeneCards |
| PBX2 | 2.26893568 | GeneCards |
| GSTO2 | 2.267033815 | GeneCards |
| CASP1 | 2.262506723 | GeneCards |
| ANGPT1 | 2.262388229 | GeneCards |
| TRPA1 | 2.25930357 | GeneCards |
| FMR1 | 2.257056713 | GeneCards |
| MICB | 2.243876934 | GeneCards |
| PRKCZ | 2.231833458 | GeneCards |
| HTR2A | 2.231329203 | GeneCards |
| CSN1S1 | 2.230118752 | GeneCards |
| LTB | 2.227576733 | GeneCards |
| IRF7 | 2.221728325 | GeneCards |
| MLX | 2.213494062 | GeneCards |
| WDR19 | 2.213494062 | GeneCards |
| WDPCP | 2.213494062 | GeneCards |
| CD209 | 2.210341692 | GeneCards |
| ADH5 | 2.208942652 | GeneCards |
| PI3 | 2.206929445 | GeneCards |
| SFTPC | 2.201667309 | GeneCards |
| DUSP1 | 2.192136765 | GeneCards |
| BBS10 | 2.191139698 | GeneCards |
| BBS5 | 2.191139698 | GeneCards |
| LRRC32 | 2.189529657 | GeneCards |
| TNXB | 2.187136412 | GeneCards |
| LCN2 | 2.180071831 | GeneCards |
| PIK3C2A | 2.176949024 | GeneCards |
| CYP1A1 | 2.174040079 | GeneCards |
| MMP8 | 2.173350573 | GeneCards |
| NDFIP1 | 2.172973633 | GeneCards |
| IL17RB | 2.17168808 | GeneCards |
| TLR5 | 2.170708179 | GeneCards |
| TXN | 2.16696763 | GeneCards |
| PRL | 2.165860176 | GeneCards |
| CHRM1 | 2.161325693 | GeneCards |
| CRHR2 | 2.15852952 | GeneCards |
| MUC2 | 2.15410924 | GeneCards |
| GHITM | 2.147810459 | GeneCards |
| MAVS | 2.140672922 | GeneCards |
| CD36 | 2.137884617 | GeneCards |
| TBXT | 2.129465818 | GeneCards |
| CYP2R1 | 2.127071142 | GeneCards |
| DDX39B | 2.12603116 | GeneCards |
| FLT1 | 2.120020866 | GeneCards |
| PTGER1 | 2.119316101 | GeneCards |
| INPP4A | 2.092643976 | GeneCards |
| SOCS2 | 2.091326475 | GeneCards |
| IRF3 | 2.085631132 | GeneCards |
| RETN | 2.085620642 | GeneCards |
| IL7R | 2.084369659 | GeneCards |
| SUOX | 2.079951525 | GeneCards |
| CSTA | 2.069741726 | GeneCards |
| TIMD4 | 2.061705589 | GeneCards |
| MUC1 | 2.058335304 | GeneCards |
| SERPINB2 | 2.057177782 | GeneCards |
| IL27 | 2.056320429 | GeneCards |
| S100A12 | 2.055903196 | GeneCards |
| TNFSF13B | 2.054041386 | GeneCards |
| TNFAIP3 | 2.051447868 | GeneCards |
| PSMD3 | 2.050813913 | GeneCards |
| IL22 | 2.049944162 | GeneCards |
| HDC | 2.04554081 | GeneCards |
| PARP1 | 2.044525146 | GeneCards |
| CCR7 | 2.041435242 | GeneCards |
| HDAC1 | 2.040311575 | GeneCards |
| IDO1 | 2.033631086 | GeneCards |
| EDNRA | 2.033341169 | GeneCards |
| BCL2 | 2.02591157 | GeneCards |
| TIMELESS | 2.024738312 | GeneCards |
| BTNL2 | 2.024358511 | GeneCards |
| IFRD1 | 2.01309967 | GeneCards |
| NTS | 2.009940863 | GeneCards |
| KLK7 | 2.005671024 | GeneCards |
| IFNA2 | 2.003633022 | GeneCards |
| SLC26A4 | 2.002349854 | GeneCards |
| IL18RAP | 1.999734521 | GeneCards |
| IFIH1 | 1.996865869 | GeneCards |
| ANXA2 | 1.992384672 | GeneCards |
| HLX | 1.991401434 | GeneCards |
| AOC1 | 1.991223097 | GeneCards |
| CYP2C19 | 1.990808845 | GeneCards |
| PON1 | 1.990172982 | GeneCards |
| IL10RA | 1.989688396 | GeneCards |
| FN1 | 1.98788476 | GeneCards |
| MICA | 1.98509109 | GeneCards |
| ITLN1 | 1.982659221 | GeneCards |
| PTGES | 1.98097682 | GeneCards |
| CAV1 | 1.979417562 | GeneCards |
| LORICRIN | 1.97630024 | GeneCards |
| PCNA | 1.972014427 | GeneCards |
| MIR31 | 1.95635426 | GeneCards |
| DDX58 | 1.954399824 | GeneCards |
| FCGR1A | 1.95028615 | GeneCards |
| SLC7A2 | 1.944928408 | GeneCards |
| SDCCAG8 | 1.943511963 | GeneCards |
| CLIC2 | 1.943511963 | GeneCards |
| RAB39B | 1.943511963 | GeneCards |
| CCN2 | 1.938620567 | GeneCards |
| TTLL1 | 1.937908888 | GeneCards |
| CCND1 | 1.932810187 | GeneCards |
| COX5A | 1.93258357 | GeneCards |
| SPATS2L | 1.931109667 | GeneCards |
| DNAH8 | 1.927540421 | GeneCards |
| LYN | 1.921054721 | GeneCards |
| PYHIN1 | 1.919416785 | GeneCards |
| CRCT1 | 1.919416785 | GeneCards |
| ERBB4 | 1.911971331 | GeneCards |
| MIR145 | 1.907953978 | GeneCards |
| RORC | 1.906910896 | GeneCards |
| IFNL1 | 1.904980421 | GeneCards |
| HERC5 | 1.904358387 | GeneCards |
| IL23A | 1.896306038 | GeneCards |
| CD274 | 1.894291401 | GeneCards |
| RPS27A | 1.893044591 | GeneCards |
| CDK2 | 1.892448425 | GeneCards |
| SMAD2 | 1.880994678 | GeneCards |
| CYP1A2 | 1.878092408 | GeneCards |
| CYP24A1 | 1.8708148 | GeneCards |
| MIR338 | 1.870783687 | GeneCards |
| POU2F1 | 1.868031859 | GeneCards |
| EPRS1 | 1.867111444 | GeneCards |
| IL21R-AS1 | 1.865473628 | GeneCards |
| CYP27B1 | 1.864903331 | GeneCards |
| MYH11 | 1.852205873 | GeneCards |
| PIK3CD | 1.852024436 | GeneCards |
| GSTO1 | 1.850832582 | GeneCards |
| NPS | 1.84975934 | GeneCards |
| ITGB1 | 1.845817089 | GeneCards |
| CD27 | 1.845542908 | GeneCards |
| PCDH1 | 1.837927222 | GeneCards |
| PRKAA2 | 1.836863756 | GeneCards |
| CLEC16A | 1.836713433 | GeneCards |
| TNFRSF1A | 1.83656621 | GeneCards |
| TPSB2 | 1.833979011 | GeneCards |
| PRNP | 1.833580971 | GeneCards |
| TLR8 | 1.831758857 | GeneCards |
| IL37 | 1.829950571 | GeneCards |
| RBM17 | 1.822894692 | GeneCards |
| LTBP1 | 1.82118547 | GeneCards |
| EMSY | 1.819738984 | GeneCards |
| STAT2 | 1.818808794 | GeneCards |
| SLC22A4 | 1.815481544 | GeneCards |
| AQP5 | 1.814365506 | GeneCards |
| EPHX2 | 1.814309359 | GeneCards |
| IGSF3 | 1.81184721 | GeneCards |
| STX1A | 1.810998917 | GeneCards |
| ALAD | 1.810998917 | GeneCards |
| TRIM32 | 1.810998917 | GeneCards |
| NPHP1 | 1.810998917 | GeneCards |
| ATRIP | 1.810998917 | GeneCards |
| TREX1 | 1.810998917 | GeneCards |
| TGM5 | 1.810998917 | GeneCards |
| SUZ12 | 1.810998917 | GeneCards |
| KIF7 | 1.810998917 | GeneCards |
| TTC8 | 1.810998917 | GeneCards |
| LZTFL1 | 1.810998917 | GeneCards |
| MKKS | 1.810998917 | GeneCards |
| DCTN4 | 1.810998917 | GeneCards |
| MIA3 | 1.810998917 | GeneCards |
| CLCA4 | 1.810998917 | GeneCards |
| NCKAP1L | 1.810998917 | GeneCards |
| ASTN2 | 1.810998917 | GeneCards |
| IFT27 | 1.810998917 | GeneCards |
| ARSL | 1.810998917 | GeneCards |
| ZDHHC24 | 1.810998917 | GeneCards |
| BBIP1 | 1.810998917 | GeneCards |
| DOP1A | 1.810998917 | GeneCards |
| DOCK8-AS1 | 1.810998917 | GeneCards |
| MTOR-AS1 | 1.810998917 | GeneCards |
| SEPT5-GP1BB | 1.810998917 | GeneCards |
| LOC105373021 | 1.810998917 | GeneCards |
| ATRIP-TREX1 | 1.810998917 | GeneCards |
| GH-LCR | 1.810998917 | GeneCards |
| LOC107303343 | 1.810998917 | GeneCards |
| LOC113633877 | 1.810998917 | GeneCards |
| LOC113664106 | 1.810998917 | GeneCards |
| LOC111674463 | 1.810998917 | GeneCards |
| LOC111674472 | 1.810998917 | GeneCards |
| LOC111674475 | 1.810998917 | GeneCards |
| LOC111674477 | 1.810998917 | GeneCards |
| LOC106050102 | 1.810998917 | GeneCards |
| DEL16P13.3 | 1.810998917 | GeneCards |
| DUPXQ28 | 1.810998917 | GeneCards |
| PLA2G10 | 1.810549736 | GeneCards |
| IRF1-AS1 | 1.808432221 | GeneCards |
| LBR | 1.807372332 | GeneCards |
| PGAP3 | 1.806055546 | GeneCards |
| HIF1A | 1.804487944 | GeneCards |
| MEFV | 1.80142796 | GeneCards |
| HTR3A | 1.799132109 | GeneCards |
| CYP27A1 | 1.793080091 | GeneCards |
| MIR19A | 1.788301587 | GeneCards |
| PTGIR | 1.784596562 | GeneCards |
| RUNX3 | 1.781425476 | GeneCards |
| NEGR1 | 1.779276013 | GeneCards |
| PSIP1 | 1.777096987 | GeneCards |
| FLG2 | 1.768030405 | GeneCards |
| LY86 | 1.767194152 | GeneCards |
| GPX4 | 1.764520407 | GeneCards |
| ZBTB10 | 1.758459091 | GeneCards |
| P2RX7 | 1.756073594 | GeneCards |
| TAC3 | 1.751330495 | GeneCards |
| GZMA | 1.750995636 | GeneCards |
| PRKCQ | 1.744107008 | GeneCards |
| NEAT1 | 1.743862391 | GeneCards |
| CFL1 | 1.740989447 | GeneCards |
| XDH | 1.740194082 | GeneCards |
| HPGDS | 1.739880085 | GeneCards |
| EDIL3 | 1.735841393 | GeneCards |
| ATP2A2 | 1.730414391 | GeneCards |
| KIAA1109 | 1.730368972 | GeneCards |
| TGM2 | 1.729179859 | GeneCards |
| MIR192 | 1.722464561 | GeneCards |
| ARRB2 | 1.720749378 | GeneCards |
| IL18BP | 1.720749378 | GeneCards |
| TAP2 | 1.718566775 | GeneCards |
| APOA1 | 1.714958429 | GeneCards |
| TPSD1 | 1.713739872 | GeneCards |
| MAPK8 | 1.711586237 | GeneCards |
| PTPN22 | 1.710871696 | GeneCards |
| TNFRSF4 | 1.702628374 | GeneCards |
| HSPA1B | 1.696685195 | GeneCards |
| DEFB4A | 1.686687231 | GeneCards |
| LBP | 1.680480957 | GeneCards |
| KRT19 | 1.679808378 | GeneCards |
| KDR | 1.679520249 | GeneCards |
| PDGFB | 1.677073479 | GeneCards |
| CTSS | 1.676296949 | GeneCards |
| ICAM3 | 1.675954342 | GeneCards |
| MED24 | 1.670673013 | GeneCards |
| MUC22 | 1.670197248 | GeneCards |
| SETDB2 | 1.669134617 | GeneCards |
| HSPA4 | 1.661195278 | GeneCards |
| HDAC9 | 1.660170674 | GeneCards |
| CEBPB | 1.65993166 | GeneCards |
| SLC6A11 | 1.657075644 | GeneCards |
| CCR10 | 1.657075644 | GeneCards |
| SH2B3 | 1.655614018 | GeneCards |
| KRT14 | 1.654719234 | GeneCards |
| HRNR | 1.652209759 | GeneCards |
| CTNNB1 | 1.651111245 | GeneCards |
| BRD2 | 1.650199533 | GeneCards |
| ATF6B | 1.650199533 | GeneCards |
| HCG23 | 1.650199533 | GeneCards |
| PTPRE | 1.647235394 | GeneCards |
| NTRK3 | 1.644078493 | GeneCards |
| LOC100287329 | 1.643600345 | GeneCards |
| GPX3 | 1.641914845 | GeneCards |
| IL20 | 1.641233683 | GeneCards |
| CCR1 | 1.640572667 | GeneCards |
| CPS1 | 1.639336705 | GeneCards |
| MIR196A2 | 1.639336705 | GeneCards |
| SELPLG | 1.634181857 | GeneCards |
| GPX1 | 1.632357836 | GeneCards |
| INSIG2 | 1.631095886 | GeneCards |
| BDKRB2 | 1.630907655 | GeneCards |
| CD63 | 1.630852103 | GeneCards |
| TNFRSF10B | 1.630714178 | GeneCards |
| PTGDS | 1.630714178 | GeneCards |
| SPRR2B | 1.627845287 | GeneCards |
| MBP | 1.626849651 | GeneCards |
| PRKCE | 1.626082778 | GeneCards |
| SLC24A2 | 1.626082778 | GeneCards |
| ABO | 1.626082778 | GeneCards |
| MMRN1 | 1.625023961 | GeneCards |
| GSTM3 | 1.623079658 | GeneCards |
| ANGPT2 | 1.622821093 | GeneCards |
| CASP3 | 1.620780706 | GeneCards |
| ELP1 | 1.620780706 | GeneCards |
| CACNG6 | 1.620038986 | GeneCards |
| ACVRL1 | 1.619140267 | GeneCards |
| EZR | 1.61698246 | GeneCards |
| CSF2RB | 1.612264037 | GeneCards |
| HSPD1 | 1.607663631 | GeneCards |
| LCE3B | 1.607055902 | GeneCards |
| IRF2 | 1.60418272 | GeneCards |
| CCHCR1 | 1.602972627 | GeneCards |
| HLA-DMA | 1.602070689 | GeneCards |
| GNB3 | 1.600636482 | GeneCards |
| PSMB8 | 1.600179672 | GeneCards |
| IL19 | 1.599276781 | GeneCards |
| MMP19 | 1.59842515 | GeneCards |
| FADS2 | 1.59842515 | GeneCards |
| ERBB2 | 1.597955823 | GeneCards |
| SMARCE1 | 1.597855806 | GeneCards |
| CYP1B1 | 1.597801566 | GeneCards |
| GAB1 | 1.597801566 | GeneCards |
| SLC6A12 | 1.597801566 | GeneCards |
| CDH23 | 1.597801566 | GeneCards |
| SLC30A8 | 1.597801566 | GeneCards |
| GPR12 | 1.597801566 | GeneCards |
| CSMD1 | 1.597801566 | GeneCards |
| BMP2 | 1.596862078 | GeneCards |
| COL2A1 | 1.589472294 | GeneCards |
| OPN3 | 1.587927699 | GeneCards |
| BPI | 1.587494612 | GeneCards |
| MIR200B | 1.587494612 | GeneCards |
| IL23R | 1.587363243 | GeneCards |
| VDAC1 | 1.58538866 | GeneCards |
| FPR2 | 1.583966613 | GeneCards |
| DDIT3 | 1.580739021 | GeneCards |
| AIF1 | 1.580739021 | GeneCards |
| PRRC2A | 1.580739021 | GeneCards |
| TMEM132D | 1.580067158 | GeneCards |
| TXK | 1.578948021 | GeneCards |
| CTTN | 1.578826189 | GeneCards |
| FGF2 | 1.576411605 | GeneCards |
| TGM3 | 1.573805094 | GeneCards |
| TMEM79 | 1.573805094 | GeneCards |
| CD247 | 1.573191047 | GeneCards |
| TPSG1 | 1.565150261 | GeneCards |
| SERPINB10 | 1.562349081 | GeneCards |
| SLC11A1 | 1.560082316 | GeneCards |
| ITGB4 | 1.559009314 | GeneCards |
| MMP7 | 1.555612564 | GeneCards |
| PDE3B | 1.553471088 | GeneCards |
| SEMA3E | 1.552393556 | GeneCards |
| BEST1 | 1.550836802 | GeneCards |
| OSM | 1.550074816 | GeneCards |
| RHOA | 1.549642563 | GeneCards |
| INHBA | 1.549402595 | GeneCards |
| BAX | 1.547336102 | GeneCards |
| KRT18 | 1.544165254 | GeneCards |
| IKZF4 | 1.542351246 | GeneCards |
| PMEL | 1.542351246 | GeneCards |
| TSBP1 | 1.542351246 | GeneCards |
| LGALS9 | 1.540683746 | GeneCards |
| TNFRSF18 | 1.540539742 | GeneCards |
| LOC110806262 | 1.540539742 | GeneCards |
| TGFB3 | 1.539215684 | GeneCards |
| CDH26 | 1.537888885 | GeneCards |
| MX1 | 1.532141447 | GeneCards |
| ADCYAP1R1 | 1.530434966 | GeneCards |
| CYP2J2 | 1.530434966 | GeneCards |
| C5orf46 | 1.53025353 | GeneCards |
| TNS1 | 1.530225158 | GeneCards |
| DEFB103B | 1.529769778 | GeneCards |
| KDM4C | 1.528293133 | GeneCards |
| DRD2 | 1.524770141 | GeneCards |
| TNFRSF1B | 1.522277474 | GeneCards |
| BIRC5 | 1.522277474 | GeneCards |
| CXCR5 | 1.520231485 | GeneCards |
| ELF5 | 1.520086646 | GeneCards |
| TAGLN | 1.519842029 | GeneCards |
| ENO1 | 1.515672445 | GeneCards |
| FTO | 1.515327811 | GeneCards |
| MUC4 | 1.515008688 | GeneCards |
| FCRL3 | 1.509476304 | GeneCards |
| CLCN3 | 1.508658409 | GeneCards |
| CD38 | 1.507940412 | GeneCards |
| HLA-DMB | 1.507054567 | GeneCards |
| HLA-DOB | 1.507054567 | GeneCards |
| HLA-DRB5 | 1.507054567 | GeneCards |
| FCAR | 1.505171776 | GeneCards |
| LTB4R2 | 1.50275135 | GeneCards |
| TNC | 1.502686858 | GeneCards |
| SRC | 1.499923229 | GeneCards |
| CA10 | 1.49984324 | GeneCards |
| AOAH | 1.49858284 | GeneCards |
| MDM2 | 1.49572897 | GeneCards |
| NACA | 1.495680571 | GeneCards |
| RPTN | 1.491239071 | GeneCards |
| ITGAV | 1.488895178 | GeneCards |
| S100A7 | 1.488855839 | GeneCards |
| ATOD5 | 1.488855839 | GeneCards |
| ATOD6 | 1.488855839 | GeneCards |
| TRPV4 | 1.487382174 | GeneCards |
| FUT2 | 1.487382174 | GeneCards |
| CREB5 | 1.486004472 | GeneCards |
| KLK11 | 1.48587501 | GeneCards |
| DNASE1L3 | 1.484058738 | GeneCards |
| VCAN | 1.480742335 | GeneCards |
| BTK | 1.4791646 | GeneCards |
| CYP4F22 | 1.477165699 | GeneCards |
| TH2-LCR | 1.475717664 | GeneCards |
| NNMT | 1.475120783 | GeneCards |
| C11orf71 | 1.475120783 | GeneCards |
| LOC101928940 | 1.475120783 | GeneCards |
| HSPA1A | 1.471212149 | GeneCards |
| HDAC7 | 1.469927907 | GeneCards |
| SULT1A3 | 1.465977192 | GeneCards |
| ATF6 | 1.464467287 | GeneCards |
| WIF1 | 1.463945508 | GeneCards |
| GCLM | 1.463945508 | GeneCards |
| MIR199A1 | 1.462535501 | GeneCards |
| MS4A1 | 1.455524445 | GeneCards |
| DSG1 | 1.452982187 | GeneCards |
| MIR125B1 | 1.45240128 | GeneCards |
| SIRT1 | 1.451640129 | GeneCards |
| EBI3 | 1.451640129 | GeneCards |
| IL31RA | 1.450043678 | GeneCards |
| CD83 | 1.446434975 | GeneCards |
| CD48 | 1.445419788 | GeneCards |
| PECAM1 | 1.443182468 | GeneCards |
| ACE2 | 1.438886881 | GeneCards |
| PHB | 1.438809037 | GeneCards |
| MIR16-1 | 1.43858254 | GeneCards |
| CYP2E1 | 1.437994242 | GeneCards |
| PDE4B | 1.437994242 | GeneCards |
| ACKR1 | 1.435071468 | GeneCards |
| ZNF365 | 1.434421062 | GeneCards |
| PTHLH | 1.434218407 | GeneCards |
| RGS2 | 1.431397796 | GeneCards |
| TFAP2A | 1.431002498 | GeneCards |
| ITGB7 | 1.425632954 | GeneCards |
| KCNMB1 | 1.425632954 | GeneCards |
| SOCS5 | 1.425632954 | GeneCards |
| DAP3 | 1.425632954 | GeneCards |
| MIR15A | 1.424120069 | GeneCards |
| VAV3 | 1.420399666 | GeneCards |
| SIM2 | 1.420399666 | GeneCards |
| XKR6 | 1.420399666 | GeneCards |
| TAFA2 | 1.420399666 | GeneCards |
| ZBTB38 | 1.414423347 | GeneCards |
| APC | 1.413006067 | GeneCards |
| GTF2H4 | 1.409578681 | GeneCards |
| F8 | 1.408882618 | GeneCards |
| ACO1 | 1.405937195 | GeneCards |
| ZNF665 | 1.405937195 | GeneCards |
| C9orf24 | 1.40173614 | GeneCards |
| HSPB1 | 1.397351742 | GeneCards |
| IPO13 | 1.397351742 | GeneCards |
| IL1R2 | 1.393955708 | GeneCards |
| TRPC3 | 1.392303467 | GeneCards |
| AHR | 1.390831947 | GeneCards |
| PLA2G6 | 1.390831947 | GeneCards |
| CETP | 1.388779521 | GeneCards |
| FOS | 1.38778019 | GeneCards |
| CCL8 | 1.387314916 | GeneCards |
| SIGIRR | 1.381539822 | GeneCards |
| IL15RA | 1.38054359 | GeneCards |
| SEMA3A | 1.37893033 | GeneCards |
| MIR200C | 1.376920938 | GeneCards |
| MC4R | 1.376780987 | GeneCards |
| PDE10A | 1.376780987 | GeneCards |
| GBE1 | 1.376780987 | GeneCards |
| ROBO1 | 1.376780987 | GeneCards |
| DGKG | 1.376780987 | GeneCards |
| SH2B1 | 1.376780987 | GeneCards |
| STK33 | 1.376780987 | GeneCards |
| CHST8 | 1.376780987 | GeneCards |
| MTCH2 | 1.376780987 | GeneCards |
| GNPDA2 | 1.376780987 | GeneCards |
| ETV5 | 1.376780987 | GeneCards |
| KCTD15 | 1.376780987 | GeneCards |
| CTNNBL1 | 1.376780987 | GeneCards |
| RASAL2 | 1.376780987 | GeneCards |
| SEC16B | 1.376780987 | GeneCards |
| FAIM2 | 1.376780987 | GeneCards |
| TMEM18 | 1.376780987 | GeneCards |
| BCDIN3D | 1.376780987 | GeneCards |
| COL6A5 | 1.375721097 | GeneCards |
| PCDH20 | 1.374655128 | GeneCards |
| CTRL | 1.374127984 | GeneCards |
| PTEN | 1.373961449 | GeneCards |
| KPNB1 | 1.37196672 | GeneCards |
| IRAK1 | 1.367502689 | GeneCards |
| KCNJ11 | 1.367475033 | GeneCards |
| HRH3 | 1.366069674 | GeneCards |
| FSIP1 | 1.366069674 | GeneCards |
| SCG3 | 1.362772226 | GeneCards |
| DMXL2 | 1.362772226 | GeneCards |
| ORMDL2 | 1.358189344 | GeneCards |
| H2AC18 | 1.356342793 | GeneCards |
| MIR124-1 | 1.355829716 | GeneCards |
| COL1A1 | 1.353871703 | GeneCards |
| TNFRSF6B | 1.348953605 | GeneCards |
| RAB11FIP2 | 1.348953605 | GeneCards |
| CRISPLD2 | 1.348953605 | GeneCards |
| RAP1GAP2 | 1.348953605 | GeneCards |
| C6orf118 | 1.348953605 | GeneCards |
| MMADHC-DT | 1.348953605 | GeneCards |
| RIPK2 | 1.348835468 | GeneCards |
| PTAFR | 1.348835468 | GeneCards |
| PKDCC | 1.348835468 | GeneCards |
| LOC111365141 | 1.348835468 | GeneCards |
| KRT13 | 1.340417981 | GeneCards |
| PLG | 1.340353012 | GeneCards |
| IL17RA | 1.339284301 | GeneCards |
| TOP2A | 1.338699102 | GeneCards |
| CYP21A2 | 1.338699102 | GeneCards |
| MAP3K7 | 1.338425994 | GeneCards |
| CEBPA | 1.338425994 | GeneCards |
| POLI | 1.337414861 | GeneCards |
| TNFAIP8L2 | 1.337300301 | GeneCards |
| BECN1 | 1.334491134 | GeneCards |
| DCLK1 | 1.334491134 | GeneCards |
| XRCC3 | 1.334491134 | GeneCards |
| C1orf100 | 1.334491134 | GeneCards |
| AXL | 1.330562949 | GeneCards |
| CD276 | 1.328389645 | GeneCards |
| ST2 | 1.328389645 | GeneCards |
| MYD88 | 1.327877879 | GeneCards |
| NTRK2 | 1.326799035 | GeneCards |
| LGALS1 | 1.326799035 | GeneCards |
| MIR223 | 1.326161742 | GeneCards |
| CLU | 1.325398684 | GeneCards |
| MRC1 | 1.325398684 | GeneCards |
| ITPR3 | 1.319285512 | GeneCards |
| PSAP | 1.319285512 | GeneCards |
| XPR1 | 1.319285512 | GeneCards |
| SMAD7 | 1.319285512 | GeneCards |
| AIMP1 | 1.319285512 | GeneCards |
| HPSE2 | 1.319285512 | GeneCards |
| ATG3 | 1.319285512 | GeneCards |
| MKLN1 | 1.319285512 | GeneCards |
| ABI3BP | 1.319285512 | GeneCards |
| KLHL5 | 1.319285512 | GeneCards |
| UTS2 | 1.318041444 | GeneCards |
| GAST | 1.314196587 | GeneCards |
| AKR1C3 | 1.312396288 | GeneCards |
| LCK | 1.312336683 | GeneCards |
| ZAP70 | 1.312336683 | GeneCards |
| TJP1 | 1.312336683 | GeneCards |
| CCR9 | 1.312336683 | GeneCards |
| TFRC | 1.311432362 | GeneCards |
| FBN2 | 1.310085177 | GeneCards |
| PLA2R1 | 1.310085177 | GeneCards |
| MIR9-1 | 1.309049129 | GeneCards |
| TRPM8 | 1.30833745 | GeneCards |
| CALB2 | 1.308327079 | GeneCards |
| CYP2D6 | 1.307430983 | GeneCards |
| PTGFR | 1.307430983 | GeneCards |
| PTPN11 | 1.305801153 | GeneCards |
| RAC2 | 1.303209066 | GeneCards |
| NCOA1 | 1.303209066 | GeneCards |
| PCDH12 | 1.303209066 | GeneCards |
| MIRLET7A1 | 1.300340056 | GeneCards |
| CCL19 | 1.297131062 | GeneCards |
| S1PR1 | 1.296537519 | GeneCards |
| EHF | 1.296537519 | GeneCards |
| CHML | 1.296537519 | GeneCards |
| STIN2-VNTR | 1.296537519 | GeneCards |
| MIR29C | 1.292968273 | GeneCards |
| FSTL1 | 1.287086129 | GeneCards |
| TEK | 1.286092162 | GeneCards |
| CFD | 1.286092162 | GeneCards |
| TGFBR3 | 1.285336971 | GeneCards |
| ADAM12 | 1.285336971 | GeneCards |
| TNKS | 1.283230305 | GeneCards |
| IL26 | 1.281054497 | GeneCards |
| CSK | 1.280854821 | GeneCards |
| ROM1 | 1.280854821 | GeneCards |
| MIR125A | 1.274578452 | GeneCards |
| ACAA1 | 1.273801804 | GeneCards |
| NRIP2 | 1.273801804 | GeneCards |
| EGR1 | 1.270642281 | GeneCards |
| KLF2 | 1.267702222 | GeneCards |
| ADGRA1 | 1.267702222 | GeneCards |
| P2RY2 | 1.263737917 | GeneCards |
| GUSB | 1.26087606 | GeneCards |
| NOTCH1 | 1.260324955 | GeneCards |
| TERT | 1.256613612 | GeneCards |
| PPL | 1.254580617 | GeneCards |
| MIR222 | 1.254580617 | GeneCards |
| PIN1 | 1.249860406 | GeneCards |
| NRG1 | 1.249594927 | GeneCards |
| NAT1 | 1.249594927 | GeneCards |
| SNW1 | 1.249594927 | GeneCards |
| MYCN | 1.247704387 | GeneCards |
| OPRK1 | 1.247704387 | GeneCards |
| DDX1 | 1.247704387 | GeneCards |
| CDH13 | 1.247704387 | GeneCards |
| DGKH | 1.247704387 | GeneCards |
| TLN1 | 1.247704387 | GeneCards |
| TBCD | 1.247704387 | GeneCards |
| CRIM1 | 1.247704387 | GeneCards |
| DOCK10 | 1.247704387 | GeneCards |
| FLRT2 | 1.247704387 | GeneCards |
| FBXL7 | 1.247704387 | GeneCards |
| SYNPO2 | 1.247704387 | GeneCards |
| MPP7 | 1.247704387 | GeneCards |
| ZNF71 | 1.247704387 | GeneCards |
| COMMD10 | 1.247704387 | GeneCards |
| VSIR | 1.247704387 | GeneCards |
| OR6X1 | 1.247704387 | GeneCards |
| DCDC2C | 1.247704387 | GeneCards |
| GNG5P5 | 1.247704387 | GeneCards |
| KRT8P16 | 1.247704387 | GeneCards |
| GBA | 1.246652126 | GeneCards |
| TPMT | 1.246652126 | GeneCards |
| TGM1 | 1.246652126 | GeneCards |
| CANX | 1.246652126 | GeneCards |
| CTSE | 1.246652126 | GeneCards |
| KLK5 | 1.246652126 | GeneCards |
| LALBA | 1.246652126 | GeneCards |
| CSN3 | 1.246652126 | GeneCards |
| DEFB103A | 1.246652126 | GeneCards |
| ATOD7 | 1.246652126 | GeneCards |
| ATOD8 | 1.246652126 | GeneCards |
| ATOD9 | 1.246652126 | GeneCards |
| HSP90AB1 | 1.245547771 | GeneCards |
| PHLDB1 | 1.24470365 | GeneCards |
| CD163 | 1.243599415 | GeneCards |
| KRT1 | 1.24045527 | GeneCards |
| ETS1 | 1.239837408 | GeneCards |
| SMAD6 | 1.239026189 | GeneCards |
| RNASEH2C | 1.234742165 | GeneCards |
| EDNRB | 1.233229637 | GeneCards |
| BDKRB1 | 1.233229637 | GeneCards |
| MIR30A | 1.232460856 | GeneCards |
| MIR23A | 1.232460856 | GeneCards |
| ORAI1 | 1.230577707 | GeneCards |
| HSD11B2 | 1.22752285 | GeneCards |
| BIRC3 | 1.22752285 | GeneCards |
| ANO3 | 1.225584626 | GeneCards |
| MUC15 | 1.225584626 | GeneCards |
| CHD7 | 1.225350142 | GeneCards |
| GRN | 1.223587751 | GeneCards |
| DOCK1 | 1.223587751 | GeneCards |
| FUT3 | 1.223587751 | GeneCards |
| SULT1A1 | 1.223587751 | GeneCards |
| LPA | 1.223587751 | GeneCards |
| SNX9 | 1.223587751 | GeneCards |
| NOX3 | 1.223587751 | GeneCards |
| IGHG1 | 1.223587751 | GeneCards |
| FANK1 | 1.223587751 | GeneCards |
| TAC4 | 1.223587751 | GeneCards |
| MIR98 | 1.223587751 | GeneCards |
| AAA1 | 1.223587751 | GeneCards |
| FYN | 1.221435189 | GeneCards |
| SLC9A1 | 1.220495701 | GeneCards |
| GCG | 1.220495701 | GeneCards |
| ATF3 | 1.214636564 | GeneCards |
| PSMB9 | 1.214361429 | GeneCards |
| MIR203A | 1.209769011 | GeneCards |
| CIITA | 1.209196925 | GeneCards |
| MIR149 | 1.20735383 | GeneCards |
| MIRLET7B | 1.20735383 | GeneCards |
| BMP4 | 1.203230381 | GeneCards |
| MGAM | 1.203230381 | GeneCards |
| NEDD4L | 1.2004776 | GeneCards |
| HSD3B1 | 1.2004776 | GeneCards |
| MALAT1 | 1.2004776 | GeneCards |
| MAPK10 | 1.199599981 | GeneCards |
| COTL1 | 1.198883295 | GeneCards |
| CSF1R | 1.19530654 | GeneCards |
| SOX9 | 1.19530654 | GeneCards |
| ATP2A3 | 1.19530654 | GeneCards |
| S100B | 1.19530654 | GeneCards |
| AGL | 1.19530654 | GeneCards |
| DKK1 | 1.19530654 | GeneCards |
| GNGT1 | 1.19530654 | GeneCards |
| PDE9A | 1.19530654 | GeneCards |
| PON2 | 1.19530654 | GeneCards |
| CTNND2 | 1.19530654 | GeneCards |
| ANK1 | 1.19530654 | GeneCards |
| TUSC3 | 1.19530654 | GeneCards |
| SFRP2 | 1.19530654 | GeneCards |
| UGT1A6 | 1.19530654 | GeneCards |
| KANK1 | 1.19530654 | GeneCards |
| TNFAIP1 | 1.19530654 | GeneCards |
| TREM1 | 1.19530654 | GeneCards |
| PLXNA4 | 1.19530654 | GeneCards |
| SLC24A3 | 1.19530654 | GeneCards |
| SLC6A7 | 1.19530654 | GeneCards |
| PCDH15 | 1.19530654 | GeneCards |
| SFRP5 | 1.19530654 | GeneCards |
| TAF7 | 1.19530654 | GeneCards |
| CNTLN | 1.19530654 | GeneCards |
| SVEP1 | 1.19530654 | GeneCards |
| IGHG3 | 1.19530654 | GeneCards |
| RPS28P1 | 1.19530654 | GeneCards |
| CTNNA1 | 1.192016125 | GeneCards |
| NIPAL4 | 1.192007065 | GeneCards |
| MIR26A1 | 1.191049099 | GeneCards |
| MIR140 | 1.189998627 | GeneCards |
| IKBKB | 1.186067343 | GeneCards |
| KCNN4 | 1.186067343 | GeneCards |
| GLB1 | 1.185130954 | GeneCards |
| KCNQ1 | 1.183992743 | GeneCards |
| TTR | 1.183992743 | GeneCards |
| NPPB | 1.18344152 | GeneCards |
| GRP | 1.18344152 | GeneCards |
| ITGAX | 1.180100918 | GeneCards |
| FOXO3 | 1.180100918 | GeneCards |
| TRG | 1.180100918 | GeneCards |
| SMAD4 | 1.178368092 | GeneCards |
| CASR | 1.178123355 | GeneCards |
| NTRK1 | 1.178123355 | GeneCards |
| CYP11A1 | 1.170695901 | GeneCards |
| PPARGC1A | 1.170695901 | GeneCards |
| JUND | 1.170695901 | GeneCards |
| HLA-E | 1.170695901 | GeneCards |
| SMOC2 | 1.170695901 | GeneCards |
| SLC5A12 | 1.170695901 | GeneCards |
| MIR26A2 | 1.166842103 | GeneCards |
| PROC | 1.164024472 | GeneCards |
| GPR1 | 1.164024472 | GeneCards |
| GPRASP1 | 1.164024472 | GeneCards |
| MT-CO2 | 1.164024472 | GeneCards |
| MIR499A | 1.164024472 | GeneCards |
| PIK3CB | 1.153882146 | GeneCards |
| AGT | 1.153612256 | GeneCards |
| MMP21 | 1.150101542 | GeneCards |
| MMP28 | 1.150101542 | GeneCards |
| SCNN1A | 1.148341656 | GeneCards |
| ALOX12 | 1.147659183 | GeneCards |
| ACVR1 | 1.146907568 | GeneCards |
| TPO | 1.146907568 | GeneCards |
| DUSP10 | 1.146907568 | GeneCards |
| CD1D | 1.146907568 | GeneCards |
| MAOA | 1.144629359 | GeneCards |
| ADRA1A | 1.144629359 | GeneCards |
| GNAI1 | 1.137753129 | GeneCards |
| FCGR2B | 1.137483716 | GeneCards |
| MUC6 | 1.136756539 | GeneCards |
| UBE3C | 1.132535696 | GeneCards |
| FTL | 1.13188374 | GeneCards |
| ADAM23 | 1.128517628 | GeneCards |
| RCBTB1 | 1.128517628 | GeneCards |
| TOR1B | 1.12602067 | GeneCards |
| B3GAT1 | 1.124791384 | GeneCards |
| LPL | 1.115398884 | GeneCards |
| PTPRC | 1.111098289 | GeneCards |
| NFATC1 | 1.108519793 | GeneCards |
| BMP7 | 1.108519793 | GeneCards |
| SPINT2 | 1.108519793 | GeneCards |
| IGKV2D-29 | 1.108519793 | GeneCards |
| CCR5AS | 1.108519793 | GeneCards |
| NCOA2 | 1.106528401 | GeneCards |
| EFEMP2 | 1.106513143 | GeneCards |
| MPZ | 1.106513143 | GeneCards |
| MIR28 | 1.106513143 | GeneCards |
| REN | 1.106366277 | GeneCards |
| CEACAM3 | 1.104779243 | GeneCards |
| SMPD1 | 1.102390051 | GeneCards |
| NGFR | 1.100496769 | GeneCards |
| CD160 | 1.100496769 | GeneCards |
| CSNK2B | 1.098473907 | GeneCards |
| CYBB | 1.093244195 | GeneCards |
| NOX4 | 1.093244195 | GeneCards |
| IL10RB | 1.09270978 | GeneCards |
| TGFA | 1.089249372 | GeneCards |
| COL5A1 | 1.089192629 | GeneCards |
| BMPR1B | 1.086400032 | GeneCards |
| BMPR2 | 1.086400032 | GeneCards |
| GATA2 | 1.086400032 | GeneCards |
| NTF4 | 1.086400032 | GeneCards |
| SERPINB3 | 1.086400032 | GeneCards |
| IKZF2 | 1.086400032 | GeneCards |
| ANKEF1 | 1.086400032 | GeneCards |
| BCL2A1 | 1.083379984 | GeneCards |
| WNT5A | 1.081342578 | GeneCards |
| NR1H2 | 1.081342578 | GeneCards |
| EPO | 1.081342578 | GeneCards |
| PLAT | 1.078247309 | GeneCards |
| GYPA | 1.078247309 | GeneCards |
| FLT3LG | 1.078247309 | GeneCards |
| MEN1 | 1.077282667 | GeneCards |
| EHMT1 | 1.077282667 | GeneCards |
| TP53 | 1.071129441 | GeneCards |
| LY96 | 1.069037199 | GeneCards |
| PRODH | 1.067794442 | GeneCards |
| ANPEP | 1.063041687 | GeneCards |
| ZMPSTE24 | 1.061293006 | GeneCards |
| GPR183 | 1.05921948 | GeneCards |
| ABL1 | 1.056283951 | GeneCards |
| SERPINE2 | 1.054730892 | GeneCards |
| PYY | 1.052780151 | GeneCards |
| SLC6A2 | 1.050928473 | GeneCards |
| DRD4 | 1.050928473 | GeneCards |
| PTK7 | 1.050928473 | GeneCards |
| AKT1 | 1.050861597 | GeneCards |
| HMGCR | 1.050861597 | GeneCards |
| APOE | 1.049970031 | GeneCards |
| CD200 | 1.046965122 | GeneCards |
| RETNLB | 1.046965122 | GeneCards |
| MYDGF | 1.046965122 | GeneCards |
| SEMA4A | 1.044992208 | GeneCards |
| INSR | 1.044052243 | GeneCards |
| GYS1 | 1.044052243 | GeneCards |
| RHO | 1.044052243 | GeneCards |
| ADRA1D | 1.044052243 | GeneCards |
| PNMT | 1.044052243 | GeneCards |
| IAPP | 1.044052243 | GeneCards |
| HCRT | 1.044052243 | GeneCards |
| MAF | 1.043030024 | GeneCards |
| ADAMTS9 | 1.038182855 | GeneCards |
| ALPP | 1.031511307 | GeneCards |
| ELAC2 | 1.031511307 | GeneCards |
| GGT1 | 1.029750228 | GeneCards |
| KIFC1 | 1.02943778 | GeneCards |
| LTBR | 1.029211283 | GeneCards |
| GZMK | 1.029211283 | GeneCards |
| F5 | 1.028741837 | GeneCards |
| VWF | 1.021697998 | GeneCards |
| SLC18A2 | 1.021697998 | GeneCards |
| GP6 | 1.021697998 | GeneCards |
| CHGA | 1.021697998 | GeneCards |
| ANXA1 | 1.019031405 | GeneCards |
| PLCB1 | 1.014748812 | GeneCards |
| HDAC5 | 1.014748812 | GeneCards |
| A2M | 1.014748812 | GeneCards |
| LOX | 1.014748812 | GeneCards |
| MTNR1A | 1.014748812 | GeneCards |
| RGS4 | 1.014748812 | GeneCards |
| TNFSF12 | 1.014748812 | GeneCards |
| LOC110973015 | 1.014748812 | GeneCards |
| CASP10 | 1.01327312 | GeneCards |
| CFLAR | 1.01327312 | GeneCards |
| IL24 | 1.011458397 | GeneCards |
| TRPC1 | 1.011458397 | GeneCards |
| DCBLD2 | 1.011458397 | GeneCards |
| ADAM17 | 1.010491371 | GeneCards |
| RAF1 | 1.00962007 | GeneCards |
| HCG27 | 1.004773021 | GeneCards |
| ZNF331 | 1.003647327 | GeneCards |
| PTK2 | 1.00363481 | GeneCards |
| PPIA | 1.002535582 | GeneCards |
| F10 | 0.999543309 | GeneCards |
| PPP1R12A | 0.999543309 | GeneCards |
| LRG1 | 0.999543309 | GeneCards |
| FCRL6 | 0.999543309 | GeneCards |
| STIP1 | 0.997765362 | GeneCards |
| CCL15 | 0.997765362 | GeneCards |
| LELP1 | 0.996438384 | GeneCards |
| RELA | 0.995801389 | GeneCards |
| NR3C2 | 0.992699206 | GeneCards |
| CCL25 | 0.991460562 | GeneCards |
| TRIM26 | 0.99062562 | GeneCards |
| SCP2 | 0.989294946 | GeneCards |
| STOML2 | 0.988236606 | GeneCards |
| IGF1 | 0.985292435 | GeneCards |
| ELAVL2 | 0.984262645 | GeneCards |
| ABCA1 | 0.983466744 | GeneCards |
| NMUR1 | 0.983466744 | GeneCards |
| TET1 | 0.983466744 | GeneCards |
| TRA | 0.983466744 | GeneCards |
| TRB | 0.983466744 | GeneCards |
| SPTBN2 | 0.982430637 | GeneCards |
| DRD3 | 0.981433511 | GeneCards |
| SLC2A10 | 0.981433511 | GeneCards |
| MLN | 0.981433511 | GeneCards |
| RCOR1 | 0.978585184 | GeneCards |
| MIR34C | 0.978585184 | GeneCards |
| ADCY1 | 0.973853171 | GeneCards |
| ADRA2A | 0.973853171 | GeneCards |
| IL1RAPL2 | 0.973853171 | GeneCards |
| GRK2 | 0.973853171 | GeneCards |
| TRA2B | 0.973531187 | GeneCards |
| RPL32P28 | 0.972160041 | GeneCards |
| IFNAR1 | 0.969340801 | GeneCards |
| NETO1 | 0.966902494 | GeneCards |
| XIAP | 0.96634984 | GeneCards |
| HYAL1 | 0.96634984 | GeneCards |
| HHIP | 0.96634984 | GeneCards |
| TRD | 0.96634984 | GeneCards |
| MAP3K1 | 0.9602772 | GeneCards |
| MFAP4 | 0.9602772 | GeneCards |
| RPS6KA3 | 0.959079266 | GeneCards |
| FLNA | 0.959079266 | GeneCards |
| MECP2 | 0.959079266 | GeneCards |
| SLC26A2 | 0.959079266 | GeneCards |
| COL5A2 | 0.959079266 | GeneCards |
| KLK4 | 0.959079266 | GeneCards |
| KLK6 | 0.959079266 | GeneCards |
| DSC1 | 0.959079266 | GeneCards |
| KLK14 | 0.959079266 | GeneCards |
| DCD | 0.959079266 | GeneCards |
| ZNF750 | 0.959079266 | GeneCards |
| SPINK9 | 0.959079266 | GeneCards |
| TNFSF14 | 0.950056851 | GeneCards |
| RTEL1 | 0.950056851 | GeneCards |
| NAGLU | 0.949354768 | GeneCards |
| KRT24 | 0.949354768 | GeneCards |
| MEI1 | 0.949354768 | GeneCards |
| TNFRSF9 | 0.94795996 | GeneCards |
| FGG | 0.946458519 | GeneCards |
| FGB | 0.946458519 | GeneCards |
| PRDX5 | 0.946458519 | GeneCards |
| PIM2 | 0.946458519 | GeneCards |
| NFIA | 0.946458519 | GeneCards |
| NAGA | 0.946458519 | GeneCards |
| ALOX15B | 0.946458519 | GeneCards |
| KCNIP4 | 0.946458519 | GeneCards |
| MUC19 | 0.946458519 | GeneCards |
| P2RX3 | 0.941015959 | GeneCards |
| DNASE1 | 0.940095305 | GeneCards |
| EIF2AK2 | 0.938571215 | GeneCards |
| WNT11 | 0.937503219 | GeneCards |
| NAB2 | 0.937503219 | GeneCards |
| SLC22A1 | 0.935040951 | GeneCards |
| DUSP19 | 0.935040951 | GeneCards |
| PRMT1 | 0.932939947 | GeneCards |
| GRM1 | 0.931996047 | GeneCards |
| RAC1 | 0.931996047 | GeneCards |
| ITPR1 | 0.931996047 | GeneCards |
| F13A1 | 0.931996047 | GeneCards |
| SMARCD1 | 0.931996047 | GeneCards |
| DAD1 | 0.931996047 | GeneCards |
| MIR124-2 | 0.931996047 | GeneCards |
| MIR124-3 | 0.931996047 | GeneCards |
| MIR133A1 | 0.931996047 | GeneCards |
| LOC109286563 | 0.931996047 | GeneCards |
| ERBB3 | 0.931291461 | GeneCards |
| TYRO3 | 0.931291461 | GeneCards |
| CCN4 | 0.927962184 | GeneCards |
| RIOX2 | 0.927962184 | GeneCards |
| ENO2 | 0.922025144 | GeneCards |
| STARD7 | 0.919883549 | GeneCards |
| ADAM9 | 0.916790485 | GeneCards |
| NR1I2 | 0.916790485 | GeneCards |
| TIMP3 | 0.916790485 | GeneCards |
| ARRB1 | 0.916790485 | GeneCards |
| HAS2 | 0.916790485 | GeneCards |
| DMRT1 | 0.916790485 | GeneCards |
| DUSP2 | 0.916790485 | GeneCards |
| PDCD1LG2 | 0.916790485 | GeneCards |
| CEACAM6 | 0.916790485 | GeneCards |
| UCN3 | 0.916790485 | GeneCards |
| MIR625 | 0.916790485 | GeneCards |
| GSTK1 | 0.916655481 | GeneCards |
| HDAC4 | 0.915148973 | GeneCards |
| CXCL13 | 0.914452076 | GeneCards |
| STUB1 | 0.913576663 | GeneCards |
| IFITM3 | 0.908834696 | GeneCards |
| IFITM1 | 0.908834696 | GeneCards |
| IFITM2 | 0.908834696 | GeneCards |
| CHEK1 | 0.905842364 | GeneCards |
| PPP5C | 0.905842364 | GeneCards |
| XCL1 | 0.905842364 | GeneCards |
| TAS2R1 | 0.905842364 | GeneCards |
| BCL6 | 0.901509881 | GeneCards |
| FOXA3 | 0.901509881 | GeneCards |
| HDAC3 | 0.90071398 | GeneCards |
| CTH | 0.90071398 | GeneCards |
| PLCB3 | 0.90071398 | GeneCards |
| ECE1 | 0.90071398 | GeneCards |
| PLCB4 | 0.90071398 | GeneCards |
| ITPR2 | 0.90071398 | GeneCards |
| CD3E | 0.90071398 | GeneCards |
| PLCB2 | 0.90071398 | GeneCards |
| ADCY7 | 0.90071398 | GeneCards |
| SMARCB1 | 0.90071398 | GeneCards |
| HTR4 | 0.90071398 | GeneCards |
| ICAM2 | 0.90071398 | GeneCards |
| PLN | 0.90071398 | GeneCards |
| HAT1 | 0.90071398 | GeneCards |
| GRK4 | 0.90071398 | GeneCards |
| XRCC1 | 0.90071398 | GeneCards |
| NR0B2 | 0.90071398 | GeneCards |
| CPM | 0.90071398 | GeneCards |
| ETS2 | 0.90071398 | GeneCards |
| CREM | 0.90071398 | GeneCards |
| BATF | 0.90071398 | GeneCards |
| MFNG | 0.90071398 | GeneCards |
| RECK | 0.90071398 | GeneCards |
| CREB3L2 | 0.90071398 | GeneCards |
| GRK7 | 0.90071398 | GeneCards |
| BST2 | 0.90071398 | GeneCards |
| CRHBP | 0.90071398 | GeneCards |
| RGS12 | 0.90071398 | GeneCards |
| RGS16 | 0.90071398 | GeneCards |
| NEIL1 | 0.90071398 | GeneCards |
| FCRL5 | 0.90071398 | GeneCards |
| TXNIP | 0.90071398 | GeneCards |
| RACK1 | 0.90071398 | GeneCards |
| ATPAF1 | 0.90071398 | GeneCards |
| MS4A3 | 0.90071398 | GeneCards |
| CREBL2 | 0.90071398 | GeneCards |
| SFSWAP | 0.90071398 | GeneCards |
| MIR629 | 0.90071398 | GeneCards |
| RXRB | 0.900006294 | GeneCards |
| HLA-DPB2 | 0.900006294 | GeneCards |
| PDE11A | 0.89899826 | GeneCards |
| CAMK4 | 0.898690999 | GeneCards |
| IL1RL2 | 0.898690999 | GeneCards |
| ADAD1 | 0.898690999 | GeneCards |
| ITGA7 | 0.898586154 | GeneCards |
| BPIFA1 | 0.892195821 | GeneCards |
| UBC | 0.888469577 | GeneCards |
| NT5E | 0.883597076 | GeneCards |
| SCD | 0.883597076 | GeneCards |
| COL1A2 | 0.883597076 | GeneCards |
| AQP1 | 0.883597076 | GeneCards |
| FOXA2 | 0.883597076 | GeneCards |
| ADAMTS1 | 0.883597076 | GeneCards |
| IL1RAPL1 | 0.883597076 | GeneCards |
| TRPV2 | 0.883597076 | GeneCards |
| PAPPA | 0.883597076 | GeneCards |
| ADAMTS12 | 0.883597076 | GeneCards |
| SEMA3D | 0.883597076 | GeneCards |
| MYH15 | 0.883597076 | GeneCards |
| CLEC12A | 0.883597076 | GeneCards |
| ADAMTS15 | 0.883597076 | GeneCards |
| CRTAM | 0.883597076 | GeneCards |
| ERO1B | 0.883597076 | GeneCards |
| OR2AG2 | 0.883597076 | GeneCards |
| CHRNA7 | 0.883380592 | GeneCards |
| STS | 0.883212984 | GeneCards |
| NPTN | 0.883212984 | GeneCards |
| MIR10A | 0.883212984 | GeneCards |
| PDHA1 | 0.880735278 | GeneCards |
| PEBP1 | 0.879172444 | GeneCards |
| SPRY2 | 0.87243247 | GeneCards |
| TRAF6 | 0.867304087 | GeneCards |
| ENPP1 | 0.865207195 | GeneCards |
| PRLR | 0.865207195 | GeneCards |
| TNFRSF13B | 0.865207195 | GeneCards |
| TEC | 0.865207195 | GeneCards |
| CR2 | 0.865207195 | GeneCards |
| NPR3 | 0.865207195 | GeneCards |
| VNN1 | 0.865207195 | GeneCards |
| SLC7A1 | 0.865207195 | GeneCards |
| SFRP4 | 0.865207195 | GeneCards |
| ATG7 | 0.865207195 | GeneCards |
| LGALS3BP | 0.865207195 | GeneCards |
| FFAR2 | 0.865207195 | GeneCards |
| CLDN18 | 0.865207195 | GeneCards |
| ELF3 | 0.865207195 | GeneCards |
| DDO | 0.865207195 | GeneCards |
| AGMAT | 0.865207195 | GeneCards |
| AANAT | 0.865207195 | GeneCards |
| SRSF3 | 0.865207195 | GeneCards |
| RARRES2 | 0.865207195 | GeneCards |
| CLEC4D | 0.865207195 | GeneCards |
| UCN | 0.865207195 | GeneCards |
| ZFR | 0.865207195 | GeneCards |
| PHTF1 | 0.865207195 | GeneCards |
| CXCL17 | 0.865207195 | GeneCards |
| CCL3L1 | 0.865207195 | GeneCards |
| METTL24 | 0.865207195 | GeneCards |
| CDKN2B-AS1 | 0.865207195 | GeneCards |
| MIR943 | 0.865207195 | GeneCards |
| CTSK | 0.862697661 | GeneCards |
| AGFG1 | 0.862697661 | GeneCards |
| MGA | 0.862697661 | GeneCards |
| LPP | 0.859436572 | GeneCards |
| SLCO1B1 | 0.856144488 | GeneCards |
| PDPK1 | 0.850953698 | GeneCards |
| CTSH | 0.850953698 | GeneCards |
| GNB1 | 0.850953698 | GeneCards |
| APOH | 0.850953698 | GeneCards |
| PRSS21 | 0.850953698 | GeneCards |
| CISH | 0.850187182 | GeneCards |
| TNFRSF14 | 0.850187182 | GeneCards |
| IL2RG | 0.850078225 | GeneCards |
| PDGFRB | 0.845824957 | GeneCards |
| BLK | 0.84520936 | GeneCards |
| HEXB | 0.84520936 | GeneCards |
| ADCY3 | 0.84520936 | GeneCards |
| NAMPT | 0.84520936 | GeneCards |
| USP15 | 0.84520936 | GeneCards |
| PLD2 | 0.84520936 | GeneCards |
| AQP4 | 0.84520936 | GeneCards |
| MUSK | 0.84520936 | GeneCards |
| TXN2 | 0.84520936 | GeneCards |
| LAMA4 | 0.84520936 | GeneCards |
| GCLC | 0.84520936 | GeneCards |
| RAMP1 | 0.84520936 | GeneCards |
| NOX1 | 0.84520936 | GeneCards |
| ACSL3 | 0.84520936 | GeneCards |
| NR6A1 | 0.84520936 | GeneCards |
| SOCS6 | 0.84520936 | GeneCards |
| HAS1 | 0.84520936 | GeneCards |
| ITGAE | 0.84520936 | GeneCards |
| LRRTM1 | 0.84520936 | GeneCards |
| ESM1 | 0.84520936 | GeneCards |
| SYNE2 | 0.84520936 | GeneCards |
| SLC45A3 | 0.84520936 | GeneCards |
| PDCD5 | 0.84520936 | GeneCards |
| KIR2DL4 | 0.84520936 | GeneCards |
| FFAR3 | 0.84520936 | GeneCards |
| WDR11 | 0.84520936 | GeneCards |
| PMEPA1 | 0.84520936 | GeneCards |
| PCDH9 | 0.84520936 | GeneCards |
| LAG3 | 0.84520936 | GeneCards |
| FAM167A | 0.84520936 | GeneCards |
| CPO | 0.84520936 | GeneCards |
| BARHL2 | 0.84520936 | GeneCards |
| MEG3 | 0.84520936 | GeneCards |
| DLEU7 | 0.84520936 | GeneCards |
| DLEU1 | 0.84520936 | GeneCards |
| DEUP1 | 0.84520936 | GeneCards |
| MIR1-1 | 0.84520936 | GeneCards |
| MIR1-2 | 0.84520936 | GeneCards |
| ZDHHC8P1 | 0.84520936 | GeneCards |
| MIR3162 | 0.84520936 | GeneCards |
| RPL10AP3 | 0.84520936 | GeneCards |
| PDE9A-AS1 | 0.84520936 | GeneCards |
| NASPP1 | 0.84520936 | GeneCards |
| HNRNPA1P2 | 0.84520936 | GeneCards |
| STARP1 | 0.84520936 | GeneCards |
| RPL34P26 | 0.84520936 | GeneCards |
| RPS3P6 | 0.84520936 | GeneCards |
| RNU7-51P | 0.84520936 | GeneCards |
| LOC102724802 | 0.84520936 | GeneCards |
| PIGR | 0.841340065 | GeneCards |
| CFH | 0.831797302 | GeneCards |
| PTPRT | 0.831797302 | GeneCards |
| ID2 | 0.829654932 | GeneCards |
| SCG2 | 0.829654932 | GeneCards |
| LINC00299 | 0.829654932 | GeneCards |
| ANO1 | 0.827832937 | GeneCards |
| MRGPRX2 | 0.827832937 | GeneCards |
| IRF5 | 0.824971139 | GeneCards |
| MMP14 | 0.8230896 | GeneCards |
| GJA1 | 0.8230896 | GeneCards |
| COL4A1 | 0.8230896 | GeneCards |
| FGR | 0.8230896 | GeneCards |
| FST | 0.8230896 | GeneCards |
| SMC3 | 0.8230896 | GeneCards |
| FABP4 | 0.8230896 | GeneCards |
| FBLN1 | 0.8230896 | GeneCards |
| CITED2 | 0.8230896 | GeneCards |
| LPIN2 | 0.8230896 | GeneCards |
| PDCD4 | 0.8230896 | GeneCards |
| CD1C | 0.8230896 | GeneCards |
| SSH1 | 0.8230896 | GeneCards |
| CD207 | 0.8230896 | GeneCards |
| GPRC6A | 0.8230896 | GeneCards |
| STARD13 | 0.8230896 | GeneCards |
| CD53 | 0.8230896 | GeneCards |
| MAML3 | 0.8230896 | GeneCards |
| DENND1A | 0.8230896 | GeneCards |
| ADGRL1 | 0.8230896 | GeneCards |
| SERBP1 | 0.8230896 | GeneCards |
| PTTG1IP | 0.8230896 | GeneCards |
| ADGRL3 | 0.8230896 | GeneCards |
| EFR3B | 0.8230896 | GeneCards |
| KCNQ1OT1 | 0.8230896 | GeneCards |
| CHORDC1P4 | 0.8230896 | GeneCards |
| LOC101060400 | 0.8230896 | GeneCards |
| ARSB | 0.820135951 | GeneCards |
| HSPA8 | 0.819875777 | GeneCards |
| TSPO | 0.818675697 | GeneCards |
| SOD3 | 0.818675697 | GeneCards |
| USP10 | 0.817543745 | GeneCards |
| IFNA14 | 0.817543745 | GeneCards |
| LCE3C | 0.817543745 | GeneCards |
| HCRTR1 | 0.811866283 | GeneCards |
| CDC5L | 0.811866283 | GeneCards |
| DYNC2H1 | 0.811866283 | GeneCards |
| CFHR2 | 0.811866283 | GeneCards |
| SDK2 | 0.811866283 | GeneCards |
| FAM189A1 | 0.811866283 | GeneCards |
| MIR30B | 0.811866283 | GeneCards |
| MIR181A1 | 0.811866283 | GeneCards |
| MIR574 | 0.811866283 | GeneCards |
| KLF6 | 0.811799467 | GeneCards |
| CD58 | 0.811799467 | GeneCards |
| ASB3 | 0.811799467 | GeneCards |
| SMARCA4 | 0.809443057 | GeneCards |
| SLC4A4 | 0.809443057 | GeneCards |
| MAP4K4 | 0.804990113 | GeneCards |
| MAP2K5 | 0.804990113 | GeneCards |
| POU5F1 | 0.804990113 | GeneCards |
| COL11A2 | 0.804990113 | GeneCards |
| TBL1XR1 | 0.804990113 | GeneCards |
| PFKFB3 | 0.804990113 | GeneCards |
| SKIV2L | 0.804990113 | GeneCards |
| AGPAT1 | 0.804990113 | GeneCards |
| RAB5B | 0.804990113 | GeneCards |
| BACH2 | 0.804990113 | GeneCards |
| RING1 | 0.804990113 | GeneCards |
| D2HGDH | 0.804990113 | GeneCards |
| LSM2 | 0.804990113 | GeneCards |
| MDC1 | 0.804990113 | GeneCards |
| DHX16 | 0.804990113 | GeneCards |
| ATG4B | 0.804990113 | GeneCards |
| MSH5 | 0.804990113 | GeneCards |
| SLC9A2 | 0.804990113 | GeneCards |
| PHF1 | 0.804990113 | GeneCards |
| RPS18 | 0.804990113 | GeneCards |
| ABCF1 | 0.804990113 | GeneCards |
| AAGAB | 0.804990113 | GeneCards |
| TIPIN | 0.804990113 | GeneCards |
| STK19 | 0.804990113 | GeneCards |
| SLC7A10 | 0.804990113 | GeneCards |
| ING5 | 0.804990113 | GeneCards |
| JAZF1 | 0.804990113 | GeneCards |
| GJA10 | 0.804990113 | GeneCards |
| PPP1R10 | 0.804990113 | GeneCards |
| CREG1 | 0.804990113 | GeneCards |
| MDN1 | 0.804990113 | GeneCards |
| UBAC2 | 0.804990113 | GeneCards |
| PPT2 | 0.804990113 | GeneCards |
| HLA-DQB2 | 0.804990113 | GeneCards |
| FNIP1 | 0.804990113 | GeneCards |
| TCF19 | 0.804990113 | GeneCards |
| NXPH4 | 0.804990113 | GeneCards |
| DPY19L3 | 0.804990113 | GeneCards |
| MBD6 | 0.804990113 | GeneCards |
| BAG6 | 0.804990113 | GeneCards |
| CEP89 | 0.804990113 | GeneCards |
| NABP2 | 0.804990113 | GeneCards |
| ZNF652 | 0.804990113 | GeneCards |
| VPS52 | 0.804990113 | GeneCards |
| SLC9A4 | 0.804990113 | GeneCards |
| ZWILCH | 0.804990113 | GeneCards |
| TPD52L3 | 0.804990113 | GeneCards |
| GAL3ST2 | 0.804990113 | GeneCards |
| EGFL8 | 0.804990113 | GeneCards |
| ZNF507 | 0.804990113 | GeneCards |
| THAP4 | 0.804990113 | GeneCards |
| NRM | 0.804990113 | GeneCards |
| RMI2 | 0.804990113 | GeneCards |
| IQCH | 0.804990113 | GeneCards |
| GPSM3 | 0.804990113 | GeneCards |
| GPANK1 | 0.804990113 | GeneCards |
| SKOR1 | 0.804990113 | GeneCards |
| ZCCHC10 | 0.804990113 | GeneCards |
| GPATCH1 | 0.804990113 | GeneCards |
| VWA7 | 0.804990113 | GeneCards |
| PSORS1C2 | 0.804990113 | GeneCards |
| C6orf47 | 0.804990113 | GeneCards |
| LY6G5B | 0.804990113 | GeneCards |
| HCP5 | 0.804990113 | GeneCards |
| KIAA2026 | 0.804990113 | GeneCards |
| LY6G5C | 0.804990113 | GeneCards |
| SFTA2 | 0.804990113 | GeneCards |
| MEIKIN | 0.804990113 | GeneCards |
| PSORS1C3 | 0.804990113 | GeneCards |
| HCG22 | 0.804990113 | GeneCards |
| MIR4435-2HG | 0.804990113 | GeneCards |
| GVQW3 | 0.804990113 | GeneCards |
| GATA3-AS1 | 0.804990113 | GeneCards |
| HLA-DRB6 | 0.804990113 | GeneCards |
| CYP21A1P | 0.804990113 | GeneCards |
| SNORD16 | 0.804990113 | GeneCards |
| IQCH-AS1 | 0.804990113 | GeneCards |
| HLA-DQB1-AS1 | 0.804990113 | GeneCards |
| MIR3936HG | 0.804990113 | GeneCards |
| TH2LCRR | 0.804990113 | GeneCards |
| LINC00709 | 0.804990113 | GeneCards |
| TBL1XR1-AS1 | 0.804990113 | GeneCards |
| MIR6891 | 0.804990113 | GeneCards |
| HNRNPCP4 | 0.804990113 | GeneCards |
| IL1R1-AS1 | 0.804990113 | GeneCards |
| LINC02863 | 0.804990113 | GeneCards |
| HLA-DPA3 | 0.804990113 | GeneCards |
| EMSY-DT | 0.804990113 | GeneCards |
| EIF2S2P3 | 0.804990113 | GeneCards |
| ENSG00000272501 | 0.804990113 | GeneCards |
| ENSG00000260271 | 0.804990113 | GeneCards |
| STK19B | 0.804990113 | GeneCards |
| RPL3P2 | 0.804990113 | GeneCards |
| HSPD1P4 | 0.804990113 | GeneCards |
| AK4P4 | 0.804990113 | GeneCards |
| LOC101928272 | 0.804990113 | GeneCards |
| ENSG00000250948 | 0.804990113 | GeneCards |
| ENSG00000259202 | 0.804990113 | GeneCards |
| ENSG00000262039 | 0.804990113 | GeneCards |
| ENSG00000234793 | 0.804990113 | GeneCards |
| ENSG00000202533 | 0.804990113 | GeneCards |
| RNU6-681P | 0.804990113 | GeneCards |
| MTCO3P1 | 0.804990113 | GeneCards |
| LOC107984360 | 0.804990113 | GeneCards |
| ENSG00000253238 | 0.804990113 | GeneCards |
| ENSG00000254810 | 0.804990113 | GeneCards |
| ENSG00000272221 | 0.804990113 | GeneCards |
| ENSG00000272540 | 0.804990113 | GeneCards |
| ENSG00000260773 | 0.804990113 | GeneCards |
| ENSG00000270212 | 0.804990113 | GeneCards |
| ENSG00000223808 | 0.804990113 | GeneCards |
| ENSG00000201207 | 0.804990113 | GeneCards |
| LOC105373514 | 0.804990113 | GeneCards |
| ENSG00000248428 | 0.804990113 | GeneCards |
| ENSG00000271581 | 0.804990113 | GeneCards |
| ENSG00000248648 | 0.804990113 | GeneCards |
| ENSG00000273160 | 0.804990113 | GeneCards |
| ENSG00000283782 | 0.804990113 | GeneCards |
| ENSG00000224431 | 0.804990113 | GeneCards |
| ENSG00000228739 | 0.804990113 | GeneCards |
| AC116366.1 | 0.804990113 | GeneCards |
| lnc-IQCH-7 | 0.804990113 | GeneCards |
| lnc-MAP3K7-3 | 0.804990113 | GeneCards |
| lnc-CD247-1 | 0.804990113 | GeneCards |
| lnc-HLA-C-2 | 0.804990113 | GeneCards |
| GTF3AP1 | 0.804990113 | GeneCards |
| ENSG00000254755 | 0.804990113 | GeneCards |
| ENSG00000287218 | 0.804990113 | GeneCards |
| lnc-NEMP1-2 | 0.804990113 | GeneCards |
| HSALNG0049429 | 0.804990113 | GeneCards |
| HSALNG0049258 | 0.804990113 | GeneCards |
| piR-33458 | 0.804990113 | GeneCards |
| piR-39701-054 | 0.804990113 | GeneCards |
| MG828730-053 | 0.804990113 | GeneCards |
| MK280269-056 | 0.804990113 | GeneCards |
| MN298114-196 | 0.804990113 | GeneCards |
| NONHSAG043472.2 | 0.804990113 | GeneCards |
| lnc-HLA-DQA1-9 | 0.804990113 | GeneCards |
| lnc-HLA-DRB1-7 | 0.804990113 | GeneCards |
| lnc-HLA-DRB1-8 | 0.804990113 | GeneCards |
| RF00017-4351 | 0.804990113 | GeneCards |
| MK280269-012 | 0.804990113 | GeneCards |
| piR-52740 | 0.804990113 | GeneCards |
| RF00017-3761 | 0.804990113 | GeneCards |
| lnc-GATA3-19-001 | 0.804990113 | GeneCards |
| lnc-GATA3-20 | 0.804990113 | GeneCards |
| lnc-KIN-10 | 0.804990113 | GeneCards |
| piR-47864 | 0.804990113 | GeneCards |
| piR-50208 | 0.804990113 | GeneCards |
| RF00017-2646 | 0.804990113 | GeneCards |
| piR-59241 | 0.804990113 | GeneCards |
| HSALNG0044846 | 0.804990113 | GeneCards |
| HSALNG0044847 | 0.804990113 | GeneCards |
| HSALNG0049423 | 0.804990113 | GeneCards |
| HSALNG0049424 | 0.804990113 | GeneCards |
| HSALNG0049427 | 0.804990113 | GeneCards |
| HSALNG0049428 | 0.804990113 | GeneCards |
| HSALNG0106744 | 0.804990113 | GeneCards |
| MN298214 | 0.804990113 | GeneCards |
| piR-30396 | 0.804990113 | GeneCards |
| HSALNG0075913 | 0.804990113 | GeneCards |
| NONHSAG017238.2 | 0.804990113 | GeneCards |
| lnc-IQCH-5 | 0.804990113 | GeneCards |
| RF00017-4938 | 0.804990113 | GeneCards |
| RF00017-6843 | 0.804990113 | GeneCards |
| lnc-GATA3-19-002 | 0.804990113 | GeneCards |
| NONHSAG043568.2 | 0.804990113 | GeneCards |
| piR-47234 | 0.804990113 | GeneCards |
| piR-49732-033 | 0.804990113 | GeneCards |
| ENSG00000250728 | 0.804990113 | GeneCards |
| ENSG00000270779 | 0.804990113 | GeneCards |
| HSALNG0044120 | 0.804990113 | GeneCards |
| HSALNG0044122 | 0.804990113 | GeneCards |
| HSALNG0049431 | 0.804990113 | GeneCards |
| HSALNG0023719 | 0.804990113 | GeneCards |
| HSALNG0023718 | 0.804990113 | GeneCards |
| piR-50308-096 | 0.804990113 | GeneCards |
| RF00026-726 | 0.804990113 | GeneCards |
| EDN2 | 0.803851902 | GeneCards |
| CCK | 0.802752733 | GeneCards |
| OVOL1 | 0.798181951 | GeneCards |
| NOTCH3 | 0.797982514 | GeneCards |
| CNR1 | 0.797982514 | GeneCards |
| ACVR1B | 0.797982514 | GeneCards |
| CTSL | 0.797982514 | GeneCards |
| CHRNA1 | 0.797982514 | GeneCards |
| THBS1 | 0.797982514 | GeneCards |
| SLC44A1 | 0.797982514 | GeneCards |
| SOAT1 | 0.797982514 | GeneCards |
| DOK1 | 0.797982514 | GeneCards |
| MID1 | 0.797982514 | GeneCards |
| GSTA2 | 0.797982514 | GeneCards |
| UNC119 | 0.797982514 | GeneCards |
| TFF2 | 0.797982514 | GeneCards |
| RGS5 | 0.797982514 | GeneCards |
| PITRM1 | 0.797982514 | GeneCards |
| FASTK | 0.797982514 | GeneCards |
| MRPL42 | 0.797982514 | GeneCards |
| OXER1 | 0.797982514 | GeneCards |
| NLRP4 | 0.797982514 | GeneCards |
| NFATC2IP | 0.797982514 | GeneCards |
| SEC14L3 | 0.797982514 | GeneCards |
| CYYR1 | 0.797982514 | GeneCards |
| NIBAN1 | 0.797982514 | GeneCards |
| MIRLET7C | 0.797982514 | GeneCards |
| MIR323A | 0.797982514 | GeneCards |
| MIR885 | 0.797982514 | GeneCards |
| ADH1C | 0.797104955 | GeneCards |
| TPH1 | 0.796555877 | GeneCards |
| MIR143 | 0.796555877 | GeneCards |
| FADD | 0.7951895 | GeneCards |
| ITGB8 | 0.790842772 | GeneCards |
| TPD52 | 0.790842772 | GeneCards |
| RNF144A | 0.790842772 | GeneCards |
| ARRDC1 | 0.790842772 | GeneCards |
| HCG26 | 0.790842772 | GeneCards |
| HLA-DRB9 | 0.790842772 | GeneCards |
| PVALB | 0.789679706 | GeneCards |
| ZNF432 | 0.789679706 | GeneCards |
| ZNF614 | 0.789679706 | GeneCards |
| ZNF841 | 0.789679706 | GeneCards |
| PHYH | 0.789512038 | GeneCards |
| CASP14 | 0.789512038 | GeneCards |
| AVPR1B | 0.789512038 | GeneCards |
| U2AF1 | 0.789512038 | GeneCards |
| KRT10 | 0.789512038 | GeneCards |
| ALOXE3 | 0.789512038 | GeneCards |
| ALOX12B | 0.789512038 | GeneCards |
| ABCA12 | 0.789512038 | GeneCards |
| PDCL | 0.789512038 | GeneCards |
| GHRH | 0.789512038 | GeneCards |
| FKBP14 | 0.789512038 | GeneCards |
| TMEM67 | 0.789512038 | GeneCards |
| PNPLA1 | 0.789512038 | GeneCards |
| ASPRV1 | 0.789512038 | GeneCards |
| SRY | 0.789512038 | GeneCards |
| MT-ATP6 | 0.789512038 | GeneCards |
| MIR200A | 0.789512038 | GeneCards |
| MIR17 | 0.789512038 | GeneCards |
| MIR210 | 0.789512038 | GeneCards |
| MIR196A1 | 0.789512038 | GeneCards |
| MIR24-1 | 0.789512038 | GeneCards |
| MIR15B | 0.789512038 | GeneCards |
| MIR423 | 0.789512038 | GeneCards |
| FRAXA | 0.789512038 | GeneCards |
| ABCG2 | 0.785743892 | GeneCards |
| CXCL11 | 0.785607815 | GeneCards |
| SMARCA2 | 0.782635868 | GeneCards |
| KAT2A | 0.782635868 | GeneCards |
| ALDH1A2 | 0.782635868 | GeneCards |
| NRXN1 | 0.782635868 | GeneCards |
| KAT5 | 0.782635868 | GeneCards |
| PSMA6 | 0.782635868 | GeneCards |
| HAND2 | 0.782635868 | GeneCards |
| GALNS | 0.782635868 | GeneCards |
| HERC2 | 0.782635868 | GeneCards |
| LNX1 | 0.782635868 | GeneCards |
| PSMD2 | 0.782635868 | GeneCards |
| XRCC4 | 0.782635868 | GeneCards |
| RNF5 | 0.782635868 | GeneCards |
| CHIC2 | 0.782635868 | GeneCards |
| UBXN11 | 0.782635868 | GeneCards |
| DESI2 | 0.782635868 | GeneCards |
| TMEM216 | 0.782635868 | GeneCards |
| H4C8 | 0.782635868 | GeneCards |
| H4C11 | 0.782635868 | GeneCards |
| H4C12 | 0.782635868 | GeneCards |
| H4C13 | 0.782635868 | GeneCards |
| MSR1 | 0.778721273 | GeneCards |
| SCT | 0.774070263 | GeneCards |
| FBLN5 | 0.771448851 | GeneCards |
| MIR139 | 0.771448851 | GeneCards |
| MAP2K1 | 0.768200874 | GeneCards |
| GNAS | 0.768200874 | GeneCards |
| CTSB | 0.768200874 | GeneCards |
| BMPR1A | 0.768200874 | GeneCards |
| GABBR2 | 0.768200874 | GeneCards |
| LAMA1 | 0.768200874 | GeneCards |
| OPRL1 | 0.768200874 | GeneCards |
| MKNK1 | 0.768200874 | GeneCards |
| KCNA5 | 0.768200874 | GeneCards |
| DOCK2 | 0.768200874 | GeneCards |
| TFAM | 0.768200874 | GeneCards |
| TAT | 0.768200874 | GeneCards |
| SIX1 | 0.768200874 | GeneCards |
| INHA | 0.768200874 | GeneCards |
| ADCY2 | 0.768200874 | GeneCards |
| BSG | 0.768200874 | GeneCards |
| STK10 | 0.768200874 | GeneCards |
| PIP5K1B | 0.768200874 | GeneCards |
| NRF1 | 0.768200874 | GeneCards |
| BST1 | 0.768200874 | GeneCards |
| FGFRL1 | 0.768200874 | GeneCards |
| AGTPBP1 | 0.768200874 | GeneCards |
| MR1 | 0.768200874 | GeneCards |
| MT2A | 0.768200874 | GeneCards |
| PKP1 | 0.768200874 | GeneCards |
| TAGLN2 | 0.768200874 | GeneCards |
| TNNI1 | 0.768200874 | GeneCards |
| IL1F10 | 0.768200874 | GeneCards |
| GLIS3 | 0.768200874 | GeneCards |
| ZMYND8 | 0.768200874 | GeneCards |
| RPH3AL | 0.768200874 | GeneCards |
| C1QTNF3 | 0.768200874 | GeneCards |
| CEP68 | 0.768200874 | GeneCards |
| PNOC | 0.768200874 | GeneCards |
| SIPA1L2 | 0.768200874 | GeneCards |
| CNIH3 | 0.768200874 | GeneCards |
| SKA1 | 0.768200874 | GeneCards |
| HS3ST4 | 0.768200874 | GeneCards |
| FSCB | 0.768200874 | GeneCards |
| ZNF248 | 0.768200874 | GeneCards |
| CWC22 | 0.768200874 | GeneCards |
| TMCO4 | 0.768200874 | GeneCards |
| TTC17 | 0.768200874 | GeneCards |
| MIR204 | 0.768200874 | GeneCards |
| RPL13AP5 | 0.768200874 | GeneCards |
| RPS10P7 | 0.768200874 | GeneCards |
| GLCCI1-DT | 0.768200874 | GeneCards |
| RPS15AP30 | 0.768200874 | GeneCards |
| TMED10P2 | 0.768200874 | GeneCards |
| SETDB2-PHF11 | 0.768200874 | GeneCards |
| RPL21P126 | 0.768200874 | GeneCards |
| ISCA1P2 | 0.768200874 | GeneCards |
| RPS15AP27 | 0.768200874 | GeneCards |
| DEPDC1P2 | 0.768200874 | GeneCards |
| RPS15P8 | 0.768200874 | GeneCards |
| RPL7P37 | 0.768200874 | GeneCards |
| RPL12P40 | 0.768200874 | GeneCards |
| RNU7-17P | 0.768200874 | GeneCards |
| RPL26P25 | 0.768200874 | GeneCards |
| RPS27P27 | 0.768200874 | GeneCards |
| RPL7AP13 | 0.768200874 | GeneCards |
| HSPG2 | 0.767325461 | GeneCards |
| SLC26A9 | 0.767325461 | GeneCards |
| MIR20B | 0.767325461 | GeneCards |
| THRB | 0.76457262 | GeneCards |
| DPP6 | 0.76457262 | GeneCards |
| COL22A1 | 0.76457262 | GeneCards |
| ERCC6 | 0.760281622 | GeneCards |
| CD2 | 0.760281622 | GeneCards |
| FIP1L1 | 0.760281622 | GeneCards |
| CCL28 | 0.757498741 | GeneCards |
| DCN | 0.754392266 | GeneCards |
| SRF | 0.74303633 | GeneCards |
| TSC2 | 0.741667211 | GeneCards |
| TSC1 | 0.741667211 | GeneCards |
| ABCC2 | 0.735258102 | GeneCards |
| ADRA2B | 0.735258102 | GeneCards |
| P2RY1 | 0.735258102 | GeneCards |
| MAOB | 0.735258102 | GeneCards |
| PLEK | 0.735258102 | GeneCards |
| SLC8A1 | 0.734790981 | GeneCards |
| TAPBP | 0.734790981 | GeneCards |
| DUSP4 | 0.734790981 | GeneCards |
| SLC30A1 | 0.734790981 | GeneCards |
| OBSCN | 0.734790981 | GeneCards |
| PPARGC1B | 0.734790981 | GeneCards |
| MUCL3 | 0.734790981 | GeneCards |
| TNFRSF11B | 0.731508613 | GeneCards |
| SOX2 | 0.729388714 | GeneCards |
| ACP5 | 0.729388714 | GeneCards |
| PDK1 | 0.729388714 | GeneCards |
| SNAI1 | 0.729388714 | GeneCards |
| CYP4F3 | 0.729388714 | GeneCards |
| ATP8A1 | 0.729388714 | GeneCards |
| PPP1R12B | 0.729388714 | GeneCards |
| SERPINA7 | 0.729388714 | GeneCards |
| TMEM196 | 0.729388714 | GeneCards |
| HSD11B1 | 0.718440592 | GeneCards |
| PROS1 | 0.718440592 | GeneCards |
| FABP1 | 0.718440592 | GeneCards |
| HAO1 | 0.716649413 | GeneCards |
| PACRG | 0.716649413 | GeneCards |
| DYNLT1 | 0.716649413 | GeneCards |
| KIR3DL3 | 0.716649413 | GeneCards |
| IDH2 | 0.712436736 | GeneCards |
| MYH9 | 0.712436736 | GeneCards |
| GSN | 0.712436736 | GeneCards |
| HDAC8 | 0.712436736 | GeneCards |
| TACR3 | 0.711754203 | GeneCards |
| GABBR1 | 0.71145153 | GeneCards |
| FOXJ1 | 0.71145153 | GeneCards |
| LIF | 0.710336447 | GeneCards |
| ISG15 | 0.709051847 | GeneCards |
| XPO1 | 0.709051847 | GeneCards |
| ITCH | 0.709051847 | GeneCards |
| RANBP2 | 0.709051847 | GeneCards |
| PML | 0.709051847 | GeneCards |
| EIF2S1 | 0.709051847 | GeneCards |
| PABPN1 | 0.709051847 | GeneCards |
| TMPRSS2 | 0.709051847 | GeneCards |
| TRIM25 | 0.709051847 | GeneCards |
| SLC25A6 | 0.709051847 | GeneCards |
| NUP98 | 0.709051847 | GeneCards |
| KPNA3 | 0.709051847 | GeneCards |
| NXF1 | 0.709051847 | GeneCards |
| DNAJC3 | 0.709051847 | GeneCards |
| NLRX1 | 0.709051847 | GeneCards |
| RSAD2 | 0.709051847 | GeneCards |
| CPSF4 | 0.709051847 | GeneCards |
| IVNS1ABP | 0.709051847 | GeneCards |
| CALCOCO2 | 0.709051847 | GeneCards |
| IK | 0.709051847 | GeneCards |
| KPNA5 | 0.709051847 | GeneCards |
| ZBP1 | 0.709051847 | GeneCards |
| MX2 | 0.709051847 | GeneCards |
| CHD6 | 0.709051847 | GeneCards |
| STAU1 | 0.709051847 | GeneCards |
| MORC3 | 0.709051847 | GeneCards |
| SMU1 | 0.709051847 | GeneCards |
| PAF1 | 0.709051847 | GeneCards |
| FAM76B | 0.709051847 | GeneCards |
| RRP1B | 0.709051847 | GeneCards |
| FAM81B | 0.709051847 | GeneCards |
| TTC38 | 0.709051847 | GeneCards |
| OR4D10 | 0.709051847 | GeneCards |
| FAM216A | 0.709051847 | GeneCards |
| RTRAF | 0.709051847 | GeneCards |
| WDR87 | 0.709051847 | GeneCards |
| OR8K3 | 0.709051847 | GeneCards |
| PRKN | 0.702854991 | GeneCards |
| HLA-DRB4 | 0.702064395 | GeneCards |
| HLA-DRB3 | 0.702064395 | GeneCards |
| LOC102723407 | 0.702064395 | GeneCards |
| TNFRSF11A | 0.701727033 | GeneCards |
| FADS1 | 0.701727033 | GeneCards |
| SLC39A7 | 0.701727033 | GeneCards |
| CRNN | 0.701727033 | GeneCards |
| DNAJC14 | 0.701727033 | GeneCards |
| SFRP1 | 0.701578736 | GeneCards |
| RARA | 0.697141886 | GeneCards |
| MAP3K11 | 0.697141886 | GeneCards |
| GLDC | 0.697141886 | GeneCards |
| NCSTN | 0.697141886 | GeneCards |
| DDX6 | 0.697141886 | GeneCards |
| ACADS | 0.697141886 | GeneCards |
| RFC1 | 0.697141886 | GeneCards |
| HNF1A | 0.697141886 | GeneCards |
| EHMT2 | 0.697141886 | GeneCards |
| BCL2L11 | 0.697141886 | GeneCards |
| SPRY4 | 0.697141886 | GeneCards |
| PIP5K1A | 0.697141886 | GeneCards |
| SDHC | 0.697141886 | GeneCards |
| NDUFS2 | 0.697141886 | GeneCards |
| KMT2A | 0.697141886 | GeneCards |
| APOA2 | 0.697141886 | GeneCards |
| DAXX | 0.697141886 | GeneCards |
| LGR5 | 0.697141886 | GeneCards |
| ZFP36L1 | 0.697141886 | GeneCards |
| PTPRK | 0.697141886 | GeneCards |
| IGFBP4 | 0.697141886 | GeneCards |
| ITPKA | 0.697141886 | GeneCards |
| DDAH2 | 0.697141886 | GeneCards |
| ADAMTS4 | 0.697141886 | GeneCards |
| TREH | 0.697141886 | GeneCards |
| VTI1B | 0.697141886 | GeneCards |
| SETDB1 | 0.697141886 | GeneCards |
| NEUROD2 | 0.697141886 | GeneCards |
| CLIC1 | 0.697141886 | GeneCards |
| CELF2 | 0.697141886 | GeneCards |
| UBE2Z | 0.697141886 | GeneCards |
| VPS11 | 0.697141886 | GeneCards |
| RFX5 | 0.697141886 | GeneCards |
| RERE | 0.697141886 | GeneCards |
| NDUFAF1 | 0.697141886 | GeneCards |
| MED1 | 0.697141886 | GeneCards |
| COQ5 | 0.697141886 | GeneCards |
| HSD17B8 | 0.697141886 | GeneCards |
| GNPDA1 | 0.697141886 | GeneCards |
| CRTAP | 0.697141886 | GeneCards |
| ANAPC1 | 0.697141886 | GeneCards |
| B3GALT4 | 0.697141886 | GeneCards |
| CDK12 | 0.697141886 | GeneCards |
| TAX1BP1 | 0.697141886 | GeneCards |
| SYVN1 | 0.697141886 | GeneCards |
| SLC44A4 | 0.697141886 | GeneCards |
| SIPA1 | 0.697141886 | GeneCards |
| SERPINB7 | 0.697141886 | GeneCards |
| APOM | 0.697141886 | GeneCards |
| B4GALT3 | 0.697141886 | GeneCards |
| KLF3 | 0.697141886 | GeneCards |
| GPR18 | 0.697141886 | GeneCards |
| AP1S3 | 0.697141886 | GeneCards |
| CATSPER1 | 0.697141886 | GeneCards |
| TUFT1 | 0.697141886 | GeneCards |
| UHRF2 | 0.697141886 | GeneCards |
| TSPAN8 | 0.697141886 | GeneCards |
| TDRKH | 0.697141886 | GeneCards |
| THEM4 | 0.697141886 | GeneCards |
| TNFSF18 | 0.697141886 | GeneCards |
| MRPL44 | 0.697141886 | GeneCards |
| PTPRR | 0.697141886 | GeneCards |
| NCR3 | 0.697141886 | GeneCards |
| GPA33 | 0.697141886 | GeneCards |
| ATP6V1G2 | 0.697141886 | GeneCards |
| CTSW | 0.697141886 | GeneCards |
| SNX27 | 0.697141886 | GeneCards |
| TEF | 0.697141886 | GeneCards |
| RAD51B | 0.697141886 | GeneCards |
| ILDR1 | 0.697141886 | GeneCards |
| LRP3 | 0.697141886 | GeneCards |
| CGN | 0.697141886 | GeneCards |
| TRIM39 | 0.697141886 | GeneCards |
| TM9SF2 | 0.697141886 | GeneCards |
| ZNF687 | 0.697141886 | GeneCards |
| THEMIS | 0.697141886 | GeneCards |
| SNX32 | 0.697141886 | GeneCards |
| STARD4 | 0.697141886 | GeneCards |
| MRPL11 | 0.697141886 | GeneCards |
| PHOSPHO1 | 0.697141886 | GeneCards |
| RPS25 | 0.697141886 | GeneCards |
| FKBPL | 0.697141886 | GeneCards |
| CASC3 | 0.697141886 | GeneCards |
| DUSP12 | 0.697141886 | GeneCards |
| VARS1 | 0.697141886 | GeneCards |
| UPK2 | 0.697141886 | GeneCards |
| RTF1 | 0.697141886 | GeneCards |
| RBM14 | 0.697141886 | GeneCards |
| H2AX | 0.697141886 | GeneCards |
| KDM8 | 0.697141886 | GeneCards |
| LST1 | 0.697141886 | GeneCards |
| DEDD | 0.697141886 | GeneCards |
| CELF3 | 0.697141886 | GeneCards |
| SPPL3 | 0.697141886 | GeneCards |
| WDFY1 | 0.697141886 | GeneCards |
| WIPF2 | 0.697141886 | GeneCards |
| THEM5 | 0.697141886 | GeneCards |
| RCSD1 | 0.697141886 | GeneCards |
| KRT28 | 0.697141886 | GeneCards |
| BCL9L | 0.697141886 | GeneCards |
| MRPL41 | 0.697141886 | GeneCards |
| MRPL9 | 0.697141886 | GeneCards |
| NELFE | 0.697141886 | GeneCards |
| DXO | 0.697141886 | GeneCards |
| TCHHL1 | 0.697141886 | GeneCards |
| MSL1 | 0.697141886 | GeneCards |
| TOMM40L | 0.697141886 | GeneCards |
| ZFPL1 | 0.697141886 | GeneCards |
| TESPA1 | 0.697141886 | GeneCards |
| TPRG1 | 0.697141886 | GeneCards |
| ZMYND19 | 0.697141886 | GeneCards |
| ICE2 | 0.697141886 | GeneCards |
| ATAT1 | 0.697141886 | GeneCards |
| OAZ3 | 0.697141886 | GeneCards |
| RAPGEFL1 | 0.697141886 | GeneCards |
| EHBP1L1 | 0.697141886 | GeneCards |
| MUCL1 | 0.697141886 | GeneCards |
| PCDHB7 | 0.697141886 | GeneCards |
| KRT222 | 0.697141886 | GeneCards |
| C1orf56 | 0.697141886 | GeneCards |
| ATP5MG | 0.697141886 | GeneCards |
| LINGO4 | 0.697141886 | GeneCards |
| ZNF704 | 0.697141886 | GeneCards |
| KRTAP3-2 | 0.697141886 | GeneCards |
| MCCD1 | 0.697141886 | GeneCards |
| TMPPE | 0.697141886 | GeneCards |
| TSGA10IP | 0.697141886 | GeneCards |
| THAP12 | 0.697141886 | GeneCards |
| SEPTIN8 | 0.697141886 | GeneCards |
| KRTAP2-1 | 0.697141886 | GeneCards |
| CCNI2 | 0.697141886 | GeneCards |
| TMEM232 | 0.697141886 | GeneCards |
| CFAP126 | 0.697141886 | GeneCards |
| OTULINL | 0.697141886 | GeneCards |
| SPATA32 | 0.697141886 | GeneCards |
| SAPCD1 | 0.697141886 | GeneCards |
| KRTAP2-2 | 0.697141886 | GeneCards |
| LCE1D | 0.697141886 | GeneCards |
| LCE5A | 0.697141886 | GeneCards |
| CENATAC | 0.697141886 | GeneCards |
| LCE1C | 0.697141886 | GeneCards |
| HNF1A-AS1 | 0.697141886 | GeneCards |
| OIP5-AS1 | 0.697141886 | GeneCards |
| SFTA1P | 0.697141886 | GeneCards |
| SNORD117 | 0.697141886 | GeneCards |
| IL21-AS1 | 0.697141886 | GeneCards |
| LINC00298 | 0.697141886 | GeneCards |
| JAZF1-AS1 | 0.697141886 | GeneCards |
| MAP3K14-AS1 | 0.697141886 | GeneCards |
| MIR554 | 0.697141886 | GeneCards |
| HCG24 | 0.697141886 | GeneCards |
| HCG25 | 0.697141886 | GeneCards |
| FLJ40194 | 0.697141886 | GeneCards |
| PRKCQ-AS1 | 0.697141886 | GeneCards |
| MIR4464 | 0.697141886 | GeneCards |
| ID2-AS1 | 0.697141886 | GeneCards |
| DDX39B-AS1 | 0.697141886 | GeneCards |
| OVOL1-AS1 | 0.697141886 | GeneCards |
| SNORD124 | 0.697141886 | GeneCards |
| LINC01527 | 0.697141886 | GeneCards |
| LRP1-AS | 0.697141886 | GeneCards |
| MIR623 | 0.697141886 | GeneCards |
| LOC100287049 | 0.697141886 | GeneCards |
| RPS3AP21 | 0.697141886 | GeneCards |
| KRT8P26 | 0.697141886 | GeneCards |
| LINC02648 | 0.697141886 | GeneCards |
| TSBP1-AS1 | 0.697141886 | GeneCards |
| UQCRHP1 | 0.697141886 | GeneCards |
| TDRKH-AS1 | 0.697141886 | GeneCards |
| RFX5-AS1 | 0.697141886 | GeneCards |
| LINC02676 | 0.697141886 | GeneCards |
| AKR1D1P1 | 0.697141886 | GeneCards |
| ENSG00000285016 | 0.697141886 | GeneCards |
| ENSG00000248714 | 0.697141886 | GeneCards |
| ENSG00000265799 | 0.697141886 | GeneCards |
| ENSG00000254855 | 0.697141886 | GeneCards |
| ENSG00000258053 | 0.697141886 | GeneCards |
| ENSG00000249318 | 0.697141886 | GeneCards |
| ENSG00000255038 | 0.697141886 | GeneCards |
| ENSG00000224228 | 0.697141886 | GeneCards |
| ENSG00000232937 | 0.697141886 | GeneCards |
| ENSG00000226375 | 0.697141886 | GeneCards |
| LOC102724596 | 0.697141886 | GeneCards |
| PPIAP6 | 0.697141886 | GeneCards |
| RPL32P1 | 0.697141886 | GeneCards |
| RPS10P6 | 0.697141886 | GeneCards |
| PPIAP9 | 0.697141886 | GeneCards |
| RPL15P4 | 0.697141886 | GeneCards |
| PTPRK-AS1 | 0.697141886 | GeneCards |
| SOCAR | 0.697141886 | GeneCards |
| LINC02761 | 0.697141886 | GeneCards |
| LOC102725019 | 0.697141886 | GeneCards |
| ENSG00000250186 | 0.697141886 | GeneCards |
| ENSG00000257870 | 0.697141886 | GeneCards |
| ENSG00000269621 | 0.697141886 | GeneCards |
| ENSG00000248969 | 0.697141886 | GeneCards |
| ENSG00000263033 | 0.697141886 | GeneCards |
| ENSG00000252840 | 0.697141886 | GeneCards |
| ENSG00000224645 | 0.697141886 | GeneCards |
| ENSG00000230612 | 0.697141886 | GeneCards |
| ENSG00000233411 | 0.697141886 | GeneCards |
| MIR5708 | 0.697141886 | GeneCards |
| RPL12P33 | 0.697141886 | GeneCards |
| RN7SL688P | 0.697141886 | GeneCards |
| RPL29P24 | 0.697141886 | GeneCards |
| RNU6-1213P | 0.697141886 | GeneCards |
| KRT8P37 | 0.697141886 | GeneCards |
| EEF1A1P27 | 0.697141886 | GeneCards |
| RNA5SP158 | 0.697141886 | GeneCards |
| ENSG00000266208 | 0.697141886 | GeneCards |
| ENSG00000264968 | 0.697141886 | GeneCards |
| ENSG00000206649 | 0.697141886 | GeneCards |
| ENSG00000207127 | 0.697141886 | GeneCards |
| ENSG00000197670 | 0.697141886 | GeneCards |
| ENSG00000236427 | 0.697141886 | GeneCards |
| RNU6-979P | 0.697141886 | GeneCards |
| ENSG00000286239 | 0.697141886 | GeneCards |
| lnc-NCR3-1 | 0.697141886 | GeneCards |
| ENSG00000245869 | 0.697141886 | GeneCards |
| lnc-PRKCQ-3 | 0.697141886 | GeneCards |
| FAM183DP | 0.697141886 | GeneCards |
| ENSG00000279775 | 0.697141886 | GeneCards |
| ENSG00000283411 | 0.697141886 | GeneCards |
| HSALNG0007916 | 0.697141886 | GeneCards |
| lnc-ICE2-7 | 0.697141886 | GeneCards |
| lnc-PTPRR-3 | 0.697141886 | GeneCards |
| lnc-TNF-1 | 0.697141886 | GeneCards |
| lnc-TNFSF18-4 | 0.697141886 | GeneCards |
| HSALNG0049290 | 0.697141886 | GeneCards |
| HSALNG0049291 | 0.697141886 | GeneCards |
| HSALNG0049430 | 0.697141886 | GeneCards |
| AB372574 | 0.697141886 | GeneCards |
| AB675034 | 0.697141886 | GeneCards |
| lnc-ZMYND19-2 | 0.697141886 | GeneCards |
| HSALNG0007192-001 | 0.697141886 | GeneCards |
| HSALNG0007192-002 | 0.697141886 | GeneCards |
| HSALNG0012816 | 0.697141886 | GeneCards |
| HSALNG0008504 | 0.697141886 | GeneCards |
| HSALNG0022604 | 0.697141886 | GeneCards |
| HSALNG0092336-001 | 0.697141886 | GeneCards |
| HSALNG0122068-001 | 0.697141886 | GeneCards |
| JA662189 | 0.697141886 | GeneCards |
| MK280492 | 0.697141886 | GeneCards |
| HSALNG0091393-001 | 0.697141886 | GeneCards |
| HSALNG0091400 | 0.697141886 | GeneCards |
| NONHSAG018964.2 | 0.697141886 | GeneCards |
| NONHSAG043493.2 | 0.697141886 | GeneCards |
| NONHSAG045982.2 | 0.697141886 | GeneCards |
| piR-33804-057 | 0.697141886 | GeneCards |
| lnc-HLA-DQB1-2 | 0.697141886 | GeneCards |
| lnc-HLA-DQA1-8 | 0.697141886 | GeneCards |
| lnc-HLA-DRB1-6 | 0.697141886 | GeneCards |
| lnc-SMARCE1-1 | 0.697141886 | GeneCards |
| lnc-SMARCE1-2 | 0.697141886 | GeneCards |
| lnc-SMARCE1-4 | 0.697141886 | GeneCards |
| lnc-THAP12-4 | 0.697141886 | GeneCards |
| RF00017-4493 | 0.697141886 | GeneCards |
| HSALNG0087578 | 0.697141886 | GeneCards |
| lnc-NDFIP1-1 | 0.697141886 | GeneCards |
| lnc-RAB5B-2 | 0.697141886 | GeneCards |
| piR-32023-098 | 0.697141886 | GeneCards |
| piR-33432-124 | 0.697141886 | GeneCards |
| piR-35516 | 0.697141886 | GeneCards |
| piR-36419 | 0.697141886 | GeneCards |
| RF00017-4926 | 0.697141886 | GeneCards |
| RF00026-955 | 0.697141886 | GeneCards |
| HSALNG0105356-001 | 0.697141886 | GeneCards |
| lnc-APOA2-2 | 0.697141886 | GeneCards |
| NONHSAG041785.2 | 0.697141886 | GeneCards |
| piR-38351-029 | 0.697141886 | GeneCards |
| piR-43107-023 | 0.697141886 | GeneCards |
| piR-44329 | 0.697141886 | GeneCards |
| RF00017-304 | 0.697141886 | GeneCards |
| ENSG00000270234 | 0.697141886 | GeneCards |
| LOC105377891 | 0.697141886 | GeneCards |
| LOC107986589 | 0.697141886 | GeneCards |
| HE856132 | 0.697141886 | GeneCards |
| HSALNG0008232 | 0.697141886 | GeneCards |
| HSALNG0044839 | 0.697141886 | GeneCards |
| HSALNG0017403 | 0.697141886 | GeneCards |
| HSALNG0117177 | 0.697141886 | GeneCards |
| HSALNG0117178 | 0.697141886 | GeneCards |
| piR-51710 | 0.697141886 | GeneCards |
| HSALNG0075965 | 0.697141886 | GeneCards |
| HSALNG0087584 | 0.697141886 | GeneCards |
| HSALNG0091473 | 0.697141886 | GeneCards |
| HSALNG0091590 | 0.697141886 | GeneCards |
| piR-38344-081 | 0.697141886 | GeneCards |
| piR-61289-077 | 0.697141886 | GeneCards |
| HSALNG0087581 | 0.697141886 | GeneCards |
| HSALNG0102088 | 0.697141886 | GeneCards |
| lnc-IL4R-4 | 0.697141886 | GeneCards |
| piR-50444-270 | 0.697141886 | GeneCards |
| RF00017-1441 | 0.697141886 | GeneCards |
| HSALNG0075447 | 0.697141886 | GeneCards |
| HSALNG0075448 | 0.697141886 | GeneCards |
| HSALNG0091474 | 0.697141886 | GeneCards |
| HSALNG0094551 | 0.697141886 | GeneCards |
| HSALNG0094555 | 0.697141886 | GeneCards |
| HSALNG0106356 | 0.697141886 | GeneCards |
| HSALNG0109575 | 0.697141886 | GeneCards |
| HSALNG0109576 | 0.697141886 | GeneCards |
| HSALNG0116381 | 0.697141886 | GeneCards |
| piR-53029-049 | 0.697141886 | GeneCards |
| RF00017-5175 | 0.697141886 | GeneCards |
| RF00017-6582 | 0.697141886 | GeneCards |
| RF00017-952 | 0.697141886 | GeneCards |
| LOC105375208 | 0.697141886 | GeneCards |
| LOC101928512 | 0.697141886 | GeneCards |
| ENSG00000280047 | 0.697141886 | GeneCards |
| HSALNG0007917 | 0.697141886 | GeneCards |
| HSALNG0007918 | 0.697141886 | GeneCards |
| LOC107986073 | 0.697141886 | GeneCards |
| HSALNG0023716 | 0.697141886 | GeneCards |
| HSALNG0025039 | 0.697141886 | GeneCards |
| HSALNG0056490-001 | 0.697141886 | GeneCards |
| HSALNG0017407 | 0.697141886 | GeneCards |
| HSALNG0056490-002 | 0.697141886 | GeneCards |
| HSALNG0116307 | 0.697141886 | GeneCards |
| NONHSAG045795.2 | 0.697141886 | GeneCards |
| piR-48772-016 | 0.697141886 | GeneCards |
| lnc-FLG2-1 | 0.697141886 | GeneCards |
| lnc-MLANA-3 | 0.697141886 | GeneCards |
| RF00017-4499 | 0.697141886 | GeneCards |
| RF00026-1042 | 0.697141886 | GeneCards |
| lnc-LINGO4-1 | 0.697141886 | GeneCards |
| piR-48209-689 | 0.697141886 | GeneCards |
| RF00917 | 0.697141886 | GeneCards |
| HSALNG0066497 | 0.697141886 | GeneCards |
| HSALNG0066498 | 0.697141886 | GeneCards |
| MN298065 | 0.697141886 | GeneCards |
| MN309005 | 0.697141886 | GeneCards |
| piR-41838-002 | 0.697141886 | GeneCards |
| RF00017-6585 | 0.697141886 | GeneCards |
| RF00017-6587 | 0.697141886 | GeneCards |
| HSALNG0076093 | 0.697141886 | GeneCards |
| piR-37972-677 | 0.697141886 | GeneCards |
| HSALNG0076086 | 0.697141886 | GeneCards |
| piR-41525-047 | 0.697141886 | GeneCards |
| RYR1 | 0.69597882 | GeneCards |
| KL | 0.69597882 | GeneCards |
| ELK3 | 0.69597882 | GeneCards |
| ATRN | 0.69597882 | GeneCards |
| ALLC | 0.69597882 | GeneCards |
| KIAA0825 | 0.69597882 | GeneCards |
| TNXA | 0.69597882 | GeneCards |
| OR7E156P | 0.69597882 | GeneCards |
| PTGES3 | 0.685030699 | GeneCards |
| LGALS7 | 0.680074871 | GeneCards |
| CD34 | 0.673624575 | GeneCards |
| CLCN5 | 0.673624575 | GeneCards |
| IL3RA | 0.673624575 | GeneCards |
| PSMC4 | 0.673624575 | GeneCards |
| ACTA2 | 0.67263931 | GeneCards |
| ABCC8 | 0.67263931 | GeneCards |
| COL18A1 | 0.670623481 | GeneCards |
| NCF4 | 0.665829778 | GeneCards |
| MYC | 0.662914813 | GeneCards |
| PRDX6 | 0.662914813 | GeneCards |
| RNF41 | 0.662914813 | GeneCards |
| BCL2L13 | 0.662914813 | GeneCards |
| HLA-W | 0.662914813 | GeneCards |
| MAP3K5 | 0.6568048 | GeneCards |
| NFATC2 | 0.650404334 | GeneCards |
| PDGFA | 0.642342329 | GeneCards |
| FGFR2 | 0.641557217 | GeneCards |
| DDC | 0.641557217 | GeneCards |
| AR | 0.641557217 | GeneCards |
| APP | 0.641557217 | GeneCards |
| CA2 | 0.641557217 | GeneCards |
| TH | 0.641557217 | GeneCards |
| DBH | 0.641557217 | GeneCards |
| CACNA1B | 0.641557217 | GeneCards |
| POR | 0.641557217 | GeneCards |
| PAH | 0.641557217 | GeneCards |
| LIPE | 0.641557217 | GeneCards |
| GCGR | 0.641557217 | GeneCards |
| GCH1 | 0.641557217 | GeneCards |
| AQP2 | 0.641557217 | GeneCards |
| ABCB11 | 0.641557217 | GeneCards |
| TOP2B | 0.641557217 | GeneCards |
| PDP1 | 0.641557217 | GeneCards |
| AFP | 0.641557217 | GeneCards |
| APOB | 0.641557217 | GeneCards |
| CRAT | 0.641557217 | GeneCards |
| CA9 | 0.641557217 | GeneCards |
| CANT1 | 0.641557217 | GeneCards |
| OPRD1 | 0.641557217 | GeneCards |
| RAP1B | 0.641557217 | GeneCards |
| UGT2B7 | 0.641557217 | GeneCards |
| SLC17A5 | 0.641557217 | GeneCards |
| MKI67 | 0.641557217 | GeneCards |
| AOC3 | 0.641557217 | GeneCards |
| IGFBP1 | 0.641557217 | GeneCards |
| GH1 | 0.641557217 | GeneCards |
| ESD | 0.641557217 | GeneCards |
| PDYN | 0.641557217 | GeneCards |
| SERPINF2 | 0.641557217 | GeneCards |
| LAMP1 | 0.641557217 | GeneCards |
| CGA | 0.641557217 | GeneCards |
| TG | 0.641557217 | GeneCards |
| PPP1R3A | 0.641557217 | GeneCards |
| PEMT | 0.641557217 | GeneCards |
| MVP | 0.641557217 | GeneCards |
| OXT | 0.641557217 | GeneCards |
| SHBG | 0.641557217 | GeneCards |
| NISCH | 0.641557217 | GeneCards |
| GIP | 0.641557217 | GeneCards |
| GRK3 | 0.641557217 | GeneCards |
| DSPP | 0.641557217 | GeneCards |
| TAAR1 | 0.641557217 | GeneCards |
| CGB5 | 0.641557217 | GeneCards |
| CBLN3 | 0.641557217 | GeneCards |
| UGT1A | 0.641557217 | GeneCards |
| ADAMTS10 | 0.640660048 | GeneCards |
| ACTL9 | 0.640660048 | GeneCards |
| ATP6V1G2-DDX39B | 0.640660048 | GeneCards |
| NOTCH2 | 0.635687828 | GeneCards |
| CYP26A1 | 0.635687828 | GeneCards |
| CD1A | 0.635687828 | GeneCards |
| CLDN5 | 0.635687828 | GeneCards |
| ILVBL | 0.635687828 | GeneCards |
| TERC | 0.635687828 | GeneCards |
| SLURP1 | 0.633132279 | GeneCards |
| MGST1 | 0.632014394 | GeneCards |
| AGPS | 0.631301165 | GeneCards |
| SERPINB8 | 0.631301165 | GeneCards |
| RAB27A | 0.629085064 | GeneCards |
| CXCL2 | 0.629085064 | GeneCards |
| CASP9 | 0.627136767 | GeneCards |
| KCNS3 | 0.627136767 | GeneCards |
| FCGR2C | 0.620378971 | GeneCards |
| ROCK1 | 0.612016618 | GeneCards |
| RUNX2 | 0.612016618 | GeneCards |
| H4-16 | 0.609660149 | GeneCards |
| TNNT1 | 0.609154105 | GeneCards |
| MARCO | 0.609154105 | GeneCards |
| CYCS | 0.609087288 | GeneCards |
| ENPP2 | 0.607125044 | GeneCards |
| PRDM1 | 0.602277935 | GeneCards |
| IQGAP1 | 0.602277935 | GeneCards |
| CD70 | 0.602277935 | GeneCards |
| USP21 | 0.602277935 | GeneCards |
| MIR20A | 0.596773028 | GeneCards |
| TNFRSF10D | 0.593943357 | GeneCards |
| PTPN6 | 0.593306303 | GeneCards |
| SPDEF | 0.593306303 | GeneCards |
| FOSL1 | 0.589896858 | GeneCards |
| TRDN | 0.589896858 | GeneCards |
| GATA1 | 0.589662373 | GeneCards |
| SOS1 | 0.579923689 | GeneCards |
| GPT2 | 0.578938425 | GeneCards |
| SCN9A | 0.578938425 | GeneCards |
| KDM1A | 0.578938425 | GeneCards |
| ALDH18A1 | 0.578938425 | GeneCards |
| SALL1 | 0.578938425 | GeneCards |
| GPX7 | 0.578938425 | GeneCards |
| CDX2 | 0.578938425 | GeneCards |
| RPIA | 0.578938425 | GeneCards |
| PGC | 0.578938425 | GeneCards |
| MFAP5 | 0.578938425 | GeneCards |
| ACTL6A | 0.578938425 | GeneCards |
| ASCC1 | 0.578938425 | GeneCards |
| CTHRC1 | 0.578938425 | GeneCards |
| ZNF469 | 0.578938425 | GeneCards |
| PGA3 | 0.578938425 | GeneCards |
| CAPN14 | 0.578938425 | GeneCards |
| SDAD1 | 0.578938425 | GeneCards |
| FAM120AOS | 0.578938425 | GeneCards |
| NEXMIF | 0.578938425 | GeneCards |
| MIR30E | 0.578938425 | GeneCards |
| MIR532 | 0.578938425 | GeneCards |
| PPARA | 0.578843892 | GeneCards |
| CD55 | 0.575553417 | GeneCards |
| SPTLC1 | 0.575553417 | GeneCards |
| ORMDL1 | 0.575553417 | GeneCards |
| CCL23 | 0.575553417 | GeneCards |
| ATP1A1 | 0.569213927 | GeneCards |
| PFKM | 0.569213927 | GeneCards |
| CYLD | 0.569213927 | GeneCards |
| THRA | 0.569213927 | GeneCards |
| FLI1 | 0.569213927 | GeneCards |
| DMPK | 0.569213927 | GeneCards |
| ACO2 | 0.569213927 | GeneCards |
| FOXO1 | 0.569213927 | GeneCards |
| GNA11 | 0.569213927 | GeneCards |
| TRAF3 | 0.569213927 | GeneCards |
| NR1D1 | 0.569213927 | GeneCards |
| KIF11 | 0.569213927 | GeneCards |
| FEN1 | 0.569213927 | GeneCards |
| ERCC1 | 0.569213927 | GeneCards |
| CDC25B | 0.569213927 | GeneCards |
| PPP2R1B | 0.569213927 | GeneCards |
| SLC19A3 | 0.569213927 | GeneCards |
| P4HA2 | 0.569213927 | GeneCards |
| BID | 0.569213927 | GeneCards |
| CDC6 | 0.569213927 | GeneCards |
| SMARCC2 | 0.569213927 | GeneCards |
| RELB | 0.569213927 | GeneCards |
| ADCY8 | 0.569213927 | GeneCards |
| MAP3K14 | 0.569213927 | GeneCards |
| CFL2 | 0.569213927 | GeneCards |
| TRIO | 0.569213927 | GeneCards |
| SYNGAP1 | 0.569213927 | GeneCards |
| RUVBL1 | 0.569213927 | GeneCards |
| PI4KB | 0.569213927 | GeneCards |
| ARNT | 0.569213927 | GeneCards |
| GRB7 | 0.569213927 | GeneCards |
| HSPA1L | 0.569213927 | GeneCards |
| C4B | 0.569213927 | GeneCards |
| EGR2 | 0.569213927 | GeneCards |
| SPOP | 0.569213927 | GeneCards |
| TRAF1 | 0.569213927 | GeneCards |
| UNC13D | 0.569213927 | GeneCards |
| SLC1A5 | 0.569213927 | GeneCards |
| PDCD6IP | 0.569213927 | GeneCards |
| PANK2 | 0.569213927 | GeneCards |
| NMT1 | 0.569213927 | GeneCards |
| PPP1R1B | 0.569213927 | GeneCards |
| SIK2 | 0.569213927 | GeneCards |
| FZR1 | 0.569213927 | GeneCards |
| HHEX | 0.569213927 | GeneCards |
| KAT7 | 0.569213927 | GeneCards |
| EFTUD2 | 0.569213927 | GeneCards |
| GNA15 | 0.569213927 | GeneCards |
| ATP6V1G3 | 0.569213927 | GeneCards |
| ATXN2 | 0.569213927 | GeneCards |
| ABCB5 | 0.569213927 | GeneCards |
| DTYMK | 0.569213927 | GeneCards |
| TCAP | 0.569213927 | GeneCards |
| PRIM1 | 0.569213927 | GeneCards |
| SENP1 | 0.569213927 | GeneCards |
| LYVE1 | 0.569213927 | GeneCards |
| RPS26 | 0.569213927 | GeneCards |
| RPL19 | 0.569213927 | GeneCards |
| SCYL1 | 0.569213927 | GeneCards |
| SEC61A1 | 0.569213927 | GeneCards |
| NIN | 0.569213927 | GeneCards |
| CDC27 | 0.569213927 | GeneCards |
| LTK | 0.569213927 | GeneCards |
| BRAP | 0.569213927 | GeneCards |
| AHI1 | 0.569213927 | GeneCards |
| ALG9 | 0.569213927 | GeneCards |
| DTNBP1 | 0.569213927 | GeneCards |
| INCENP | 0.569213927 | GeneCards |
| ABCB9 | 0.569213927 | GeneCards |
| CCNT1 | 0.569213927 | GeneCards |
| TRIM71 | 0.569213927 | GeneCards |
| PLCD3 | 0.569213927 | GeneCards |
| PA2G4 | 0.569213927 | GeneCards |
| RAB18 | 0.569213927 | GeneCards |
| RASA2 | 0.569213927 | GeneCards |
| SNRPD2 | 0.569213927 | GeneCards |
| SLC39A5 | 0.569213927 | GeneCards |
| COLEC10 | 0.569213927 | GeneCards |
| COL16A1 | 0.569213927 | GeneCards |
| BAZ2A | 0.569213927 | GeneCards |
| KLB | 0.569213927 | GeneCards |
| LRP1B | 0.569213927 | GeneCards |
| NEK6 | 0.569213927 | GeneCards |
| GNGT2 | 0.569213927 | GeneCards |
| DEF6 | 0.569213927 | GeneCards |
| CLNS1A | 0.569213927 | GeneCards |
| CLASP2 | 0.569213927 | GeneCards |
| CTDSP2 | 0.569213927 | GeneCards |
| STARD3 | 0.569213927 | GeneCards |
| ZFP57 | 0.569213927 | GeneCards |
| TAF3 | 0.569213927 | GeneCards |
| SYMPK | 0.569213927 | GeneCards |
| TMSB4X | 0.569213927 | GeneCards |
| SP2 | 0.569213927 | GeneCards |
| PPP2R3C | 0.569213927 | GeneCards |
| PKN3 | 0.569213927 | GeneCards |
| PHF19 | 0.569213927 | GeneCards |
| MTA2 | 0.569213927 | GeneCards |
| MYLIP | 0.569213927 | GeneCards |
| MYOG | 0.569213927 | GeneCards |
| RALGAPA1 | 0.569213927 | GeneCards |
| APOF | 0.569213927 | GeneCards |
| HEXIM1 | 0.569213927 | GeneCards |
| CLIC6 | 0.569213927 | GeneCards |
| DIS3L | 0.569213927 | GeneCards |
| ZNF217 | 0.569213927 | GeneCards |
| TOB2 | 0.569213927 | GeneCards |
| ZFYVE26 | 0.569213927 | GeneCards |
| TINAGL1 | 0.569213927 | GeneCards |
| SPSB1 | 0.569213927 | GeneCards |
| POLR2G | 0.569213927 | GeneCards |
| PSMD5 | 0.569213927 | GeneCards |
| ABI3 | 0.569213927 | GeneCards |
| CTR9 | 0.569213927 | GeneCards |
| COG6 | 0.569213927 | GeneCards |
| ADO | 0.569213927 | GeneCards |
| INHBE | 0.569213927 | GeneCards |
| KIN | 0.569213927 | GeneCards |
| FBXL20 | 0.569213927 | GeneCards |
| ESYT1 | 0.569213927 | GeneCards |
| CDK2AP1 | 0.569213927 | GeneCards |
| CSDC2 | 0.569213927 | GeneCards |
| ACBD4 | 0.569213927 | GeneCards |
| BANP | 0.569213927 | GeneCards |
| ARFRP1 | 0.569213927 | GeneCards |
| BTG4 | 0.569213927 | GeneCards |
| CDHR5 | 0.569213927 | GeneCards |
| CHP1 | 0.569213927 | GeneCards |
| DOCK9 | 0.569213927 | GeneCards |
| OLIG3 | 0.569213927 | GeneCards |
| NAV2 | 0.569213927 | GeneCards |
| ZNF76 | 0.569213927 | GeneCards |
| TNS4 | 0.569213927 | GeneCards |
| TSHZ2 | 0.569213927 | GeneCards |
| SNF8 | 0.569213927 | GeneCards |
| SSR3 | 0.569213927 | GeneCards |
| SCUBE3 | 0.569213927 | GeneCards |
| PEF1 | 0.569213927 | GeneCards |
| OTULIN | 0.569213927 | GeneCards |
| NMRK2 | 0.569213927 | GeneCards |
| SEC61B | 0.569213927 | GeneCards |
| PAN2 | 0.569213927 | GeneCards |
| HOXB3 | 0.569213927 | GeneCards |
| ARHGAP27 | 0.569213927 | GeneCards |
| FCHSD1 | 0.569213927 | GeneCards |
| CNTRL | 0.569213927 | GeneCards |
| INO80 | 0.569213927 | GeneCards |
| IPCEF1 | 0.569213927 | GeneCards |
| LY6G6F | 0.569213927 | GeneCards |
| LAYN | 0.569213927 | GeneCards |
| METTL7B | 0.569213927 | GeneCards |
| MEX3C | 0.569213927 | GeneCards |
| GANC | 0.569213927 | GeneCards |
| MACC1 | 0.569213927 | GeneCards |
| FBXW2 | 0.569213927 | GeneCards |
| FMNL1 | 0.569213927 | GeneCards |
| FADS3 | 0.569213927 | GeneCards |
| EAPP | 0.569213927 | GeneCards |
| FAM177A1 | 0.569213927 | GeneCards |
| DCAKD | 0.569213927 | GeneCards |
| DMRTA1 | 0.569213927 | GeneCards |
| ARPP21 | 0.569213927 | GeneCards |
| STAC2 | 0.569213927 | GeneCards |
| TMTC2 | 0.569213927 | GeneCards |
| SNRNP40 | 0.569213927 | GeneCards |
| PLXDC1 | 0.569213927 | GeneCards |
| PDS5B | 0.569213927 | GeneCards |
| PHF5A | 0.569213927 | GeneCards |
| OSBPL7 | 0.569213927 | GeneCards |
| MRPS27 | 0.569213927 | GeneCards |
| LHX2 | 0.569213927 | GeneCards |
| KLHDC4 | 0.569213927 | GeneCards |
| TOMM7 | 0.569213927 | GeneCards |
| ADAMTSL3 | 0.569213927 | GeneCards |
| FOXR1 | 0.569213927 | GeneCards |
| GABPB2 | 0.569213927 | GeneCards |
| DNAJC17 | 0.569213927 | GeneCards |
| GTF3C1 | 0.569213927 | GeneCards |
| IRF2BP1 | 0.569213927 | GeneCards |
| EEFSEC | 0.569213927 | GeneCards |
| CCDC134 | 0.569213927 | GeneCards |
| CPSF7 | 0.569213927 | GeneCards |
| DMWD | 0.569213927 | GeneCards |
| ADGRB2 | 0.569213927 | GeneCards |
| ASB8 | 0.569213927 | GeneCards |
| TCP11 | 0.569213927 | GeneCards |
| ZBTB22 | 0.569213927 | GeneCards |
| SNX20 | 0.569213927 | GeneCards |
| PPP1R3F | 0.569213927 | GeneCards |
| PITPNM2 | 0.569213927 | GeneCards |
| PPFIA4 | 0.569213927 | GeneCards |
| MPHOSPH9 | 0.569213927 | GeneCards |
| INSM2 | 0.569213927 | GeneCards |
| BRMS1L | 0.569213927 | GeneCards |
| TBKBP1 | 0.569213927 | GeneCards |
| VPS13C | 0.569213927 | GeneCards |
| UHRF1BP1 | 0.569213927 | GeneCards |
| ZBTB8A | 0.569213927 | GeneCards |
| ZC3H10 | 0.569213927 | GeneCards |
| PIGX | 0.569213927 | GeneCards |
| SLC48A1 | 0.569213927 | GeneCards |
| NRROS | 0.569213927 | GeneCards |
| NSMCE1 | 0.569213927 | GeneCards |
| MYRF | 0.569213927 | GeneCards |
| ANKRD52 | 0.569213927 | GeneCards |
| DNAJB8 | 0.569213927 | GeneCards |
| C6orf58 | 0.569213927 | GeneCards |
| LY6G6C | 0.569213927 | GeneCards |
| MFSD9 | 0.569213927 | GeneCards |
| MICAL3 | 0.569213927 | GeneCards |
| MIEN1 | 0.569213927 | GeneCards |
| HUS1B | 0.569213927 | GeneCards |
| KANSL2 | 0.569213927 | GeneCards |
| DCAF5 | 0.569213927 | GeneCards |
| EXD1 | 0.569213927 | GeneCards |
| ABHD16A | 0.569213927 | GeneCards |
| CAPSL | 0.569213927 | GeneCards |
| EML3 | 0.569213927 | GeneCards |
| ENDOU | 0.569213927 | GeneCards |
| ZNF366 | 0.569213927 | GeneCards |
| TMEM106C | 0.569213927 | GeneCards |
| TRIM15 | 0.569213927 | GeneCards |
| ZBTB12 | 0.569213927 | GeneCards |
| SUSD5 | 0.569213927 | GeneCards |
| PRM2 | 0.569213927 | GeneCards |
| RPAP1 | 0.569213927 | GeneCards |
| RPAP3 | 0.569213927 | GeneCards |
| SCRN2 | 0.569213927 | GeneCards |
| PTCD2 | 0.569213927 | GeneCards |
| QPCTL | 0.569213927 | GeneCards |
| R3HDM2 | 0.569213927 | GeneCards |
| TNP2 | 0.569213927 | GeneCards |
| ZCCHC2 | 0.569213927 | GeneCards |
| DBX1 | 0.569213927 | GeneCards |
| C12orf43 | 0.569213927 | GeneCards |
| C6orf15 | 0.569213927 | GeneCards |
| LY6G6D | 0.569213927 | GeneCards |
| GHDC | 0.569213927 | GeneCards |
| FAM114A1 | 0.569213927 | GeneCards |
| CNOT10 | 0.569213927 | GeneCards |
| CLASRP | 0.569213927 | GeneCards |
| ZNF557 | 0.569213927 | GeneCards |
| TSSK3 | 0.569213927 | GeneCards |
| MTFR2 | 0.569213927 | GeneCards |
| POU5F1B | 0.569213927 | GeneCards |
| SETD4 | 0.569213927 | GeneCards |
| OR10AD1 | 0.569213927 | GeneCards |
| SERPINB11 | 0.569213927 | GeneCards |
| PRUNE1 | 0.569213927 | GeneCards |
| ZBTB9 | 0.569213927 | GeneCards |
| CCDC61 | 0.569213927 | GeneCards |
| LMBRD2 | 0.569213927 | GeneCards |
| ZC3H18 | 0.569213927 | GeneCards |
| SBNO2 | 0.569213927 | GeneCards |
| SLC45A1 | 0.569213927 | GeneCards |
| MPIG6B | 0.569213927 | GeneCards |
| AP5B1 | 0.569213927 | GeneCards |
| FAM9C | 0.569213927 | GeneCards |
| DEXI | 0.569213927 | GeneCards |
| ZNF311 | 0.569213927 | GeneCards |
| PRR5L | 0.569213927 | GeneCards |
| RPUSD2 | 0.569213927 | GeneCards |
| SOGA3 | 0.569213927 | GeneCards |
| ZBTB39 | 0.569213927 | GeneCards |
| FBXO46 | 0.569213927 | GeneCards |
| FAM117A | 0.569213927 | GeneCards |
| TMEM258 | 0.569213927 | GeneCards |
| OR10P1 | 0.569213927 | GeneCards |
| ZBTB8B | 0.569213927 | GeneCards |
| IRAG1 | 0.569213927 | GeneCards |
| EFCAB13 | 0.569213927 | GeneCards |
| CCDC184 | 0.569213927 | GeneCards |
| PRM3 | 0.569213927 | GeneCards |
| MYPOP | 0.569213927 | GeneCards |
| SPRR2F | 0.569213927 | GeneCards |
| RPL41 | 0.569213927 | GeneCards |
| PYM1 | 0.569213927 | GeneCards |
| KATNIP | 0.569213927 | GeneCards |
| RELCH | 0.569213927 | GeneCards |
| ILRUN | 0.569213927 | GeneCards |
| LRRC3C | 0.569213927 | GeneCards |
| NEMP1 | 0.569213927 | GeneCards |
| STEAP1B | 0.569213927 | GeneCards |
| LY6G6E | 0.569213927 | GeneCards |
| EEF1AKMT3 | 0.569213927 | GeneCards |
| SNHG32 | 0.569213927 | GeneCards |
| HOATZ | 0.569213927 | GeneCards |
| KRT10-AS1 | 0.569213927 | GeneCards |
| MIR34B | 0.569213927 | GeneCards |
| KIRREL3-AS3 | 0.569213927 | GeneCards |
| MIR219A1 | 0.569213927 | GeneCards |
| SNORD52 | 0.569213927 | GeneCards |
| HCG18 | 0.569213927 | GeneCards |
| TEX41 | 0.569213927 | GeneCards |
| RUNX1-IT1 | 0.569213927 | GeneCards |
| MSH5-SAPCD1 | 0.569213927 | GeneCards |
| SNORA21 | 0.569213927 | GeneCards |
| TIPARP-AS1 | 0.569213927 | GeneCards |
| MIR611 | 0.569213927 | GeneCards |
| RAD51-AS1 | 0.569213927 | GeneCards |
| SNORA38 | 0.569213927 | GeneCards |
| SNORD84 | 0.569213927 | GeneCards |
| SPRR2C | 0.569213927 | GeneCards |
| SENCR | 0.569213927 | GeneCards |
| SNORD48 | 0.569213927 | GeneCards |
| AGAP2-AS1 | 0.569213927 | GeneCards |
| GUCY2EP | 0.569213927 | GeneCards |
| SPRY4-AS1 | 0.569213927 | GeneCards |
| PABPC1P2 | 0.569213927 | GeneCards |
| SLC8A1-AS1 | 0.569213927 | GeneCards |
| IRAG1-AS1 | 0.569213927 | GeneCards |
| LINC01208 | 0.569213927 | GeneCards |
| LPP-AS1 | 0.569213927 | GeneCards |
| TMEM198B | 0.569213927 | GeneCards |
| MTMR9LP | 0.569213927 | GeneCards |
| LOC100506178 | 0.569213927 | GeneCards |
| WAKMAR2 | 0.569213927 | GeneCards |
| FLJ42393 | 0.569213927 | GeneCards |
| MIR548AN | 0.569213927 | GeneCards |
| SDHAP1 | 0.569213927 | GeneCards |
| RARA-AS1 | 0.569213927 | GeneCards |
| IL6-AS1 | 0.569213927 | GeneCards |
| MICD | 0.569213927 | GeneCards |
| MIR4646 | 0.569213927 | GeneCards |
| LINC02098 | 0.569213927 | GeneCards |
| HLA-DQB3 | 0.569213927 | GeneCards |
| ETS1-AS1 | 0.569213927 | GeneCards |
| ATXN2-AS | 0.569213927 | GeneCards |
| MRPL45P2 | 0.569213927 | GeneCards |
| NSMCE1-DT | 0.569213927 | GeneCards |
| SRP54-AS1 | 0.569213927 | GeneCards |
| RPL21P124 | 0.569213927 | GeneCards |
| LINC01991 | 0.569213927 | GeneCards |
| NFE2L1-DT | 0.569213927 | GeneCards |
| GNA15-DT | 0.569213927 | GeneCards |
| CNOT10-AS1 | 0.569213927 | GeneCards |
| CUTALP | 0.569213927 | GeneCards |
| LOC101929563 | 0.569213927 | GeneCards |
| TMED2-DT | 0.569213927 | GeneCards |
| ENSG00000257411 | 0.569213927 | GeneCards |
| LINC01149 | 0.569213927 | GeneCards |
| LINC02757 | 0.569213927 | GeneCards |
| LINC02354 | 0.569213927 | GeneCards |
| NFILZ | 0.569213927 | GeneCards |
| UBE2D3P3 | 0.569213927 | GeneCards |
| RNU1-27P | 0.569213927 | GeneCards |
| ENSG00000250751 | 0.569213927 | GeneCards |
| ENSG00000253347 | 0.569213927 | GeneCards |
| ENSG00000257303 | 0.569213927 | GeneCards |
| ENSG00000259199 | 0.569213927 | GeneCards |
| ENSG00000266601 | 0.569213927 | GeneCards |
| ENSG00000262837 | 0.569213927 | GeneCards |
| ENSG00000250264 | 0.569213927 | GeneCards |
| ENSG00000259940 | 0.569213927 | GeneCards |
| ENSG00000261030 | 0.569213927 | GeneCards |
| ENSG00000263020 | 0.569213927 | GeneCards |
| ENSG00000225172 | 0.569213927 | GeneCards |
| ENSG00000234389 | 0.569213927 | GeneCards |
| ENSG00000229167 | 0.569213927 | GeneCards |
| ENSG00000234775 | 0.569213927 | GeneCards |
| ENSG00000230533 | 0.569213927 | GeneCards |
| LOC100294145 | 0.569213927 | GeneCards |
| SUGT1P2 | 0.569213927 | GeneCards |
| SCYGR8 | 0.569213927 | GeneCards |
| PEF1-AS1 | 0.569213927 | GeneCards |
| PHC1P1 | 0.569213927 | GeneCards |
| RPL32P23 | 0.569213927 | GeneCards |
| RPL6P5 | 0.569213927 | GeneCards |
| RPS19P3 | 0.569213927 | GeneCards |
| RNY4P10 | 0.569213927 | GeneCards |
| PANK2-AS1 | 0.569213927 | GeneCards |
| CCR12P | 0.569213927 | GeneCards |
| GOT2P2 | 0.569213927 | GeneCards |
| RN7SL809P | 0.569213927 | GeneCards |
| PTP4A2P1 | 0.569213927 | GeneCards |
| ENSG00000258317 | 0.569213927 | GeneCards |
| ENSG00000269514 | 0.569213927 | GeneCards |
| ENSG00000254975 | 0.569213927 | GeneCards |
| ENSG00000261002 | 0.569213927 | GeneCards |
| ENSG00000261573 | 0.569213927 | GeneCards |
| ENSG00000267114 | 0.569213927 | GeneCards |
| ENSG00000270120 | 0.569213927 | GeneCards |
| ENSG00000254980 | 0.569213927 | GeneCards |
| ENSG00000256569 | 0.569213927 | GeneCards |
| ENSG00000257740 | 0.569213927 | GeneCards |
| ENSG00000268810 | 0.569213927 | GeneCards |
| ENSG00000205653 | 0.569213927 | GeneCards |
| ENSG00000212228 | 0.569213927 | GeneCards |
| ENSG00000221083 | 0.569213927 | GeneCards |
| ENSG00000232638 | 0.569213927 | GeneCards |
| ENSG00000225107 | 0.569213927 | GeneCards |
| ENSG00000205537 | 0.569213927 | GeneCards |
| SLC25A38P1 | 0.569213927 | GeneCards |
| MIR6832 | 0.569213927 | GeneCards |
| RNU6-887P | 0.569213927 | GeneCards |
| RNY4P14 | 0.569213927 | GeneCards |
| RNU6-343P | 0.569213927 | GeneCards |
| RNU6-850P | 0.569213927 | GeneCards |
| RNU6-1169P | 0.569213927 | GeneCards |
| BCLAF1P1 | 0.569213927 | GeneCards |
| DNAJC8P1 | 0.569213927 | GeneCards |
| ENSG00000251405 | 0.569213927 | GeneCards |
| ENSG00000269148 | 0.569213927 | GeneCards |
| ENSG00000226990 | 0.569213927 | GeneCards |
| ENSG00000229664 | 0.569213927 | GeneCards |
| ENSG00000199332 | 0.569213927 | GeneCards |
| YBX1P5 | 0.569213927 | GeneCards |
| SPRR5 | 0.569213927 | GeneCards |
| SUMO2P10 | 0.569213927 | GeneCards |
| SEC13P1 | 0.569213927 | GeneCards |
| PEBP1P3 | 0.569213927 | GeneCards |
| CHCHD3P1 | 0.569213927 | GeneCards |
| LOC102725180 | 0.569213927 | GeneCards |
| ENSG00000267446 | 0.569213927 | GeneCards |
| ENSG00000269489 | 0.569213927 | GeneCards |
| ENSG00000273424 | 0.569213927 | GeneCards |
| ENSG00000274737 | 0.569213927 | GeneCards |
| ENSG00000283265 | 0.569213927 | GeneCards |
| ENSG00000285448 | 0.569213927 | GeneCards |
| ENSG00000268069 | 0.569213927 | GeneCards |
| ENSG00000236864 | 0.569213927 | GeneCards |
| LOC390876 | 0.569213927 | GeneCards |
| SRSF10P1 | 0.569213927 | GeneCards |
| RN7SL335P | 0.569213927 | GeneCards |
| lnc-LRRC3C-2 | 0.569213927 | GeneCards |
| lnc-STK11-2 | 0.569213927 | GeneCards |
| lnc-GNA15-1 | 0.569213927 | GeneCards |
| lnc-GTF3C1-3 | 0.569213927 | GeneCards |
| lnc-LTK-1 | 0.569213927 | GeneCards |
| MTCYBP42 | 0.569213927 | GeneCards |
| ENSG00000266944 | 0.569213927 | GeneCards |
| ENSG00000250347 | 0.569213927 | GeneCards |
| ENSG00000273576 | 0.569213927 | GeneCards |
| ENSG00000285551 | 0.569213927 | GeneCards |
| ENSG00000276691 | 0.569213927 | GeneCards |
| ENSG00000285837 | 0.569213927 | GeneCards |
| ENSG00000287121 | 0.569213927 | GeneCards |
| ENSG00000271547 | 0.569213927 | GeneCards |
| ENSG00000237868 | 0.569213927 | GeneCards |
| LOC100533842 | 0.569213927 | GeneCards |
| lnc-COL11A2-1 | 0.569213927 | GeneCards |
| lnc-DTYMK-5 | 0.569213927 | GeneCards |
| lnc-ETS1-8 | 0.569213927 | GeneCards |
| lnc-GRB7-1 | 0.569213927 | GeneCards |
| lnc-NEMP1-1 | 0.569213927 | GeneCards |
| lnc-PXYLP1-3 | 0.569213927 | GeneCards |
| lnc-STAT6-1 | 0.569213927 | GeneCards |
| lnc-TFDP2-12 | 0.569213927 | GeneCards |
| lnc-TOB2-1 | 0.569213927 | GeneCards |
| HSALNG0031449 | 0.569213927 | GeneCards |
| HSALNG0049245-002 | 0.569213927 | GeneCards |
| HSALNG0049410 | 0.569213927 | GeneCards |
| HSALNG0049418 | 0.569213927 | GeneCards |
| ENSG00000240399 | 0.569213927 | GeneCards |
| AC005262 | 0.569213927 | GeneCards |
| lnc-BCL6-7 | 0.569213927 | GeneCards |
| lnc-BCL6-9 | 0.569213927 | GeneCards |
| lnc-BTG4-4 | 0.569213927 | GeneCards |
| HSALNG0017399 | 0.569213927 | GeneCards |
| HSALNG0031953 | 0.569213927 | GeneCards |
| HSALNG0045489 | 0.569213927 | GeneCards |
| HSALNG0076038 | 0.569213927 | GeneCards |
| HSALNG0117030 | 0.569213927 | GeneCards |
| HSALNG0117176 | 0.569213927 | GeneCards |
| piR-45012-054 | 0.569213927 | GeneCards |
| HSALNG0076041 | 0.569213927 | GeneCards |
| HSALNG0078328 | 0.569213927 | GeneCards |
| HSALNG0082653-001 | 0.569213927 | GeneCards |
| HSALNG0116294 | 0.569213927 | GeneCards |
| HSALNG0116298 | 0.569213927 | GeneCards |
| HSALNG0132875 | 0.569213927 | GeneCards |
| HSALNG0136688-001 | 0.569213927 | GeneCards |
| L13304-009 | 0.569213927 | GeneCards |
| LINC01862 | 0.569213927 | GeneCards |
| lnc-ADGRB2-1 | 0.569213927 | GeneCards |
| MN296981 | 0.569213927 | GeneCards |
| NONHSAG005179.2-001 | 0.569213927 | GeneCards |
| NONHSAG005179.2-002 | 0.569213927 | GeneCards |
| NONHSAG011408.2 | 0.569213927 | GeneCards |
| piR-36475-004 | 0.569213927 | GeneCards |
| piR-37824 | 0.569213927 | GeneCards |
| piR-48841 | 0.569213927 | GeneCards |
| lnc-FLI1-3 | 0.569213927 | GeneCards |
| lnc-GJA10-23 | 0.569213927 | GeneCards |
| lnc-GSDMB-1 | 0.569213927 | GeneCards |
| lnc-GSDMB-2 | 0.569213927 | GeneCards |
| lnc-GTF3C1-4 | 0.569213927 | GeneCards |
| lnc-INSM2-5 | 0.569213927 | GeneCards |
| lnc-IRF2BP1-1 | 0.569213927 | GeneCards |
| lnc-LYVE1-1 | 0.569213927 | GeneCards |
| lnc-MIEN1-1 | 0.569213927 | GeneCards |
| lnc-VARS-3 | 0.569213927 | GeneCards |
| piR-59591 | 0.569213927 | GeneCards |
| HSALNG0084560 | 0.569213927 | GeneCards |
| HSALNG0084561-001 | 0.569213927 | GeneCards |
| HSALNG0100587 | 0.569213927 | GeneCards |
| HSALNG0100588 | 0.569213927 | GeneCards |
| lnc-ATP6V1G3-5 | 0.569213927 | GeneCards |
| lnc-ATP6V1G3-6 | 0.569213927 | GeneCards |
| lnc-NAB2-1 | 0.569213927 | GeneCards |
| lnc-TMPPE-1-001 | 0.569213927 | GeneCards |
| lnc-TMPPE-1-002 | 0.569213927 | GeneCards |
| lnc-TMSB4X-10 | 0.569213927 | GeneCards |
| RF00017-3789 | 0.569213927 | GeneCards |
| RF00017-3803 | 0.569213927 | GeneCards |
| RF00017-4970 | 0.569213927 | GeneCards |
| RF00994-504 | 0.569213927 | GeneCards |
| HSALNG0091490 | 0.569213927 | GeneCards |
| HSALNG0105356-002 | 0.569213927 | GeneCards |
| HSALNG0132882 | 0.569213927 | GeneCards |
| hsa-miR-5096-184 | 0.569213927 | GeneCards |
| lnc-AES-7 | 0.569213927 | GeneCards |
| lnc-GATA3-14 | 0.569213927 | GeneCards |
| lnc-HLA-DOA-1 | 0.569213927 | GeneCards |
| lnc-HLA-DPA1-2 | 0.569213927 | GeneCards |
| lnc-HLA-DQA1-10 | 0.569213927 | GeneCards |
| lnc-LRP1-1 | 0.569213927 | GeneCards |
| lnc-NEK7-4 | 0.569213927 | GeneCards |
| lnc-OLIG3-1 | 0.569213927 | GeneCards |
| lnc-TLR1-1 | 0.569213927 | GeneCards |
| lnc-TMEM258-1 | 0.569213927 | GeneCards |
| MN297160 | 0.569213927 | GeneCards |
| NONHSAG003874.2 | 0.569213927 | GeneCards |
| piR-52079-043 | 0.569213927 | GeneCards |
| RF00017-2182 | 0.569213927 | GeneCards |
| RF00017-2723 | 0.569213927 | GeneCards |
| RF00017-7561 | 0.569213927 | GeneCards |
| LOC107984204 | 0.569213927 | GeneCards |
| ENSG00000271251 | 0.569213927 | GeneCards |
| ENSG00000285619 | 0.569213927 | GeneCards |
| LOC102723798 | 0.569213927 | GeneCards |
| LOC112268187 | 0.569213927 | GeneCards |
| lnc-POP5-3 | 0.569213927 | GeneCards |
| lnc-RAPGEF3-2 | 0.569213927 | GeneCards |
| HSALNG0033906 | 0.569213927 | GeneCards |
| HSALNG0044870 | 0.569213927 | GeneCards |
| HSALNG0056489 | 0.569213927 | GeneCards |
| ENSG00000244061 | 0.569213927 | GeneCards |
| lnc-ZPBP2-5 | 0.569213927 | GeneCards |
| lnc-BRD2-4 | 0.569213927 | GeneCards |
| lnc-BRD2-5 | 0.569213927 | GeneCards |
| lnc-BRD2-6 | 0.569213927 | GeneCards |
| HSALNG0041154 | 0.569213927 | GeneCards |
| HSALNG0049308 | 0.569213927 | GeneCards |
| HSALNG0049307 | 0.569213927 | GeneCards |
| HSALNG0017433 | 0.569213927 | GeneCards |
| HSALNG0025043 | 0.569213927 | GeneCards |
| HSALNG0031446 | 0.569213927 | GeneCards |
| HSALNG0047289 | 0.569213927 | GeneCards |
| HSALNG0049306 | 0.569213927 | GeneCards |
| HSALNG0049436 | 0.569213927 | GeneCards |
| HSALNG0049667 | 0.569213927 | GeneCards |
| HSALNG0049668 | 0.569213927 | GeneCards |
| HSALNG0088091-002 | 0.569213927 | GeneCards |
| HSALNG0088091-003 | 0.569213927 | GeneCards |
| HSALNG0088091-004 | 0.569213927 | GeneCards |
| HSALNG0088091-005 | 0.569213927 | GeneCards |
| HSALNG0088094 | 0.569213927 | GeneCards |
| HSALNG0098905 | 0.569213927 | GeneCards |
| HSALNG0117031-001 | 0.569213927 | GeneCards |
| HSALNG0082653-002 | 0.569213927 | GeneCards |
| HSALNG0085862 | 0.569213927 | GeneCards |
| HSALNG0110446 | 0.569213927 | GeneCards |
| lnc-ADGRB2-2 | 0.569213927 | GeneCards |
| piR-31462-009 | 0.569213927 | GeneCards |
| piR-31470-402 | 0.569213927 | GeneCards |
| piR-38051-185 | 0.569213927 | GeneCards |
| piR-38319 | 0.569213927 | GeneCards |
| lnc-HLA-DRB1-3 | 0.569213927 | GeneCards |
| lnc-PMEL-4 | 0.569213927 | GeneCards |
| RF00017-4498 | 0.569213927 | GeneCards |
| HSALNG0087123 | 0.569213927 | GeneCards |
| HSALNG0100593 | 0.569213927 | GeneCards |
| lnc-LINGO4-2 | 0.569213927 | GeneCards |
| piR-31264 | 0.569213927 | GeneCards |
| HSALNG0085871 | 0.569213927 | GeneCards |
| lnc-GATA3-13 | 0.569213927 | GeneCards |
| NONHSAG019426.2 | 0.569213927 | GeneCards |
| piR-41111-001 | 0.569213927 | GeneCards |
| piR-52438-090 | 0.569213927 | GeneCards |
| piR-61101-081 | 0.569213927 | GeneCards |
| LOC105370790 | 0.569213927 | GeneCards |
| LOC105371081 | 0.569213927 | GeneCards |
| LOC107984408 | 0.569213927 | GeneCards |
| ENSG00000279737 | 0.569213927 | GeneCards |
| LOC105378204 | 0.569213927 | GeneCards |
| LOC105378327 | 0.569213927 | GeneCards |
| lnc-OR2Z1-2 | 0.569213927 | GeneCards |
| lnc-TRPC3-3 | 0.569213927 | GeneCards |
| HSALNG0017411 | 0.569213927 | GeneCards |
| HSALNG0042579 | 0.569213927 | GeneCards |
| HSALNG0007189 | 0.569213927 | GeneCards |
| HSALNG0044865-001 | 0.569213927 | GeneCards |
| HSALNG0036841 | 0.569213927 | GeneCards |
| HSALNG0028790 | 0.569213927 | GeneCards |
| HSALNG0033896 | 0.569213927 | GeneCards |
| HSALNG0045490 | 0.569213927 | GeneCards |
| HSALNG0045491 | 0.569213927 | GeneCards |
| HSALNG0047290 | 0.569213927 | GeneCards |
| HSALNG0049325 | 0.569213927 | GeneCards |
| HSALNG0076061 | 0.569213927 | GeneCards |
| HSALNG0123782 | 0.569213927 | GeneCards |
| piR-39098-183 | 0.569213927 | GeneCards |
| piR-41306-189 | 0.569213927 | GeneCards |
| HSALNG0069841 | 0.569213927 | GeneCards |
| HSALNG0116286 | 0.569213927 | GeneCards |
| HSALNG0116300 | 0.569213927 | GeneCards |
| HSALNG0137876 | 0.569213927 | GeneCards |
| NONHSAG008489.2 | 0.569213927 | GeneCards |
| NONHSAG045745.2 | 0.569213927 | GeneCards |
| piR-42491-150 | 0.569213927 | GeneCards |
| lnc-LTA-1 | 0.569213927 | GeneCards |
| piR-61945-206 | 0.569213927 | GeneCards |
| HSALNG0082077 | 0.569213927 | GeneCards |
| HSALNG0082078 | 0.569213927 | GeneCards |
| HSALNG0084553 | 0.569213927 | GeneCards |
| HSALNG0084561-002 | 0.569213927 | GeneCards |
| lnc-IL21-2 | 0.569213927 | GeneCards |
| piR-40666-037 | 0.569213927 | GeneCards |
| RF00017-1190 | 0.569213927 | GeneCards |
| RF00017-4252 | 0.569213927 | GeneCards |
| RF00017-4925 | 0.569213927 | GeneCards |
| HSALNG0085863 | 0.569213927 | GeneCards |
| HSALNG0094046 | 0.569213927 | GeneCards |
| HSALNG0098909 | 0.569213927 | GeneCards |
| NONHSAG046314.2 | 0.569213927 | GeneCards |
| piR-37170-024 | 0.569213927 | GeneCards |
| piR-38580-086 | 0.569213927 | GeneCards |
| piR-56037-030 | 0.569213927 | GeneCards |
| RF00017-2135 | 0.569213927 | GeneCards |
| RF00017-3481 | 0.569213927 | GeneCards |
| RF00019-026 | 0.569213927 | GeneCards |
| LOC105375021 | 0.569213927 | GeneCards |
| ENSG00000229172 | 0.569213927 | GeneCards |
| HSALNG0008502 | 0.569213927 | GeneCards |
| HSALNG0036840 | 0.569213927 | GeneCards |
| HSALNG0123784 | 0.569213927 | GeneCards |
| HSALNG0116288 | 0.569213927 | GeneCards |
| piR-57133-512 | 0.569213927 | GeneCards |
| piR-59907-004 | 0.569213927 | GeneCards |
| piR-58302 | 0.569213927 | GeneCards |
| CDKN3 | 0.567542613 | GeneCards |
| MMP13 | 0.564789772 | GeneCards |
| GRB14 | 0.564789772 | GeneCards |
| UBE2E2 | 0.564789772 | GeneCards |
| SART1 | 0.564789772 | GeneCards |
| POU2AF1 | 0.564789772 | GeneCards |
| FCGR3B | 0.56363833 | GeneCards |
| CXCL6 | 0.56363833 | GeneCards |
| BCL2L1 | 0.555555642 | GeneCards |
| IL1RAP | 0.555555642 | GeneCards |
| PRKCD | 0.547561765 | GeneCards |
| CDKN2A | 0.547561765 | GeneCards |
| IRAK4 | 0.547561765 | GeneCards |
| CDKN2B | 0.547561765 | GeneCards |
| IRS2 | 0.547561765 | GeneCards |
| EFNA1 | 0.547561765 | GeneCards |
| SMAP2 | 0.547561765 | GeneCards |
| CCDC26 | 0.547561765 | GeneCards |
| PLK1 | 0.542435527 | GeneCards |
| MPL | 0.542435527 | GeneCards |
| SLC9A3R1 | 0.542435527 | GeneCards |
| APEX1 | 0.542435527 | GeneCards |
| CD9 | 0.542435527 | GeneCards |
| PSMC6 | 0.542435527 | GeneCards |
| LACTB | 0.542435527 | GeneCards |
| ABCA3 | 0.541884303 | GeneCards |
| FGF7 | 0.541884303 | GeneCards |
| IFI27 | 0.541884303 | GeneCards |
| FLT3 | 0.537600279 | GeneCards |
| NRAS | 0.537600279 | GeneCards |
| DGKE | 0.537600279 | GeneCards |
| ERCC3 | 0.537600279 | GeneCards |
| F9 | 0.537600279 | GeneCards |
| ATP7A | 0.537600279 | GeneCards |
| GNPAT | 0.537600279 | GeneCards |
| GALR1 | 0.537600279 | GeneCards |
| DCLRE1C | 0.537600279 | GeneCards |
| PSMA3 | 0.537600279 | GeneCards |
| RNASEH2A | 0.537600279 | GeneCards |
| PEX7 | 0.537600279 | GeneCards |
| FAR1 | 0.537600279 | GeneCards |
| CETN2 | 0.537600279 | GeneCards |
| PSMD12 | 0.537600279 | GeneCards |
| SNAP29 | 0.537600279 | GeneCards |
| GJC2 | 0.537600279 | GeneCards |
| CEP290 | 0.537600279 | GeneCards |
| TSHZ1 | 0.537600279 | GeneCards |
| SLC35C1 | 0.537600279 | GeneCards |
| SMTN | 0.537600279 | GeneCards |
| NELFA | 0.537600279 | GeneCards |
| RNASEH2B | 0.537600279 | GeneCards |
| SLAMF8 | 0.537600279 | GeneCards |
| SALL3 | 0.537600279 | GeneCards |
| PEX5L | 0.537600279 | GeneCards |
| ZNF516 | 0.537600279 | GeneCards |
| ZNF236 | 0.537600279 | GeneCards |
| TULP4 | 0.537600279 | GeneCards |
| TTC28 | 0.537600279 | GeneCards |
| SEPTIN5 | 0.537600279 | GeneCards |
| CEP295 | 0.537600279 | GeneCards |
| RMRP | 0.537600279 | GeneCards |
| TTF2 | 0.535008132 | GeneCards |
| HAS2-AS1 | 0.535008132 | GeneCards |
| DUBR | 0.535008132 | GeneCards |
| CDKN1B | 0.533435881 | GeneCards |
| CSF3R | 0.533435881 | GeneCards |
| CXCL3 | 0.533435881 | GeneCards |
| CRISP3 | 0.533435881 | GeneCards |
| DDR1 | 0.532078803 | GeneCards |
| TYK2 | 0.53044492 | GeneCards |
| KCNMA1 | 0.53044492 | GeneCards |
| CTNNAL1 | 0.53044492 | GeneCards |
| MIR216A | 0.53044492 | GeneCards |
| GATA4 | 0.512653887 | GeneCards |
| TKT | 0.512653887 | GeneCards |
| ENTPD1 | 0.512653887 | GeneCards |
| FGF10 | 0.512653887 | GeneCards |
| GUCA2A | 0.512653887 | GeneCards |
| ITGA5 | 0.512055039 | GeneCards |
| ANG | 0.512055039 | GeneCards |
| S1PR3 | 0.512055039 | GeneCards |
| USF1 | 0.512055039 | GeneCards |
| IL22RA1 | 0.512055039 | GeneCards |
| TNFRSF10C | 0.512055039 | GeneCards |
| FCGR1B | 0.512055039 | GeneCards |
| ADH1B | 0.510553539 | GeneCards |
| SPTLC2 | 0.508328795 | GeneCards |
| TNFAIP6 | 0.508328795 | GeneCards |
| TMPRSS11D | 0.508328795 | GeneCards |
| LMF1 | 0.508328795 | GeneCards |
| HS3ST6 | 0.508328795 | GeneCards |
| FBXL16 | 0.508328795 | GeneCards |
| WDR90 | 0.508328795 | GeneCards |
| PRR25 | 0.508328795 | GeneCards |
| CPB2 | 0.506747663 | GeneCards |
| ACRV1 | 0.506747663 | GeneCards |
| GAD1 | 0.503072143 | GeneCards |
| LRRK2 | 0.503072143 | GeneCards |
| SNCA | 0.503072143 | GeneCards |
| GAD2 | 0.503072143 | GeneCards |
| ITGA3 | 0.503072143 | GeneCards |
| CNTN2 | 0.503072143 | GeneCards |
| CCKBR | 0.503072143 | GeneCards |
| BTD | 0.503072143 | GeneCards |
| CEACAM5 | 0.503072143 | GeneCards |
| G6PC3 | 0.503072143 | GeneCards |
| SIGLEC5 | 0.503072143 | GeneCards |
| MIR29A | 0.503072143 | GeneCards |
| MIR132 | 0.503072143 | GeneCards |
| MIR483 | 0.503072143 | GeneCards |
| MIR486-1 | 0.503072143 | GeneCards |
| BCR | 0.496195942 | GeneCards |
| RARB | 0.496195942 | GeneCards |
| VLDLR | 0.496195942 | GeneCards |
| IDE | 0.496195942 | GeneCards |
| TNFSF11 | 0.496195942 | GeneCards |
| ITPKB | 0.496195942 | GeneCards |
| BAK1 | 0.496195942 | GeneCards |
| TCF7 | 0.496195942 | GeneCards |
| TNFSF15 | 0.496195942 | GeneCards |
| PTPN3 | 0.496195942 | GeneCards |
| ARHGAP5 | 0.496195942 | GeneCards |
| ECM1 | 0.496195942 | GeneCards |
| PEX14 | 0.496195942 | GeneCards |
| GRB10 | 0.496195942 | GeneCards |
| S100A11 | 0.496195942 | GeneCards |
| ACTN3 | 0.496195942 | GeneCards |
| NPAS3 | 0.496195942 | GeneCards |
| CIRBP | 0.496195942 | GeneCards |
| PLCL1 | 0.496195942 | GeneCards |
| SLC52A2 | 0.496195942 | GeneCards |
| THSD4 | 0.496195942 | GeneCards |
| SPRR1B | 0.496195942 | GeneCards |
| MUC16 | 0.496195942 | GeneCards |
| TNFSF8 | 0.496195942 | GeneCards |
| TONSL | 0.496195942 | GeneCards |
| SPRR2A | 0.496195942 | GeneCards |
| MUC21 | 0.496195942 | GeneCards |
| OR10J3 | 0.496195942 | GeneCards |
| OR10J7P | 0.496195942 | GeneCards |
| NCF2 | 0.492057204 | GeneCards |
| RTN4 | 0.492057204 | GeneCards |
| PDE7A | 0.492057204 | GeneCards |
| MCAM | 0.480885565 | GeneCards |
| ITGA2B | 0.473841697 | GeneCards |
| RIPK1 | 0.473841697 | GeneCards |
| KAT2B | 0.473841697 | GeneCards |
| KDM6A | 0.473841697 | GeneCards |
| TBX5 | 0.473841697 | GeneCards |
| DAB2 | 0.473841697 | GeneCards |
| PSMA5 | 0.473841697 | GeneCards |
| PGF | 0.473841697 | GeneCards |
| SI | 0.473841697 | GeneCards |
| IFI16 | 0.473841697 | GeneCards |
| KAT6B | 0.473841697 | GeneCards |
| DPEP2 | 0.473841697 | GeneCards |
| LILRB3 | 0.473841697 | GeneCards |
| RLN1 | 0.473841697 | GeneCards |
| ESS2 | 0.473841697 | GeneCards |
| MIR183 | 0.473841697 | GeneCards |
| YY1 | 0.469937444 | GeneCards |
| NFIL3 | 0.469937444 | GeneCards |
| CD151 | 0.458630741 | GeneCards |
| SLCO1B3 | 0.453649431 | GeneCards |
| ADGRG3 | 0.453649431 | GeneCards |
| CACNA1C | 0.447692126 | GeneCards |
| ACHE | 0.447692126 | GeneCards |
| MMP10 | 0.447692126 | GeneCards |
| COL4A3 | 0.447692126 | GeneCards |
| CHRNA3 | 0.447692126 | GeneCards |
| CHRNA5 | 0.447692126 | GeneCards |
| PDE5A | 0.447692126 | GeneCards |
| EMX2 | 0.447692126 | GeneCards |
| MMP25 | 0.447692126 | GeneCards |
| ZFP36 | 0.447692126 | GeneCards |
| CDKN1A | 0.444830358 | GeneCards |
| PLD1 | 0.444830358 | GeneCards |
| RB1 | 0.444830358 | GeneCards |
| GLS | 0.444830358 | GeneCards |
| PRF1 | 0.444830358 | GeneCards |
| PDE4C | 0.444830358 | GeneCards |
| KLK1 | 0.444830358 | GeneCards |
| MSLN | 0.444830358 | GeneCards |
| RGS11 | 0.444830358 | GeneCards |
| CNKSR3 | 0.444830358 | GeneCards |
| ADAM10 | 0.429302216 | GeneCards |
| ESR2 | 0.429302216 | GeneCards |
| YES1 | 0.429302216 | GeneCards |
| FPR1 | 0.429302216 | GeneCards |
| HMOX2 | 0.429302216 | GeneCards |
| DLC1 | 0.429302216 | GeneCards |
| OXTR | 0.429302216 | GeneCards |
| BRD4 | 0.429302216 | GeneCards |
| PDIA3 | 0.429302216 | GeneCards |
| SMYD3 | 0.429302216 | GeneCards |
| CACNA2D3 | 0.429302216 | GeneCards |
| ANK2 | 0.429302216 | GeneCards |
| AMBN | 0.429302216 | GeneCards |
| ST13 | 0.429302216 | GeneCards |
| UPK3A | 0.429302216 | GeneCards |
| TSHZ3 | 0.429302216 | GeneCards |
| PRDM12 | 0.429302216 | GeneCards |
| PAPPA2 | 0.429302216 | GeneCards |
| LRTM1 | 0.429302216 | GeneCards |
| LYRM9 | 0.429302216 | GeneCards |
| LINC00426 | 0.429302216 | GeneCards |
| LINC00882 | 0.429302216 | GeneCards |
| NETO1-DT | 0.429302216 | GeneCards |
| PIK3CA | 0.415048718 | GeneCards |
| CACNA1H | 0.415048718 | GeneCards |
| IFNAR2 | 0.415048718 | GeneCards |
| UBE2I | 0.415048718 | GeneCards |
| AXIN1 | 0.415048718 | GeneCards |
| IRF9 | 0.415048718 | GeneCards |
| SPHK2 | 0.415048718 | GeneCards |
| PIGQ | 0.415048718 | GeneCards |
| NME3 | 0.415048718 | GeneCards |
| HAGH | 0.415048718 | GeneCards |
| HBA1 | 0.415048718 | GeneCards |
| HBA2 | 0.415048718 | GeneCards |
| PLA2G2D | 0.415048718 | GeneCards |
| NME4 | 0.415048718 | GeneCards |
| CHN2 | 0.415048718 | GeneCards |
| SSTR5 | 0.415048718 | GeneCards |
| PDE8A | 0.415048718 | GeneCards |
| MPG | 0.415048718 | GeneCards |
| IL27RA | 0.415048718 | GeneCards |
| MAPK8IP3 | 0.415048718 | GeneCards |
| MSRB1 | 0.415048718 | GeneCards |
| ARFGEF1 | 0.415048718 | GeneCards |
| DECR2 | 0.415048718 | GeneCards |
| HBZ | 0.415048718 | GeneCards |
| IFT140 | 0.415048718 | GeneCards |
| CXCL16 | 0.415048718 | GeneCards |
| CARD10 | 0.415048718 | GeneCards |
| ARHGEF5 | 0.415048718 | GeneCards |
| YBX1 | 0.415048718 | GeneCards |
| TELO2 | 0.415048718 | GeneCards |
| POLR3K | 0.415048718 | GeneCards |
| RHOT2 | 0.415048718 | GeneCards |
| ARHGDIG | 0.415048718 | GeneCards |
| GNLY | 0.415048718 | GeneCards |
| CXCL14 | 0.415048718 | GeneCards |
| BAIAP3 | 0.415048718 | GeneCards |
| PDIA2 | 0.415048718 | GeneCards |
| RPL3L | 0.415048718 | GeneCards |
| CCL16 | 0.415048718 | GeneCards |
| LUC7L | 0.415048718 | GeneCards |
| FAHD1 | 0.415048718 | GeneCards |
| SOX8 | 0.415048718 | GeneCards |
| RHBDF1 | 0.415048718 | GeneCards |
| MRPL28 | 0.415048718 | GeneCards |
| IFNA6 | 0.415048718 | GeneCards |
| GNG13 | 0.415048718 | GeneCards |
| RAB11FIP3 | 0.415048718 | GeneCards |
| ART1 | 0.415048718 | GeneCards |
| CCDC78 | 0.415048718 | GeneCards |
| HBQ1 | 0.415048718 | GeneCards |
| CCL14 | 0.415048718 | GeneCards |
| RHBDL1 | 0.415048718 | GeneCards |
| UNKL | 0.415048718 | GeneCards |
| IFNA8 | 0.415048718 | GeneCards |
| CNOT6L | 0.415048718 | GeneCards |
| IFNA13 | 0.415048718 | GeneCards |
| CPQ | 0.415048718 | GeneCards |
| CAPN15 | 0.415048718 | GeneCards |
| EME2 | 0.415048718 | GeneCards |
| MEIOB | 0.415048718 | GeneCards |
| WFIKKN1 | 0.415048718 | GeneCards |
| RAB40C | 0.415048718 | GeneCards |
| HAGHL | 0.415048718 | GeneCards |
| TMEM204 | 0.415048718 | GeneCards |
| SPSB3 | 0.415048718 | GeneCards |
| METRN | 0.415048718 | GeneCards |
| RPUSD1 | 0.415048718 | GeneCards |
| WDR24 | 0.415048718 | GeneCards |
| TSR3 | 0.415048718 | GeneCards |
| JMJD8 | 0.415048718 | GeneCards |
| C16orf91 | 0.415048718 | GeneCards |
| C9orf135 | 0.415048718 | GeneCards |
| PTX4 | 0.415048718 | GeneCards |
| CIAO3 | 0.415048718 | GeneCards |
| FAM234A | 0.415048718 | GeneCards |
| CRAMP1 | 0.415048718 | GeneCards |
| JPT2 | 0.415048718 | GeneCards |
| PGAP6 | 0.415048718 | GeneCards |
| ANTKMT | 0.415048718 | GeneCards |
| METTL26 | 0.415048718 | GeneCards |
| MSLNL | 0.415048718 | GeneCards |
| WASIR2 | 0.415048718 | GeneCards |
| LINC02890 | 0.415048718 | GeneCards |
| BRAF | 0.409371257 | GeneCards |
| MET | 0.409371257 | GeneCards |
| RET | 0.409371257 | GeneCards |
| CDC42 | 0.409371257 | GeneCards |
| CD19 | 0.409371257 | GeneCards |
| ACTB | 0.409371257 | GeneCards |
| WAS | 0.409371257 | GeneCards |
| SLC6A3 | 0.409371257 | GeneCards |
| GLRB | 0.409371257 | GeneCards |
| HTR2C | 0.409371257 | GeneCards |
| HTR1A | 0.409371257 | GeneCards |
| MAT2A | 0.409371257 | GeneCards |
| TPH2 | 0.409371257 | GeneCards |
| COL3A1 | 0.409371257 | GeneCards |
| AVPR1A | 0.409371257 | GeneCards |
| CCKAR | 0.409371257 | GeneCards |
| HCFC1 | 0.409371257 | GeneCards |
| SCN10A | 0.409371257 | GeneCards |
| GRM7 | 0.409371257 | GeneCards |
| ABAT | 0.409371257 | GeneCards |
| FUS | 0.409371257 | GeneCards |
| FARSB | 0.409371257 | GeneCards |
| GABRA3 | 0.409371257 | GeneCards |
| HTR1B | 0.409371257 | GeneCards |
| KRT16 | 0.409371257 | GeneCards |
| DRD1 | 0.409371257 | GeneCards |
| FGF14 | 0.409371257 | GeneCards |
| RNASEL | 0.409371257 | GeneCards |
| TMPO | 0.409371257 | GeneCards |
| TOR1A | 0.409371257 | GeneCards |
| HLCS | 0.409371257 | GeneCards |
| GREM1 | 0.409371257 | GeneCards |
| MBTPS1 | 0.409371257 | GeneCards |
| FIG4 | 0.409371257 | GeneCards |
| ASIC1 | 0.409371257 | GeneCards |
| TRH | 0.409371257 | GeneCards |
| MSMO1 | 0.409371257 | GeneCards |
| MYBPC1 | 0.409371257 | GeneCards |
| COPA | 0.409371257 | GeneCards |
| GDF15 | 0.409371257 | GeneCards |
| RECQL4 | 0.409371257 | GeneCards |
| NPY5R | 0.409371257 | GeneCards |
| VPS37A | 0.409371257 | GeneCards |
| SCN11A | 0.409371257 | GeneCards |
| CPA4 | 0.409371257 | GeneCards |
| CD5 | 0.409371257 | GeneCards |
| LTBP4 | 0.409371257 | GeneCards |
| ASIC2 | 0.409371257 | GeneCards |
| ZMYM2 | 0.409371257 | GeneCards |
| PLRG1 | 0.409371257 | GeneCards |
| SENP8 | 0.409371257 | GeneCards |
| LAPTM4A | 0.409371257 | GeneCards |
| LCOR | 0.409371257 | GeneCards |
| FAF2 | 0.409371257 | GeneCards |
| DNAJC13 | 0.409371257 | GeneCards |
| EML4 | 0.409371257 | GeneCards |
| PRPF19 | 0.409371257 | GeneCards |
| ANO2 | 0.409371257 | GeneCards |
| BCL7B | 0.409371257 | GeneCards |
| GZF1 | 0.409371257 | GeneCards |
| FAM111B | 0.409371257 | GeneCards |
| KDM1B | 0.409371257 | GeneCards |
| ZMYM3 | 0.409371257 | GeneCards |
| EVPL | 0.409371257 | GeneCards |
| EPPIN | 0.409371257 | GeneCards |
| EPC2 | 0.409371257 | GeneCards |
| TUT1 | 0.409371257 | GeneCards |
| NARS1 | 0.409371257 | GeneCards |
| AMY1A | 0.409371257 | GeneCards |
| CLEC5A | 0.409371257 | GeneCards |
| TENM4 | 0.409371257 | GeneCards |
| PLEKHG1 | 0.409371257 | GeneCards |
| DCAF11 | 0.409371257 | GeneCards |
| ERVW-1 | 0.409371257 | GeneCards |
| DAOA | 0.409371257 | GeneCards |
| ANKAR | 0.409371257 | GeneCards |
| GGT2 | 0.409371257 | GeneCards |
| NSMCE3 | 0.409371257 | GeneCards |
| EEF2KMT | 0.409371257 | GeneCards |
| EEF1AKMT1 | 0.409371257 | GeneCards |
| NEMP2 | 0.409371257 | GeneCards |
| MIR141 | 0.409371257 | GeneCards |
| MIR150 | 0.409371257 | GeneCards |
| MIR27A | 0.409371257 | GeneCards |
| MIR34A | 0.409371257 | GeneCards |
| MIR122 | 0.409371257 | GeneCards |
| GGTLC3 | 0.409371257 | GeneCards |
| MIRLET7D | 0.409371257 | GeneCards |
| MIR127 | 0.409371257 | GeneCards |
| MIR373 | 0.409371257 | GeneCards |
| MIR182 | 0.409371257 | GeneCards |
| MIR214 | 0.409371257 | GeneCards |
| MIR29B1 | 0.409371257 | GeneCards |
| MIR93 | 0.409371257 | GeneCards |
| MIRLET7E | 0.409371257 | GeneCards |
| RNU4ATAC | 0.409371257 | GeneCards |
| MIR146B | 0.409371257 | GeneCards |
| MIR342 | 0.409371257 | GeneCards |
| MIR23B | 0.409371257 | GeneCards |
| DAOA-AS1 | 0.409371257 | GeneCards |
| MIR92A1 | 0.409371257 | GeneCards |
| MIRLET7G | 0.409371257 | GeneCards |
| MIR151A | 0.409371257 | GeneCards |
| MIR193A | 0.409371257 | GeneCards |
| MIR455 | 0.409371257 | GeneCards |
| MIR106A | 0.409371257 | GeneCards |
| MIR18A | 0.409371257 | GeneCards |
| MIR19B1 | 0.409371257 | GeneCards |
| MIR335 | 0.409371257 | GeneCards |
| RNU6-1 | 0.409371257 | GeneCards |
| MIR144 | 0.409371257 | GeneCards |
| RNU5A-1 | 0.409371257 | GeneCards |
| MIR424 | 0.409371257 | GeneCards |
| MIR488 | 0.409371257 | GeneCards |
| RNU6ATAC | 0.409371257 | GeneCards |
| MIR3614 | 0.409371257 | GeneCards |
| NOTCH2NLC | 0.409371257 | GeneCards |
| LINC01136 | 0.409371257 | GeneCards |
| TMPO-AS1 | 0.409371257 | GeneCards |
| BANCR | 0.409371257 | GeneCards |
| TGFB2-AS1 | 0.409371257 | GeneCards |
| TGFB2-OT1 | 0.409371257 | GeneCards |
| LOC101448202 | 0.409371257 | GeneCards |
| ENSG00000259039 | 0.409371257 | GeneCards |
| ETM2 | 0.409371257 | GeneCards |
| LOC106627981 | 0.409371257 | GeneCards |
| PAND1 | 0.409371257 | GeneCards |
| LOC113939944 | 0.409371257 | GeneCards |
| PAND3 | 0.409371257 | GeneCards |
| PAND2 | 0.409371257 | GeneCards |
| STIM1 | 0.40930444 | GeneCards |
| WNT3A | 0.40930444 | GeneCards |
| CD46 | 0.40930444 | GeneCards |
| ABCC4 | 0.40930444 | GeneCards |
| HBEGF | 0.40930444 | GeneCards |
| SLCO2B1 | 0.40930444 | GeneCards |
| CADM1 | 0.40930444 | GeneCards |
| CTF1 | 0.40930444 | GeneCards |
| FRMD4A | 0.403742045 | GeneCards |
| CRYGN | 0.403742045 | GeneCards |
| CDK4 | 0.402495056 | GeneCards |
| IDH1 | 0.402495056 | GeneCards |
| DNM1 | 0.402495056 | GeneCards |
| DYRK1A | 0.402495056 | GeneCards |
| ACTN1 | 0.402495056 | GeneCards |
| MERTK | 0.402495056 | GeneCards |
| PRKCH | 0.402495056 | GeneCards |
| RPS6KA1 | 0.402495056 | GeneCards |
| SNAP25 | 0.402495056 | GeneCards |
| GLI3 | 0.402495056 | GeneCards |
| CYP17A1 | 0.402495056 | GeneCards |
| BLM | 0.402495056 | GeneCards |
| EPHA4 | 0.402495056 | GeneCards |
| EPAS1 | 0.402495056 | GeneCards |
| STK11 | 0.402495056 | GeneCards |
| PIK3C3 | 0.402495056 | GeneCards |
| PSEN2 | 0.402495056 | GeneCards |
| PBX1 | 0.402495056 | GeneCards |
| EPHA7 | 0.402495056 | GeneCards |
| GDNF | 0.402495056 | GeneCards |
| LPIN1 | 0.402495056 | GeneCards |
| IKZF1 | 0.402495056 | GeneCards |
| MAP3K3 | 0.402495056 | GeneCards |
| GFAP | 0.402495056 | GeneCards |
| FES | 0.402495056 | GeneCards |
| APRT | 0.402495056 | GeneCards |
| ATP1B1 | 0.402495056 | GeneCards |
| ALDH7A1 | 0.402495056 | GeneCards |
| MECOM | 0.402495056 | GeneCards |
| TEAD1 | 0.402495056 | GeneCards |
| PIKFYVE | 0.402495056 | GeneCards |
| PDE6A | 0.402495056 | GeneCards |
| PTPN12 | 0.402495056 | GeneCards |
| PAX3 | 0.402495056 | GeneCards |
| RPL5 | 0.402495056 | GeneCards |
| KCNJ6 | 0.402495056 | GeneCards |
| CHRNA2 | 0.402495056 | GeneCards |
| KYNU | 0.402495056 | GeneCards |
| MAP2K4 | 0.402495056 | GeneCards |
| GPD2 | 0.402495056 | GeneCards |
| GRM3 | 0.402495056 | GeneCards |
| HSD17B4 | 0.402495056 | GeneCards |
| ITGB6 | 0.402495056 | GeneCards |
| MAP3K8 | 0.402495056 | GeneCards |
| FER | 0.402495056 | GeneCards |
| DDX5 | 0.402495056 | GeneCards |
| ETV6 | 0.402495056 | GeneCards |
| CDK9 | 0.402495056 | GeneCards |
| CTSF | 0.402495056 | GeneCards |
| CUL3 | 0.402495056 | GeneCards |
| ACVR2A | 0.402495056 | GeneCards |
| CACNA2D1 | 0.402495056 | GeneCards |
| C2 | 0.402495056 | GeneCards |
| EIF4G1 | 0.402495056 | GeneCards |
| EPHA3 | 0.402495056 | GeneCards |
| UBE2D3 | 0.402495056 | GeneCards |
| TWIST1 | 0.402495056 | GeneCards |
| TYRP1 | 0.402495056 | GeneCards |
| ZBTB16 | 0.402495056 | GeneCards |
| PSMB7 | 0.402495056 | GeneCards |
| SLC27A4 | 0.402495056 | GeneCards |
| PGD | 0.402495056 | GeneCards |
| PCYT1A | 0.402495056 | GeneCards |
| NCOA3 | 0.402495056 | GeneCards |
| PAK2 | 0.402495056 | GeneCards |
| MDH1 | 0.402495056 | GeneCards |
| LIG1 | 0.402495056 | GeneCards |
| AP3B1 | 0.402495056 | GeneCards |
| FOXP1 | 0.402495056 | GeneCards |
| INPP5D | 0.402495056 | GeneCards |
| IKBKE | 0.402495056 | GeneCards |
| MAP3K12 | 0.402495056 | GeneCards |
| DGKB | 0.402495056 | GeneCards |
| ERCC4 | 0.402495056 | GeneCards |
| CKB | 0.402495056 | GeneCards |
| CYC1 | 0.402495056 | GeneCards |
| CFB | 0.402495056 | GeneCards |
| BCAT2 | 0.402495056 | GeneCards |
| TRPS1 | 0.402495056 | GeneCards |
| TCF3 | 0.402495056 | GeneCards |
| NUP62 | 0.402495056 | GeneCards |
| THBS2 | 0.402495056 | GeneCards |
| TNK2 | 0.402495056 | GeneCards |
| PNKP | 0.402495056 | GeneCards |
| PKN2 | 0.402495056 | GeneCards |
| SHMT2 | 0.402495056 | GeneCards |
| DPM1 | 0.402495056 | GeneCards |
| KLF5 | 0.402495056 | GeneCards |
| GGPS1 | 0.402495056 | GeneCards |
| GRM4 | 0.402495056 | GeneCards |
| MAPK4 | 0.402495056 | GeneCards |
| LITAF | 0.402495056 | GeneCards |
| NDUFA6 | 0.402495056 | GeneCards |
| CBX5 | 0.402495056 | GeneCards |
| EPHB3 | 0.402495056 | GeneCards |
| DFFA | 0.402495056 | GeneCards |
| EZH1 | 0.402495056 | GeneCards |
| CLDN14 | 0.402495056 | GeneCards |
| ARID1A | 0.402495056 | GeneCards |
| CHRNB2 | 0.402495056 | GeneCards |
| DOT1L | 0.402495056 | GeneCards |
| TFG | 0.402495056 | GeneCards |
| PRMT7 | 0.402495056 | GeneCards |
| SLC7A5 | 0.402495056 | GeneCards |
| SKI | 0.402495056 | GeneCards |
| SLC12A4 | 0.402495056 | GeneCards |
| RUVBL2 | 0.402495056 | GeneCards |
| PDGFD | 0.402495056 | GeneCards |
| PYGB | 0.402495056 | GeneCards |
| RDH12 | 0.402495056 | GeneCards |
| PTH2R | 0.402495056 | GeneCards |
| RPL18 | 0.402495056 | GeneCards |
| S100A10 | 0.402495056 | GeneCards |
| RBBP8 | 0.402495056 | GeneCards |
| SLC34A1 | 0.402495056 | GeneCards |
| FARS2 | 0.402495056 | GeneCards |
| COLEC11 | 0.402495056 | GeneCards |
| ATP6V1E1 | 0.402495056 | GeneCards |
| CCS | 0.402495056 | GeneCards |
| CA5A | 0.402495056 | GeneCards |
| MAP4K1 | 0.402495056 | GeneCards |
| HSD17B1 | 0.402495056 | GeneCards |
| LAMA2 | 0.402495056 | GeneCards |
| LIPG | 0.402495056 | GeneCards |
| HNF4G | 0.402495056 | GeneCards |
| NFATC3 | 0.402495056 | GeneCards |
| CACNB1 | 0.402495056 | GeneCards |
| FLRT3 | 0.402495056 | GeneCards |
| GFRA2 | 0.402495056 | GeneCards |
| FGF9 | 0.402495056 | GeneCards |
| DYSF | 0.402495056 | GeneCards |
| EFNA5 | 0.402495056 | GeneCards |
| CRADD | 0.402495056 | GeneCards |
| CKMT2 | 0.402495056 | GeneCards |
| ACD | 0.402495056 | GeneCards |
| A4GALT | 0.402495056 | GeneCards |
| ABCA7 | 0.402495056 | GeneCards |
| ARHGEF18 | 0.402495056 | GeneCards |
| BCL11A | 0.402495056 | GeneCards |
| EIF2B1 | 0.402495056 | GeneCards |
| MYO9B | 0.402495056 | GeneCards |
| UBTF | 0.402495056 | GeneCards |
| TRAP1 | 0.402495056 | GeneCards |
| TRPM1 | 0.402495056 | GeneCards |
| WNK4 | 0.402495056 | GeneCards |
| SPG7 | 0.402495056 | GeneCards |
| SOX4 | 0.402495056 | GeneCards |
| PARVA | 0.402495056 | GeneCards |
| POLR2E | 0.402495056 | GeneCards |
| PLAGL1 | 0.402495056 | GeneCards |
| PLK2 | 0.402495056 | GeneCards |
| PPOX | 0.402495056 | GeneCards |
| PREX1 | 0.402495056 | GeneCards |
| PROK2 | 0.402495056 | GeneCards |
| SLC18A3 | 0.402495056 | GeneCards |
| SLC7A6 | 0.402495056 | GeneCards |
| SLC29A3 | 0.402495056 | GeneCards |
| SLC16A7 | 0.402495056 | GeneCards |
| SLC39A8 | 0.402495056 | GeneCards |
| NRIP1 | 0.402495056 | GeneCards |
| NRP2 | 0.402495056 | GeneCards |
| P2RX4 | 0.402495056 | GeneCards |
| MUS81 | 0.402495056 | GeneCards |
| MLF1 | 0.402495056 | GeneCards |
| QKI | 0.402495056 | GeneCards |
| RELN | 0.402495056 | GeneCards |
| RGS14 | 0.402495056 | GeneCards |
| SCN2B | 0.402495056 | GeneCards |
| MSRA | 0.402495056 | GeneCards |
| NR1D2 | 0.402495056 | GeneCards |
| PTTG1 | 0.402495056 | GeneCards |
| ARHGEF3 | 0.402495056 | GeneCards |
| ADAM19 | 0.402495056 | GeneCards |
| FRS2 | 0.402495056 | GeneCards |
| CYTH1 | 0.402495056 | GeneCards |
| ADNP | 0.402495056 | GeneCards |
| ATP6V0A1 | 0.402495056 | GeneCards |
| ITGA8 | 0.402495056 | GeneCards |
| KLC2 | 0.402495056 | GeneCards |
| KRT7 | 0.402495056 | GeneCards |
| MBD4 | 0.402495056 | GeneCards |
| HAPLN1 | 0.402495056 | GeneCards |
| KIF1A | 0.402495056 | GeneCards |
| MANBA | 0.402495056 | GeneCards |
| MAP1LC3B | 0.402495056 | GeneCards |
| FBXO11 | 0.402495056 | GeneCards |
| GPAA1 | 0.402495056 | GeneCards |
| EEF1D | 0.402495056 | GeneCards |
| CPT1C | 0.402495056 | GeneCards |
| DCTD | 0.402495056 | GeneCards |
| AIPL1 | 0.402495056 | GeneCards |
| ACSF3 | 0.402495056 | GeneCards |
| BANF1 | 0.402495056 | GeneCards |
| BRD7 | 0.402495056 | GeneCards |
| CDCA7 | 0.402495056 | GeneCards |
| CDK5RAP2 | 0.402495056 | GeneCards |
| ENTPD6 | 0.402495056 | GeneCards |
| EPM2A | 0.402495056 | GeneCards |
| CSNK1G3 | 0.402495056 | GeneCards |
| TMC6 | 0.402495056 | GeneCards |
| RNF168 | 0.402495056 | GeneCards |
| USP25 | 0.402495056 | GeneCards |
| UVRAG | 0.402495056 | GeneCards |
| TAF4B | 0.402495056 | GeneCards |
| TARS2 | 0.402495056 | GeneCards |
| TFEB | 0.402495056 | GeneCards |
| THY1 | 0.402495056 | GeneCards |
| SPRY1 | 0.402495056 | GeneCards |
| PREP | 0.402495056 | GeneCards |
| PRPF6 | 0.402495056 | GeneCards |
| PRMT3 | 0.402495056 | GeneCards |
| SLC6A13 | 0.402495056 | GeneCards |
| SARDH | 0.402495056 | GeneCards |
| SMARCD2 | 0.402495056 | GeneCards |
| NTSR2 | 0.402495056 | GeneCards |
| PIGV | 0.402495056 | GeneCards |
| OCA2 | 0.402495056 | GeneCards |
| RAB1B | 0.402495056 | GeneCards |
| RPL13A | 0.402495056 | GeneCards |
| SGCG | 0.402495056 | GeneCards |
| SEC31A | 0.402495056 | GeneCards |
| NFASC | 0.402495056 | GeneCards |
| MYL6 | 0.402495056 | GeneCards |
| HNRNPU | 0.402495056 | GeneCards |
| LGMN | 0.402495056 | GeneCards |
| PTBP1 | 0.402495056 | GeneCards |
| SMUG1 | 0.402495056 | GeneCards |
| SMURF2 | 0.402495056 | GeneCards |
| S1PR4 | 0.402495056 | GeneCards |
| GALNT12 | 0.402495056 | GeneCards |
| COL12A1 | 0.402495056 | GeneCards |
| CYTH2 | 0.402495056 | GeneCards |
| DPF2 | 0.402495056 | GeneCards |
| HIBADH | 0.402495056 | GeneCards |
| IL17B | 0.402495056 | GeneCards |
| CD37 | 0.402495056 | GeneCards |
| CISD2 | 0.402495056 | GeneCards |
| CSGALNACT2 | 0.402495056 | GeneCards |
| ALG2 | 0.402495056 | GeneCards |
| CACNG3 | 0.402495056 | GeneCards |
| ARCN1 | 0.402495056 | GeneCards |
| CEP57 | 0.402495056 | GeneCards |
| CHRD | 0.402495056 | GeneCards |
| RNF14 | 0.402495056 | GeneCards |
| UQCRQ | 0.402495056 | GeneCards |
| TRIM27 | 0.402495056 | GeneCards |
| POLRMT | 0.402495056 | GeneCards |
| POLG2 | 0.402495056 | GeneCards |
| PLEKHM1 | 0.402495056 | GeneCards |
| NPEPPS | 0.402495056 | GeneCards |
| RPL8 | 0.402495056 | GeneCards |
| MSRB2 | 0.402495056 | GeneCards |
| RAB5C | 0.402495056 | GeneCards |
| PABPC4 | 0.402495056 | GeneCards |
| TOP1MT | 0.402495056 | GeneCards |
| APOBEC3B | 0.402495056 | GeneCards |
| FLVCR1 | 0.402495056 | GeneCards |
| DOCK3 | 0.402495056 | GeneCards |
| DPH1 | 0.402495056 | GeneCards |
| BCL3 | 0.402495056 | GeneCards |
| AS3MT | 0.402495056 | GeneCards |
| ASAP1 | 0.402495056 | GeneCards |
| MAGI1 | 0.402495056 | GeneCards |
| HERPUD1 | 0.402495056 | GeneCards |
| MBD1 | 0.402495056 | GeneCards |
| GTPBP3 | 0.402495056 | GeneCards |
| HTATIP2 | 0.402495056 | GeneCards |
| GRID1 | 0.402495056 | GeneCards |
| HSD17B6 | 0.402495056 | GeneCards |
| EIF3H | 0.402495056 | GeneCards |
| FIBP | 0.402495056 | GeneCards |
| GDF11 | 0.402495056 | GeneCards |
| GDI2 | 0.402495056 | GeneCards |
| GFI1 | 0.402495056 | GeneCards |
| EPS15L1 | 0.402495056 | GeneCards |
| CLIC4 | 0.402495056 | GeneCards |
| CNTN6 | 0.402495056 | GeneCards |
| BCLAF1 | 0.402495056 | GeneCards |
| DKK3 | 0.402495056 | GeneCards |
| CSRP2 | 0.402495056 | GeneCards |
| ACOT7 | 0.402495056 | GeneCards |
| ACTR1A | 0.402495056 | GeneCards |
| ANKH | 0.402495056 | GeneCards |
| ADI1 | 0.402495056 | GeneCards |
| AKAP6 | 0.402495056 | GeneCards |
| CADM3 | 0.402495056 | GeneCards |
| B3GALT5 | 0.402495056 | GeneCards |
| BRD1 | 0.402495056 | GeneCards |
| CDH17 | 0.402495056 | GeneCards |
| CDC42BPB | 0.402495056 | GeneCards |
| EEF1G | 0.402495056 | GeneCards |
| E2F6 | 0.402495056 | GeneCards |
| EFHC1 | 0.402495056 | GeneCards |
| OS9 | 0.402495056 | GeneCards |
| SYNGR1 | 0.402495056 | GeneCards |
| RAPGEF5 | 0.402495056 | GeneCards |
| TRIM63 | 0.402495056 | GeneCards |
| TSEN2 | 0.402495056 | GeneCards |
| THG1L | 0.402495056 | GeneCards |
| SUPT3H | 0.402495056 | GeneCards |
| TRAPPC4 | 0.402495056 | GeneCards |
| SULF2 | 0.402495056 | GeneCards |
| PLXND1 | 0.402495056 | GeneCards |
| POLA2 | 0.402495056 | GeneCards |
| POLR2H | 0.402495056 | GeneCards |
| MRPS28 | 0.402495056 | GeneCards |
| SMOC1 | 0.402495056 | GeneCards |
| RGS19 | 0.402495056 | GeneCards |
| SLC6A15 | 0.402495056 | GeneCards |
| RYBP | 0.402495056 | GeneCards |
| SHARPIN | 0.402495056 | GeneCards |
| SLC4A11 | 0.402495056 | GeneCards |
| SMARCA5 | 0.402495056 | GeneCards |
| NUBP1 | 0.402495056 | GeneCards |
| PIGN | 0.402495056 | GeneCards |
| POGLUT1 | 0.402495056 | GeneCards |
| RAB8A | 0.402495056 | GeneCards |
| RPS6KC1 | 0.402495056 | GeneCards |
| PPP1R15A | 0.402495056 | GeneCards |
| SH3BP4 | 0.402495056 | GeneCards |
| NR2F6 | 0.402495056 | GeneCards |
| LIN28A | 0.402495056 | GeneCards |
| KLF13 | 0.402495056 | GeneCards |
| RASSF5 | 0.402495056 | GeneCards |
| RARRES1 | 0.402495056 | GeneCards |
| RBM4 | 0.402495056 | GeneCards |
| TMPRSS15 | 0.402495056 | GeneCards |
| AP3D1 | 0.402495056 | GeneCards |
| ACMSD | 0.402495056 | GeneCards |
| ACSL6 | 0.402495056 | GeneCards |
| FARP2 | 0.402495056 | GeneCards |
| FPR3 | 0.402495056 | GeneCards |
| CYTH3 | 0.402495056 | GeneCards |
| ATP8B2 | 0.402495056 | GeneCards |
| CD200R1 | 0.402495056 | GeneCards |
| GRIN3B | 0.402495056 | GeneCards |
| IP6K3 | 0.402495056 | GeneCards |
| KRT81 | 0.402495056 | GeneCards |
| MCM9 | 0.402495056 | GeneCards |
| MED25 | 0.402495056 | GeneCards |
| IFT122 | 0.402495056 | GeneCards |
| ID4 | 0.402495056 | GeneCards |
| HOXC4 | 0.402495056 | GeneCards |
| KCNN1 | 0.402495056 | GeneCards |
| KDELR1 | 0.402495056 | GeneCards |
| MAF1 | 0.402495056 | GeneCards |
| GIGYF2 | 0.402495056 | GeneCards |
| FAU | 0.402495056 | GeneCards |
| FGF20 | 0.402495056 | GeneCards |
| GLRX5 | 0.402495056 | GeneCards |
| GDF9 | 0.402495056 | GeneCards |
| FGL2 | 0.402495056 | GeneCards |
| EXOC2 | 0.402495056 | GeneCards |
| BPTF | 0.402495056 | GeneCards |
| CFHR5 | 0.402495056 | GeneCards |
| CNTN4 | 0.402495056 | GeneCards |
| CPSF1 | 0.402495056 | GeneCards |
| CST6 | 0.402495056 | GeneCards |
| AKT1S1 | 0.402495056 | GeneCards |
| BMPER | 0.402495056 | GeneCards |
| ANKS1B | 0.402495056 | GeneCards |
| AP2A1 | 0.402495056 | GeneCards |
| ASF1A | 0.402495056 | GeneCards |
| ARHGAP15 | 0.402495056 | GeneCards |
| ARHGAP24 | 0.402495056 | GeneCards |
| CDCA2 | 0.402495056 | GeneCards |
| CDK17 | 0.402495056 | GeneCards |
| DUSP22 | 0.402495056 | GeneCards |
| ZNF202 | 0.402495056 | GeneCards |
| UBASH3A | 0.402495056 | GeneCards |
| NBAS | 0.402495056 | GeneCards |
| NCAM2 | 0.402495056 | GeneCards |
| TMC8 | 0.402495056 | GeneCards |
| ME3 | 0.402495056 | GeneCards |
| VPS28 | 0.402495056 | GeneCards |
| UTRN | 0.402495056 | GeneCards |
| ZBTB18 | 0.402495056 | GeneCards |
| TIAM2 | 0.402495056 | GeneCards |
| TOMM20 | 0.402495056 | GeneCards |
| SRSF5 | 0.402495056 | GeneCards |
| PARVB | 0.402495056 | GeneCards |
| PLXNC1 | 0.402495056 | GeneCards |
| SLC9A9 | 0.402495056 | GeneCards |
| SLCO1A2 | 0.402495056 | GeneCards |
| RIN1 | 0.402495056 | GeneCards |
| SF3B2 | 0.402495056 | GeneCards |
| SLC15A2 | 0.402495056 | GeneCards |
| SLC25A39 | 0.402495056 | GeneCards |
| NUCB1 | 0.402495056 | GeneCards |
| OAS2 | 0.402495056 | GeneCards |
| PDLIM4 | 0.402495056 | GeneCards |
| NPTXR | 0.402495056 | GeneCards |
| MYL6B | 0.402495056 | GeneCards |
| MLLT3 | 0.402495056 | GeneCards |
| MLANA | 0.402495056 | GeneCards |
| RRAGA | 0.402495056 | GeneCards |
| RPS11 | 0.402495056 | GeneCards |
| RPL18A | 0.402495056 | GeneCards |
| SEMA6D | 0.402495056 | GeneCards |
| SESN1 | 0.402495056 | GeneCards |
| SESN3 | 0.402495056 | GeneCards |
| SEMA6A | 0.402495056 | GeneCards |
| PSMD6 | 0.402495056 | GeneCards |
| MSI1 | 0.402495056 | GeneCards |
| MYO1A | 0.402495056 | GeneCards |
| KRT86 | 0.402495056 | GeneCards |
| RAD54L2 | 0.402495056 | GeneCards |
| ARL4A | 0.402495056 | GeneCards |
| COX7A2 | 0.402495056 | GeneCards |
| CYTH4 | 0.402495056 | GeneCards |
| CD101 | 0.402495056 | GeneCards |
| CD248 | 0.402495056 | GeneCards |
| INPP4B | 0.402495056 | GeneCards |
| KLF12 | 0.402495056 | GeneCards |
| KLHL1 | 0.402495056 | GeneCards |
| LEMD2 | 0.402495056 | GeneCards |
| KHDRBS3 | 0.402495056 | GeneCards |
| LHPP | 0.402495056 | GeneCards |
| LMBRD1 | 0.402495056 | GeneCards |
| ITSN2 | 0.402495056 | GeneCards |
| KANSL1 | 0.402495056 | GeneCards |
| HMGN2 | 0.402495056 | GeneCards |
| GPR158 | 0.402495056 | GeneCards |
| EXOC6 | 0.402495056 | GeneCards |
| ASAP2 | 0.402495056 | GeneCards |
| CNKSR1 | 0.402495056 | GeneCards |
| CLSTN2 | 0.402495056 | GeneCards |
| BDH2 | 0.402495056 | GeneCards |
| DHX58 | 0.402495056 | GeneCards |
| CCNT2 | 0.402495056 | GeneCards |
| DNAJC7 | 0.402495056 | GeneCards |
| EDC4 | 0.402495056 | GeneCards |
| ZNF175 | 0.402495056 | GeneCards |
| UBE2Q1 | 0.402495056 | GeneCards |
| TRIM38 | 0.402495056 | GeneCards |
| SUMO4 | 0.402495056 | GeneCards |
| PRICKLE2 | 0.402495056 | GeneCards |
| PRSS23 | 0.402495056 | GeneCards |
| SMOX | 0.402495056 | GeneCards |
| PDE7B | 0.402495056 | GeneCards |
| PLCH1 | 0.402495056 | GeneCards |
| PGS1 | 0.402495056 | GeneCards |
| MKRN2 | 0.402495056 | GeneCards |
| PAPOLG | 0.402495056 | GeneCards |
| RCE1 | 0.402495056 | GeneCards |
| NKIRAS2 | 0.402495056 | GeneCards |
| MYOZ2 | 0.402495056 | GeneCards |
| RAPGEF6 | 0.402495056 | GeneCards |
| ARHGAP21 | 0.402495056 | GeneCards |
| COL15A1 | 0.402495056 | GeneCards |
| BNC2 | 0.402495056 | GeneCards |
| IMPG2 | 0.402495056 | GeneCards |
| ISCA1 | 0.402495056 | GeneCards |
| KRT83 | 0.402495056 | GeneCards |
| LCLAT1 | 0.402495056 | GeneCards |
| MICAL2 | 0.402495056 | GeneCards |
| GPX5 | 0.402495056 | GeneCards |
| HOXC6 | 0.402495056 | GeneCards |
| HOXC8 | 0.402495056 | GeneCards |
| INHBC | 0.402495056 | GeneCards |
| KCNH4 | 0.402495056 | GeneCards |
| KIF16B | 0.402495056 | GeneCards |
| LMAN2 | 0.402495056 | GeneCards |
| MED6 | 0.402495056 | GeneCards |
| MED7 | 0.402495056 | GeneCards |
| JAKMIP1 | 0.402495056 | GeneCards |
| HIVEP2 | 0.402495056 | GeneCards |
| NIT1 | 0.402495056 | GeneCards |
| CARD6 | 0.402495056 | GeneCards |
| CBLC | 0.402495056 | GeneCards |
| FBXO8 | 0.402495056 | GeneCards |
| FMNL2 | 0.402495056 | GeneCards |
| DAZAP1 | 0.402495056 | GeneCards |
| DCAF8 | 0.402495056 | GeneCards |
| DDX42 | 0.402495056 | GeneCards |
| EXOSC4 | 0.402495056 | GeneCards |
| EVI5 | 0.402495056 | GeneCards |
| EYA2 | 0.402495056 | GeneCards |
| BOLL | 0.402495056 | GeneCards |
| BRMS1 | 0.402495056 | GeneCards |
| CENPB | 0.402495056 | GeneCards |
| CENPT | 0.402495056 | GeneCards |
| CDK11A | 0.402495056 | GeneCards |
| CLYBL | 0.402495056 | GeneCards |
| CIZ1 | 0.402495056 | GeneCards |
| DIDO1 | 0.402495056 | GeneCards |
| DHX15 | 0.402495056 | GeneCards |
| ANKRD11 | 0.402495056 | GeneCards |
| AIG1 | 0.402495056 | GeneCards |
| ADAMTSL4 | 0.402495056 | GeneCards |
| BATF3 | 0.402495056 | GeneCards |
| BTN2A1 | 0.402495056 | GeneCards |
| ANKRD6 | 0.402495056 | GeneCards |
| AP2A2 | 0.402495056 | GeneCards |
| APOBEC3C | 0.402495056 | GeneCards |
| TSPAN31 | 0.402495056 | GeneCards |
| ZNF180 | 0.402495056 | GeneCards |
| ZNF224 | 0.402495056 | GeneCards |
| NPNT | 0.402495056 | GeneCards |
| NADK2 | 0.402495056 | GeneCards |
| TNFAIP8 | 0.402495056 | GeneCards |
| UGT3A1 | 0.402495056 | GeneCards |
| UNC5B | 0.402495056 | GeneCards |
| UCKL1 | 0.402495056 | GeneCards |
| USP36 | 0.402495056 | GeneCards |
| TRIB2 | 0.402495056 | GeneCards |
| ZFP64 | 0.402495056 | GeneCards |
| ZFPM1 | 0.402495056 | GeneCards |
| TIGIT | 0.402495056 | GeneCards |
| THAP11 | 0.402495056 | GeneCards |
| TMPRSS5 | 0.402495056 | GeneCards |
| TMEM30A | 0.402495056 | GeneCards |
| SPRR3 | 0.402495056 | GeneCards |
| SRRM1 | 0.402495056 | GeneCards |
| STAC | 0.402495056 | GeneCards |
| POLR3H | 0.402495056 | GeneCards |
| RD3 | 0.402495056 | GeneCards |
| RDH16 | 0.402495056 | GeneCards |
| SIN3B | 0.402495056 | GeneCards |
| RTN4RL1 | 0.402495056 | GeneCards |
| SENP5 | 0.402495056 | GeneCards |
| SH3PXD2A | 0.402495056 | GeneCards |
| SLU7 | 0.402495056 | GeneCards |
| NUF2 | 0.402495056 | GeneCards |
| PLCL2 | 0.402495056 | GeneCards |
| PDZD2 | 0.402495056 | GeneCards |
| PGLYRP4 | 0.402495056 | GeneCards |
| MUC20 | 0.402495056 | GeneCards |
| MYBPH | 0.402495056 | GeneCards |
| MYNN | 0.402495056 | GeneCards |
| RIN3 | 0.402495056 | GeneCards |
| RPL17 | 0.402495056 | GeneCards |
| SDR9C7 | 0.402495056 | GeneCards |
| SEMA6C | 0.402495056 | GeneCards |
| SCGN | 0.402495056 | GeneCards |
| MYLK4 | 0.402495056 | GeneCards |
| MYOM2 | 0.402495056 | GeneCards |
| RBFOX1 | 0.402495056 | GeneCards |
| RCAN3 | 0.402495056 | GeneCards |
| SNTB2 | 0.402495056 | GeneCards |
| YEATS4 | 0.402495056 | GeneCards |
| ARID4B | 0.402495056 | GeneCards |
| ATG2A | 0.402495056 | GeneCards |
| ATP10B | 0.402495056 | GeneCards |
| FOXB1 | 0.402495056 | GeneCards |
| FTSJ3 | 0.402495056 | GeneCards |
| COPS8 | 0.402495056 | GeneCards |
| COLGALT1 | 0.402495056 | GeneCards |
| DPT | 0.402495056 | GeneCards |
| DRAP1 | 0.402495056 | GeneCards |
| CD52 | 0.402495056 | GeneCards |
| GREB1 | 0.402495056 | GeneCards |
| LXN | 0.402495056 | GeneCards |
| HIVEP1 | 0.402495056 | GeneCards |
| HORMAD1 | 0.402495056 | GeneCards |
| MFSD1 | 0.402495056 | GeneCards |
| MBOAT1 | 0.402495056 | GeneCards |
| HEXIM2 | 0.402495056 | GeneCards |
| HOXC10 | 0.402495056 | GeneCards |
| HOXC9 | 0.402495056 | GeneCards |
| HS3ST3A1 | 0.402495056 | GeneCards |
| IFI44 | 0.402495056 | GeneCards |
| GRIN3A | 0.402495056 | GeneCards |
| KRT25 | 0.402495056 | GeneCards |
| L3MBTL3 | 0.402495056 | GeneCards |
| LUC7L2 | 0.402495056 | GeneCards |
| CASZ1 | 0.402495056 | GeneCards |
| EGFLAM | 0.402495056 | GeneCards |
| FBXO31 | 0.402495056 | GeneCards |
| FIGN | 0.402495056 | GeneCards |
| GNL1 | 0.402495056 | GeneCards |
| GPR182 | 0.402495056 | GeneCards |
| FAM126A | 0.402495056 | GeneCards |
| FAM172A | 0.402495056 | GeneCards |
| DUS2 | 0.402495056 | GeneCards |
| CNPY2 | 0.402495056 | GeneCards |
| CLSTN1 | 0.402495056 | GeneCards |
| CNTN5 | 0.402495056 | GeneCards |
| DKKL1 | 0.402495056 | GeneCards |
| DHDH | 0.402495056 | GeneCards |
| AHNAK | 0.402495056 | GeneCards |
| AKAP11 | 0.402495056 | GeneCards |
| ACER2 | 0.402495056 | GeneCards |
| ARHGEF25 | 0.402495056 | GeneCards |
| BTN2A2 | 0.402495056 | GeneCards |
| CDC42EP4 | 0.402495056 | GeneCards |
| ZNF184 | 0.402495056 | GeneCards |
| ZNF34 | 0.402495056 | GeneCards |
| TCFL5 | 0.402495056 | GeneCards |
| TULP2 | 0.402495056 | GeneCards |
| ZNF45 | 0.402495056 | GeneCards |
| MND1 | 0.402495056 | GeneCards |
| ORC5 | 0.402495056 | GeneCards |
| STAC3 | 0.402495056 | GeneCards |
| TENM3 | 0.402495056 | GeneCards |
| MMEL1 | 0.402495056 | GeneCards |
| RNF126 | 0.402495056 | GeneCards |
| ZNF7 | 0.402495056 | GeneCards |
| USE1 | 0.402495056 | GeneCards |
| WDR72 | 0.402495056 | GeneCards |
| TRAPPC2L | 0.402495056 | GeneCards |
| TUBG2 | 0.402495056 | GeneCards |
| ZGPAT | 0.402495056 | GeneCards |
| ZIC4 | 0.402495056 | GeneCards |
| SYT9 | 0.402495056 | GeneCards |
| TIMMDC1 | 0.402495056 | GeneCards |
| TFCP2L1 | 0.402495056 | GeneCards |
| TMEM9 | 0.402495056 | GeneCards |
| TMEM38A | 0.402495056 | GeneCards |
| SOX1 | 0.402495056 | GeneCards |
| SRGAP2 | 0.402495056 | GeneCards |
| STMN3 | 0.402495056 | GeneCards |
| PLA2G15 | 0.402495056 | GeneCards |
| PRSS16 | 0.402495056 | GeneCards |
| REEP3 | 0.402495056 | GeneCards |
| SLC2A4RG | 0.402495056 | GeneCards |
| SARNP | 0.402495056 | GeneCards |
| SENP7 | 0.402495056 | GeneCards |
| SLC37A1 | 0.402495056 | GeneCards |
| PLEKHF2 | 0.402495056 | GeneCards |
| PGLYRP3 | 0.402495056 | GeneCards |
| LYRM7 | 0.402495056 | GeneCards |
| PAQR8 | 0.402495056 | GeneCards |
| MKX | 0.402495056 | GeneCards |
| OR11A1 | 0.402495056 | GeneCards |
| RPRD2 | 0.402495056 | GeneCards |
| RANBP10 | 0.402495056 | GeneCards |
| NECTIN2 | 0.402495056 | GeneCards |
| NDUFA4L2 | 0.402495056 | GeneCards |
| NEK10 | 0.402495056 | GeneCards |
| NEU4 | 0.402495056 | GeneCards |
| NEURL1 | 0.402495056 | GeneCards |
| MRPS21 | 0.402495056 | GeneCards |
| MPPED2 | 0.402495056 | GeneCards |
| MRPL10 | 0.402495056 | GeneCards |
| NOL4 | 0.402495056 | GeneCards |
| RASIP1 | 0.402495056 | GeneCards |
| RBMS2 | 0.402495056 | GeneCards |
| PTOV1 | 0.402495056 | GeneCards |
| SAMD12 | 0.402495056 | GeneCards |
| SCHIP1 | 0.402495056 | GeneCards |
| TNFSF9 | 0.402495056 | GeneCards |
| YPEL5 | 0.402495056 | GeneCards |
| ZFAT | 0.402495056 | GeneCards |
| APOBEC3H | 0.402495056 | GeneCards |
| ADSS2 | 0.402495056 | GeneCards |
| AFAP1L1 | 0.402495056 | GeneCards |
| CORT | 0.402495056 | GeneCards |
| CHERP | 0.402495056 | GeneCards |
| BLOC1S3 | 0.402495056 | GeneCards |
| C8orf37 | 0.402495056 | GeneCards |
| KLHL11 | 0.402495056 | GeneCards |
| KRT80 | 0.402495056 | GeneCards |
| MEX3D | 0.402495056 | GeneCards |
| MIIP | 0.402495056 | GeneCards |
| HELZ | 0.402495056 | GeneCards |
| HSPA12B | 0.402495056 | GeneCards |
| GSTCD | 0.402495056 | GeneCards |
| HOXC11 | 0.402495056 | GeneCards |
| MACROD2 | 0.402495056 | GeneCards |
| MED26 | 0.402495056 | GeneCards |
| GBA3 | 0.402495056 | GeneCards |
| ENGASE | 0.402495056 | GeneCards |
| FBXL17 | 0.402495056 | GeneCards |
| GPATCH8 | 0.402495056 | GeneCards |
| DCAF7 | 0.402495056 | GeneCards |
| DDX55 | 0.402495056 | GeneCards |
| EFHD1 | 0.402495056 | GeneCards |
| BTN3A2 | 0.402495056 | GeneCards |
| CECR2 | 0.402495056 | GeneCards |
| CLUAP1 | 0.402495056 | GeneCards |
| ALPK2 | 0.402495056 | GeneCards |
| ADGRL4 | 0.402495056 | GeneCards |
| ADCK5 | 0.402495056 | GeneCards |
| ALDH16A1 | 0.402495056 | GeneCards |
| ARMC2 | 0.402495056 | GeneCards |
| ARHGAP39 | 0.402495056 | GeneCards |
| CCDC33 | 0.402495056 | GeneCards |
| CCDC103 | 0.402495056 | GeneCards |
| CDIP1 | 0.402495056 | GeneCards |
| CEP83 | 0.402495056 | GeneCards |
| DTX3 | 0.402495056 | GeneCards |
| CSMD3 | 0.402495056 | GeneCards |
| ZNF165 | 0.402495056 | GeneCards |
| ZNF174 | 0.402495056 | GeneCards |
| TCHH | 0.402495056 | GeneCards |
| UBAP2L | 0.402495056 | GeneCards |
| NREP | 0.402495056 | GeneCards |
| MDFI | 0.402495056 | GeneCards |
| RBMS3 | 0.402495056 | GeneCards |
| ZNF608 | 0.402495056 | GeneCards |
| UNC119B | 0.402495056 | GeneCards |
| WDR18 | 0.402495056 | GeneCards |
| TRAM2 | 0.402495056 | GeneCards |
| UQCC2 | 0.402495056 | GeneCards |
| USP38 | 0.402495056 | GeneCards |
| TRMT10C | 0.402495056 | GeneCards |
| TATDN1 | 0.402495056 | GeneCards |
| SPRR1A | 0.402495056 | GeneCards |
| STK31 | 0.402495056 | GeneCards |
| SP8 | 0.402495056 | GeneCards |
| NCLN | 0.402495056 | GeneCards |
| PARVG | 0.402495056 | GeneCards |
| PARD3B | 0.402495056 | GeneCards |
| MOB3B | 0.402495056 | GeneCards |
| SDK1 | 0.402495056 | GeneCards |
| SH3BGR | 0.402495056 | GeneCards |
| PCP4 | 0.402495056 | GeneCards |
| PDIK1L | 0.402495056 | GeneCards |
| PDZRN4 | 0.402495056 | GeneCards |
| PITPNC1 | 0.402495056 | GeneCards |
| PIH1D1 | 0.402495056 | GeneCards |
| OR2B6 | 0.402495056 | GeneCards |
| OR2H1 | 0.402495056 | GeneCards |
| MLLT6 | 0.402495056 | GeneCards |
| NPAS4 | 0.402495056 | GeneCards |
| PPP1R11 | 0.402495056 | GeneCards |
| SH3BGRL3 | 0.402495056 | GeneCards |
| NDST3 | 0.402495056 | GeneCards |
| NEUROD4 | 0.402495056 | GeneCards |
| MED16 | 0.402495056 | GeneCards |
| NAALADL1 | 0.402495056 | GeneCards |
| KCNG1 | 0.402495056 | GeneCards |
| RASGEF1A | 0.402495056 | GeneCards |
| RBM4B | 0.402495056 | GeneCards |
| TMEM30B | 0.402495056 | GeneCards |
| ZDHHC1 | 0.402495056 | GeneCards |
| IQSEC3 | 0.402495056 | GeneCards |
| LBH | 0.402495056 | GeneCards |
| LHFPL3 | 0.402495056 | GeneCards |
| IGFN1 | 0.402495056 | GeneCards |
| HSH2D | 0.402495056 | GeneCards |
| KIF18B | 0.402495056 | GeneCards |
| MAL2 | 0.402495056 | GeneCards |
| IZUMO1 | 0.402495056 | GeneCards |
| JMJD4 | 0.402495056 | GeneCards |
| FBXL6 | 0.402495056 | GeneCards |
| GOLIM4 | 0.402495056 | GeneCards |
| EVA1A | 0.402495056 | GeneCards |
| CMTR1 | 0.402495056 | GeneCards |
| CTU1 | 0.402495056 | GeneCards |
| ABT1 | 0.402495056 | GeneCards |
| C1QL1 | 0.402495056 | GeneCards |
| ARHGAP42 | 0.402495056 | GeneCards |
| CEP85 | 0.402495056 | GeneCards |
| CEP85L | 0.402495056 | GeneCards |
| CCDC91 | 0.402495056 | GeneCards |
| EFR3A | 0.402495056 | GeneCards |
| ENKUR | 0.402495056 | GeneCards |
| OVCA2 | 0.402495056 | GeneCards |
| NBEAL1 | 0.402495056 | GeneCards |
| SPEF2 | 0.402495056 | GeneCards |
| TMCC3 | 0.402495056 | GeneCards |
| NANOS2 | 0.402495056 | GeneCards |
| ZNF577 | 0.402495056 | GeneCards |
| ZNF593 | 0.402495056 | GeneCards |
| ZNF707 | 0.402495056 | GeneCards |
| ZNF75A | 0.402495056 | GeneCards |
| WDR20 | 0.402495056 | GeneCards |
| VASH2 | 0.402495056 | GeneCards |
| ZBTB46 | 0.402495056 | GeneCards |
| ZBTB11 | 0.402495056 | GeneCards |
| ZKSCAN4 | 0.402495056 | GeneCards |
| SRBD1 | 0.402495056 | GeneCards |
| S100A7A | 0.402495056 | GeneCards |
| NUFIP1 | 0.402495056 | GeneCards |
| OGFOD2 | 0.402495056 | GeneCards |
| SNED1 | 0.402495056 | GeneCards |
| TMUB2 | 0.402495056 | GeneCards |
| ARRDC4 | 0.402495056 | GeneCards |
| COMMD5 | 0.402495056 | GeneCards |
| ATF7IP2 | 0.402495056 | GeneCards |
| ATP5F1D | 0.402495056 | GeneCards |
| C11orf68 | 0.402495056 | GeneCards |
| CCDC85B | 0.402495056 | GeneCards |
| GSX2 | 0.402495056 | GeneCards |
| HAUS8 | 0.402495056 | GeneCards |
| LRRC14 | 0.402495056 | GeneCards |
| KRTCAP2 | 0.402495056 | GeneCards |
| GPR31 | 0.402495056 | GeneCards |
| GALNT18 | 0.402495056 | GeneCards |
| FUT11 | 0.402495056 | GeneCards |
| GPX6 | 0.402495056 | GeneCards |
| HOXC5 | 0.402495056 | GeneCards |
| KRT26 | 0.402495056 | GeneCards |
| LIME1 | 0.402495056 | GeneCards |
| LRRC3B | 0.402495056 | GeneCards |
| GLI4 | 0.402495056 | GeneCards |
| FAM184A | 0.402495056 | GeneCards |
| FAM135B | 0.402495056 | GeneCards |
| FAM170A | 0.402495056 | GeneCards |
| CTU2 | 0.402495056 | GeneCards |
| DENND1C | 0.402495056 | GeneCards |
| ACTBL2 | 0.402495056 | GeneCards |
| AJAP1 | 0.402495056 | GeneCards |
| AMZ1 | 0.402495056 | GeneCards |
| CASP8AP2 | 0.402495056 | GeneCards |
| ARL4C | 0.402495056 | GeneCards |
| ARMC3 | 0.402495056 | GeneCards |
| ASXL3 | 0.402495056 | GeneCards |
| CDC42SE2 | 0.402495056 | GeneCards |
| CARF | 0.402495056 | GeneCards |
| ZNF16 | 0.402495056 | GeneCards |
| ZNF517 | 0.402495056 | GeneCards |
| ZNF227 | 0.402495056 | GeneCards |
| ZNF230 | 0.402495056 | GeneCards |
| ZNF250 | 0.402495056 | GeneCards |
| ZNF251 | 0.402495056 | GeneCards |
| OIP5 | 0.402495056 | GeneCards |
| TATDN3 | 0.402495056 | GeneCards |
| TMEM132B | 0.402495056 | GeneCards |
| MTMR10 | 0.402495056 | GeneCards |
| ZNF600 | 0.402495056 | GeneCards |
| URM1 | 0.402495056 | GeneCards |
| WDR88 | 0.402495056 | GeneCards |
| TSKU | 0.402495056 | GeneCards |
| ZNF112 | 0.402495056 | GeneCards |
| YIF1A | 0.402495056 | GeneCards |
| ZKSCAN3 | 0.402495056 | GeneCards |
| THNSL1 | 0.402495056 | GeneCards |
| SYF2 | 0.402495056 | GeneCards |
| SYT16 | 0.402495056 | GeneCards |
| STARD6 | 0.402495056 | GeneCards |
| SP6 | 0.402495056 | GeneCards |
| SLC35E1 | 0.402495056 | GeneCards |
| SLC9B1 | 0.402495056 | GeneCards |
| SCAF8 | 0.402495056 | GeneCards |
| OR2W1 | 0.402495056 | GeneCards |
| PGBD1 | 0.402495056 | GeneCards |
| OR2J3 | 0.402495056 | GeneCards |
| NOL11 | 0.402495056 | GeneCards |
| NUSAP1 | 0.402495056 | GeneCards |
| OR6C4 | 0.402495056 | GeneCards |
| RFTN2 | 0.402495056 | GeneCards |
| SHROOM1 | 0.402495056 | GeneCards |
| MRPL55 | 0.402495056 | GeneCards |
| PRTFDC1 | 0.402495056 | GeneCards |
| RBM34 | 0.402495056 | GeneCards |
| ARHGEF37 | 0.402495056 | GeneCards |
| ANKRD50 | 0.402495056 | GeneCards |
| FRMD8 | 0.402495056 | GeneCards |
| CHTF8 | 0.402495056 | GeneCards |
| DBF4B | 0.402495056 | GeneCards |
| C19orf44 | 0.402495056 | GeneCards |
| H1-5 | 0.402495056 | GeneCards |
| H3-4 | 0.402495056 | GeneCards |
| LEAP2 | 0.402495056 | GeneCards |
| MIDN | 0.402495056 | GeneCards |
| MIER2 | 0.402495056 | GeneCards |
| GRWD1 | 0.402495056 | GeneCards |
| IGSF5 | 0.402495056 | GeneCards |
| KRT27 | 0.402495056 | GeneCards |
| INTS12 | 0.402495056 | GeneCards |
| KRT84 | 0.402495056 | GeneCards |
| LRRC46 | 0.402495056 | GeneCards |
| LRRN2 | 0.402495056 | GeneCards |
| HMGN4 | 0.402495056 | GeneCards |
| FERD3L | 0.402495056 | GeneCards |
| GJD3 | 0.402495056 | GeneCards |
| GLT1D1 | 0.402495056 | GeneCards |
| FBXO45 | 0.402495056 | GeneCards |
| FIBIN | 0.402495056 | GeneCards |
| FAM171A1 | 0.402495056 | GeneCards |
| FAM13C | 0.402495056 | GeneCards |
| FAM71A | 0.402495056 | GeneCards |
| FAM71B | 0.402495056 | GeneCards |
| ATP5F1C | 0.402495056 | GeneCards |
| CMYA5 | 0.402495056 | GeneCards |
| CYB561A3 | 0.402495056 | GeneCards |
| DMXL1 | 0.402495056 | GeneCards |
| ACOXL | 0.402495056 | GeneCards |
| CAVIN1 | 0.402495056 | GeneCards |
| EIF1AD | 0.402495056 | GeneCards |
| CTIF | 0.402495056 | GeneCards |
| ZNF155 | 0.402495056 | GeneCards |
| ZNF221 | 0.402495056 | GeneCards |
| ZNF225 | 0.402495056 | GeneCards |
| ZNF226 | 0.402495056 | GeneCards |
| ZNF234 | 0.402495056 | GeneCards |
| UBXN7 | 0.402495056 | GeneCards |
| UBN2 | 0.402495056 | GeneCards |
| ZNF468 | 0.402495056 | GeneCards |
| ZSCAN12 | 0.402495056 | GeneCards |
| OTOG | 0.402495056 | GeneCards |
| RNFT2 | 0.402495056 | GeneCards |
| RBM15B | 0.402495056 | GeneCards |
| ZNF561 | 0.402495056 | GeneCards |
| WDR53 | 0.402495056 | GeneCards |
| UNC79 | 0.402495056 | GeneCards |
| TMEM163 | 0.402495056 | GeneCards |
| WBP1L | 0.402495056 | GeneCards |
| ZCCHC14 | 0.402495056 | GeneCards |
| ZFYVE19 | 0.402495056 | GeneCards |
| STN1 | 0.402495056 | GeneCards |
| PLEKHG4B | 0.402495056 | GeneCards |
| PRDM11 | 0.402495056 | GeneCards |
| PRSS35 | 0.402495056 | GeneCards |
| SLC26A10 | 0.402495056 | GeneCards |
| SMCP | 0.402495056 | GeneCards |
| PHF14 | 0.402495056 | GeneCards |
| OR2J2 | 0.402495056 | GeneCards |
| P3H4 | 0.402495056 | GeneCards |
| SEMG2 | 0.402495056 | GeneCards |
| SELENON | 0.402495056 | GeneCards |
| NKAPL | 0.402495056 | GeneCards |
| GPN2 | 0.402495056 | GeneCards |
| ZC3H12D | 0.402495056 | GeneCards |
| COX8C | 0.402495056 | GeneCards |
| GSAP | 0.402495056 | GeneCards |
| GSG1L | 0.402495056 | GeneCards |
| DCAF1 | 0.402495056 | GeneCards |
| DCUN1D5 | 0.402495056 | GeneCards |
| ANKRD16 | 0.402495056 | GeneCards |
| DTWD2 | 0.402495056 | GeneCards |
| ZNF223 | 0.402495056 | GeneCards |
| ZNF296 | 0.402495056 | GeneCards |
| ZNF404 | 0.402495056 | GeneCards |
| TMEM109 | 0.402495056 | GeneCards |
| ZSCAN16 | 0.402495056 | GeneCards |
| ZNF701 | 0.402495056 | GeneCards |
| ZNF696 | 0.402495056 | GeneCards |
| UTP23 | 0.402495056 | GeneCards |
| UTP4 | 0.402495056 | GeneCards |
| ZKSCAN8 | 0.402495056 | GeneCards |
| ZNF83 | 0.402495056 | GeneCards |
| TIGD7 | 0.402495056 | GeneCards |
| THUMPD2 | 0.402495056 | GeneCards |
| SPHKAP | 0.402495056 | GeneCards |
| POLR2M | 0.402495056 | GeneCards |
| SMTNL2 | 0.402495056 | GeneCards |
| SARAF | 0.402495056 | GeneCards |
| PALS1 | 0.402495056 | GeneCards |
| OR2B2 | 0.402495056 | GeneCards |
| OR2B3 | 0.402495056 | GeneCards |
| OR12D3 | 0.402495056 | GeneCards |
| PXYLP1 | 0.402495056 | GeneCards |
| MTX3 | 0.402495056 | GeneCards |
| KIAA1958 | 0.402495056 | GeneCards |
| PRR3 | 0.402495056 | GeneCards |
| SCAF1 | 0.402495056 | GeneCards |
| C1orf35 | 0.402495056 | GeneCards |
| C1orf174 | 0.402495056 | GeneCards |
| C7orf25 | 0.402495056 | GeneCards |
| GALNT17 | 0.402495056 | GeneCards |
| MAP1LC3B2 | 0.402495056 | GeneCards |
| LRRC37A | 0.402495056 | GeneCards |
| LRRIQ3 | 0.402495056 | GeneCards |
| FAM131A | 0.402495056 | GeneCards |
| FAM72A | 0.402495056 | GeneCards |
| BTBD7 | 0.402495056 | GeneCards |
| CEP95 | 0.402495056 | GeneCards |
| DYNLT2B | 0.402495056 | GeneCards |
| ZNF283 | 0.402495056 | GeneCards |
| ZNF222 | 0.402495056 | GeneCards |
| ZNF235 | 0.402495056 | GeneCards |
| ZNF28 | 0.402495056 | GeneCards |
| ZNF766 | 0.402495056 | GeneCards |
| ZSCAN26 | 0.402495056 | GeneCards |
| ZSCAN32 | 0.402495056 | GeneCards |
| ZNF616 | 0.402495056 | GeneCards |
| TMEM259 | 0.402495056 | GeneCards |
| TTC9C | 0.402495056 | GeneCards |
| TXNDC8 | 0.402495056 | GeneCards |
| TRIL | 0.402495056 | GeneCards |
| ZNF80 | 0.402495056 | GeneCards |
| ZNF816 | 0.402495056 | GeneCards |
| ZSCAN31 | 0.402495056 | GeneCards |
| ZSCAN9 | 0.402495056 | GeneCards |
| TLE5 | 0.402495056 | GeneCards |
| SUSD6 | 0.402495056 | GeneCards |
| SPOCD1 | 0.402495056 | GeneCards |
| MTFR1L | 0.402495056 | GeneCards |
| PRIMA1 | 0.402495056 | GeneCards |
| PRM1 | 0.402495056 | GeneCards |
| PRR12 | 0.402495056 | GeneCards |
| NUP210L | 0.402495056 | GeneCards |
| MVB12A | 0.402495056 | GeneCards |
| OR1M1 | 0.402495056 | GeneCards |
| OR12D2 | 0.402495056 | GeneCards |
| OR52N2 | 0.402495056 | GeneCards |
| OR5V1 | 0.402495056 | GeneCards |
| OR8D4 | 0.402495056 | GeneCards |
| RSPH6A | 0.402495056 | GeneCards |
| SFMBT2 | 0.402495056 | GeneCards |
| MPND | 0.402495056 | GeneCards |
| MRO | 0.402495056 | GeneCards |
| MROH7 | 0.402495056 | GeneCards |
| TMEM26 | 0.402495056 | GeneCards |
| ZBED9 | 0.402495056 | GeneCards |
| ZCCHC9 | 0.402495056 | GeneCards |
| ZDHHC12 | 0.402495056 | GeneCards |
| AP5S1 | 0.402495056 | GeneCards |
| C1orf189 | 0.402495056 | GeneCards |
| C1orf68 | 0.402495056 | GeneCards |
| H2AC13 | 0.402495056 | GeneCards |
| KPRP | 0.402495056 | GeneCards |
| ENKD1 | 0.402495056 | GeneCards |
| FAM220A | 0.402495056 | GeneCards |
| FLYWCH2 | 0.402495056 | GeneCards |
| CENPS | 0.402495056 | GeneCards |
| CCDC171 | 0.402495056 | GeneCards |
| ARHGAP45 | 0.402495056 | GeneCards |
| C11orf1 | 0.402495056 | GeneCards |
| ELSPBP1 | 0.402495056 | GeneCards |
| ZNF284 | 0.402495056 | GeneCards |
| ZNF391 | 0.402495056 | GeneCards |
| ZNF836 | 0.402495056 | GeneCards |
| OR10C1 | 0.402495056 | GeneCards |
| RNF187 | 0.402495056 | GeneCards |
| ZNF578 | 0.402495056 | GeneCards |
| ZNF613 | 0.402495056 | GeneCards |
| ZNF611 | 0.402495056 | GeneCards |
| ZNF623 | 0.402495056 | GeneCards |
| TUSC1 | 0.402495056 | GeneCards |
| TIGD5 | 0.402495056 | GeneCards |
| SQOR | 0.402495056 | GeneCards |
| RTP2 | 0.402495056 | GeneCards |
| PCP4L1 | 0.402495056 | GeneCards |
| PFN3 | 0.402495056 | GeneCards |
| OR14J1 | 0.402495056 | GeneCards |
| RIPOR2 | 0.402495056 | GeneCards |
| SAXO1 | 0.402495056 | GeneCards |
| MROH1 | 0.402495056 | GeneCards |
| KLRG2 | 0.402495056 | GeneCards |
| LCE3D | 0.402495056 | GeneCards |
| PTCHD3 | 0.402495056 | GeneCards |
| DAW1 | 0.402495056 | GeneCards |
| C15orf61 | 0.402495056 | GeneCards |
| H2AC15 | 0.402495056 | GeneCards |
| H2AC16 | 0.402495056 | GeneCards |
| H2AC17 | 0.402495056 | GeneCards |
| H2BC5 | 0.402495056 | GeneCards |
| H3C10 | 0.402495056 | GeneCards |
| MICALCL | 0.402495056 | GeneCards |
| MILR1 | 0.402495056 | GeneCards |
| IGHA1 | 0.402495056 | GeneCards |
| H2AW | 0.402495056 | GeneCards |
| H3C12 | 0.402495056 | GeneCards |
| KIAA0408 | 0.402495056 | GeneCards |
| LRRC43 | 0.402495056 | GeneCards |
| HGH1 | 0.402495056 | GeneCards |
| FAM228A | 0.402495056 | GeneCards |
| EQTN | 0.402495056 | GeneCards |
| CCDC167 | 0.402495056 | GeneCards |
| CCDC178 | 0.402495056 | GeneCards |
| CCDC81 | 0.402495056 | GeneCards |
| CAVIN3 | 0.402495056 | GeneCards |
| ARL5C | 0.402495056 | GeneCards |
| EFCAB12 | 0.402495056 | GeneCards |
| ZNRD2 | 0.402495056 | GeneCards |
| MORN3 | 0.402495056 | GeneCards |
| SPOUT1 | 0.402495056 | GeneCards |
| ZNF678 | 0.402495056 | GeneCards |
| TUT7 | 0.402495056 | GeneCards |
| TENT4B | 0.402495056 | GeneCards |
| ZNF808 | 0.402495056 | GeneCards |
| TDRD10 | 0.402495056 | GeneCards |
| SOWAHA | 0.402495056 | GeneCards |
| SPRR2G | 0.402495056 | GeneCards |
| SPRR2D | 0.402495056 | GeneCards |
| PRORP | 0.402495056 | GeneCards |
| NXPE3 | 0.402495056 | GeneCards |
| MTRFR | 0.402495056 | GeneCards |
| OR6M1 | 0.402495056 | GeneCards |
| SAMD13 | 0.402495056 | GeneCards |
| SAMD5 | 0.402495056 | GeneCards |
| ZC2HC1B | 0.402495056 | GeneCards |
| H3C11 | 0.402495056 | GeneCards |
| H1-10 | 0.402495056 | GeneCards |
| H2BC15 | 0.402495056 | GeneCards |
| MARCHF1 | 0.402495056 | GeneCards |
| FDXACB1 | 0.402495056 | GeneCards |
| ABRAXAS2 | 0.402495056 | GeneCards |
| ZNF285 | 0.402495056 | GeneCards |
| ZNF615 | 0.402495056 | GeneCards |
| TASOR | 0.402495056 | GeneCards |
| SWI5 | 0.402495056 | GeneCards |
| SLF1 | 0.402495056 | GeneCards |
| ARL17B | 0.402495056 | GeneCards |
| FNDC9 | 0.402495056 | GeneCards |
| H2BC6 | 0.402495056 | GeneCards |
| MINDY3 | 0.402495056 | GeneCards |
| H2AC14 | 0.402495056 | GeneCards |
| H2BC13 | 0.402495056 | GeneCards |
| H2BC14 | 0.402495056 | GeneCards |
| MAB21L3 | 0.402495056 | GeneCards |
| FBH1 | 0.402495056 | GeneCards |
| FAM110D | 0.402495056 | GeneCards |
| ATP23 | 0.402495056 | GeneCards |
| BORCS7 | 0.402495056 | GeneCards |
| ANKRD30B | 0.402495056 | GeneCards |
| C2orf83 | 0.402495056 | GeneCards |
| TMEM235 | 0.402495056 | GeneCards |
| TMEM225 | 0.402495056 | GeneCards |
| ZSCAN23 | 0.402495056 | GeneCards |
| SPACA7 | 0.402495056 | GeneCards |
| SPRR2E | 0.402495056 | GeneCards |
| PRAG1 | 0.402495056 | GeneCards |
| SMIM7 | 0.402495056 | GeneCards |
| ODAD2 | 0.402495056 | GeneCards |
| PHETA1 | 0.402495056 | GeneCards |
| DMAC1 | 0.402495056 | GeneCards |
| C4orf50 | 0.402495056 | GeneCards |
| LRATD1 | 0.402495056 | GeneCards |
| H2BC17 | 0.402495056 | GeneCards |
| LCE1B | 0.402495056 | GeneCards |
| LCE2B | 0.402495056 | GeneCards |
| LHFPL6 | 0.402495056 | GeneCards |
| MARCHF9 | 0.402495056 | GeneCards |
| HDHD5 | 0.402495056 | GeneCards |
| HMSD | 0.402495056 | GeneCards |
| GRAMD2B | 0.402495056 | GeneCards |
| TCP10L2 | 0.402495056 | GeneCards |
| POM121L2 | 0.402495056 | GeneCards |
| TMEM131L | 0.402495056 | GeneCards |
| TAFA5 | 0.402495056 | GeneCards |
| SPRING1 | 0.402495056 | GeneCards |
| SPRR4 | 0.402495056 | GeneCards |
| RTP5 | 0.402495056 | GeneCards |
| S100A7L2 | 0.402495056 | GeneCards |
| RIIAD1 | 0.402495056 | GeneCards |
| RSRP1 | 0.402495056 | GeneCards |
| HOTAIR | 0.402495056 | GeneCards |
| LCE3A | 0.402495056 | GeneCards |
| PRXL2B | 0.402495056 | GeneCards |
| H2BU1 | 0.402495056 | GeneCards |
| MFSD13A | 0.402495056 | GeneCards |
| LCE1A | 0.402495056 | GeneCards |
| LCE2D | 0.402495056 | GeneCards |
| LCE4A | 0.402495056 | GeneCards |
| HLA-H | 0.402495056 | GeneCards |
| FMC1 | 0.402495056 | GeneCards |
| DNAAF9 | 0.402495056 | GeneCards |
| DIPK1A | 0.402495056 | GeneCards |
| C10orf105 | 0.402495056 | GeneCards |
| NIBAN3 | 0.402495056 | GeneCards |
| NBR2 | 0.402495056 | GeneCards |
| TCL6 | 0.402495056 | GeneCards |
| ZNF880 | 0.402495056 | GeneCards |
| PCNX3 | 0.402495056 | GeneCards |
| LCE3E | 0.402495056 | GeneCards |
| BBLN | 0.402495056 | GeneCards |
| LCE2A | 0.402495056 | GeneCards |
| LCE2C | 0.402495056 | GeneCards |
| LCE6A | 0.402495056 | GeneCards |
| FAM169B | 0.402495056 | GeneCards |
| STUM | 0.402495056 | GeneCards |
| MARCHF11 | 0.402495056 | GeneCards |
| LCE1F | 0.402495056 | GeneCards |
| LCE1E | 0.402495056 | GeneCards |
| CEP295NL | 0.402495056 | GeneCards |
| DNAAF8 | 0.402495056 | GeneCards |
| CRYBG2 | 0.402495056 | GeneCards |
| AHSA2P | 0.402495056 | GeneCards |
| C2CD4D | 0.402495056 | GeneCards |
| CFAP54 | 0.402495056 | GeneCards |
| CSKMT | 0.402495056 | GeneCards |
| SPEM2 | 0.402495056 | GeneCards |
| TMEM270 | 0.402495056 | GeneCards |
| USP17L3 | 0.402495056 | GeneCards |
| SPATA45 | 0.402495056 | GeneCards |
| IQCJ-SCHIP1 | 0.402495056 | GeneCards |
| HYMAI | 0.402495056 | GeneCards |
| ASB16-AS1 | 0.402495056 | GeneCards |
| BTN2A3P | 0.402495056 | GeneCards |
| PRR9 | 0.402495056 | GeneCards |
| ADM5 | 0.402495056 | GeneCards |
| C16orf82 | 0.402495056 | GeneCards |
| WDR97 | 0.402495056 | GeneCards |
| GDNF-AS1 | 0.402495056 | GeneCards |
| GGNBP1 | 0.402495056 | GeneCards |
| CENPS-CORT | 0.402495056 | GeneCards |
| MIR615 | 0.402495056 | GeneCards |
| MTRNR2L4 | 0.402495056 | GeneCards |
| MKRN2OS | 0.402495056 | GeneCards |
| FTCDNL1 | 0.402495056 | GeneCards |
| BTNL10 | 0.402495056 | GeneCards |
| C9orf92 | 0.402495056 | GeneCards |
| MIR181B1 | 0.402495056 | GeneCards |
| LINC02909 | 0.402495056 | GeneCards |
| CYP1B1-AS1 | 0.402495056 | GeneCards |
| B3GALT5-AS1 | 0.402495056 | GeneCards |
| LINC00271 | 0.402495056 | GeneCards |
| HLA-J | 0.402495056 | GeneCards |
| CASC15 | 0.402495056 | GeneCards |
| ZNF137P | 0.402495056 | GeneCards |
| ZNF561-AS1 | 0.402495056 | GeneCards |
| PIK3CD-AS1 | 0.402495056 | GeneCards |
| RTEL1-TNFRSF6B | 0.402495056 | GeneCards |
| MIR30D | 0.402495056 | GeneCards |
| HCG11 | 0.402495056 | GeneCards |
| LINC00528 | 0.402495056 | GeneCards |
| LINC02912 | 0.402495056 | GeneCards |
| LINC00636 | 0.402495056 | GeneCards |
| C4B_2 | 0.402495056 | GeneCards |
| GUSBP1 | 0.402495056 | GeneCards |
| IGH | 0.402495056 | GeneCards |
| HOXC-AS1 | 0.402495056 | GeneCards |
| HSP90AB2P | 0.402495056 | GeneCards |
| LINC00877 | 0.402495056 | GeneCards |
| HLA-F-AS1 | 0.402495056 | GeneCards |
| FMC1-LUC7L2 | 0.402495056 | GeneCards |
| EPB41L4A-AS1 | 0.402495056 | GeneCards |
| CALML3-AS1 | 0.402495056 | GeneCards |
| WDFY3-AS2 | 0.402495056 | GeneCards |
| MIR873 | 0.402495056 | GeneCards |
| MIR802 | 0.402495056 | GeneCards |
| NAMA | 0.402495056 | GeneCards |
| KRT87P | 0.402495056 | GeneCards |
| LINC00243 | 0.402495056 | GeneCards |
| LINC00598 | 0.402495056 | GeneCards |
| LINC01101 | 0.402495056 | GeneCards |
| LINC01556 | 0.402495056 | GeneCards |
| KLF3-AS1 | 0.402495056 | GeneCards |
| SNORA65 | 0.402495056 | GeneCards |
| SNORA66 | 0.402495056 | GeneCards |
| SNORA14B | 0.402495056 | GeneCards |
| LINC01550 | 0.402495056 | GeneCards |
| LINC00160 | 0.402495056 | GeneCards |
| MIR3679 | 0.402495056 | GeneCards |
| MIR1908 | 0.402495056 | GeneCards |
| MIR147A | 0.402495056 | GeneCards |
| HCG4B | 0.402495056 | GeneCards |
| HOXC-AS3 | 0.402495056 | GeneCards |
| LINC00535 | 0.402495056 | GeneCards |
| LINC02210 | 0.402495056 | GeneCards |
| MACC1-AS1 | 0.402495056 | GeneCards |
| CARMN | 0.402495056 | GeneCards |
| NBAT1 | 0.402495056 | GeneCards |
| PPT2-EGFL8 | 0.402495056 | GeneCards |
| SNAP25-AS1 | 0.402495056 | GeneCards |
| RPL32P3 | 0.402495056 | GeneCards |
| MROH7-TTC4 | 0.402495056 | GeneCards |
| LINC00635 | 0.402495056 | GeneCards |
| LINC01122 | 0.402495056 | GeneCards |
| LINC01433 | 0.402495056 | GeneCards |
| LINC00707 | 0.402495056 | GeneCards |
| LINC00885 | 0.402495056 | GeneCards |
| SNORD21 | 0.402495056 | GeneCards |
| SNORD33 | 0.402495056 | GeneCards |
| SNORD35A | 0.402495056 | GeneCards |
| COX10-AS1 | 0.402495056 | GeneCards |
| HCG17 | 0.402495056 | GeneCards |
| MIR181A1HG | 0.402495056 | GeneCards |
| MIR320B1 | 0.402495056 | GeneCards |
| MIR1236 | 0.402495056 | GeneCards |
| MIR4254 | 0.402495056 | GeneCards |
| HOXC-AS2 | 0.402495056 | GeneCards |
| LINC00501 | 0.402495056 | GeneCards |
| LINC00536 | 0.402495056 | GeneCards |
| LINC00308 | 0.402495056 | GeneCards |
| FALEC | 0.402495056 | GeneCards |
| CEBPA-DT | 0.402495056 | GeneCards |
| CADM3-AS1 | 0.402495056 | GeneCards |
| UBAC2-AS1 | 0.402495056 | GeneCards |
| ZNRD1ASP | 0.402495056 | GeneCards |
| TEX48 | 0.402495056 | GeneCards |
| LOC283710 | 0.402495056 | GeneCards |
| STX18-AS1 | 0.402495056 | GeneCards |
| MSC-AS1 | 0.402495056 | GeneCards |
| SLC25A25-AS1 | 0.402495056 | GeneCards |
| LINC00592 | 0.402495056 | GeneCards |
| SNORD35B | 0.402495056 | GeneCards |
| SNORD66 | 0.402495056 | GeneCards |
| ADAM1A | 0.402495056 | GeneCards |
| ADAMTS9-AS1 | 0.402495056 | GeneCards |
| APOBEC3B-AS1 | 0.402495056 | GeneCards |
| CHCHD2P9 | 0.402495056 | GeneCards |
| C8orf37-AS1 | 0.402495056 | GeneCards |
| HCG14 | 0.402495056 | GeneCards |
| LINC01387 | 0.402495056 | GeneCards |
| LINC01426 | 0.402495056 | GeneCards |
| MIR3142HG | 0.402495056 | GeneCards |
| LPP-AS2 | 0.402495056 | GeneCards |
| MIR1204 | 0.402495056 | GeneCards |
| MIR3936 | 0.402495056 | GeneCards |
| MIR4772 | 0.402495056 | GeneCards |
| MIR1228 | 0.402495056 | GeneCards |
| MIR4492 | 0.402495056 | GeneCards |
| IGFBP7-AS1 | 0.402495056 | GeneCards |
| IBA57-DT | 0.402495056 | GeneCards |
| IDH1-AS1 | 0.402495056 | GeneCards |
| LINC00578 | 0.402495056 | GeneCards |
| LINC00702 | 0.402495056 | GeneCards |
| LINC01091 | 0.402495056 | GeneCards |
| LRRC37A4P | 0.402495056 | GeneCards |
| KANSL1-AS1 | 0.402495056 | GeneCards |
| HLA-L | 0.402495056 | GeneCards |
| FLVCR1-DT | 0.402495056 | GeneCards |
| CYP17A1-AS1 | 0.402495056 | GeneCards |
| CCNT2-AS1 | 0.402495056 | GeneCards |
| ZNF213-AS1 | 0.402495056 | GeneCards |
| ZNF204P | 0.402495056 | GeneCards |
| LOC100131635 | 0.402495056 | GeneCards |
| LOC100287015 | 0.402495056 | GeneCards |
| ZSCAN16-AS1 | 0.402495056 | GeneCards |
| VCAN-AS1 | 0.402495056 | GeneCards |
| UGDH-AS1 | 0.402495056 | GeneCards |
| TPRG1-AS2 | 0.402495056 | GeneCards |
| ZIM2-AS1 | 0.402495056 | GeneCards |
| FLJ12825 | 0.402495056 | GeneCards |
| TOB2P1 | 0.402495056 | GeneCards |
| LOC284241 | 0.402495056 | GeneCards |
| PRICKLE2-AS2 | 0.402495056 | GeneCards |
| PRICKLE2-AS3 | 0.402495056 | GeneCards |
| MIR647 | 0.402495056 | GeneCards |
| RORA-AS1 | 0.402495056 | GeneCards |
| SDHAP2 | 0.402495056 | GeneCards |
| KLKP1 | 0.402495056 | GeneCards |
| LINC00317 | 0.402495056 | GeneCards |
| LINC00330 | 0.402495056 | GeneCards |
| LINC00332 | 0.402495056 | GeneCards |
| LINC01277 | 0.402495056 | GeneCards |
| SNORD93 | 0.402495056 | GeneCards |
| LINC01750 | 0.402495056 | GeneCards |
| MIR4632 | 0.402495056 | GeneCards |
| MIR3155A | 0.402495056 | GeneCards |
| MINCR | 0.402495056 | GeneCards |
| LINC00393 | 0.402495056 | GeneCards |
| LINC00395 | 0.402495056 | GeneCards |
| LINC00449 | 0.402495056 | GeneCards |
| LINC00999 | 0.402495056 | GeneCards |
| LINC01063 | 0.402495056 | GeneCards |
| LINC02085 | 0.402495056 | GeneCards |
| HLA-K | 0.402495056 | GeneCards |
| FAM172BP | 0.402495056 | GeneCards |
| FAM205C | 0.402495056 | GeneCards |
| CKMT2-AS1 | 0.402495056 | GeneCards |
| ACOXL-AS1 | 0.402495056 | GeneCards |
| ARMC2-AS1 | 0.402495056 | GeneCards |
| MELTF-AS1 | 0.402495056 | GeneCards |
| MCPH1-AS1 | 0.402495056 | GeneCards |
| RNF5P1 | 0.402495056 | GeneCards |
| PTOV1-AS2 | 0.402495056 | GeneCards |
| TPRG1-AS1 | 0.402495056 | GeneCards |
| LOC100288123 | 0.402495056 | GeneCards |
| VN1R10P | 0.402495056 | GeneCards |
| ZKSCAN8P1 | 0.402495056 | GeneCards |
| LOC100506098 | 0.402495056 | GeneCards |
| STARD4-AS1 | 0.402495056 | GeneCards |
| LOC157273 | 0.402495056 | GeneCards |
| LOC401040 | 0.402495056 | GeneCards |
| TSG1 | 0.402495056 | GeneCards |
| STRIT1 | 0.402495056 | GeneCards |
| PLEKHM1P1 | 0.402495056 | GeneCards |
| NUCB1-AS1 | 0.402495056 | GeneCards |
| OR12D1 | 0.402495056 | GeneCards |
| RNU6-8 | 0.402495056 | GeneCards |
| NEURL1-AS1 | 0.402495056 | GeneCards |
| MDFIC2 | 0.402495056 | GeneCards |
| NRAD1 | 0.402495056 | GeneCards |
| LINC00548 | 0.402495056 | GeneCards |
| LINC00626 | 0.402495056 | GeneCards |
| LINC01623 | 0.402495056 | GeneCards |
| LINC00708 | 0.402495056 | GeneCards |
| LINC01168 | 0.402495056 | GeneCards |
| LINC01169 | 0.402495056 | GeneCards |
| LINC01170 | 0.402495056 | GeneCards |
| PSMB8-AS1 | 0.402495056 | GeneCards |
| P4HA2-AS1 | 0.402495056 | GeneCards |
| PTOV1-AS1 | 0.402495056 | GeneCards |
| B3GALT9 | 0.402495056 | GeneCards |
| GRASLND | 0.402495056 | GeneCards |
| LINC01425 | 0.402495056 | GeneCards |
| MIR5047 | 0.402495056 | GeneCards |
| LNCARSR | 0.402495056 | GeneCards |
| MIR4665 | 0.402495056 | GeneCards |
| MIR4728 | 0.402495056 | GeneCards |
| MIR4474 | 0.402495056 | GeneCards |
| MIR4489 | 0.402495056 | GeneCards |
| KRT8P11 | 0.402495056 | GeneCards |
| LINC02268 | 0.402495056 | GeneCards |
| LINC02240 | 0.402495056 | GeneCards |
| LNC-LBCS | 0.402495056 | GeneCards |
| FMNL1-DT | 0.402495056 | GeneCards |
| DHFRP2 | 0.402495056 | GeneCards |
| DARS1-AS1 | 0.402495056 | GeneCards |
| C2CD4D-AS1 | 0.402495056 | GeneCards |
| ELK2AP | 0.402495056 | GeneCards |
| UBXN7-AS1 | 0.402495056 | GeneCards |
| UBE2Q1-AS1 | 0.402495056 | GeneCards |
| LOC101928596 | 0.402495056 | GeneCards |
| LOC101928728 | 0.402495056 | GeneCards |
| LOC101929295 | 0.402495056 | GeneCards |
| LOC100506406 | 0.402495056 | GeneCards |
| LOC100506422 | 0.402495056 | GeneCards |
| NBPF18P | 0.402495056 | GeneCards |
| LOC101928386 | 0.402495056 | GeneCards |
| ZSCAN12P1 | 0.402495056 | GeneCards |
| LOC100240735 | 0.402495056 | GeneCards |
| LOC101927513 | 0.402495056 | GeneCards |
| LOC401312 | 0.402495056 | GeneCards |
| LOC283387 | 0.402495056 | GeneCards |
| LOC644135 | 0.402495056 | GeneCards |
| SEC1P | 0.402495056 | GeneCards |
| NCF4-AS1 | 0.402495056 | GeneCards |
| MIR8085 | 0.402495056 | GeneCards |
| MIR548F3 | 0.402495056 | GeneCards |
| RORA-AS2 | 0.402495056 | GeneCards |
| RPL21P28 | 0.402495056 | GeneCards |
| LINC01222 | 0.402495056 | GeneCards |
| LINC01231 | 0.402495056 | GeneCards |
| LINC01232 | 0.402495056 | GeneCards |
| LINC01127 | 0.402495056 | GeneCards |
| LINC01310 | 0.402495056 | GeneCards |
| LINC00706 | 0.402495056 | GeneCards |
| LINC01241 | 0.402495056 | GeneCards |
| LINC01931 | 0.402495056 | GeneCards |
| LINC02054 | 0.402495056 | GeneCards |
| KRT7-AS | 0.402495056 | GeneCards |
| HLA-V | 0.402495056 | GeneCards |
| MIR4527HG | 0.402495056 | GeneCards |
| MIR4753 | 0.402495056 | GeneCards |
| MIR4637 | 0.402495056 | GeneCards |
| MIR4694 | 0.402495056 | GeneCards |
| LINC01209 | 0.402495056 | GeneCards |
| IL6R-AS1 | 0.402495056 | GeneCards |
| LINC02101 | 0.402495056 | GeneCards |
| LINC02544 | 0.402495056 | GeneCards |
| LINC02649 | 0.402495056 | GeneCards |
| LINC02073 | 0.402495056 | GeneCards |
| FAM86B3P | 0.402495056 | GeneCards |
| AQP5-AS1 | 0.402495056 | GeneCards |
| DHDDS-AS1 | 0.402495056 | GeneCards |
| ADAMTSL4-AS2 | 0.402495056 | GeneCards |
| ADGRA1-AS1 | 0.402495056 | GeneCards |
| CES5AP1 | 0.402495056 | GeneCards |
| CEP83-DT | 0.402495056 | GeneCards |
| LOC101927314 | 0.402495056 | GeneCards |
| LOC101927636 | 0.402495056 | GeneCards |
| LOC100505715 | 0.402495056 | GeneCards |
| LOC100506405 | 0.402495056 | GeneCards |
| LOC100130744 | 0.402495056 | GeneCards |
| NAV2-AS3 | 0.402495056 | GeneCards |
| MIR6721 | 0.402495056 | GeneCards |
| MIR6731 | 0.402495056 | GeneCards |
| MUC20-OT1 | 0.402495056 | GeneCards |
| ENSG00000255224 | 0.402495056 | GeneCards |
| LOC100506403 | 0.402495056 | GeneCards |
| CTD-2350J17.1 | 0.402495056 | GeneCards |
| LOC730338 | 0.402495056 | GeneCards |
| LOC220729 | 0.402495056 | GeneCards |
| SNORA10B | 0.402495056 | GeneCards |
| MIR7846 | 0.402495056 | GeneCards |
| LINC01814 | 0.402495056 | GeneCards |
| LINC01840 | 0.402495056 | GeneCards |
| LINC01739 | 0.402495056 | GeneCards |
| RPL17P9 | 0.402495056 | GeneCards |
| LINC01615 | 0.402495056 | GeneCards |
| LINC01543 | 0.402495056 | GeneCards |
| SOCS3-DT | 0.402495056 | GeneCards |
| C2-AS1 | 0.402495056 | GeneCards |
| ITGB8-AS1 | 0.402495056 | GeneCards |
| LINC01242 | 0.402495056 | GeneCards |
| LINC01996 | 0.402495056 | GeneCards |
| MIR3135B | 0.402495056 | GeneCards |
| MIR4471 | 0.402495056 | GeneCards |
| LINC02688 | 0.402495056 | GeneCards |
| MICE | 0.402495056 | GeneCards |
| MIR3155B | 0.402495056 | GeneCards |
| MIR4305 | 0.402495056 | GeneCards |
| MICB-DT | 0.402495056 | GeneCards |
| IFNA22P | 0.402495056 | GeneCards |
| LINC00430 | 0.402495056 | GeneCards |
| LINC02110 | 0.402495056 | GeneCards |
| LINC02352 | 0.402495056 | GeneCards |
| LINC02121 | 0.402495056 | GeneCards |
| LINC02270 | 0.402495056 | GeneCards |
| LINC02341 | 0.402495056 | GeneCards |
| HLA-T | 0.402495056 | GeneCards |
| HMGN1P30 | 0.402495056 | GeneCards |
| EHMT2-AS1 | 0.402495056 | GeneCards |
| LOC105371414 | 0.402495056 | GeneCards |
| TDGF1P2 | 0.402495056 | GeneCards |
| LOC101927040 | 0.402495056 | GeneCards |
| LOC101927358 | 0.402495056 | GeneCards |
| LOC101927769 | 0.402495056 | GeneCards |
| LOC101927964 | 0.402495056 | GeneCards |
| LOC101929551 | 0.402495056 | GeneCards |
| LOC101929470 | 0.402495056 | GeneCards |
| LOC101929577 | 0.402495056 | GeneCards |
| LOC101928009 | 0.402495056 | GeneCards |
| ENSG00000255921 | 0.402495056 | GeneCards |
| ENSG00000262370 | 0.402495056 | GeneCards |
| ENSG00000229618 | 0.402495056 | GeneCards |
| ENSG00000237370 | 0.402495056 | GeneCards |
| ENSG00000225744 | 0.402495056 | GeneCards |
| ENSG00000236924 | 0.402495056 | GeneCards |
| LOC100996583 | 0.402495056 | GeneCards |
| LOC101927845 | 0.402495056 | GeneCards |
| TTC4P1 | 0.402495056 | GeneCards |
| TSKU-AS1 | 0.402495056 | GeneCards |
| ZBTB11-AS1 | 0.402495056 | GeneCards |
| LOC100129603 | 0.402495056 | GeneCards |
| ST13P6 | 0.402495056 | GeneCards |
| ENSG00000240207 | 0.402495056 | GeneCards |
| SLC6A12-AS1 | 0.402495056 | GeneCards |
| SBNO1-AS1 | 0.402495056 | GeneCards |
| LINC01804 | 0.402495056 | GeneCards |
| MIR5706 | 0.402495056 | GeneCards |
| RAB9BP1 | 0.402495056 | GeneCards |
| RPL23AP1 | 0.402495056 | GeneCards |
| RPL23P8 | 0.402495056 | GeneCards |
| RERE-AS1 | 0.402495056 | GeneCards |
| LINC01012 | 0.402495056 | GeneCards |
| LINC01044 | 0.402495056 | GeneCards |
| LINC01303 | 0.402495056 | GeneCards |
| PABPC4-AS1 | 0.402495056 | GeneCards |
| SNORA58B | 0.402495056 | GeneCards |
| ENSG00000243620 | 0.402495056 | GeneCards |
| LINC01898 | 0.402495056 | GeneCards |
| HLA-U | 0.402495056 | GeneCards |
| LINC02698 | 0.402495056 | GeneCards |
| GSTCD-AS1 | 0.402495056 | GeneCards |
| LINC02128 | 0.402495056 | GeneCards |
| LINC02075 | 0.402495056 | GeneCards |
| LINC02573 | 0.402495056 | GeneCards |
| LINC02494 | 0.402495056 | GeneCards |
| MAP2K1P1 | 0.402495056 | GeneCards |
| EDIL3-DT | 0.402495056 | GeneCards |
| LOC100996664 | 0.402495056 | GeneCards |
| LOC101927243 | 0.402495056 | GeneCards |
| LOC101927770 | 0.402495056 | GeneCards |
| LOC101929172 | 0.402495056 | GeneCards |
| LOC101927531 | 0.402495056 | GeneCards |
| LOC105370802 | 0.402495056 | GeneCards |
| MIR6833 | 0.402495056 | GeneCards |
| MIR6884 | 0.402495056 | GeneCards |
| MTND1P8 | 0.402495056 | GeneCards |
| RNA5SP192 | 0.402495056 | GeneCards |
| LOC105377623 | 0.402495056 | GeneCards |
| ENSG00000253519 | 0.402495056 | GeneCards |
| ENSG00000255182 | 0.402495056 | GeneCards |
| ENSG00000255400 | 0.402495056 | GeneCards |
| ENSG00000257449 | 0.402495056 | GeneCards |
| ENSG00000258035 | 0.402495056 | GeneCards |
| ENSG00000258989 | 0.402495056 | GeneCards |
| ENSG00000259508 | 0.402495056 | GeneCards |
| ENSG00000260793 | 0.402495056 | GeneCards |
| ENSG00000271958 | 0.402495056 | GeneCards |
| ENSG00000272462 | 0.402495056 | GeneCards |
| ENSG00000249494 | 0.402495056 | GeneCards |
| ENSG00000254089 | 0.402495056 | GeneCards |
| ENSG00000259097 | 0.402495056 | GeneCards |
| ENSG00000260470 | 0.402495056 | GeneCards |
| ENSG00000261000 | 0.402495056 | GeneCards |
| ENSG00000267505 | 0.402495056 | GeneCards |
| ENSG00000267737 | 0.402495056 | GeneCards |
| ENSG00000268047 | 0.402495056 | GeneCards |
| ENSG00000250740 | 0.402495056 | GeneCards |
| ENSG00000254561 | 0.402495056 | GeneCards |
| ENSG00000256947 | 0.402495056 | GeneCards |
| ENSG00000266903 | 0.402495056 | GeneCards |
| ENSG00000269934 | 0.402495056 | GeneCards |
| ENSG00000248544 | 0.402495056 | GeneCards |
| ENSG00000254028 | 0.402495056 | GeneCards |
| ENSG00000254461 | 0.402495056 | GeneCards |
| ENSG00000255320 | 0.402495056 | GeneCards |
| ENSG00000255946 | 0.402495056 | GeneCards |
| ENSG00000267275 | 0.402495056 | GeneCards |
| ENSG00000267758 | 0.402495056 | GeneCards |
| ENSG00000271989 | 0.402495056 | GeneCards |
| ENSG00000205414 | 0.402495056 | GeneCards |
| ENSG00000223881 | 0.402495056 | GeneCards |
| ENSG00000224374 | 0.402495056 | GeneCards |
| ENSG00000224505 | 0.402495056 | GeneCards |
| ENSG00000224844 | 0.402495056 | GeneCards |
| ENSG00000236352 | 0.402495056 | GeneCards |
| ENSG00000214919 | 0.402495056 | GeneCards |
| ENSG00000229533 | 0.402495056 | GeneCards |
| ENSG00000236194 | 0.402495056 | GeneCards |
| ENSG00000204971 | 0.402495056 | GeneCards |
| ENSG00000224132 | 0.402495056 | GeneCards |
| ENSG00000224269 | 0.402495056 | GeneCards |
| ENSG00000225643 | 0.402495056 | GeneCards |
| ENSG00000233175 | 0.402495056 | GeneCards |
| ENSG00000234084 | 0.402495056 | GeneCards |
| ENSG00000235078 | 0.402495056 | GeneCards |
| ENSG00000214546 | 0.402495056 | GeneCards |
| ENSG00000227192 | 0.402495056 | GeneCards |
| ENSG00000228944 | 0.402495056 | GeneCards |
| ENSG00000229299 | 0.402495056 | GeneCards |
| ENSG00000230773 | 0.402495056 | GeneCards |
| ENSG00000231953 | 0.402495056 | GeneCards |
| ENSG00000232807 | 0.402495056 | GeneCards |
| ENSG00000236471 | 0.402495056 | GeneCards |
| ENSG00000236536 | 0.402495056 | GeneCards |
| LOC101927815 | 0.402495056 | GeneCards |
| ZNF602P | 0.402495056 | GeneCards |
| ZNF603P | 0.402495056 | GeneCards |
| USP38-DT | 0.402495056 | GeneCards |
| TRP-AGG2-1 | 0.402495056 | GeneCards |
| TRP-CGG1-1 | 0.402495056 | GeneCards |
| ZFPM1-AS1 | 0.402495056 | GeneCards |
| FKSG29 | 0.402495056 | GeneCards |
| LOC100129636 | 0.402495056 | GeneCards |
| NCRNA00250 | 0.402495056 | GeneCards |
| TSL | 0.402495056 | GeneCards |
| PFN1P11 | 0.402495056 | GeneCards |
| PAIP1P1 | 0.402495056 | GeneCards |
| MIR6830 | 0.402495056 | GeneCards |
| MIR6866 | 0.402495056 | GeneCards |
| MIR6867 | 0.402495056 | GeneCards |
| MIR8086 | 0.402495056 | GeneCards |
| NPM1P39 | 0.402495056 | GeneCards |
| MIR5700 | 0.402495056 | GeneCards |
| MIR6829 | 0.402495056 | GeneCards |
| MIR7977 | 0.402495056 | GeneCards |
| RANP1 | 0.402495056 | GeneCards |
| RNU7-57P | 0.402495056 | GeneCards |
| RSL1D1-DT | 0.402495056 | GeneCards |
| RPL23AP15 | 0.402495056 | GeneCards |
| RPSAP36 | 0.402495056 | GeneCards |
| RPLP0P3 | 0.402495056 | GeneCards |
| PTGES3P4 | 0.402495056 | GeneCards |
| RNU6-588P | 0.402495056 | GeneCards |
| RNU6-61P | 0.402495056 | GeneCards |
| RNU6-653P | 0.402495056 | GeneCards |
| RPL17P46 | 0.402495056 | GeneCards |
| RPS27P25 | 0.402495056 | GeneCards |
| RPS9P4 | 0.402495056 | GeneCards |
| RNU4-24P | 0.402495056 | GeneCards |
| MRPL35P2 | 0.402495056 | GeneCards |
| KRT18P1 | 0.402495056 | GeneCards |
| PSMA2P2 | 0.402495056 | GeneCards |
| RUNX3-AS1 | 0.402495056 | GeneCards |
| ENSG00000245156 | 0.402495056 | GeneCards |
| LINC01896 | 0.402495056 | GeneCards |
| LINC01997 | 0.402495056 | GeneCards |
| KRT8P34 | 0.402495056 | GeneCards |
| HSD3BP1 | 0.402495056 | GeneCards |
| KRT18P39 | 0.402495056 | GeneCards |
| LINC02168 | 0.402495056 | GeneCards |
| LINC02556 | 0.402495056 | GeneCards |
| LINC02238 | 0.402495056 | GeneCards |
| MAPK8IP1P1 | 0.402495056 | GeneCards |
| MAPK8IP1P2 | 0.402495056 | GeneCards |
| LINC02773 | 0.402495056 | GeneCards |
| FKBP4P1 | 0.402495056 | GeneCards |
| CLIC1P1 | 0.402495056 | GeneCards |
| CCDC195 | 0.402495056 | GeneCards |
| CICP26 | 0.402495056 | GeneCards |
| ZNF45-AS1 | 0.402495056 | GeneCards |
| LOC101929006 | 0.402495056 | GeneCards |
| LOC105369519 | 0.402495056 | GeneCards |
| ENSG00000251867 | 0.402495056 | GeneCards |
| ENSG00000254027 | 0.402495056 | GeneCards |
| ENSG00000255462 | 0.402495056 | GeneCards |
| ENSG00000256364 | 0.402495056 | GeneCards |
| ENSG00000257452 | 0.402495056 | GeneCards |
| ENSG00000260651 | 0.402495056 | GeneCards |
| ENSG00000271955 | 0.402495056 | GeneCards |
| ENSG00000272374 | 0.402495056 | GeneCards |
| ENSG00000285783 | 0.402495056 | GeneCards |
| ENSG00000250602 | 0.402495056 | GeneCards |
| ENSG00000250957 | 0.402495056 | GeneCards |
| ENSG00000251213 | 0.402495056 | GeneCards |
| ENSG00000253824 | 0.402495056 | GeneCards |
| ENSG00000254086 | 0.402495056 | GeneCards |
| ENSG00000254302 | 0.402495056 | GeneCards |
| ENSG00000259877 | 0.402495056 | GeneCards |
| ENSG00000267344 | 0.402495056 | GeneCards |
| ENSG00000267904 | 0.402495056 | GeneCards |
| ENSG00000270165 | 0.402495056 | GeneCards |
| ENSG00000272217 | 0.402495056 | GeneCards |
| ENSG00000249706 | 0.402495056 | GeneCards |
| ENSG00000253980 | 0.402495056 | GeneCards |
| ENSG00000254237 | 0.402495056 | GeneCards |
| ENSG00000254664 | 0.402495056 | GeneCards |
| ENSG00000254991 | 0.402495056 | GeneCards |
| ENSG00000258199 | 0.402495056 | GeneCards |
| ENSG00000258927 | 0.402495056 | GeneCards |
| ENSG00000260233 | 0.402495056 | GeneCards |
| ENSG00000261799 | 0.402495056 | GeneCards |
| ENSG00000265218 | 0.402495056 | GeneCards |
| ENSG00000265912 | 0.402495056 | GeneCards |
| ENSG00000267699 | 0.402495056 | GeneCards |
| ENSG00000267898 | 0.402495056 | GeneCards |
| ENSG00000269825 | 0.402495056 | GeneCards |
| ENSG00000249207 | 0.402495056 | GeneCards |
| ENSG00000262514 | 0.402495056 | GeneCards |
| ENSG00000268087 | 0.402495056 | GeneCards |
| ENSG00000269427 | 0.402495056 | GeneCards |
| ENSG00000269578 | 0.402495056 | GeneCards |
| ENSG00000270179 | 0.402495056 | GeneCards |
| ENSG00000224713 | 0.402495056 | GeneCards |
| ENSG00000228334 | 0.402495056 | GeneCards |
| ENSG00000231760 | 0.402495056 | GeneCards |
| ENSG00000238280 | 0.402495056 | GeneCards |
| ENSG00000223374 | 0.402495056 | GeneCards |
| ENSG00000231756 | 0.402495056 | GeneCards |
| ENSG00000235351 | 0.402495056 | GeneCards |
| ENSG00000200334 | 0.402495056 | GeneCards |
| ENSG00000225330 | 0.402495056 | GeneCards |
| ENSG00000227269 | 0.402495056 | GeneCards |
| ENSG00000227681 | 0.402495056 | GeneCards |
| ENSG00000232949 | 0.402495056 | GeneCards |
| ENSG00000237844 | 0.402495056 | GeneCards |
| ENSG00000215023 | 0.402495056 | GeneCards |
| ENSG00000227252 | 0.402495056 | GeneCards |
| ENSG00000228566 | 0.402495056 | GeneCards |
| ENSG00000229370 | 0.402495056 | GeneCards |
| ENSG00000230732 | 0.402495056 | GeneCards |
| ENSG00000232774 | 0.402495056 | GeneCards |
| YWHAQP6 | 0.402495056 | GeneCards |
| SRMP1 | 0.402495056 | GeneCards |
| SLC16A6P1 | 0.402495056 | GeneCards |
| MIR8062 | 0.402495056 | GeneCards |
| LINC01908 | 0.402495056 | GeneCards |
| MIR6729 | 0.402495056 | GeneCards |
| MIR6783 | 0.402495056 | GeneCards |
| MIR6784 | 0.402495056 | GeneCards |
| RNU6-474P | 0.402495056 | GeneCards |
| RNU6-823P | 0.402495056 | GeneCards |
| RNU7-38P | 0.402495056 | GeneCards |
| RPL4P5 | 0.402495056 | GeneCards |
| RNU6-955P | 0.402495056 | GeneCards |
| RNY3P13 | 0.402495056 | GeneCards |
| RN7SKP57 | 0.402495056 | GeneCards |
| RN7SL363P | 0.402495056 | GeneCards |
| RN7SL689P | 0.402495056 | GeneCards |
| RPL10P11 | 0.402495056 | GeneCards |
| RPL35AP4 | 0.402495056 | GeneCards |
| RNU6-12P | 0.402495056 | GeneCards |
| RNU6-1196P | 0.402495056 | GeneCards |
| RNU6-1208P | 0.402495056 | GeneCards |
| RNU6-1209P | 0.402495056 | GeneCards |
| KRT18P16 | 0.402495056 | GeneCards |
| KRT18P9 | 0.402495056 | GeneCards |
| KRT223P | 0.402495056 | GeneCards |
| LINC01683 | 0.402495056 | GeneCards |
| ARPC3P5 | 0.402495056 | GeneCards |
| ARHGAP27P1-BPTFP1-KPNA2P3 | 0.402495056 | GeneCards |
| COPS5P1 | 0.402495056 | GeneCards |
| CCT6P2 | 0.402495056 | GeneCards |
| LINC02752 | 0.402495056 | GeneCards |
| GAPDHP58 | 0.402495056 | GeneCards |
| GAPDHP68 | 0.402495056 | GeneCards |
| GAPDHP32 | 0.402495056 | GeneCards |
| HCG4P5 | 0.402495056 | GeneCards |
| HSPA9P1 | 0.402495056 | GeneCards |
| LINC02373 | 0.402495056 | GeneCards |
| HNRNPA1P32 | 0.402495056 | GeneCards |
| HMGB3P4 | 0.402495056 | GeneCards |
| NIPAL4-DT | 0.402495056 | GeneCards |
| FGFR3P1 | 0.402495056 | GeneCards |
| FGFR1OP2P1 | 0.402495056 | GeneCards |
| ABCD1P2 | 0.402495056 | GeneCards |
| BDP1P | 0.402495056 | GeneCards |
| CDK7P1 | 0.402495056 | GeneCards |
| EEF1DP7 | 0.402495056 | GeneCards |
| EFCAB15P | 0.402495056 | GeneCards |
| LOC105371253 | 0.402495056 | GeneCards |
| LOC101927745 | 0.402495056 | GeneCards |
| LOC101927825 | 0.402495056 | GeneCards |
| LOC101929188 | 0.402495056 | GeneCards |
| LOC105369302 | 0.402495056 | GeneCards |
| LOC105370969 | 0.402495056 | GeneCards |
| ENSG00000248268 | 0.402495056 | GeneCards |
| ENSG00000253880 | 0.402495056 | GeneCards |
| ENSG00000254566 | 0.402495056 | GeneCards |
| ENSG00000259814 | 0.402495056 | GeneCards |
| ENSG00000260121 | 0.402495056 | GeneCards |
| ENSG00000271709 | 0.402495056 | GeneCards |
| ENSG00000273217 | 0.402495056 | GeneCards |
| ENSG00000274414 | 0.402495056 | GeneCards |
| ENSG00000285713 | 0.402495056 | GeneCards |
| ENSG00000287217 | 0.402495056 | GeneCards |
| ENSG00000287265 | 0.402495056 | GeneCards |
| ENSG00000249388 | 0.402495056 | GeneCards |
| ENSG00000250949 | 0.402495056 | GeneCards |
| ENSG00000251861 | 0.402495056 | GeneCards |
| ENSG00000251922 | 0.402495056 | GeneCards |
| ENSG00000253584 | 0.402495056 | GeneCards |
| ENSG00000253613 | 0.402495056 | GeneCards |
| ENSG00000255384 | 0.402495056 | GeneCards |
| ENSG00000261402 | 0.402495056 | GeneCards |
| ENSG00000261584 | 0.402495056 | GeneCards |
| ENSG00000261644 | 0.402495056 | GeneCards |
| ENSG00000261650 | 0.402495056 | GeneCards |
| ENSG00000263041 | 0.402495056 | GeneCards |
| ENSG00000263756 | 0.402495056 | GeneCards |
| ENSG00000267409 | 0.402495056 | GeneCards |
| ENSG00000267642 | 0.402495056 | GeneCards |
| ENSG00000269903 | 0.402495056 | GeneCards |
| ENSG00000285907 | 0.402495056 | GeneCards |
| ENSG00000286084 | 0.402495056 | GeneCards |
| ENSG00000250258 | 0.402495056 | GeneCards |
| ENSG00000254687 | 0.402495056 | GeneCards |
| ENSG00000254733 | 0.402495056 | GeneCards |
| ENSG00000258554 | 0.402495056 | GeneCards |
| ENSG00000259907 | 0.402495056 | GeneCards |
| ENSG00000260670 | 0.402495056 | GeneCards |
| ENSG00000264151 | 0.402495056 | GeneCards |
| ENSG00000268093 | 0.402495056 | GeneCards |
| ENSG00000268743 | 0.402495056 | GeneCards |
| ENSG00000270039 | 0.402495056 | GeneCards |
| ENSG00000282278 | 0.402495056 | GeneCards |
| ENSG00000285708 | 0.402495056 | GeneCards |
| ENSG00000286863 | 0.402495056 | GeneCards |
| ENSG00000248533 | 0.402495056 | GeneCards |
| ENSG00000249626 | 0.402495056 | GeneCards |
| ENSG00000253100 | 0.402495056 | GeneCards |
| ENSG00000253496 | 0.402495056 | GeneCards |
| ENSG00000253507 | 0.402495056 | GeneCards |
| ENSG00000255605 | 0.402495056 | GeneCards |
| ENSG00000257910 | 0.402495056 | GeneCards |
| ENSG00000258860 | 0.402495056 | GeneCards |
| ENSG00000262267 | 0.402495056 | GeneCards |
| ENSG00000262372 | 0.402495056 | GeneCards |
| ENSG00000267002 | 0.402495056 | GeneCards |
| ENSG00000268970 | 0.402495056 | GeneCards |
| ENSG00000271730 | 0.402495056 | GeneCards |
| ENSG00000272172 | 0.402495056 | GeneCards |
| ENSG00000273107 | 0.402495056 | GeneCards |
| ENSG00000276728 | 0.402495056 | GeneCards |
| ENSG00000199867 | 0.402495056 | GeneCards |
| ENSG00000215692 | 0.402495056 | GeneCards |
| ENSG00000236408 | 0.402495056 | GeneCards |
| ENSG00000223727 | 0.402495056 | GeneCards |
| ENSG00000235862 | 0.402495056 | GeneCards |
| ENSG00000237832 | 0.402495056 | GeneCards |
| ENSG00000222529 | 0.402495056 | GeneCards |
| ENSG00000224272 | 0.402495056 | GeneCards |
| ENSG00000225458 | 0.402495056 | GeneCards |
| ENSG00000226445 | 0.402495056 | GeneCards |
| ENSG00000226455 | 0.402495056 | GeneCards |
| ENSG00000226655 | 0.402495056 | GeneCards |
| ENSG00000227227 | 0.402495056 | GeneCards |
| ENSG00000228560 | 0.402495056 | GeneCards |
| ENSG00000231297 | 0.402495056 | GeneCards |
| ENSG00000231324 | 0.402495056 | GeneCards |
| ENSG00000235620 | 0.402495056 | GeneCards |
| ENSG00000236782 | 0.402495056 | GeneCards |
| ENSG00000093100 | 0.402495056 | GeneCards |
| ENSG00000201786 | 0.402495056 | GeneCards |
| ENSG00000203469 | 0.402495056 | GeneCards |
| ENSG00000227485 | 0.402495056 | GeneCards |
| ENSG00000228033 | 0.402495056 | GeneCards |
| ENSG00000232328 | 0.402495056 | GeneCards |
| ENSG00000236356 | 0.402495056 | GeneCards |
| LOC101927668 | 0.402495056 | GeneCards |
| ZNF70P1 | 0.402495056 | GeneCards |
| TOMM22P3 | 0.402495056 | GeneCards |
| TRW-CCA3-1 | 0.402495056 | GeneCards |
| TRT-AGT1-3 | 0.402495056 | GeneCards |
| TRT-CGT6-1 | 0.402495056 | GeneCards |
| ZDHHC20P2 | 0.402495056 | GeneCards |
| LOC100131289 | 0.402495056 | GeneCards |
| LOC105373085 | 0.402495056 | GeneCards |
| TRA-CGC4-1 | 0.402495056 | GeneCards |
| SPTLC1P4 | 0.402495056 | GeneCards |
| SRIP1 | 0.402495056 | GeneCards |
| ENSG00000239828 | 0.402495056 | GeneCards |
| ENSG00000241490 | 0.402495056 | GeneCards |
| MPP7-DT | 0.402495056 | GeneCards |
| MPPED2-AS1 | 0.402495056 | GeneCards |
| MTCO1P28 | 0.402495056 | GeneCards |
| PRDX3P4 | 0.402495056 | GeneCards |
| PPIAP21 | 0.402495056 | GeneCards |
| RLIMP1 | 0.402495056 | GeneCards |
| OR1M4P | 0.402495056 | GeneCards |
| LINC01779 | 0.402495056 | GeneCards |
| MIR6129 | 0.402495056 | GeneCards |
| RNU7-97P | 0.402495056 | GeneCards |
| RPL32P20 | 0.402495056 | GeneCards |
| RPL21P17 | 0.402495056 | GeneCards |
| RPL21P96 | 0.402495056 | GeneCards |
| RPL23AP28 | 0.402495056 | GeneCards |
| RPL24P7 | 0.402495056 | GeneCards |
| RPL9P29 | 0.402495056 | GeneCards |
| RPSAP2 | 0.402495056 | GeneCards |
| RNU7-130P | 0.402495056 | GeneCards |
| RPL26P13 | 0.402495056 | GeneCards |
| RPL41P2 | 0.402495056 | GeneCards |
| RN7SL120P | 0.402495056 | GeneCards |
| RNU6-603P | 0.402495056 | GeneCards |
| RPL17P33 | 0.402495056 | GeneCards |
| SELENOKP3 | 0.402495056 | GeneCards |
| RNU6-1218P | 0.402495056 | GeneCards |
| RNU6-206P | 0.402495056 | GeneCards |
| RNU6-13P | 0.402495056 | GeneCards |
| MRPS17P5 | 0.402495056 | GeneCards |
| MRPS18BP2 | 0.402495056 | GeneCards |
| NOP56P1 | 0.402495056 | GeneCards |
| PABPC1P10 | 0.402495056 | GeneCards |
| YWHAZP3 | 0.402495056 | GeneCards |
| ENSG00000287937 | 0.402495056 | GeneCards |
| lnc-NR1D1-1 | 0.402495056 | GeneCards |
| DPP3P2 | 0.402495056 | GeneCards |
| HLA-Z | 0.402495056 | GeneCards |
| GAPDHP50 | 0.402495056 | GeneCards |
| GAPDHP57 | 0.402495056 | GeneCards |
| H3C9P | 0.402495056 | GeneCards |
| ICE2P2 | 0.402495056 | GeneCards |
| HNRNPA1P31 | 0.402495056 | GeneCards |
| EEF1DP6 | 0.402495056 | GeneCards |
| BTF3P14 | 0.402495056 | GeneCards |
| CYLD-AS1 | 0.402495056 | GeneCards |
| CSRP2P1 | 0.402495056 | GeneCards |
| DDX6P2 | 0.402495056 | GeneCards |
| BRI3P2 | 0.402495056 | GeneCards |
| LOC105374301 | 0.402495056 | GeneCards |
| UBBP5 | 0.402495056 | GeneCards |
| NDUFA5P6 | 0.402495056 | GeneCards |
| NUP50P3 | 0.402495056 | GeneCards |
| NPM1P31 | 0.402495056 | GeneCards |
| ME2P1 | 0.402495056 | GeneCards |
| MTND5P15 | 0.402495056 | GeneCards |
| RNA5SP461 | 0.402495056 | GeneCards |
| RNU1-21P | 0.402495056 | GeneCards |
| ENSG00000253067 | 0.402495056 | GeneCards |
| ENSG00000258687 | 0.402495056 | GeneCards |
| ENSG00000263635 | 0.402495056 | GeneCards |
| ENSG00000264057 | 0.402495056 | GeneCards |
| ENSG00000264058 | 0.402495056 | GeneCards |
| ENSG00000273797 | 0.402495056 | GeneCards |
| ENSG00000273890 | 0.402495056 | GeneCards |
| ENSG00000282804 | 0.402495056 | GeneCards |
| ENSG00000283064 | 0.402495056 | GeneCards |
| ENSG00000283563 | 0.402495056 | GeneCards |
| ENSG00000285622 | 0.402495056 | GeneCards |
| ENSG00000286530 | 0.402495056 | GeneCards |
| ENSG00000286924 | 0.402495056 | GeneCards |
| ENSG00000287161 | 0.402495056 | GeneCards |
| ENSG00000287197 | 0.402495056 | GeneCards |
| ENSG00000287310 | 0.402495056 | GeneCards |
| ENSG00000248262 | 0.402495056 | GeneCards |
| ENSG00000249359 | 0.402495056 | GeneCards |
| ENSG00000253207 | 0.402495056 | GeneCards |
| ENSG00000261329 | 0.402495056 | GeneCards |
| ENSG00000261349 | 0.402495056 | GeneCards |
| ENSG00000268218 | 0.402495056 | GeneCards |
| ENSG00000272088 | 0.402495056 | GeneCards |
| ENSG00000273391 | 0.402495056 | GeneCards |
| ENSG00000286423 | 0.402495056 | GeneCards |
| ENSG00000287539 | 0.402495056 | GeneCards |
| ENSG00000248794 | 0.402495056 | GeneCards |
| ENSG00000251049 | 0.402495056 | GeneCards |
| ENSG00000253656 | 0.402495056 | GeneCards |
| ENSG00000260261 | 0.402495056 | GeneCards |
| ENSG00000263276 | 0.402495056 | GeneCards |
| ENSG00000265400 | 0.402495056 | GeneCards |
| ENSG00000265982 | 0.402495056 | GeneCards |
| ENSG00000266918 | 0.402495056 | GeneCards |
| ENSG00000268191 | 0.402495056 | GeneCards |
| ENSG00000280878 | 0.402495056 | GeneCards |
| ENSG00000281883 | 0.402495056 | GeneCards |
| ENSG00000285848 | 0.402495056 | GeneCards |
| ENSG00000285859 | 0.402495056 | GeneCards |
| ENSG00000285875 | 0.402495056 | GeneCards |
| ENSG00000286043 | 0.402495056 | GeneCards |
| ENSG00000286889 | 0.402495056 | GeneCards |
| ENSG00000262880 | 0.402495056 | GeneCards |
| ENSG00000267340 | 0.402495056 | GeneCards |
| ENSG00000272650 | 0.402495056 | GeneCards |
| ENSG00000273333 | 0.402495056 | GeneCards |
| ENSG00000276680 | 0.402495056 | GeneCards |
| ENSG00000277825 | 0.402495056 | GeneCards |
| ENSG00000278254 | 0.402495056 | GeneCards |
| ENSG00000278376 | 0.402495056 | GeneCards |
| ENSG00000278829 | 0.402495056 | GeneCards |
| ENSG00000278834 | 0.402495056 | GeneCards |
| ENSG00000204876 | 0.402495056 | GeneCards |
| ENSG00000224605 | 0.402495056 | GeneCards |
| ENSG00000225044 | 0.402495056 | GeneCards |
| ENSG00000225931 | 0.402495056 | GeneCards |
| ENSG00000235726 | 0.402495056 | GeneCards |
| ENSG00000237262 | 0.402495056 | GeneCards |
| ENSG00000235286 | 0.402495056 | GeneCards |
| ENSG00000203496 | 0.402495056 | GeneCards |
| ENSG00000212224 | 0.402495056 | GeneCards |
| ENSG00000212553 | 0.402495056 | GeneCards |
| ENSG00000228037 | 0.402495056 | GeneCards |
| ENSG00000237371 | 0.402495056 | GeneCards |
| TREHP1 | 0.402495056 | GeneCards |
| LOC105369781 | 0.402495056 | GeneCards |
| RPL6P23 | 0.402495056 | GeneCards |
| LOC105379109 | 0.402495056 | GeneCards |
| lnc-CDC42BPA-5 | 0.402495056 | GeneCards |
| lnc-CEP57L1-3 | 0.402495056 | GeneCards |
| lnc-EIF4E3-5 | 0.402495056 | GeneCards |
| lnc-EIF4E3-6 | 0.402495056 | GeneCards |
| lnc-FAM76B-1 | 0.402495056 | GeneCards |
| ENSG00000240980 | 0.402495056 | GeneCards |
| PGAM1P1 | 0.402495056 | GeneCards |
| MTHFD2P5 | 0.402495056 | GeneCards |
| SALL4P5 | 0.402495056 | GeneCards |
| PAICSP4 | 0.402495056 | GeneCards |
| RNU6-329P | 0.402495056 | GeneCards |
| RNU6-976P | 0.402495056 | GeneCards |
| RPL35AP15 | 0.402495056 | GeneCards |
| RPL7L1P4 | 0.402495056 | GeneCards |
| RPS6P13 | 0.402495056 | GeneCards |
| RNU7-15P | 0.402495056 | GeneCards |
| RNU7-173P | 0.402495056 | GeneCards |
| RNU7-34P | 0.402495056 | GeneCards |
| RPS20P10 | 0.402495056 | GeneCards |
| RPS26P45 | 0.402495056 | GeneCards |
| RRN3P4 | 0.402495056 | GeneCards |
| RN7SKP93 | 0.402495056 | GeneCards |
| RN7SL493P | 0.402495056 | GeneCards |
| RN7SL547P | 0.402495056 | GeneCards |
| RNU6-612P | 0.402495056 | GeneCards |
| RPL19P8 | 0.402495056 | GeneCards |
| RNU2-47P | 0.402495056 | GeneCards |
| KPNA2P3 | 0.402495056 | GeneCards |
| KRT88P | 0.402495056 | GeneCards |
| SOCS5P5 | 0.402495056 | GeneCards |
| ENSG00000288542 | 0.402495056 | GeneCards |
| ENSG00000288587 | 0.402495056 | GeneCards |
| ENSG00000244349 | 0.402495056 | GeneCards |
| lnc-BCL3-1 | 0.402495056 | GeneCards |
| ENSG00000288575 | 0.402495056 | GeneCards |
| HSALNG0000777 | 0.402495056 | GeneCards |
| NONHSAG044923.2 | 0.402495056 | GeneCards |
| lnc-FAM84B-7 | 0.402495056 | GeneCards |
| lnc-IL1A-3 | 0.402495056 | GeneCards |
| lnc-MICAL2-5 | 0.402495056 | GeneCards |
| lnc-MPHOSPH9-2 | 0.402495056 | GeneCards |
| lnc-NCOA3-4 | 0.402495056 | GeneCards |
| lnc-PANK2-2 | 0.402495056 | GeneCards |
| lnc-RCSD1-5 | 0.402495056 | GeneCards |
| lnc-STARD4-3 | 0.402495056 | GeneCards |
| lnc-STARD4-4 | 0.402495056 | GeneCards |
| lnc-SULF2-2 | 0.402495056 | GeneCards |
| lnc-WDR53-2 | 0.402495056 | GeneCards |
| lnc-TNFAIP3-1 | 0.402495056 | GeneCards |
| lnc-CEACAM20-2 | 0.402495056 | GeneCards |
| lnc-CEP19-1 | 0.402495056 | GeneCards |
| lnc-HNF4G-2 | 0.402495056 | GeneCards |
| lnc-KCNMB2-7 | 0.402495056 | GeneCards |
| lnc-LMAN2-1 | 0.402495056 | GeneCards |
| lnc-RFX8-3 | 0.402495056 | GeneCards |
| lnc-SMOC2-8 | 0.402495056 | GeneCards |
| lnc-STEAP1B-1 | 0.402495056 | GeneCards |
| lnc-SYF2-6 | 0.402495056 | GeneCards |
| lnc-SYF2-7 | 0.402495056 | GeneCards |
| ADAM20P3 | 0.402495056 | GeneCards |
| HTATSF1P1 | 0.402495056 | GeneCards |
| IFITM3P5 | 0.402495056 | GeneCards |
| GLRX3P1 | 0.402495056 | GeneCards |
| BOLA3P2 | 0.402495056 | GeneCards |
| LOC105371272 | 0.402495056 | GeneCards |
| LOC101929578 | 0.402495056 | GeneCards |
| LOC100129728 | 0.402495056 | GeneCards |
| LOC100132669 | 0.402495056 | GeneCards |
| NDUFA5P5 | 0.402495056 | GeneCards |
| MDH1P2 | 0.402495056 | GeneCards |
| NAPGP2 | 0.402495056 | GeneCards |
| ENSG00000258790 | 0.402495056 | GeneCards |
| ENSG00000272980 | 0.402495056 | GeneCards |
| ENSG00000273550 | 0.402495056 | GeneCards |
| ENSG00000273553 | 0.402495056 | GeneCards |
| ENSG00000285616 | 0.402495056 | GeneCards |
| ENSG00000286188 | 0.402495056 | GeneCards |
| ENSG00000286328 | 0.402495056 | GeneCards |
| ENSG00000286525 | 0.402495056 | GeneCards |
| ENSG00000286641 | 0.402495056 | GeneCards |
| ENSG00000286845 | 0.402495056 | GeneCards |
| ENSG00000287027 | 0.402495056 | GeneCards |
| ENSG00000287280 | 0.402495056 | GeneCards |
| ENSG00000287454 | 0.402495056 | GeneCards |
| ENSG00000253638 | 0.402495056 | GeneCards |
| ENSG00000279199 | 0.402495056 | GeneCards |
| ENSG00000286293 | 0.402495056 | GeneCards |
| ENSG00000286596 | 0.402495056 | GeneCards |
| ENSG00000287531 | 0.402495056 | GeneCards |
| ENSG00000255093 | 0.402495056 | GeneCards |
| ENSG00000265298 | 0.402495056 | GeneCards |
| ENSG00000270679 | 0.402495056 | GeneCards |
| ENSG00000271128 | 0.402495056 | GeneCards |
| ENSG00000280381 | 0.402495056 | GeneCards |
| ENSG00000280434 | 0.402495056 | GeneCards |
| ENSG00000285543 | 0.402495056 | GeneCards |
| ENSG00000285688 | 0.402495056 | GeneCards |
| ENSG00000285842 | 0.402495056 | GeneCards |
| ENSG00000285868 | 0.402495056 | GeneCards |
| ENSG00000287279 | 0.402495056 | GeneCards |
| ENSG00000267681 | 0.402495056 | GeneCards |
| ENSG00000228513 | 0.402495056 | GeneCards |
| ENSG00000237669 | 0.402495056 | GeneCards |
| ENSG00000229853 | 0.402495056 | GeneCards |
| ENSG00000218730 | 0.402495056 | GeneCards |
| ENSG00000237493 | 0.402495056 | GeneCards |
| HSALNG0007914 | 0.402495056 | GeneCards |
| HSALNG0007915 | 0.402495056 | GeneCards |
| HSALNG0012082 | 0.402495056 | GeneCards |
| HSALNG0012084 | 0.402495056 | GeneCards |
| LOC100130044 | 0.402495056 | GeneCards |
| TRF-GAA11-1 | 0.402495056 | GeneCards |
| LOC107986649 | 0.402495056 | GeneCards |
| LOC107986777 | 0.402495056 | GeneCards |
| lnc-CARD11-5 | 0.402495056 | GeneCards |
| lnc-DDX5-1 | 0.402495056 | GeneCards |
| lnc-DYRK1A-6 | 0.402495056 | GeneCards |
| lnc-DYRK1A-7 | 0.402495056 | GeneCards |
| lnc-EPS15L1-2 | 0.402495056 | GeneCards |
| lnc-ERMP1-2 | 0.402495056 | GeneCards |
| lnc-ERMP1-3 | 0.402495056 | GeneCards |
| lnc-ERRFI1-4 | 0.402495056 | GeneCards |
| lnc-FMNL1-3 | 0.402495056 | GeneCards |
| lnc-FMNL1-4 | 0.402495056 | GeneCards |
| lnc-GRB7-2 | 0.402495056 | GeneCards |
| lnc-HIST1H2BI-3 | 0.402495056 | GeneCards |
| lnc-HOMER1-7 | 0.402495056 | GeneCards |
| lnc-MDN1-3 | 0.402495056 | GeneCards |
| lnc-MFSD9-9 | 0.402495056 | GeneCards |
| lnc-MFSD9-8 | 0.402495056 | GeneCards |
| lnc-NEUROD2-4 | 0.402495056 | GeneCards |
| lnc-NFKB1-2 | 0.402495056 | GeneCards |
| lnc-NR1D1-5 | 0.402495056 | GeneCards |
| lnc-OR1D5-2 | 0.402495056 | GeneCards |
| lnc-PLK2-3 | 0.402495056 | GeneCards |
| lnc-POLG2-1 | 0.402495056 | GeneCards |
| lnc-RAPGEF5-3 | 0.402495056 | GeneCards |
| lnc-RCOR1-3 | 0.402495056 | GeneCards |
| lnc-RTN4IP1-2 | 0.402495056 | GeneCards |
| lnc-RUNX3-2 | 0.402495056 | GeneCards |
| lnc-SBNO2-2 | 0.402495056 | GeneCards |
| lnc-STAT5A-2 | 0.402495056 | GeneCards |
| lnc-TNFSF18-1 | 0.402495056 | GeneCards |
| lnc-TNS4-4 | 0.402495056 | GeneCards |
| lnc-TRMT10C-6 | 0.402495056 | GeneCards |
| lnc-UPK2-1 | 0.402495056 | GeneCards |
| lnc-USP25-6 | 0.402495056 | GeneCards |
| lnc-USP36-2 | 0.402495056 | GeneCards |
| HSALNG0017884 | 0.402495056 | GeneCards |
| HSALNG0017872-001 | 0.402495056 | GeneCards |
| HSALNG0043020-002 | 0.402495056 | GeneCards |
| HSALNG0043563 | 0.402495056 | GeneCards |
| HSALNG0044142 | 0.402495056 | GeneCards |
| HSALNG0044854 | 0.402495056 | GeneCards |
| HSALNG0046922 | 0.402495056 | GeneCards |
| HSALNG0048645 | 0.402495056 | GeneCards |
| HSALNG0048736 | 0.402495056 | GeneCards |
| HSALNG0049413 | 0.402495056 | GeneCards |
| RFC3P1 | 0.402495056 | GeneCards |
| PKMP4 | 0.402495056 | GeneCards |
| NLRP9P1 | 0.402495056 | GeneCards |
| RNU6-243P | 0.402495056 | GeneCards |
| RNU6-168P | 0.402495056 | GeneCards |
| SOCS5P1 | 0.402495056 | GeneCards |
| ENSG00000287640 | 0.402495056 | GeneCards |
| ENSG00000287775 | 0.402495056 | GeneCards |
| ENSG00000287993 | 0.402495056 | GeneCards |
| ENSG00000287996 | 0.402495056 | GeneCards |
| ENSG00000287999 | 0.402495056 | GeneCards |
| ENSG00000288042 | 0.402495056 | GeneCards |
| ENSG00000288520 | 0.402495056 | GeneCards |
| ENSG00000243995 | 0.402495056 | GeneCards |
| lnc-ZNF296-6 | 0.402495056 | GeneCards |
| lnc-ZPBP2-6 | 0.402495056 | GeneCards |
| M31519 | 0.402495056 | GeneCards |
| MF281500 | 0.402495056 | GeneCards |
| MF281462 | 0.402495056 | GeneCards |
| lnc-ZNF80-1 | 0.402495056 | GeneCards |
| lnc-BCL3-5 | 0.402495056 | GeneCards |
| lnc-BRAP-1 | 0.402495056 | GeneCards |
| lnc-BRD2-2 | 0.402495056 | GeneCards |
| lnc-BRD2-3 | 0.402495056 | GeneCards |
| piR-54460 | 0.402495056 | GeneCards |
| lnc-BTN3A2-3 | 0.402495056 | GeneCards |
| HSALNG0001756-002 | 0.402495056 | GeneCards |
| HSALNG0001872 | 0.402495056 | GeneCards |
| HSALNG0001874 | 0.402495056 | GeneCards |
| HSALNG0013963 | 0.402495056 | GeneCards |
| HSALNG0000636-001 | 0.402495056 | GeneCards |
| HSALNG0012592 | 0.402495056 | GeneCards |
| HSALNG0012596 | 0.402495056 | GeneCards |
| HSALNG0012736 | 0.402495056 | GeneCards |
| FJ601684-149 | 0.402495056 | GeneCards |
| HSALNG0001756-001 | 0.402495056 | GeneCards |
| HSALNG0011252 | 0.402495056 | GeneCards |
| HSALNG0021688 | 0.402495056 | GeneCards |
| HSALNG0022773 | 0.402495056 | GeneCards |
| HSALNG0038623-002 | 0.402495056 | GeneCards |
| HSALNG0041149 | 0.402495056 | GeneCards |
| HSALNG0054253 | 0.402495056 | GeneCards |
| HSALNG0056695 | 0.402495056 | GeneCards |
| HSALNG0059112 | 0.402495056 | GeneCards |
| HSALNG0030166-001 | 0.402495056 | GeneCards |
| HSALNG0042939-001 | 0.402495056 | GeneCards |
| HSALNG0042939-002 | 0.402495056 | GeneCards |
| HSALNG0043020-001 | 0.402495056 | GeneCards |
| HSALNG0046106 | 0.402495056 | GeneCards |
| HSALNG0017887 | 0.402495056 | GeneCards |
| HSALNG0026812 | 0.402495056 | GeneCards |
| HSALNG0030872 | 0.402495056 | GeneCards |
| HSALNG0049404 | 0.402495056 | GeneCards |
| HSALNG0049408 | 0.402495056 | GeneCards |
| HSALNG0049409 | 0.402495056 | GeneCards |
| HSALNG0049557 | 0.402495056 | GeneCards |
| HSALNG0051895 | 0.402495056 | GeneCards |
| HSALNG0057167 | 0.402495056 | GeneCards |
| HSALNG0110443 | 0.402495056 | GeneCards |
| HSALNG0116322 | 0.402495056 | GeneCards |
| HSALNG0116360 | 0.402495056 | GeneCards |
| HSALNG0116820 | 0.402495056 | GeneCards |
| HSALNG0116841 | 0.402495056 | GeneCards |
| HSALNG0118289-002 | 0.402495056 | GeneCards |
| HSALNG0118594 | 0.402495056 | GeneCards |
| HSALNG0124433 | 0.402495056 | GeneCards |
| HSALNG0126489 | 0.402495056 | GeneCards |
| HSALNG0126491 | 0.402495056 | GeneCards |
| HSALNG0126935 | 0.402495056 | GeneCards |
| HSALNG0126931 | 0.402495056 | GeneCards |
| HSALNG0126934 | 0.402495056 | GeneCards |
| HSALNG0130838 | 0.402495056 | GeneCards |
| HSALNG0130833-002 | 0.402495056 | GeneCards |
| HSALNG0130934 | 0.402495056 | GeneCards |
| KX215110 | 0.402495056 | GeneCards |
| lnc-BATF3-4 | 0.402495056 | GeneCards |
| MK280144-094 | 0.402495056 | GeneCards |
| MN298678-016 | 0.402495056 | GeneCards |
| NONHSAG004089.2 | 0.402495056 | GeneCards |
| NONHSAG009596.2 | 0.402495056 | GeneCards |
| NONHSAG011344.2 | 0.402495056 | GeneCards |
| NONHSAG024039.2 | 0.402495056 | GeneCards |
| NONHSAG025068.2 | 0.402495056 | GeneCards |
| piR-38051-158 | 0.402495056 | GeneCards |
| HSALNG0074148-001 | 0.402495056 | GeneCards |
| HSALNG0080317 | 0.402495056 | GeneCards |
| HSALNG0084963 | 0.402495056 | GeneCards |
| HSALNG0085865 | 0.402495056 | GeneCards |
| HSALNG0087585 | 0.402495056 | GeneCards |
| HSALNG0091480 | 0.402495056 | GeneCards |
| HSALNG0091593 | 0.402495056 | GeneCards |
| HSALNG0111362 | 0.402495056 | GeneCards |
| HSALNG0118293 | 0.402495056 | GeneCards |
| HSALNG0121462 | 0.402495056 | GeneCards |
| HSALNG0121463-001 | 0.402495056 | GeneCards |
| HSALNG0124432 | 0.402495056 | GeneCards |
| HSALNG0128303 | 0.402495056 | GeneCards |
| lnc-ADAMTSL4-1 | 0.402495056 | GeneCards |
| lnc-ADGRD2-2 | 0.402495056 | GeneCards |
| lnc-APBB1-2-001 | 0.402495056 | GeneCards |
| lnc-ASF1A-5 | 0.402495056 | GeneCards |
| MK280144-566 | 0.402495056 | GeneCards |
| MN298114-213 | 0.402495056 | GeneCards |
| MN298114-217 | 0.402495056 | GeneCards |
| NONHSAG026205.2 | 0.402495056 | GeneCards |
| NONHSAG026207.2 | 0.402495056 | GeneCards |
| NONHSAG033213.2 | 0.402495056 | GeneCards |
| piR-32940 | 0.402495056 | GeneCards |
| piR-35448 | 0.402495056 | GeneCards |
| piR-38351-410 | 0.402495056 | GeneCards |
| lnc-C5-1 | 0.402495056 | GeneCards |
| lnc-C6orf229-3 | 0.402495056 | GeneCards |
| lnc-FEN1-6 | 0.402495056 | GeneCards |
| lnc-HLA-DQB1-1 | 0.402495056 | GeneCards |
| lnc-HLA-DRB1-4 | 0.402495056 | GeneCards |
| lnc-HLA-DRB5-1-002 | 0.402495056 | GeneCards |
| lnc-IL2RA-4 | 0.402495056 | GeneCards |
| lnc-JPH1-11 | 0.402495056 | GeneCards |
| lnc-KLF12-3 | 0.402495056 | GeneCards |
| lnc-KLF3-2 | 0.402495056 | GeneCards |
| lnc-MFSD9-20 | 0.402495056 | GeneCards |
| lnc-MMEL1-1 | 0.402495056 | GeneCards |
| lnc-MMEL1-2 | 0.402495056 | GeneCards |
| lnc-MPHOSPH9-1 | 0.402495056 | GeneCards |
| lnc-PPIL4-9 | 0.402495056 | GeneCards |
| lnc-PRL-11 | 0.402495056 | GeneCards |
| lnc-RAD50-4 | 0.402495056 | GeneCards |
| lnc-RERE-3 | 0.402495056 | GeneCards |
| lnc-RNF39-9 | 0.402495056 | GeneCards |
| lnc-SMARCE1-3 | 0.402495056 | GeneCards |
| lnc-SPRY4-1 | 0.402495056 | GeneCards |
| lnc-THUMPD2-2 | 0.402495056 | GeneCards |
| lnc-TLR10-1 | 0.402495056 | GeneCards |
| piR-58555-001 | 0.402495056 | GeneCards |
| piR-60051-028 | 0.402495056 | GeneCards |
| RF00005-078 | 0.402495056 | GeneCards |
| RF00017-4306 | 0.402495056 | GeneCards |
| RF00017-4910 | 0.402495056 | GeneCards |
| RF00017-4930 | 0.402495056 | GeneCards |
| RF00017-5131 | 0.402495056 | GeneCards |
| RF00017-7353 | 0.402495056 | GeneCards |
| HSALNG0066268 | 0.402495056 | GeneCards |
| HSALNG0068018 | 0.402495056 | GeneCards |
| HSALNG0084559 | 0.402495056 | GeneCards |
| HSALNG0084562 | 0.402495056 | GeneCards |
| HSALNG0119030 | 0.402495056 | GeneCards |
| HSALNG0119031 | 0.402495056 | GeneCards |
| HSALNG0126932 | 0.402495056 | GeneCards |
| HSALNG0133921 | 0.402495056 | GeneCards |
| HSALNG0135279 | 0.402495056 | GeneCards |
| L13304-030 | 0.402495056 | GeneCards |
| L13708-003 | 0.402495056 | GeneCards |
| lnc-ABCB5-7 | 0.402495056 | GeneCards |
| lnc-CCDC177-6 | 0.402495056 | GeneCards |
| lnc-CSNK1A1-1 | 0.402495056 | GeneCards |
| lnc-DKK3-9 | 0.402495056 | GeneCards |
| lnc-DYM-3 | 0.402495056 | GeneCards |
| lnc-IL5-1-001 | 0.402495056 | GeneCards |
| lnc-IL5-1-002 | 0.402495056 | GeneCards |
| lnc-IRF1-5 | 0.402495056 | GeneCards |
| lnc-IRF1-6 | 0.402495056 | GeneCards |
| lnc-KLF5-15 | 0.402495056 | GeneCards |
| lnc-KLK4-1 | 0.402495056 | GeneCards |
| lnc-MAL2-1 | 0.402495056 | GeneCards |
| lnc-MCL1-8 | 0.402495056 | GeneCards |
| lnc-MED24-6 | 0.402495056 | GeneCards |
| lnc-NAGLU-6 | 0.402495056 | GeneCards |
| lnc-ORMDL3-2 | 0.402495056 | GeneCards |
| lnc-RUNX3-3 | 0.402495056 | GeneCards |
| lnc-SEC24D-2 | 0.402495056 | GeneCards |
| lnc-SLC25A26-9 | 0.402495056 | GeneCards |
| lnc-SLC6A12-1 | 0.402495056 | GeneCards |
| lnc-SUFU-1 | 0.402495056 | GeneCards |
| lnc-SULF2-1 | 0.402495056 | GeneCards |
| lnc-TAX1BP1-4 | 0.402495056 | GeneCards |
| lnc-TEF-1 | 0.402495056 | GeneCards |
| lnc-TNFRSF14-3 | 0.402495056 | GeneCards |
| lnc-YPEL5-5 | 0.402495056 | GeneCards |
| MN297833 | 0.402495056 | GeneCards |
| MN298678-039 | 0.402495056 | GeneCards |
| MN308698 | 0.402495056 | GeneCards |
| MN309202 | 0.402495056 | GeneCards |
| piR-41223 | 0.402495056 | GeneCards |
| piR-47162-095 | 0.402495056 | GeneCards |
| piR-49097-080 | 0.402495056 | GeneCards |
| piR-51033-003 | 0.402495056 | GeneCards |
| piR-57133-490 | 0.402495056 | GeneCards |
| RF00017-4883 | 0.402495056 | GeneCards |
| RF00017-4944 | 0.402495056 | GeneCards |
| RF00017-5575 | 0.402495056 | GeneCards |
| RF00017-6847 | 0.402495056 | GeneCards |
| RF00017-6881 | 0.402495056 | GeneCards |
| RF00017-937 | 0.402495056 | GeneCards |
| RF00066-026 | 0.402495056 | GeneCards |
| HSALNG0074148-002 | 0.402495056 | GeneCards |
| HSALNG0074592 | 0.402495056 | GeneCards |
| HSALNG0081357 | 0.402495056 | GeneCards |
| HSALNG0084964 | 0.402495056 | GeneCards |
| HSALNG0084965 | 0.402495056 | GeneCards |
| HSALNG0085864 | 0.402495056 | GeneCards |
| HSALNG0094708 | 0.402495056 | GeneCards |
| HSALNG0102181 | 0.402495056 | GeneCards |
| HSALNG0104816 | 0.402495056 | GeneCards |
| HSALNG0116262 | 0.402495056 | GeneCards |
| HSALNG0116321 | 0.402495056 | GeneCards |
| HSALNG0116505 | 0.402495056 | GeneCards |
| HSALNG0130835 | 0.402495056 | GeneCards |
| L13304-027 | 0.402495056 | GeneCards |
| lnc-ATP10B-2 | 0.402495056 | GeneCards |
| lnc-C9orf92-2 | 0.402495056 | GeneCards |
| lnc-CLCN2-1 | 0.402495056 | GeneCards |
| lnc-CREB5-4 | 0.402495056 | GeneCards |
| lnc-DTWD2-15 | 0.402495056 | GeneCards |
| lnc-ENO1-4 | 0.402495056 | GeneCards |
| lnc-HIST1H2BN-2 | 0.402495056 | GeneCards |
| lnc-HLA-A-3 | 0.402495056 | GeneCards |
| lnc-HLA-DRA-3 | 0.402495056 | GeneCards |
| lnc-IDE-3 | 0.402495056 | GeneCards |
| lnc-LEPROTL1-9 | 0.402495056 | GeneCards |
| lnc-LRP1-4 | 0.402495056 | GeneCards |
| lnc-MYO1A-2 | 0.402495056 | GeneCards |
| lnc-NOTCH4-1 | 0.402495056 | GeneCards |
| lnc-PLEKHD1-3 | 0.402495056 | GeneCards |
| lnc-TFDP2-13 | 0.402495056 | GeneCards |
| lnc-VSIR-1 | 0.402495056 | GeneCards |
| MK280269-057 | 0.402495056 | GeneCards |
| MK280607-100 | 0.402495056 | GeneCards |
| MK280607-122 | 0.402495056 | GeneCards |
| NONHSAG029119.2 | 0.402495056 | GeneCards |
| NONHSAG043563.2 | 0.402495056 | GeneCards |
| piR-31059 | 0.402495056 | GeneCards |
| piR-31462-028 | 0.402495056 | GeneCards |
| piR-32214-471 | 0.402495056 | GeneCards |
| piR-44538 | 0.402495056 | GeneCards |
| piR-48209-375 | 0.402495056 | GeneCards |
| piR-50129-210 | 0.402495056 | GeneCards |
| piR-56037-112 | 0.402495056 | GeneCards |
| RF00017-1156 | 0.402495056 | GeneCards |
| RF00017-291 | 0.402495056 | GeneCards |
| RF00017-5033 | 0.402495056 | GeneCards |
| RF00017-5595 | 0.402495056 | GeneCards |
| RF00017-7355 | 0.402495056 | GeneCards |
| RF00017-4402 | 0.402495056 | GeneCards |
| RF00017-5312 | 0.402495056 | GeneCards |
| RF00017-5463 | 0.402495056 | GeneCards |
| RF00017-6846 | 0.402495056 | GeneCards |
| MIR12130 | 0.402495056 | GeneCards |
| LOC101929285 | 0.402495056 | GeneCards |
| LOC105369308 | 0.402495056 | GeneCards |
| LOC107984932 | 0.402495056 | GeneCards |
| ENSG00000254247 | 0.402495056 | GeneCards |
| ENSG00000254591 | 0.402495056 | GeneCards |
| ENSG00000284703 | 0.402495056 | GeneCards |
| ENSG00000284844 | 0.402495056 | GeneCards |
| ENSG00000285598 | 0.402495056 | GeneCards |
| ENSG00000285651 | 0.402495056 | GeneCards |
| ENSG00000286170 | 0.402495056 | GeneCards |
| ENSG00000286617 | 0.402495056 | GeneCards |
| ENSG00000286844 | 0.402495056 | GeneCards |
| ENSG00000287138 | 0.402495056 | GeneCards |
| ENSG00000251162 | 0.402495056 | GeneCards |
| ENSG00000285552 | 0.402495056 | GeneCards |
| ENSG00000286003 | 0.402495056 | GeneCards |
| ENSG00000286661 | 0.402495056 | GeneCards |
| ENSG00000286745 | 0.402495056 | GeneCards |
| ENSG00000259301 | 0.402495056 | GeneCards |
| ENSG00000279175 | 0.402495056 | GeneCards |
| ENSG00000280439 | 0.402495056 | GeneCards |
| ENSG00000286246 | 0.402495056 | GeneCards |
| ENSG00000286417 | 0.402495056 | GeneCards |
| ENSG00000287368 | 0.402495056 | GeneCards |
| ENSG00000287410 | 0.402495056 | GeneCards |
| ENSG00000249105 | 0.402495056 | GeneCards |
| ENSG00000257296 | 0.402495056 | GeneCards |
| ENSG00000273177 | 0.402495056 | GeneCards |
| ENSG00000279061 | 0.402495056 | GeneCards |
| ENSG00000227766 | 0.402495056 | GeneCards |
| ENSG00000235797 | 0.402495056 | GeneCards |
| ENSG00000217878 | 0.402495056 | GeneCards |
| HE856181 | 0.402495056 | GeneCards |
| HSALNG0007401 | 0.402495056 | GeneCards |
| HSALNG0012083 | 0.402495056 | GeneCards |
| LOC105369780 | 0.402495056 | GeneCards |
| LOC105374724 | 0.402495056 | GeneCards |
| LOC345571 | 0.402495056 | GeneCards |
| lnc-CD164-3 | 0.402495056 | GeneCards |
| lnc-CSGALNACT2-4 | 0.402495056 | GeneCards |
| lnc-FURIN-2 | 0.402495056 | GeneCards |
| lnc-GSDME-9 | 0.402495056 | GeneCards |
| lnc-MACC1-4 | 0.402495056 | GeneCards |
| lnc-PSMC3IP-4 | 0.402495056 | GeneCards |
| lnc-SLC51A-13 | 0.402495056 | GeneCards |
| lnc-TFEB-2 | 0.402495056 | GeneCards |
| HSALNG0026565 | 0.402495056 | GeneCards |
| HSALNG0026811 | 0.402495056 | GeneCards |
| HSALNG0027604 | 0.402495056 | GeneCards |
| HSALNG0028155 | 0.402495056 | GeneCards |
| HSALNG0028156 | 0.402495056 | GeneCards |
| HSALNG0030560 | 0.402495056 | GeneCards |
| HSALNG0034448 | 0.402495056 | GeneCards |
| HSALNG0044555 | 0.402495056 | GeneCards |
| HSALNG0049135 | 0.402495056 | GeneCards |
| HSALNG0051292-002 | 0.402495056 | GeneCards |
| HSALNG0051898 | 0.402495056 | GeneCards |
| MTCYBP27 | 0.402495056 | GeneCards |
| PPIAP79 | 0.402495056 | GeneCards |
| SELENOTP1 | 0.402495056 | GeneCards |
| NDUFB5P1 | 0.402495056 | GeneCards |
| ENSG00000287728 | 0.402495056 | GeneCards |
| ENSG00000287826 | 0.402495056 | GeneCards |
| AB372643-003 | 0.402495056 | GeneCards |
| lnc-BUB1-7 | 0.402495056 | GeneCards |
| HSALNG0000721 | 0.402495056 | GeneCards |
| HSALNG0012595 | 0.402495056 | GeneCards |
| HSALNG0000283 | 0.402495056 | GeneCards |
| HSALNG0000284 | 0.402495056 | GeneCards |
| HSALNG0022775 | 0.402495056 | GeneCards |
| HSALNG0023323-002 | 0.402495056 | GeneCards |
| HSALNG0023513 | 0.402495056 | GeneCards |
| HSALNG0038628 | 0.402495056 | GeneCards |
| HSALNG0048307 | 0.402495056 | GeneCards |
| HSALNG0053394 | 0.402495056 | GeneCards |
| HSALNG0055329 | 0.402495056 | GeneCards |
| HSALNG0061727 | 0.402495056 | GeneCards |
| HSALNG0017391-001 | 0.402495056 | GeneCards |
| HSALNG0017391-002 | 0.402495056 | GeneCards |
| HSALNG0020090 | 0.402495056 | GeneCards |
| HSALNG0023180 | 0.402495056 | GeneCards |
| HSALNG0030553-001 | 0.402495056 | GeneCards |
| HSALNG0037580 | 0.402495056 | GeneCards |
| HSALNG0044025 | 0.402495056 | GeneCards |
| HSALNG0048288 | 0.402495056 | GeneCards |
| HSALNG0048295 | 0.402495056 | GeneCards |
| HSALNG0048925 | 0.402495056 | GeneCards |
| HSALNG0063252 | 0.402495056 | GeneCards |
| HSALNG0049384-001 | 0.402495056 | GeneCards |
| HSALNG0049384-002 | 0.402495056 | GeneCards |
| HSALNG0049385 | 0.402495056 | GeneCards |
| HSALNG0049386 | 0.402495056 | GeneCards |
| HSALNG0053689-001 | 0.402495056 | GeneCards |
| HSALNG0054050 | 0.402495056 | GeneCards |
| HSALNG0087853 | 0.402495056 | GeneCards |
| HSALNG0087854 | 0.402495056 | GeneCards |
| HSALNG0088091-001 | 0.402495056 | GeneCards |
| HSALNG0088092 | 0.402495056 | GeneCards |
| HSALNG0091166 | 0.402495056 | GeneCards |
| HSALNG0101769 | 0.402495056 | GeneCards |
| HSALNG0112268-002 | 0.402495056 | GeneCards |
| HSALNG0116274 | 0.402495056 | GeneCards |
| HSALNG0116280 | 0.402495056 | GeneCards |
| HSALNG0116821 | 0.402495056 | GeneCards |
| lnc-ATP6V1E2-10 | 0.402495056 | GeneCards |
| MK279940 | 0.402495056 | GeneCards |
| MK280144-001 | 0.402495056 | GeneCards |
| NONHSAG011189.2 | 0.402495056 | GeneCards |
| piR-39099-192 | 0.402495056 | GeneCards |
| piR-39701-021 | 0.402495056 | GeneCards |
| piR-40476-036 | 0.402495056 | GeneCards |
| piR-42324 | 0.402495056 | GeneCards |
| piR-44110-067 | 0.402495056 | GeneCards |
| piR-47350 | 0.402495056 | GeneCards |
| piR-48965-041 | 0.402495056 | GeneCards |
| piR-51137-089 | 0.402495056 | GeneCards |
| piR-53177-024 | 0.402495056 | GeneCards |
| HSALNG0068480 | 0.402495056 | GeneCards |
| HSALNG0091591 | 0.402495056 | GeneCards |
| HSALNG0102085 | 0.402495056 | GeneCards |
| HSALNG0113505 | 0.402495056 | GeneCards |
| HSALNG0113506 | 0.402495056 | GeneCards |
| HSALNG0118099 | 0.402495056 | GeneCards |
| HSALNG0118102 | 0.402495056 | GeneCards |
| HSALNG0121540 | 0.402495056 | GeneCards |
| HSALNG0133311 | 0.402495056 | GeneCards |
| HSALNG0133917 | 0.402495056 | GeneCards |
| HSALNG0135641 | 0.402495056 | GeneCards |
| HSALNG0136146 | 0.402495056 | GeneCards |
| MN297048 | 0.402495056 | GeneCards |
| NONHSAG028751.2 | 0.402495056 | GeneCards |
| piR-31210-010 | 0.402495056 | GeneCards |
| piR-33614-204 | 0.402495056 | GeneCards |
| piR-38129-054 | 0.402495056 | GeneCards |
| piR-39701-047 | 0.402495056 | GeneCards |
| piR-48799-111 | 0.402495056 | GeneCards |
| lnc-GPSM3-1 | 0.402495056 | GeneCards |
| lnc-GTF3C1-5 | 0.402495056 | GeneCards |
| lnc-KLF5-11 | 0.402495056 | GeneCards |
| lnc-PSMB9-14 | 0.402495056 | GeneCards |
| lnc-SLC37A1-2 | 0.402495056 | GeneCards |
| piR-57929 | 0.402495056 | GeneCards |
| piR-59678 | 0.402495056 | GeneCards |
| RF00017-4421 | 0.402495056 | GeneCards |
| RF00017-4566 | 0.402495056 | GeneCards |
| RF00017-781 | 0.402495056 | GeneCards |
| RF00017-783 | 0.402495056 | GeneCards |
| RF02013 | 0.402495056 | GeneCards |
| SNODB1132 | 0.402495056 | GeneCards |
| HSALNG0067078 | 0.402495056 | GeneCards |
| HSALNG0067082 | 0.402495056 | GeneCards |
| HSALNG0083063 | 0.402495056 | GeneCards |
| HSALNG0084557 | 0.402495056 | GeneCards |
| HSALNG0087124 | 0.402495056 | GeneCards |
| HSALNG0098906 | 0.402495056 | GeneCards |
| HSALNG0112268-001 | 0.402495056 | GeneCards |
| HSALNG0114028 | 0.402495056 | GeneCards |
| HSALNG0121541 | 0.402495056 | GeneCards |
| HSALNG0128279 | 0.402495056 | GeneCards |
| hsa-miR-5095-002 | 0.402495056 | GeneCards |
| L13713-172 | 0.402495056 | GeneCards |
| L13713-212 | 0.402495056 | GeneCards |
| lnc-COPS8-18 | 0.402495056 | GeneCards |
| lnc-DPT-2 | 0.402495056 | GeneCards |
| lnc-IL1RL1-3 | 0.402495056 | GeneCards |
| lnc-KLK2-3 | 0.402495056 | GeneCards |
| lnc-LPIN1-4 | 0.402495056 | GeneCards |
| lnc-SHARPIN-4 | 0.402495056 | GeneCards |
| lnc-SMUG1-9 | 0.402495056 | GeneCards |
| lnc-TCP10L2-1 | 0.402495056 | GeneCards |
| lnc-TMEM258-2-002 | 0.402495056 | GeneCards |
| lnc-TNK2-2-001 | 0.402495056 | GeneCards |
| lnc-TNK2-2-002 | 0.402495056 | GeneCards |
| MN309032 | 0.402495056 | GeneCards |
| MN309173 | 0.402495056 | GeneCards |
| piR-32214-580 | 0.402495056 | GeneCards |
| piR-32325-169 | 0.402495056 | GeneCards |
| piR-33614-024 | 0.402495056 | GeneCards |
| piR-33614-237 | 0.402495056 | GeneCards |
| piR-36588-234 | 0.402495056 | GeneCards |
| piR-48553-160 | 0.402495056 | GeneCards |
| RF00017-308 | 0.402495056 | GeneCards |
| RF00017-3940 | 0.402495056 | GeneCards |
| RF00017-4118 | 0.402495056 | GeneCards |
| RF00017-4816 | 0.402495056 | GeneCards |
| RF00951-060 | 0.402495056 | GeneCards |
| HSALNG0068481 | 0.402495056 | GeneCards |
| HSALNG0074593 | 0.402495056 | GeneCards |
| HSALNG0076096-001 | 0.402495056 | GeneCards |
| HSALNG0076096-002 | 0.402495056 | GeneCards |
| HSALNG0093289 | 0.402495056 | GeneCards |
| HSALNG0098908 | 0.402495056 | GeneCards |
| HSALNG0101768 | 0.402495056 | GeneCards |
| HSALNG0103271 | 0.402495056 | GeneCards |
| HSALNG0104813 | 0.402495056 | GeneCards |
| HSALNG0108164-001 | 0.402495056 | GeneCards |
| HSALNG0108166 | 0.402495056 | GeneCards |
| HSALNG0114027 | 0.402495056 | GeneCards |
| HSALNG0132000 | 0.402495056 | GeneCards |
| HSALNG0131998-001 | 0.402495056 | GeneCards |
| HSALNG0133148 | 0.402495056 | GeneCards |
| lnc-AIG1-7 | 0.402495056 | GeneCards |
| lnc-CALHM3-2 | 0.402495056 | GeneCards |
| lnc-CFAP54-3 | 0.402495056 | GeneCards |
| lnc-GLB1-2 | 0.402495056 | GeneCards |
| lnc-HBS1L-2 | 0.402495056 | GeneCards |
| lnc-LHFPL6-7 | 0.402495056 | GeneCards |
| lnc-MYO1A-1 | 0.402495056 | GeneCards |
| lnc-NYAP2-12 | 0.402495056 | GeneCards |
| lnc-OR1M1-1 | 0.402495056 | GeneCards |
| lnc-TFRC-7 | 0.402495056 | GeneCards |
| lnc-TMEM258-2-001 | 0.402495056 | GeneCards |
| lnc-TTC33-6 | 0.402495056 | GeneCards |
| MK280246 | 0.402495056 | GeneCards |
| piR-31951-013 | 0.402495056 | GeneCards |
| piR-32214-183 | 0.402495056 | GeneCards |
| piR-33303-060 | 0.402495056 | GeneCards |
| piR-34303 | 0.402495056 | GeneCards |
| piR-36037-005 | 0.402495056 | GeneCards |
| piR-36358 | 0.402495056 | GeneCards |
| piR-39858-348 | 0.402495056 | GeneCards |
| piR-41829 | 0.402495056 | GeneCards |
| piR-43676-015 | 0.402495056 | GeneCards |
| piR-45349-004 | 0.402495056 | GeneCards |
| piR-49399-010 | 0.402495056 | GeneCards |
| piR-50444-306 | 0.402495056 | GeneCards |
| piR-53177-042 | 0.402495056 | GeneCards |
| piR-60812 | 0.402495056 | GeneCards |
| piR-61945-242 | 0.402495056 | GeneCards |
| RF00017-029 | 0.402495056 | GeneCards |
| RF00017-506 | 0.402495056 | GeneCards |
| RF00017-7356 | 0.402495056 | GeneCards |
| piR-58873 | 0.402495056 | GeneCards |
| piR-59409-061 | 0.402495056 | GeneCards |
| RF00017-4900 | 0.402495056 | GeneCards |
| RF00017-6845 | 0.402495056 | GeneCards |
| RF00017-843 | 0.402495056 | GeneCards |
| RF00994-1045 | 0.402495056 | GeneCards |
| RF00998-023 | 0.402495056 | GeneCards |
| LOC105371498 | 0.402495056 | GeneCards |
| LOC105375023 | 0.402495056 | GeneCards |
| LOC105375130 | 0.402495056 | GeneCards |
| LOC105373976 | 0.402495056 | GeneCards |
| LOC105372636 | 0.402495056 | GeneCards |
| LOC105373117 | 0.402495056 | GeneCards |
| LOC101929770 | 0.402495056 | GeneCards |
| LOC102723444 | 0.402495056 | GeneCards |
| RNU1-152P | 0.402495056 | GeneCards |
| LOC107984890 | 0.402495056 | GeneCards |
| LOC107984558 | 0.402495056 | GeneCards |
| LOC107984576 | 0.402495056 | GeneCards |
| LOC107984238 | 0.402495056 | GeneCards |
| LOC105369875 | 0.402495056 | GeneCards |
| ENSG00000248286 | 0.402495056 | GeneCards |
| ENSG00000257199 | 0.402495056 | GeneCards |
| ENSG00000286168 | 0.402495056 | GeneCards |
| ENSG00000273569 | 0.402495056 | GeneCards |
| ENSG00000279256 | 0.402495056 | GeneCards |
| ENSG00000280362 | 0.402495056 | GeneCards |
| ENSG00000286697 | 0.402495056 | GeneCards |
| ENSG00000286727 | 0.402495056 | GeneCards |
| ENSG00000248791 | 0.402495056 | GeneCards |
| ENSG00000250815 | 0.402495056 | GeneCards |
| ENSG00000265460 | 0.402495056 | GeneCards |
| ENSG00000270248 | 0.402495056 | GeneCards |
| ENSG00000271240 | 0.402495056 | GeneCards |
| ENSG00000277576 | 0.402495056 | GeneCards |
| ENSG00000287010 | 0.402495056 | GeneCards |
| ENSG00000271074 | 0.402495056 | GeneCards |
| ENSG00000274919 | 0.402495056 | GeneCards |
| ENSG00000276521 | 0.402495056 | GeneCards |
| ENSG00000218073 | 0.402495056 | GeneCards |
| ENSG00000234513 | 0.402495056 | GeneCards |
| ENSG00000235400 | 0.402495056 | GeneCards |
| ENSG00000233352 | 0.402495056 | GeneCards |
| ENSG00000223368 | 0.402495056 | GeneCards |
| ENSG00000228289 | 0.402495056 | GeneCards |
| HSALNG0006315 | 0.402495056 | GeneCards |
| HSALNG0011694 | 0.402495056 | GeneCards |
| HSALNG0013061 | 0.402495056 | GeneCards |
| LOC101928368 | 0.402495056 | GeneCards |
| LOC105377890 | 0.402495056 | GeneCards |
| LOC105377462 | 0.402495056 | GeneCards |
| LOC105374970 | 0.402495056 | GeneCards |
| LOC112268244 | 0.402495056 | GeneCards |
| LOC389249 | 0.402495056 | GeneCards |
| LOC391741 | 0.402495056 | GeneCards |
| LOC643172 | 0.402495056 | GeneCards |
| LOC107986431 | 0.402495056 | GeneCards |
| LOC107986455 | 0.402495056 | GeneCards |
| LOC107985371 | 0.402495056 | GeneCards |
| LOC107985499 | 0.402495056 | GeneCards |
| LOC105379354 | 0.402495056 | GeneCards |
| LOC107987046 | 0.402495056 | GeneCards |
| LOC105378924 | 0.402495056 | GeneCards |
| LOC105379185 | 0.402495056 | GeneCards |
| LOC107986115 | 0.402495056 | GeneCards |
| LOC107986884 | 0.402495056 | GeneCards |
| lnc-CDC42BPB-2 | 0.402495056 | GeneCards |
| lnc-CREBBP-3 | 0.402495056 | GeneCards |
| lnc-DDX1-9 | 0.402495056 | GeneCards |
| lnc-EFR3A-8 | 0.402495056 | GeneCards |
| lnc-OR1A2-1 | 0.402495056 | GeneCards |
| lnc-PRR5L-1 | 0.402495056 | GeneCards |
| lnc-RING1-1 | 0.402495056 | GeneCards |
| HSALNG0017416 | 0.402495056 | GeneCards |
| HSALNG0026065 | 0.402495056 | GeneCards |
| HSALNG0028897 | 0.402495056 | GeneCards |
| HSALNG0031239 | 0.402495056 | GeneCards |
| HSALNG0042030 | 0.402495056 | GeneCards |
| HSALNG0042031 | 0.402495056 | GeneCards |
| HSALNG0044557 | 0.402495056 | GeneCards |
| HSALNG0044830 | 0.402495056 | GeneCards |
| HSALNG0045487 | 0.402495056 | GeneCards |
| HSALNG0045772 | 0.402495056 | GeneCards |
| HSALNG0048753 | 0.402495056 | GeneCards |
| HSALNG0049102 | 0.402495056 | GeneCards |
| HSALNG0049272 | 0.402495056 | GeneCards |
| HSALNG0049343 | 0.402495056 | GeneCards |
| HSALNG0056688-001 | 0.402495056 | GeneCards |
| HSALNG0056688-003 | 0.402495056 | GeneCards |
| ENSG00000287723 | 0.402495056 | GeneCards |
| ENSG00000287758 | 0.402495056 | GeneCards |
| HSALNG0015243 | 0.402495056 | GeneCards |
| 5EW4_A-048 | 0.402495056 | GeneCards |
| HE856243 | 0.402495056 | GeneCards |
| HSALNG0008299-004 | 0.402495056 | GeneCards |
| BA000025 | 0.402495056 | GeneCards |
| FJ601684-402 | 0.402495056 | GeneCards |
| HG983975 | 0.402495056 | GeneCards |
| HSALNG0008299-002 | 0.402495056 | GeneCards |
| HSALNG0008485-002 | 0.402495056 | GeneCards |
| HSALNG0010381 | 0.402495056 | GeneCards |
| HSALNG0010382 | 0.402495056 | GeneCards |
| HSALNG0010383 | 0.402495056 | GeneCards |
| HSALNG0013187 | 0.402495056 | GeneCards |
| HSALNG0020420 | 0.402495056 | GeneCards |
| HSALNG0021912 | 0.402495056 | GeneCards |
| HSALNG0023187 | 0.402495056 | GeneCards |
| HSALNG0026338 | 0.402495056 | GeneCards |
| HSALNG0037567 | 0.402495056 | GeneCards |
| HSALNG0038305-002 | 0.402495056 | GeneCards |
| HSALNG0040324 | 0.402495056 | GeneCards |
| HSALNG0041369 | 0.402495056 | GeneCards |
| HSALNG0044831 | 0.402495056 | GeneCards |
| HSALNG0048926 | 0.402495056 | GeneCards |
| HSALNG0053104 | 0.402495056 | GeneCards |
| HSALNG0053673 | 0.402495056 | GeneCards |
| HSALNG0056697 | 0.402495056 | GeneCards |
| HSALNG0059262-001 | 0.402495056 | GeneCards |
| HSALNG0017434 | 0.402495056 | GeneCards |
| HSALNG0019334 | 0.402495056 | GeneCards |
| HSALNG0020089 | 0.402495056 | GeneCards |
| HSALNG0033910 | 0.402495056 | GeneCards |
| HSALNG0034449 | 0.402495056 | GeneCards |
| HSALNG0036208 | 0.402495056 | GeneCards |
| HSALNG0037564 | 0.402495056 | GeneCards |
| HSALNG0037959 | 0.402495056 | GeneCards |
| HSALNG0037960 | 0.402495056 | GeneCards |
| HSALNG0044558 | 0.402495056 | GeneCards |
| HSALNG0046115 | 0.402495056 | GeneCards |
| HSALNG0048296 | 0.402495056 | GeneCards |
| HSALNG0048310 | 0.402495056 | GeneCards |
| HSALNG0062524 | 0.402495056 | GeneCards |
| HSALNG0017867 | 0.402495056 | GeneCards |
| HSALNG0017869 | 0.402495056 | GeneCards |
| HSALNG0027584 | 0.402495056 | GeneCards |
| HSALNG0030011 | 0.402495056 | GeneCards |
| HSALNG0033900 | 0.402495056 | GeneCards |
| HSALNG0033901 | 0.402495056 | GeneCards |
| HSALNG0044653 | 0.402495056 | GeneCards |
| HSALNG0049401 | 0.402495056 | GeneCards |
| HSALNG0049451 | 0.402495056 | GeneCards |
| HSALNG0050314 | 0.402495056 | GeneCards |
| HSALNG0052460 | 0.402495056 | GeneCards |
| HSALNG0054458 | 0.402495056 | GeneCards |
| HSALNG0056499 | 0.402495056 | GeneCards |
| HSALNG0060355 | 0.402495056 | GeneCards |
| HSALNG0073221 | 0.402495056 | GeneCards |
| HSALNG0076087 | 0.402495056 | GeneCards |
| HSALNG0091470 | 0.402495056 | GeneCards |
| HSALNG0092259-002 | 0.402495056 | GeneCards |
| HSALNG0116275 | 0.402495056 | GeneCards |
| HSALNG0116302 | 0.402495056 | GeneCards |
| HSALNG0121975 | 0.402495056 | GeneCards |
| HSALNG0127280 | 0.402495056 | GeneCards |
| HSALNG0131670 | 0.402495056 | GeneCards |
| HSALNG0136145-001 | 0.402495056 | GeneCards |
| HSALNG0136145-002 | 0.402495056 | GeneCards |
| lnc-AIG1-5 | 0.402495056 | GeneCards |
| MN309174-434 | 0.402495056 | GeneCards |
| piR-32214-350 | 0.402495056 | GeneCards |
| piR-33142-027 | 0.402495056 | GeneCards |
| piR-38580-226 | 0.402495056 | GeneCards |
| piR-39488-281 | 0.402495056 | GeneCards |
| piR-39858-549 | 0.402495056 | GeneCards |
| piR-40110-676 | 0.402495056 | GeneCards |
| piR-41195-025 | 0.402495056 | GeneCards |
| piR-41405-084 | 0.402495056 | GeneCards |
| piR-42491-196 | 0.402495056 | GeneCards |
| piR-43105-567 | 0.402495056 | GeneCards |
| piR-44610-011 | 0.402495056 | GeneCards |
| piR-44855 | 0.402495056 | GeneCards |
| piR-46391-002 | 0.402495056 | GeneCards |
| piR-47820-005 | 0.402495056 | GeneCards |
| piR-50437-154 | 0.402495056 | GeneCards |
| piR-50437-332 | 0.402495056 | GeneCards |
| piR-50718-001 | 0.402495056 | GeneCards |
| piR-51137-119 | 0.402495056 | GeneCards |
| piR-51518 | 0.402495056 | GeneCards |
| piR-52079-166 | 0.402495056 | GeneCards |
| piR-52324-061 | 0.402495056 | GeneCards |
| piR-52473 | 0.402495056 | GeneCards |
| piR-53362-001 | 0.402495056 | GeneCards |
| piR-53412 | 0.402495056 | GeneCards |
| HSALNG0067921 | 0.402495056 | GeneCards |
| HSALNG0069669 | 0.402495056 | GeneCards |
| HSALNG0069845 | 0.402495056 | GeneCards |
| HSALNG0070201 | 0.402495056 | GeneCards |
| HSALNG0070307 | 0.402495056 | GeneCards |
| HSALNG0070312 | 0.402495056 | GeneCards |
| HSALNG0070313-001 | 0.402495056 | GeneCards |
| HSALNG0073220-001 | 0.402495056 | GeneCards |
| HSALNG0073920 | 0.402495056 | GeneCards |
| HSALNG0075911-001 | 0.402495056 | GeneCards |
| HSALNG0076042 | 0.402495056 | GeneCards |
| HSALNG0080295 | 0.402495056 | GeneCards |
| HSALNG0091478 | 0.402495056 | GeneCards |
| HSALNG0109572 | 0.402495056 | GeneCards |
| HSALNG0110454 | 0.402495056 | GeneCards |
| HSALNG0128838 | 0.402495056 | GeneCards |
| HSALNG0135465 | 0.402495056 | GeneCards |
| hsa-miR-5095-491 | 0.402495056 | GeneCards |
| hsa-miR-5095-494 | 0.402495056 | GeneCards |
| lnc-ATG4B-1 | 0.402495056 | GeneCards |
| MK280144-070 | 0.402495056 | GeneCards |
| MK280144-392 | 0.402495056 | GeneCards |
| NONHSAG005194.2 | 0.402495056 | GeneCards |
| NONHSAG026393.2 | 0.402495056 | GeneCards |
| NONHSAG045738.2 | 0.402495056 | GeneCards |
| piR-34839 | 0.402495056 | GeneCards |
| piR-36564-019 | 0.402495056 | GeneCards |
| piR-36887-012 | 0.402495056 | GeneCards |
| piR-37586-020 | 0.402495056 | GeneCards |
| piR-42059-001 | 0.402495056 | GeneCards |
| piR-42370-020 | 0.402495056 | GeneCards |
| piR-43107-234 | 0.402495056 | GeneCards |
| piR-50136 | 0.402495056 | GeneCards |
| lnc-CRCT1-1 | 0.402495056 | GeneCards |
| lnc-FAM135B-5 | 0.402495056 | GeneCards |
| lnc-HLA-DMB-2 | 0.402495056 | GeneCards |
| lnc-IL33-3 | 0.402495056 | GeneCards |
| lnc-IPCEF1-2 | 0.402495056 | GeneCards |
| piR-54764-623 | 0.402495056 | GeneCards |
| piR-54987-043 | 0.402495056 | GeneCards |
| piR-56133-037 | 0.402495056 | GeneCards |
| piR-57393-006 | 0.402495056 | GeneCards |
| piR-61514-023 | 0.402495056 | GeneCards |
| piR-61945-355 | 0.402495056 | GeneCards |
| RF00017-1728 | 0.402495056 | GeneCards |
| HSALNG0066501 | 0.402495056 | GeneCards |
| HSALNG0067913-001 | 0.402495056 | GeneCards |
| HSALNG0087119 | 0.402495056 | GeneCards |
| HSALNG0087120 | 0.402495056 | GeneCards |
| HSALNG0096509 | 0.402495056 | GeneCards |
| HSALNG0097921 | 0.402495056 | GeneCards |
| HSALNG0103313 | 0.402495056 | GeneCards |
| HSALNG0112268-003 | 0.402495056 | GeneCards |
| HSALNG0113161 | 0.402495056 | GeneCards |
| HSALNG0114031 | 0.402495056 | GeneCards |
| HSALNG0114029 | 0.402495056 | GeneCards |
| HSALNG0131671 | 0.402495056 | GeneCards |
| KR606819 | 0.402495056 | GeneCards |
| L13714-460 | 0.402495056 | GeneCards |
| lnc-IL1RL1-1 | 0.402495056 | GeneCards |
| lnc-LRRC37A-1 | 0.402495056 | GeneCards |
| lnc-ORMDL3-1 | 0.402495056 | GeneCards |
| lnc-TOMM70-2 | 0.402495056 | GeneCards |
| piR-31199-235 | 0.402495056 | GeneCards |
| piR-32214-616 | 0.402495056 | GeneCards |
| piR-32214-667 | 0.402495056 | GeneCards |
| piR-32382 | 0.402495056 | GeneCards |
| piR-32810-092 | 0.402495056 | GeneCards |
| piR-33103-021 | 0.402495056 | GeneCards |
| piR-33211-004 | 0.402495056 | GeneCards |
| piR-34822-025 | 0.402495056 | GeneCards |
| piR-36362-005 | 0.402495056 | GeneCards |
| piR-36588-111 | 0.402495056 | GeneCards |
| piR-36811 | 0.402495056 | GeneCards |
| piR-41141-039 | 0.402495056 | GeneCards |
| piR-46501-051 | 0.402495056 | GeneCards |
| piR-48282 | 0.402495056 | GeneCards |
| piR-48389-015 | 0.402495056 | GeneCards |
| piR-48799-021 | 0.402495056 | GeneCards |
| piR-49400-009 | 0.402495056 | GeneCards |
| piR-56480-020 | 0.402495056 | GeneCards |
| piR-56883-134 | 0.402495056 | GeneCards |
| RF00017-5086 | 0.402495056 | GeneCards |
| RF00017-5467 | 0.402495056 | GeneCards |
| RF00026-525 | 0.402495056 | GeneCards |
| RF00994-269 | 0.402495056 | GeneCards |
| HSALNG0069599 | 0.402495056 | GeneCards |
| HSALNG0069871 | 0.402495056 | GeneCards |
| HSALNG0069896 | 0.402495056 | GeneCards |
| HSALNG0070261 | 0.402495056 | GeneCards |
| HSALNG0076890 | 0.402495056 | GeneCards |
| HSALNG0082411 | 0.402495056 | GeneCards |
| HSALNG0082725 | 0.402495056 | GeneCards |
| HSALNG0083272 | 0.402495056 | GeneCards |
| HSALNG0083656-001 | 0.402495056 | GeneCards |
| HSALNG0092788 | 0.402495056 | GeneCards |
| HSALNG0094047 | 0.402495056 | GeneCards |
| HSALNG0096249 | 0.402495056 | GeneCards |
| HSALNG0098914 | 0.402495056 | GeneCards |
| HSALNG0102258 | 0.402495056 | GeneCards |
| HSALNG0103933 | 0.402495056 | GeneCards |
| HSALNG0108167 | 0.402495056 | GeneCards |
| HSALNG0133918 | 0.402495056 | GeneCards |
| KR153194-092 | 0.402495056 | GeneCards |
| lnc-CD58-8 | 0.402495056 | GeneCards |
| lnc-CHIC2-4 | 0.402495056 | GeneCards |
| lnc-DFFB-4 | 0.402495056 | GeneCards |
| lnc-MYLIP-5 | 0.402495056 | GeneCards |
| lnc-SCAF8-2 | 0.402495056 | GeneCards |
| NONHSAG016644.2 | 0.402495056 | GeneCards |
| NONHSAG023893.2 | 0.402495056 | GeneCards |
| piR-33804-136 | 0.402495056 | GeneCards |
| piR-34313-004 | 0.402495056 | GeneCards |
| piR-35031-038 | 0.402495056 | GeneCards |
| piR-36252-004 | 0.402495056 | GeneCards |
| piR-36365-003 | 0.402495056 | GeneCards |
| piR-36365-004 | 0.402495056 | GeneCards |
| piR-37560 | 0.402495056 | GeneCards |
| piR-38512-003 | 0.402495056 | GeneCards |
| piR-38519-002 | 0.402495056 | GeneCards |
| piR-38872-001 | 0.402495056 | GeneCards |
| piR-39858-341 | 0.402495056 | GeneCards |
| piR-39972-009 | 0.402495056 | GeneCards |
| piR-40732-005 | 0.402495056 | GeneCards |
| piR-41550 | 0.402495056 | GeneCards |
| piR-42376 | 0.402495056 | GeneCards |
| piR-42531 | 0.402495056 | GeneCards |
| piR-42777 | 0.402495056 | GeneCards |
| piR-44452 | 0.402495056 | GeneCards |
| piR-49423-027 | 0.402495056 | GeneCards |
| piR-50437-125 | 0.402495056 | GeneCards |
| piR-50443-325 | 0.402495056 | GeneCards |
| piR-51166 | 0.402495056 | GeneCards |
| piR-52966-015 | 0.402495056 | GeneCards |
| piR-57461-188 | 0.402495056 | GeneCards |
| piR-58297-114 | 0.402495056 | GeneCards |
| piR-58557-058 | 0.402495056 | GeneCards |
| piR-61265 | 0.402495056 | GeneCards |
| RF00017-7289 | 0.402495056 | GeneCards |
| piR-57394-169 | 0.402495056 | GeneCards |
| piR-58488 | 0.402495056 | GeneCards |
| piR-61293 | 0.402495056 | GeneCards |
| RF00017-4183 | 0.402495056 | GeneCards |
| RF00026-098 | 0.402495056 | GeneCards |
| RF00026-399 | 0.402495056 | GeneCards |
| RF00994-1108 | 0.402495056 | GeneCards |
| RF00994-257 | 0.402495056 | GeneCards |
| RF01045-010 | 0.402495056 | GeneCards |
| LOC105374172 | 0.402495056 | GeneCards |
| LOC105374412 | 0.402495056 | GeneCards |
| LOC105370236 | 0.402495056 | GeneCards |
| ENSG00000248281 | 0.402495056 | GeneCards |
| ENSG00000273844 | 0.402495056 | GeneCards |
| ENSG00000248725 | 0.402495056 | GeneCards |
| ENSG00000255853 | 0.402495056 | GeneCards |
| ENSG00000286243 | 0.402495056 | GeneCards |
| ENSG00000258358 | 0.402495056 | GeneCards |
| ENSG00000270328 | 0.402495056 | GeneCards |
| ENSG00000258619 | 0.402495056 | GeneCards |
| ENSG00000278593 | 0.402495056 | GeneCards |
| ENSG00000225253 | 0.402495056 | GeneCards |
| ENSG00000237434 | 0.402495056 | GeneCards |
| HSALNG0014786 | 0.402495056 | GeneCards |
| LOC105375959 | 0.402495056 | GeneCards |
| LOC105372228 | 0.402495056 | GeneCards |
| HSALNG0049462 | 0.402495056 | GeneCards |
| 5EW4_A-012 | 0.402495056 | GeneCards |
| HSALNG0007230 | 0.402495056 | GeneCards |
| HSALNG0007231 | 0.402495056 | GeneCards |
| HSALNG0000375 | 0.402495056 | GeneCards |
| HSALNG0059262-002 | 0.402495056 | GeneCards |
| HSALNG0017870 | 0.402495056 | GeneCards |
| HSALNG0031876 | 0.402495056 | GeneCards |
| HSALNG0031877 | 0.402495056 | GeneCards |
| piR-51247-002 | 0.402495056 | GeneCards |
| piR-51878-031 | 0.402495056 | GeneCards |
| HSALNG0116290 | 0.402495056 | GeneCards |
| HSALNG0116311 | 0.402495056 | GeneCards |
| HSALNG0134393 | 0.402495056 | GeneCards |
| NONHSAG026010.2 | 0.402495056 | GeneCards |
| piR-35002-057 | 0.402495056 | GeneCards |
| piR-60051-098 | 0.402495056 | GeneCards |
| piR-32023-024 | 0.402495056 | GeneCards |
| piR-48348-061 | 0.402495056 | GeneCards |
| piR-48759-260 | 0.402495056 | GeneCards |
| piR-56612-008 | 0.402495056 | GeneCards |
| NONHSAG023607.2-002 | 0.402495056 | GeneCards |
| piR-41562 | 0.402495056 | GeneCards |
| piR-42613-003 | 0.402495056 | GeneCards |
| RF00017-2520 | 0.402495056 | GeneCards |
| TNF | - | GeneMap |
| TBX21 | - | GeneMap |
| SCGB3A2 | - | GeneMap |
| PTGER2 | - | GeneMap |
| PTGDR | - | GeneMap |
| PLA2G7 | - | GeneMap |
| NPSR1 | - | GeneMap |
| MUC7 | - | GeneMap |
| IRAK3 | - | GeneMap |
| IL13 | - | GeneMap |
| IGES | - | GeneMap |
| HNMT | - | GeneMap |
| HLA-G | - | GeneMap |
| CHI3L1 | - | GeneMap |
| CCL11 | - | GeneMap |
| ASRT8 | - | GeneMap |
| ASRT6 | - | GeneMap |
| ASRT4 | - | GeneMap |
| ASRT3 | - | GeneMap |
| ALOX5 | - | GeneMap |
| ADRB2 | - | GeneMap |
| UGRP1 | - | GeneMap |
| TNFA | - | GeneMap |
| TBET | - | GeneMap |
| SCYA11 | - | GeneMap |
| PAFAH | - | GeneMap |
| MRT51 | - | GeneMap |
| IRAKM | - | GeneMap |
| GPR154 | - | GeneMap |
| GP39 | - | GeneMap |
| AS1 | - | GeneMap |
| ALRH | - | GeneMap |
| YKL40 | - | GeneMap |
| PAFAD | - | GeneMap |
| GPRA | - | GeneMap |
| BHR1 | - | GeneMap |
| ASRT5 | - | GeneMap |
| ASRT1 | - | GeneMap |
| VRR1 | - | GeneMap |
| ASRT7 | - | GeneMap |
| PGR14 | - | GeneMap |
| ASRT2 | - | GeneMap |
| TBX21 | 0.7 | DisGeNET |
| IL5 | 0.7 | DisGeNET |
| IL13 | 0.7 | DisGeNET |
| CCL11 | 0.7 | DisGeNET |
| TGFB1 | 0.7 | DisGeNET |
| ICAM1 | 0.6 | DisGeNET |
| IL4 | 0.6 | DisGeNET |
| IL6 | 0.6 | DisGeNET |
| MMP9 | 0.6 | DisGeNET |
| NOS2 | 0.6 | DisGeNET |
| CCL2 | 0.6 | DisGeNET |
| CCL5 | 0.6 | DisGeNET |
| SCGB3A2 | 0.5 | DisGeNET |
| ADRB2 | 0.5 | DisGeNET |
| ALOX5 | 0.5 | DisGeNET |
| HLA-DQB1 | 0.5 | DisGeNET |
| HLA-DRB1 | 0.5 | DisGeNET |
| HLA-G | 0.5 | DisGeNET |
| GSDMB | 0.5 | DisGeNET |
| TNF | 0.5 | DisGeNET |
| TSLP | 0.5 | DisGeNET |
| IL33 | 0.5 | DisGeNET |
| IL1RL1 | 0.5 | DisGeNET |
| ORMDL3 | 0.5 | DisGeNET |
| HLA-DQA1 | 0.49 | DisGeNET |
| IL6R | 0.49 | DisGeNET |
| RAD50 | 0.48 | DisGeNET |
| PDE4D | 0.47 | DisGeNET |
| IKZF3 | 0.46 | DisGeNET |
| HNMT | 0.46 | DisGeNET |
| CDHR3 | 0.45 | DisGeNET |
| KIF3A | 0.44 | DisGeNET |
| PYHIN1 | 0.43 | DisGeNET |
| MUC7 | 0.43 | DisGeNET |
| PLA2G7 | 0.43 | DisGeNET |
| DNAH5 | 0.42 | DisGeNET |
| TNIP1 | 0.41 | DisGeNET |
| WDR36 | 0.41 | DisGeNET |
| PTGDR2 | 0.4 | DisGeNET |
| CCR3 | 0.4 | DisGeNET |
| PARP1 | 0.4 | DisGeNET |
| EDN1 | 0.4 | DisGeNET |
| GATA3 | 0.4 | DisGeNET |
| GSTM1 | 0.4 | DisGeNET |
| GSTP1 | 0.4 | DisGeNET |
| HLA-DPB1 | 0.4 | DisGeNET |
| TNC | 0.4 | DisGeNET |
| IL1B | 0.4 | DisGeNET |
| IL1RN | 0.4 | DisGeNET |
| IL2RA | 0.4 | DisGeNET |
| IL4R | 0.4 | DisGeNET |
| ARG1 | 0.4 | DisGeNET |
| NPSR1 | 0.4 | DisGeNET |
| RNASE3 | 0.4 | DisGeNET |
| STAT6 | 0.4 | DisGeNET |
| VEGFA | 0.4 | DisGeNET |
| CAT | 0.4 | DisGeNET |
| CD14 | 0.4 | DisGeNET |
| ARG2 | 0.39 | DisGeNET |
| AREG | 0.38 | DisGeNET |
| NQO1 | 0.37 | DisGeNET |
| MMP1 | 0.37 | DisGeNET |
| ALDH2 | 0.36 | DisGeNET |
| CTNNA3 | 0.36 | DisGeNET |
| SOD1 | 0.36 | DisGeNET |
| BCL2 | 0.35 | DisGeNET |
| RUNX3 | 0.35 | DisGeNET |
| HMOX1 | 0.34 | DisGeNET |
| PLAU | 0.34 | DisGeNET |
| TRPA1 | 0.34 | DisGeNET |
| IFNL3 | 0.33 | DisGeNET |
| NPY | 0.33 | DisGeNET |
| PTEN | 0.33 | DisGeNET |
| MMP10 | 0.32 | DisGeNET |
| ADCY2 | 0.31 | DisGeNET |
| ADCYAP1R1 | 0.31 | DisGeNET |
| MYB | 0.31 | DisGeNET |
| BGLAP | 0.31 | DisGeNET |
| SPRR2B | 0.31 | DisGeNET |
| TIMP3 | 0.31 | DisGeNET |
| CCL26 | 0.3 | DisGeNET |
| DNMT1 | 0.3 | DisGeNET |
| EGFR | 0.3 | DisGeNET |
| NR3C1 | 0.3 | DisGeNET |
| HSD11B2 | 0.3 | DisGeNET |
| IL10 | 0.3 | DisGeNET |
| IL17A | 0.3 | DisGeNET |
| KRT19 | 0.3 | DisGeNET |
| MUC5AC | 0.3 | DisGeNET |
| SERPINE1 | 0.3 | DisGeNET |
| IL22 | 0.3 | DisGeNET |
| PDE4B | 0.3 | DisGeNET |
| SERPINA1 | 0.3 | DisGeNET |
| PPP2CA | 0.3 | DisGeNET |
| PTGS2 | 0.3 | DisGeNET |
| DPP10 | 0.3 | DisGeNET |
| CCL17 | 0.3 | DisGeNET |
| SCGB1A1 | 0.3 | DisGeNET |
| CXCL14 | 0.3 | DisGeNET |
| AGER | 0.29 | DisGeNET |
| SFTPD | 0.29 | DisGeNET |
| STAT4 | 0.29 | DisGeNET |
| CCR4 | 0.28 | DisGeNET |
| LTA4H | 0.28 | DisGeNET |
| CCL22 | 0.27 | DisGeNET |
| C5AR1 | 0.27 | DisGeNET |
| TLR3 | 0.26 | DisGeNET |
| PIN1 | 0.25 | DisGeNET |
| C3AR1 | 0.25 | DisGeNET |
| CD40 | 0.25 | DisGeNET |
| ESR2 | 0.24 | DisGeNET |
| PRMT1 | 0.24 | DisGeNET |
| FAS | 0.23 | DisGeNET |
| PLA2G4A | 0.23 | DisGeNET |
| PTGER3 | 0.23 | DisGeNET |
| TACR1 | 0.23 | DisGeNET |
| C5 | 0.23 | DisGeNET |
| VIP | 0.23 | DisGeNET |
| CD86 | 0.23 | DisGeNET |
| EDNRB | 0.22 | DisGeNET |
| RHOA | 0.22 | DisGeNET |
| MIF | 0.22 | DisGeNET |
| NTRK1 | 0.22 | DisGeNET |
| SFTPA1 | 0.22 | DisGeNET |
| UTS2 | 0.21 | DisGeNET |
| CCR8 | 0.21 | DisGeNET |
| AGTR1 | 0.21 | DisGeNET |
| AGTR2 | 0.21 | DisGeNET |
| ITPR1 | 0.21 | DisGeNET |
| PDGFB | 0.21 | DisGeNET |
| SFTPB | 0.21 | DisGeNET |
| UCN | 0.21 | DisGeNET |
| PRMT3 | 0.2 | DisGeNET |
| PRMT5 | 0.2 | DisGeNET |
| CARM1 | 0.2 | DisGeNET |
| CFTR | 0.2 | DisGeNET |
| SPINK5 | 0.2 | DisGeNET |
| CHAT | 0.2 | DisGeNET |
| CSF2RB | 0.2 | DisGeNET |
| DCN | 0.2 | DisGeNET |
| F7 | 0.2 | DisGeNET |
| MS4A2 | 0.2 | DisGeNET |
| FGA | 0.2 | DisGeNET |
| FLG | 0.2 | DisGeNET |
| PRMT2 | 0.2 | DisGeNET |
| ITGB6 | 0.2 | DisGeNET |
| PIK3CD | 0.2 | DisGeNET |
| PRF1 | 0.2 | DisGeNET |
| SLC5A7 | 0.2 | DisGeNET |
| BDKRB2 | 0.2 | DisGeNET |
| SLC18A3 | 0.2 | DisGeNET |
| SLC22A1 | 0.2 | DisGeNET |
| STAT3 | 0.2 | DisGeNET |
| IL18R1 | 0.2 | DisGeNET |
| ZPBP2 | 0.18 | DisGeNET |
| NOD2 | 0.18 | DisGeNET |
| TNFSF4 | 0.18 | DisGeNET |
| TLR6 | 0.17 | DisGeNET |
| GSDMA | 0.17 | DisGeNET |
| TLR10 | 0.17 | DisGeNET |
| SMAD3 | 0.16 | DisGeNET |
| ATG5 | 0.16 | DisGeNET |
| ADA | 0.15 | DisGeNET |
| IL1R1 | 0.15 | DisGeNET |
| DENND1B | 0.14 | DisGeNET |
| FCGR2A | 0.14 | DisGeNET |
| ICOS | 0.14 | DisGeNET |
| IL2RB | 0.14 | DisGeNET |
| TLR1 | 0.14 | DisGeNET |
| ADORA1 | 0.13 | DisGeNET |
| IL23R | 0.13 | DisGeNET |
| JAG1 | 0.13 | DisGeNET |
| ICOSLG | 0.13 | DisGeNET |
| IL7R | 0.13 | DisGeNET |
| IRF4 | 0.13 | DisGeNET |
| ITGAX | 0.13 | DisGeNET |
| PTGER2 | 0.13 | DisGeNET |
| SLC22A5 | 0.13 | DisGeNET |
| NKX2-1 | 0.13 | DisGeNET |
| COL1A1 | 0.12 | DisGeNET |
| ERBB2 | 0.12 | DisGeNET |
| MTOR | 0.12 | DisGeNET |
| GAB1 | 0.12 | DisGeNET |
| DNAI1 | 0.12 | DisGeNET |
| DLL1 | 0.12 | DisGeNET |
| DNAAF3 | 0.12 | DisGeNET |
| ITLN1 | 0.12 | DisGeNET |
| EMSY | 0.12 | DisGeNET |
| BACH2 | 0.12 | DisGeNET |
| RORA | 0.12 | DisGeNET |
| CASP8 | 0.12 | DisGeNET |
| KIAA1109 | 0.12 | DisGeNET |
| GPR65 | 0.12 | DisGeNET |
| PGAP3 | 0.12 | DisGeNET |
| FADS2 | 0.12 | DisGeNET |
| MED24 | 0.12 | DisGeNET |
| CDSN | 0.11 | DisGeNET |
| PSORS1C1 | 0.11 | DisGeNET |
| ETS1 | 0.11 | DisGeNET |
| ABCD1 | 0.11 | DisGeNET |
| FCER1G | 0.11 | DisGeNET |
| FBXL7 | 0.11 | DisGeNET |
| CLEC16A | 0.11 | DisGeNET |
| NEDD4L | 0.11 | DisGeNET |
| PTPN22 | 0.11 | DisGeNET |
| GLI2 | 0.11 | DisGeNET |
| HLA-B | 0.11 | DisGeNET |
| HLA-DPA1 | 0.11 | DisGeNET |
| HLA-DRB5 | 0.11 | DisGeNET |
| KLK3 | 0.11 | DisGeNET |
| ITPR3 | 0.11 | DisGeNET |
| KRT7 | 0.11 | DisGeNET |
| FADS1 | 0.11 | DisGeNET |
| LPP | 0.11 | DisGeNET |
| MICB | 0.11 | DisGeNET |
| NOTCH4 | 0.11 | DisGeNET |
| PBX2 | 0.11 | DisGeNET |
| CRIM1 | 0.11 | DisGeNET |
| PLEK | 0.11 | DisGeNET |
| CCHCR1 | 0.11 | DisGeNET |
| ALLC | 0.11 | DisGeNET |
| PROC | 0.11 | DisGeNET |
| PSMD3 | 0.11 | DisGeNET |
| PTCH1 | 0.11 | DisGeNET |
| PTPRC | 0.11 | DisGeNET |
| RAD51B | 0.11 | DisGeNET |
| HAS2-AS1 | 0.11 | DisGeNET |
| SLC6A12 | 0.11 | DisGeNET |
| TBX1 | 0.11 | DisGeNET |
| TNS1 | 0.11 | DisGeNET |
| NDFIP1 | 0.11 | DisGeNET |
| DOCK8 | 0.11 | DisGeNET |
| RUNX1 | 0.11 | DisGeNET |
| DNAH11 | 0.11 | DisGeNET |
| TNFRSF11A | 0.11 | DisGeNET |
| IL18RAP | 0.11 | DisGeNET |
| PTGES | 0.11 | DisGeNET |
| ZNF432 | 0.11 | DisGeNET |
| LRBA | 0.11 | DisGeNET |
| TNFSF15 | 0.11 | DisGeNET |
| CCNT2-AS1 | 0.1 | DisGeNET |
| FAM205C | 0.1 | DisGeNET |
| LINC01387 | 0.1 | DisGeNET |
| MAP3K14-AS1 | 0.1 | DisGeNET |
| RNU4ATAC | 0.1 | DisGeNET |
| MIR3681HG | 0.1 | DisGeNET |
| MROH7-TTC4 | 0.1 | DisGeNET |
| ATP6V1G2-DDX39B | 0.1 | DisGeNET |
| RTEL1-TNFRSF6B | 0.1 | DisGeNET |
| FMC1-LUC7L2 | 0.1 | DisGeNET |
| CDH17 | 0.1 | DisGeNET |
| CDK2 | 0.1 | DisGeNET |
| LINC01934 | 0.1 | DisGeNET |
| TH2LCRR | 0.1 | DisGeNET |
| LINC01425 | 0.1 | DisGeNET |
| LINC02660 | 0.1 | DisGeNET |
| LINC01527 | 0.1 | DisGeNET |
| IL12A-AS1 | 0.1 | DisGeNET |
| LINC01149 | 0.1 | DisGeNET |
| TSBP1-AS1 | 0.1 | DisGeNET |
| MMADHC-DT | 0.1 | DisGeNET |
| LINC01615 | 0.1 | DisGeNET |
| LINC01063 | 0.1 | DisGeNET |
| CCNO | 0.1 | DisGeNET |
| MRVI1 | 0.1 | DisGeNET |
| VAV3 | 0.1 | DisGeNET |
| LINC02471 | 0.1 | DisGeNET |
| LINC01862 | 0.1 | DisGeNET |
| SOCAR | 0.1 | DisGeNET |
| LINC02121 | 0.1 | DisGeNET |
| MPPED2-AS1 | 0.1 | DisGeNET |
| CEL | 0.1 | DisGeNET |
| AHSA1 | 0.1 | DisGeNET |
| POSTN | 0.1 | DisGeNET |
| TSBP1 | 0.1 | DisGeNET |
| MTCO2P12 | 0.1 | DisGeNET |
| CETP | 0.1 | DisGeNET |
| RNU1-79P | 0.1 | DisGeNET |
| RNU1-116P | 0.1 | DisGeNET |
| LRRK2-DT | 0.1 | DisGeNET |
| CYSLTR1 | 0.1 | DisGeNET |
| KRT7-AS | 0.1 | DisGeNET |
| SLC27A5 | 0.1 | DisGeNET |
| TDRKH | 0.1 | DisGeNET |
| FGFR1OP | 0.1 | DisGeNET |
| CHI3L1 | 0.1 | DisGeNET |
| POLI | 0.1 | DisGeNET |
| IL17F | 0.1 | DisGeNET |
| GLCCI1 | 0.1 | DisGeNET |
| ACOT7 | 0.1 | DisGeNET |
| CHRNA2 | 0.1 | DisGeNET |
| NLRP3 | 0.1 | DisGeNET |
| ZNF618 | 0.1 | DisGeNET |
| LRRC56 | 0.1 | DisGeNET |
| UHRF2 | 0.1 | DisGeNET |
| CCDC151 | 0.1 | DisGeNET |
| RMI2 | 0.1 | DisGeNET |
| TAGAP | 0.1 | DisGeNET |
| PARD3B | 0.1 | DisGeNET |
| CLCA1 | 0.1 | DisGeNET |
| SERPINA3 | 0.1 | DisGeNET |
| LRRK2 | 0.1 | DisGeNET |
| BTBD11 | 0.1 | DisGeNET |
| CCR5 | 0.1 | DisGeNET |
| CCR6 | 0.1 | DisGeNET |
| DNAAF1 | 0.1 | DisGeNET |
| SPATA32 | 0.1 | DisGeNET |
| COL5A1 | 0.1 | DisGeNET |
| COL5A2 | 0.1 | DisGeNET |
| RBM45 | 0.1 | DisGeNET |
| ACMSD | 0.1 | DisGeNET |
| AP1S3 | 0.1 | DisGeNET |
| COMT | 0.1 | DisGeNET |
| SLCO6A1 | 0.1 | DisGeNET |
| UBE2QL1 | 0.1 | DisGeNET |
| PIH1D3 | 0.1 | DisGeNET |
| CRK | 0.1 | DisGeNET |
| CRP | 0.1 | DisGeNET |
| ZBTB46 | 0.1 | DisGeNET |
| ADAMTS14 | 0.1 | DisGeNET |
| MPP7 | 0.1 | DisGeNET |
| MAPK14 | 0.1 | DisGeNET |
| CSF2 | 0.1 | DisGeNET |
| GLT1D1 | 0.1 | DisGeNET |
| LACC1 | 0.1 | DisGeNET |
| CARMIL2 | 0.1 | DisGeNET |
| CTLA4 | 0.1 | DisGeNET |
| CTNNB1 | 0.1 | DisGeNET |
| LINC01931 | 0.1 | DisGeNET |
| PUS10 | 0.1 | DisGeNET |
| LINC02085 | 0.1 | DisGeNET |
| JAKMIP1 | 0.1 | DisGeNET |
| PLEKHG4B | 0.1 | DisGeNET |
| CCDC167 | 0.1 | DisGeNET |
| SAXO1 | 0.1 | DisGeNET |
| SLC5A12 | 0.1 | DisGeNET |
| DGKH | 0.1 | DisGeNET |
| AP2A2 | 0.1 | DisGeNET |
| DNAAF4 | 0.1 | DisGeNET |
| EXD1 | 0.1 | DisGeNET |
| ZFPM1 | 0.1 | DisGeNET |
| ACE | 0.1 | DisGeNET |
| PRICKLE2 | 0.1 | DisGeNET |
| PRSS35 | 0.1 | DisGeNET |
| SLC30A8 | 0.1 | DisGeNET |
| DPH1 | 0.1 | DisGeNET |
| DPP6 | 0.1 | DisGeNET |
| EDNRA | 0.1 | DisGeNET |
| AHR | 0.1 | DisGeNET |
| LINC00528 | 0.1 | DisGeNET |
| CMYA5 | 0.1 | DisGeNET |
| EPHA5 | 0.1 | DisGeNET |
| ERBB3 | 0.1 | DisGeNET |
| ERBB4 | 0.1 | DisGeNET |
| ERCC2 | 0.1 | DisGeNET |
| F2RL1 | 0.1 | DisGeNET |
| TMEM26 | 0.1 | DisGeNET |
| FCER2 | 0.1 | DisGeNET |
| JMJD1C | 0.1 | DisGeNET |
| RSPH9 | 0.1 | DisGeNET |
| JAZF1 | 0.1 | DisGeNET |
| FER | 0.1 | DisGeNET |
| FGF8 | 0.1 | DisGeNET |
| FGFR1 | 0.1 | DisGeNET |
| ZNF652 | 0.1 | DisGeNET |
| CLSTN1 | 0.1 | DisGeNET |
| ZNF365 | 0.1 | DisGeNET |
| SBNO2 | 0.1 | DisGeNET |
| SEPTIN8 | 0.1 | DisGeNET |
| SIK2 | 0.1 | DisGeNET |
| CLASP1 | 0.1 | DisGeNET |
| SIK3 | 0.1 | DisGeNET |
| ZFPM2 | 0.1 | DisGeNET |
| CRB1 | 0.1 | DisGeNET |
| LRRC6 | 0.1 | DisGeNET |
| KCNE4 | 0.1 | DisGeNET |
| ALOX5AP | 0.1 | DisGeNET |
| GAS2L2 | 0.1 | DisGeNET |
| ZBTB38 | 0.1 | DisGeNET |
| ZDHHC24 | 0.1 | DisGeNET |
| GABPA | 0.1 | DisGeNET |
| TNFAIP8 | 0.1 | DisGeNET |
| RNF19A | 0.1 | DisGeNET |
| DNAH1 | 0.1 | DisGeNET |
| COPD | 0.1 | DisGeNET |
| POLDIP2 | 0.1 | DisGeNET |
| PHF19 | 0.1 | DisGeNET |
| RGS22 | 0.1 | DisGeNET |
| GAS1 | 0.1 | DisGeNET |
| PITPNC1 | 0.1 | DisGeNET |
| GAS8 | 0.1 | DisGeNET |
| HLA-DPA3 | 0.1 | DisGeNET |
| HAVCR1 | 0.1 | DisGeNET |
| STK36 | 0.1 | DisGeNET |
| CHIA | 0.1 | DisGeNET |
| IL37 | 0.1 | DisGeNET |
| GLB1 | 0.1 | DisGeNET |
| HPGDS | 0.1 | DisGeNET |
| GLDC | 0.1 | DisGeNET |
| GNAI1 | 0.1 | DisGeNET |
| GNAS | 0.1 | DisGeNET |
| GP1BA | 0.1 | DisGeNET |
| GP1BB | 0.1 | DisGeNET |
| GP9 | 0.1 | DisGeNET |
| ZNF311 | 0.1 | DisGeNET |
| EIF2S2P3 | 0.1 | DisGeNET |
| LINC00907 | 0.1 | DisGeNET |
| ZNF841 | 0.1 | DisGeNET |
| LINC01091 | 0.1 | DisGeNET |
| XKR6 | 0.1 | DisGeNET |
| ILDR1 | 0.1 | DisGeNET |
| GRID1 | 0.1 | DisGeNET |
| GSTT1 | 0.1 | DisGeNET |
| PDZRN4 | 0.1 | DisGeNET |
| HDAC2 | 0.1 | DisGeNET |
| HLA-DPB2 | 0.1 | DisGeNET |
| HLA-DQA2 | 0.1 | DisGeNET |
| HLA-DRB3 | 0.1 | DisGeNET |
| HLA-DRB9 | 0.1 | DisGeNET |
| HMGB1 | 0.1 | DisGeNET |
| UBAC2 | 0.1 | DisGeNET |
| FLG-AS1 | 0.1 | DisGeNET |
| LINC00299 | 0.1 | DisGeNET |
| CCDC39 | 0.1 | DisGeNET |
| OTOG | 0.1 | DisGeNET |
| IDS | 0.1 | DisGeNET |
| IFNA1 | 0.1 | DisGeNET |
| DUBR | 0.1 | DisGeNET |
| IFNA13 | 0.1 | DisGeNET |
| IFNB1 | 0.1 | DisGeNET |
| IRGM | 0.1 | DisGeNET |
| MCIDAS | 0.1 | DisGeNET |
| ACTBL2 | 0.1 | DisGeNET |
| IFNG | 0.1 | DisGeNET |
| RSPH4A | 0.1 | DisGeNET |
| FTCDNL1 | 0.1 | DisGeNET |
| IGHE | 0.1 | DisGeNET |
| IL1A | 0.1 | DisGeNET |
| IL2 | 0.1 | DisGeNET |
| IL3 | 0.1 | DisGeNET |
| IL5RA | 0.1 | DisGeNET |
| CXCL8 | 0.1 | DisGeNET |
| IL9 | 0.1 | DisGeNET |
| IL12B | 0.1 | DisGeNET |
| IL18 | 0.1 | DisGeNET |
| CXCL10 | 0.1 | DisGeNET |
| ISG20 | 0.1 | DisGeNET |
| ITGB8 | 0.1 | DisGeNET |
| ITPKA | 0.1 | DisGeNET |
| GSTK1 | 0.1 | DisGeNET |
| DNAJB13 | 0.1 | DisGeNET |
| MROH7 | 0.1 | DisGeNET |
| NRROS | 0.1 | DisGeNET |
| KNG1 | 0.1 | DisGeNET |
| KIF11 | 0.1 | DisGeNET |
| CCDC103 | 0.1 | DisGeNET |
| KRT81 | 0.1 | DisGeNET |
| C4orf50 | 0.1 | DisGeNET |
| LEP | 0.1 | DisGeNET |
| LIFR | 0.1 | DisGeNET |
| LIG4 | 0.1 | DisGeNET |
| CASC15 | 0.1 | DisGeNET |
| GTF2H5 | 0.1 | DisGeNET |
| LTA | 0.1 | DisGeNET |
| LTC4S | 0.1 | DisGeNET |
| MIR146A | 0.1 | DisGeNET |
| MIR155 | 0.1 | DisGeNET |
| MANBA | 0.1 | DisGeNET |
| HCG18 | 0.1 | DisGeNET |
| HCG17 | 0.1 | DisGeNET |
| MBL2 | 0.1 | DisGeNET |
| ARVCF | 0.1 | DisGeNET |
| MLLT3 | 0.1 | DisGeNET |
| POTEKP | 0.1 | DisGeNET |
| IRF1-AS1 | 0.1 | DisGeNET |
| MST1 | 0.1 | DisGeNET |
| PLF | 0.1 | DisGeNET |
| COX2 | 0.1 | DisGeNET |
| MYLK | 0.1 | DisGeNET |
| PPP1R12B | 0.1 | DisGeNET |
| RERE | 0.1 | DisGeNET |
| NFE2L2 | 0.1 | DisGeNET |
| NFKB2 | 0.1 | DisGeNET |
| NGF | 0.1 | DisGeNET |
| NODAL | 0.1 | DisGeNET |
| NOS1 | 0.1 | DisGeNET |
| NOS3 | 0.1 | DisGeNET |
| OAS2 | 0.1 | DisGeNET |
| ORM1 | 0.1 | DisGeNET |
| OVOL1 | 0.1 | DisGeNET |
| ITSN2 | 0.1 | DisGeNET |
| CDON | 0.1 | DisGeNET |
| PDE11A | 0.1 | DisGeNET |
| FOXP3 | 0.1 | DisGeNET |
| WDPCP | 0.1 | DisGeNET |
| NME8 | 0.1 | DisGeNET |
| ZMYND10 | 0.1 | DisGeNET |
| PDE4A | 0.1 | DisGeNET |
| DDX41 | 0.1 | DisGeNET |
| HDAC7 | 0.1 | DisGeNET |
| LUC7L2 | 0.1 | DisGeNET |
| SUFU | 0.1 | DisGeNET |
| SLC26A4 | 0.1 | DisGeNET |
| RAPGEF6 | 0.1 | DisGeNET |
| RTEL1 | 0.1 | DisGeNET |
| PEPD | 0.1 | DisGeNET |
| PEX14 | 0.1 | DisGeNET |
| PGM3 | 0.1 | DisGeNET |
| PIK3CA | 0.1 | DisGeNET |
| PIK3CB | 0.1 | DisGeNET |
| PIK3CG | 0.1 | DisGeNET |
| TLR9 | 0.1 | DisGeNET |
| MIR4435-2HG | 0.1 | DisGeNET |
| OTULINL | 0.1 | DisGeNET |
| MED1 | 0.1 | DisGeNET |
| FEV | 0.1 | DisGeNET |
| HYDIN | 0.1 | DisGeNET |
| BNC2 | 0.1 | DisGeNET |
| NSUN2 | 0.1 | DisGeNET |
| CDKAL1 | 0.1 | DisGeNET |
| DNAAF5 | 0.1 | DisGeNET |
| PIGX | 0.1 | DisGeNET |
| CCDC40 | 0.1 | DisGeNET |
| ATG16L1 | 0.1 | DisGeNET |
| ARMC4 | 0.1 | DisGeNET |
| DNAAF2 | 0.1 | DisGeNET |
| VPS13D | 0.1 | DisGeNET |
| KIF16B | 0.1 | DisGeNET |
| TENM3 | 0.1 | DisGeNET |
| LRP2BP | 0.1 | DisGeNET |
| PRKCE | 0.1 | DisGeNET |
| PRKCQ | 0.1 | DisGeNET |
| PRKG1 | 0.1 | DisGeNET |
| MAPK1 | 0.1 | DisGeNET |
| MYDGF | 0.1 | DisGeNET |
| BTNL2 | 0.1 | DisGeNET |
| KCNQ5 | 0.1 | DisGeNET |
| CFAP298 | 0.1 | DisGeNET |
| ADAMTS9 | 0.1 | DisGeNET |
| ACKR3 | 0.1 | DisGeNET |
| CYSLTR2 | 0.1 | DisGeNET |
| ZMIZ1 | 0.1 | DisGeNET |
| PTGDR | 0.1 | DisGeNET |
| PTGDS | 0.1 | DisGeNET |
| COG6 | 0.1 | DisGeNET |
| ARHGAP31 | 0.1 | DisGeNET |
| PTPN2 | 0.1 | DisGeNET |
| PTPRK | 0.1 | DisGeNET |
| BBS1 | 0.1 | DisGeNET |
| PEX5 | 0.1 | DisGeNET |
| RAB5B | 0.1 | DisGeNET |
| EEFSEC | 0.1 | DisGeNET |
| RPGR | 0.1 | DisGeNET |
| RREB1 | 0.1 | DisGeNET |
| BDNF | 0.1 | DisGeNET |
| ATXN2 | 0.1 | DisGeNET |
| SCN4A | 0.1 | DisGeNET |
| CCL24 | 0.1 | DisGeNET |
| SDHD | 0.1 | DisGeNET |
| POTEM | 0.1 | DisGeNET |
| IKZF4 | 0.1 | DisGeNET |
| GALNT17 | 0.1 | DisGeNET |
| DNAI2 | 0.1 | DisGeNET |
| SHH | 0.1 | DisGeNET |
| MROH3P | 0.1 | DisGeNET |
| IL25 | 0.1 | DisGeNET |
| PMEL | 0.1 | DisGeNET |
| SIX3 | 0.1 | DisGeNET |
| SLC7A2 | 0.1 | DisGeNET |
| SPAG1 | 0.1 | DisGeNET |
| SRP9 | 0.1 | DisGeNET |
| BRCA2 | 0.1 | DisGeNET |
| STAT5B | 0.1 | DisGeNET |
| BRS3 | 0.1 | DisGeNET |
| TAC1 | 0.1 | DisGeNET |
| TALDO1 | 0.1 | DisGeNET |
| TAP2 | 0.1 | DisGeNET |
| TBCD | 0.1 | DisGeNET |
| TBXA2R | 0.1 | DisGeNET |
| TDGF1 | 0.1 | DisGeNET |
| TFRC | 0.1 | DisGeNET |
| TGIF1 | 0.1 | DisGeNET |
| TLR2 | 0.1 | DisGeNET |
| TLR4 | 0.1 | DisGeNET |
| ACTG1 | 0.1 | DisGeNET |
| ACTG2 | 0.1 | DisGeNET |
| D2HGDH | 0.1 | DisGeNET |
| HIRA | 0.1 | DisGeNET |
| UFD1 | 0.1 | DisGeNET |
| VDR | 0.1 | DisGeNET |
| TRPV1 | 0.1 | DisGeNET |
| MYRF | 0.1 | DisGeNET |
| TMEM258 | 0.1 | DisGeNET |
| ZIC2 | 0.1 | DisGeNET |
| ZBTB16 | 0.1 | DisGeNET |
| EVI5 | 0.1 | DisGeNET |
| ALMS1 | 0.1 | DisGeNET |
| CCDC28B | 0.1 | DisGeNET |
| DDX39B | 0.1 | DisGeNET |
| MMEL1 | 0.1 | DisGeNET |
| EFL1 | 0.1 | DisGeNET |
| AIMP2 | 0.1 | DisGeNET |
| TBL1XR1 | 0.1 | DisGeNET |
| ARHGEF5 | 0.1 | DisGeNET |
| PRR5L | 0.1 | DisGeNET |
| ZNF614 | 0.1 | DisGeNET |
| CCDC33 | 0.1 | DisGeNET |
| SPSB1 | 0.1 | DisGeNET |
| ADAM33 | 0.1 | DisGeNET |
| ASXL3 | 0.1 | DisGeNET |
| CAMK4 | 0.1 | DisGeNET |
| POLR2M | 0.1 | DisGeNET |
| TRIM8 | 0.1 | DisGeNET |
| OR5V1 | 0.1 | DisGeNET |
| OR12D3 | 0.1 | DisGeNET |
| GATD3A | 0.1 | DisGeNET |
| LZTR1 | 0.1 | DisGeNET |
| ARHGAP24 | 0.1 | DisGeNET |
| TTC25 | 0.1 | DisGeNET |
| DNAL1 | 0.1 | DisGeNET |
| PARP9 | 0.1 | DisGeNET |
| RSPH3 | 0.1 | DisGeNET |
| TATDN1 | 0.1 | DisGeNET |
| CARD11 | 0.1 | DisGeNET |
| ATRN | 0.1 | DisGeNET |
| COX4I2 | 0.1 | DisGeNET |
| OFD1 | 0.1 | DisGeNET |
| PHF5A | 0.1 | DisGeNET |
| FBXL20 | 0.1 | DisGeNET |
| DISP1 | 0.1 | DisGeNET |
| CFAP300 | 0.1 | DisGeNET |
| ITGA8 | 0.1 | DisGeNET |
| AP3B1 | 0.1 | DisGeNET |
| CCDC65 | 0.1 | DisGeNET |
| CDK13 | 0.1 | DisGeNET |
| SERPINB7 | 0.1 | DisGeNET |
| TIMELESS | 0.1 | DisGeNET |
| HERC2 | 0.1 | DisGeNET |
| FOXH1 | 0.1 | DisGeNET |
| RSPH1 | 0.1 | DisGeNET |
| PPP1R3F | 0.1 | DisGeNET |
| NEK9 | 0.1 | DisGeNET |
| CD247 | 0.1 | DisGeNET |
| SLC16A7 | 0.1 | DisGeNET |
| ARRDC1 | 0.1 | DisGeNET |
| DRC1 | 0.1 | DisGeNET |
| ADAMTSL1 | 0.1 | DisGeNET |
| CCDC114 | 0.1 | DisGeNET |
| ADIPOQ | 0.1 | DisGeNET |
| GRAP2 | 0.1 | DisGeNET |
| POM121L2 | 0.1 | DisGeNET |
| MAP4K4 | 0.1 | DisGeNET |
| FHL5 | 0.1 | DisGeNET |
| AKAP6 | 0.1 | DisGeNET |
| ADAMTS4 | 0.1 | DisGeNET |
| SEC24C | 0.1 | DisGeNET |
| SH3PXD2A | 0.1 | DisGeNET |
| LPIN2 | 0.1 | DisGeNET |
| GREB1 | 0.1 | DisGeNET |
| PRORP | 0.1 | DisGeNET |
| RIPOR2 | 0.1 | DisGeNET |
| TESPA1 | 0.1 | DisGeNET |
| ZBTB39 | 0.1 | DisGeNET |
| CXCR6 | 0.09 | DisGeNET |
| CHIT1 | 0.09 | DisGeNET |
| ADRA1A | 0.09 | DisGeNET |
| ADRA2B | 0.09 | DisGeNET |
| GPR42 | 0.09 | DisGeNET |
| IL15 | 0.09 | DisGeNET |
| MPO | 0.09 | DisGeNET |
| PLG | 0.09 | DisGeNET |
| ANO1 | 0.09 | DisGeNET |
| SSTR4 | 0.09 | DisGeNET |
| TPI1 | 0.09 | DisGeNET |
| LPAR2 | 0.09 | DisGeNET |
| NAT2 | 0.08 | DisGeNET |
| NOD1 | 0.08 | DisGeNET |
| LTB4R | 0.08 | DisGeNET |
| AKT1 | 0.08 | DisGeNET |
| GC | 0.08 | DisGeNET |
| HIF1A | 0.08 | DisGeNET |
| PCDH1 | 0.08 | DisGeNET |
| TLR7 | 0.08 | DisGeNET |
| PPARG | 0.08 | DisGeNET |
| SLC52A1 | 0.08 | DisGeNET |
| PTGS1 | 0.08 | DisGeNET |
| VCAM1 | 0.08 | DisGeNET |
| HAVCR2 | 0.08 | DisGeNET |
| NR1I2 | 0.08 | DisGeNET |
| CD28 | 0.08 | DisGeNET |
| WDHD1 | 0.07 | DisGeNET |
| CHRM3 | 0.07 | DisGeNET |
| OXER1 | 0.07 | DisGeNET |
| FCGR3A | 0.07 | DisGeNET |
| CXCR3 | 0.07 | DisGeNET |
| IL16 | 0.07 | DisGeNET |
| MIR21 | 0.07 | DisGeNET |
| MMP12 | 0.07 | DisGeNET |
| ISYNA1 | 0.07 | DisGeNET |
| IL23A | 0.07 | DisGeNET |
| ACTB | 0.07 | DisGeNET |
| CCL20 | 0.07 | DisGeNET |
| STAT1 | 0.07 | DisGeNET |
| CAMP | 0.07 | DisGeNET |
| SOCS3 | 0.07 | DisGeNET |
| CD38 | 0.07 | DisGeNET |
| CCR1 | 0.06 | DisGeNET |
| CCN2 | 0.06 | DisGeNET |
| DPP4 | 0.06 | DisGeNET |
| AGT | 0.06 | DisGeNET |
| EPHB2 | 0.06 | DisGeNET |
| ESR1 | 0.06 | DisGeNET |
| FCER1A | 0.06 | DisGeNET |
| AKR1B1 | 0.06 | DisGeNET |
| FN1 | 0.06 | DisGeNET |
| FOS | 0.06 | DisGeNET |
| FOSB | 0.06 | DisGeNET |
| IL27 | 0.06 | DisGeNET |
| IL19 | 0.06 | DisGeNET |
| HSPA4 | 0.06 | DisGeNET |
| IL9R | 0.06 | DisGeNET |
| ITGB3 | 0.06 | DisGeNET |
| JUN | 0.06 | DisGeNET |
| JUNB | 0.06 | DisGeNET |
| JUND | 0.06 | DisGeNET |
| MIR221 | 0.06 | DisGeNET |
| MRC1 | 0.06 | DisGeNET |
| MUC1 | 0.06 | DisGeNET |
| PHF11 | 0.06 | DisGeNET |
| IL17D | 0.06 | DisGeNET |
| LTB4R2 | 0.06 | DisGeNET |
| RNASE2 | 0.06 | DisGeNET |
| CXCL12 | 0.06 | DisGeNET |
| SMPD1 | 0.06 | DisGeNET |
| TIMP1 | 0.06 | DisGeNET |
| CCR2 | 0.06 | DisGeNET |
| EPX | 0.06 | DisGeNET |
| PLA2G10 | 0.06 | DisGeNET |
| CD34 | 0.06 | DisGeNET |
| CCL27 | 0.05 | DisGeNET |
| CYP2R1 | 0.05 | DisGeNET |
| AGXT | 0.05 | DisGeNET |
| FCGR3B | 0.05 | DisGeNET |
| FKBP5 | 0.05 | DisGeNET |
| MMRN1 | 0.05 | DisGeNET |
| ALOX15 | 0.05 | DisGeNET |
| HDAC1 | 0.05 | DisGeNET |
| NRG1 | 0.05 | DisGeNET |
| CXCR2 | 0.05 | DisGeNET |
| IL13RA1 | 0.05 | DisGeNET |
| IRF1 | 0.05 | DisGeNET |
| IL31 | 0.05 | DisGeNET |
| LGALS3 | 0.05 | DisGeNET |
| SMAD2 | 0.05 | DisGeNET |
| KITLG | 0.05 | DisGeNET |
| IL17RB | 0.05 | DisGeNET |
| MAPK8 | 0.05 | DisGeNET |
| IL21 | 0.05 | DisGeNET |
| S100A8 | 0.05 | DisGeNET |
| CCL13 | 0.05 | DisGeNET |
| SOD2 | 0.05 | DisGeNET |
| SPP1 | 0.05 | DisGeNET |
| TGFB2 | 0.05 | DisGeNET |
| C3 | 0.05 | DisGeNET |
| CD1D | 0.05 | DisGeNET |
| IL32 | 0.05 | DisGeNET |
| ADAM8 | 0.04 | DisGeNET |
| TNFSF13B | 0.04 | DisGeNET |
| IRAK3 | 0.04 | DisGeNET |
| ADCY9 | 0.04 | DisGeNET |
| MRGPRX3 | 0.04 | DisGeNET |
| MRGPRX4 | 0.04 | DisGeNET |
| CMA1 | 0.04 | DisGeNET |
| COL4A5 | 0.04 | DisGeNET |
| GPR151 | 0.04 | DisGeNET |
| CPA3 | 0.04 | DisGeNET |
| CREB1 | 0.04 | DisGeNET |
| CRHR1 | 0.04 | DisGeNET |
| CSF3 | 0.04 | DisGeNET |
| CX3CR1 | 0.04 | DisGeNET |
| CYP2C19 | 0.04 | DisGeNET |
| CYP24A1 | 0.04 | DisGeNET |
| CYP27B1 | 0.04 | DisGeNET |
| DEFB1 | 0.04 | DisGeNET |
| DUSP1 | 0.04 | DisGeNET |
| EGR1 | 0.04 | DisGeNET |
| ELANE | 0.04 | DisGeNET |
| EPHX1 | 0.04 | DisGeNET |
| F3 | 0.04 | DisGeNET |
| GPRC6A | 0.04 | DisGeNET |
| FGF2 | 0.04 | DisGeNET |
| SIRT1 | 0.04 | DisGeNET |
| LPAR3 | 0.04 | DisGeNET |
| IL17RA | 0.04 | DisGeNET |
| MRGPRX1 | 0.04 | DisGeNET |
| GCG | 0.04 | DisGeNET |
| IFNL1 | 0.04 | DisGeNET |
| H19 | 0.04 | DisGeNET |
| LAMA1 | 0.04 | DisGeNET |
| GSTA1 | 0.04 | DisGeNET |
| HLA-DRA | 0.04 | DisGeNET |
| HRH1 | 0.04 | DisGeNET |
| IL7 | 0.04 | DisGeNET |
| IL11 | 0.04 | DisGeNET |
| IL13RA2 | 0.04 | DisGeNET |
| ITGB4 | 0.04 | DisGeNET |
| ITK | 0.04 | DisGeNET |
| LEPR | 0.04 | DisGeNET |
| LGALS9 | 0.04 | DisGeNET |
| MIR126 | 0.04 | DisGeNET |
| MIR145 | 0.04 | DisGeNET |
| MAS1 | 0.04 | DisGeNET |
| VN1R17P | 0.04 | DisGeNET |
| GPR166P | 0.04 | DisGeNET |
| MTHFR | 0.04 | DisGeNET |
| MYD88 | 0.04 | DisGeNET |
| ATP5F1A | 0.04 | DisGeNET |
| PLA2G2A | 0.04 | DisGeNET |
| SERPINF2 | 0.04 | DisGeNET |
| POMC | 0.04 | DisGeNET |
| SAGE1 | 0.04 | DisGeNET |
| MAPK3 | 0.04 | DisGeNET |
| TRPV4 | 0.04 | DisGeNET |
| LGR6 | 0.04 | DisGeNET |
| NPS | 0.04 | DisGeNET |
| SERPINB3 | 0.04 | DisGeNET |
| SERPINB4 | 0.04 | DisGeNET |
| SCD | 0.04 | DisGeNET |
| CCL18 | 0.04 | DisGeNET |
| CX3CL1 | 0.04 | DisGeNET |
| ECSCR | 0.04 | DisGeNET |
| ST2 | 0.04 | DisGeNET |
| TNFAIP3 | 0.04 | DisGeNET |
| TPT1 | 0.04 | DisGeNET |
| MUC5B | 0.04 | DisGeNET |
| POTEF | 0.04 | DisGeNET |
| TNFRSF4 | 0.04 | DisGeNET |
| TRPM8 | 0.04 | DisGeNET |
| TNFAIP8L2 | 0.04 | DisGeNET |
| CD276 | 0.04 | DisGeNET |
| SLC2A10 | 0.04 | DisGeNET |
| FZD4 | 0.04 | DisGeNET |
| CASP1 | 0.04 | DisGeNET |
| MBD2 | 0.04 | DisGeNET |
| TIMD4 | 0.04 | DisGeNET |
| CD19 | 0.04 | DisGeNET |
| ENTPD1 | 0.04 | DisGeNET |
| FAM13A | 0.03 | DisGeNET |
| ADAM10 | 0.03 | DisGeNET |
| TUBB4B | 0.03 | DisGeNET |
| RACK1 | 0.03 | DisGeNET |
| FSTL1 | 0.03 | DisGeNET |
| CHRM1 | 0.03 | DisGeNET |
| CLC | 0.03 | DisGeNET |
| GSTO2 | 0.03 | DisGeNET |
| CMD1B | 0.03 | DisGeNET |
| CCR7 | 0.03 | DisGeNET |
| ADH5 | 0.03 | DisGeNET |
| COX8A | 0.03 | DisGeNET |
| CPOX | 0.03 | DisGeNET |
| CYBA | 0.03 | DisGeNET |
| DECR1 | 0.03 | DisGeNET |
| EGF | 0.03 | DisGeNET |
| FCGR2B | 0.03 | DisGeNET |
| FDXR | 0.03 | DisGeNET |
| FKBP4 | 0.03 | DisGeNET |
| ATF6 | 0.03 | DisGeNET |
| FLNC | 0.03 | DisGeNET |
| TNFRSF13B | 0.03 | DisGeNET |
| GAPDH | 0.03 | DisGeNET |
| GGT1 | 0.03 | DisGeNET |
| SIGLEC8 | 0.03 | DisGeNET |
| ABO | 0.03 | DisGeNET |
| ANGPT1 | 0.03 | DisGeNET |
| GSTM2 | 0.03 | DisGeNET |
| HLX | 0.03 | DisGeNET |
| HP | 0.03 | DisGeNET |
| APOA1 | 0.03 | DisGeNET |
| IGF1 | 0.03 | DisGeNET |
| IGF1R | 0.03 | DisGeNET |
| LINC01193 | 0.03 | DisGeNET |
| IGHG3 | 0.03 | DisGeNET |
| APRT | 0.03 | DisGeNET |
| IL12A | 0.03 | DisGeNET |
| IRAK1 | 0.03 | DisGeNET |
| IRF5 | 0.03 | DisGeNET |
| ITGAM | 0.03 | DisGeNET |
| JAK3 | 0.03 | DisGeNET |
| LAG3 | 0.03 | DisGeNET |
| LIF | 0.03 | DisGeNET |
| LTB | 0.03 | DisGeNET |
| MIR152 | 0.03 | DisGeNET |
| MIR19A | 0.03 | DisGeNET |
| ARSA | 0.03 | DisGeNET |
| MFAP1 | 0.03 | DisGeNET |
| MMP2 | 0.03 | DisGeNET |
| MUC2 | 0.03 | DisGeNET |
| NEUROD1 | 0.03 | DisGeNET |
| NFKB1 | 0.03 | DisGeNET |
| NM | 0.03 | DisGeNET |
| P2RX7 | 0.03 | DisGeNET |
| SERPINB2 | 0.03 | DisGeNET |
| ACP1 | 0.03 | DisGeNET |
| PLAUR | 0.03 | DisGeNET |
| TREM1 | 0.03 | DisGeNET |
| PON1 | 0.03 | DisGeNET |
| PTPA | 0.03 | DisGeNET |
| PRKCA | 0.03 | DisGeNET |
| AXL | 0.03 | DisGeNET |
| PTAFR | 0.03 | DisGeNET |
| PTGER4 | 0.03 | DisGeNET |
| KIDINS220 | 0.03 | DisGeNET |
| PTPN11 | 0.03 | DisGeNET |
| RAC1 | 0.03 | DisGeNET |
| MOK | 0.03 | DisGeNET |
| CCND1 | 0.03 | DisGeNET |
| RGS2 | 0.03 | DisGeNET |
| S100A9 | 0.03 | DisGeNET |
| CCL19 | 0.03 | DisGeNET |
| CCL21 | 0.03 | DisGeNET |
| SELE | 0.03 | DisGeNET |
| CRLF2 | 0.03 | DisGeNET |
| IFIH1 | 0.03 | DisGeNET |
| SMARCA4 | 0.03 | DisGeNET |
| SOAT1 | 0.03 | DisGeNET |
| TACR2 | 0.03 | DisGeNET |
| THBS1 | 0.03 | DisGeNET |
| TSPO | 0.03 | DisGeNET |
| TRAF6 | 0.03 | DisGeNET |
| YY1 | 0.03 | DisGeNET |
| CXCR4 | 0.03 | DisGeNET |
| CALCA | 0.03 | DisGeNET |
| ELP1 | 0.03 | DisGeNET |
| TP63 | 0.03 | DisGeNET |
| BECN1 | 0.03 | DisGeNET |
| MS4A3 | 0.03 | DisGeNET |
| COX5A | 0.03 | DisGeNET |
| CD48 | 0.03 | DisGeNET |
| CD68 | 0.03 | DisGeNET |
| CD69 | 0.03 | DisGeNET |
| HDAC9 | 0.03 | DisGeNET |
| CDC42 | 0.03 | DisGeNET |
| CDH1 | 0.03 | DisGeNET |
| SIGLEC14 | 0.02 | DisGeNET |
| MIR744 | 0.02 | DisGeNET |
| HDAC6 | 0.02 | DisGeNET |
| LINC01672 | 0.02 | DisGeNET |
| ABCB6 | 0.02 | DisGeNET |
| PGR-AS1 | 0.02 | DisGeNET |
| LRPPRC | 0.02 | DisGeNET |
| EBI3 | 0.02 | DisGeNET |
| RAMP1 | 0.02 | DisGeNET |
| CDKN1B | 0.02 | DisGeNET |
| LOC102723996 | 0.02 | DisGeNET |
| SEMA3A | 0.02 | DisGeNET |
| KAT5 | 0.02 | DisGeNET |
| AGR2 | 0.02 | DisGeNET |
| STIP1 | 0.02 | DisGeNET |
| CKAP4 | 0.02 | DisGeNET |
| PRRT2 | 0.02 | DisGeNET |
| CHRM2 | 0.02 | DisGeNET |
| CHRNA3 | 0.02 | DisGeNET |
| SLC26A9 | 0.02 | DisGeNET |
| FCRL3 | 0.02 | DisGeNET |
| RASGRP4 | 0.02 | DisGeNET |
| MRGPRX2 | 0.02 | DisGeNET |
| CLU | 0.02 | DisGeNET |
| FRMD6 | 0.02 | DisGeNET |
| COL4A3 | 0.02 | DisGeNET |
| DCBLD2 | 0.02 | DisGeNET |
| GNPDA2 | 0.02 | DisGeNET |
| ADM | 0.02 | DisGeNET |
| PPARGC1B | 0.02 | DisGeNET |
| ADORA2A | 0.02 | DisGeNET |
| COL26A1 | 0.02 | DisGeNET |
| CR1 | 0.02 | DisGeNET |
| CREBBP | 0.02 | DisGeNET |
| CRHR2 | 0.02 | DisGeNET |
| CTSS | 0.02 | DisGeNET |
| CYBB | 0.02 | DisGeNET |
| CYLD | 0.02 | DisGeNET |
| CYP1A2 | 0.02 | DisGeNET |
| CYP2B6 | 0.02 | DisGeNET |
| DNASE1L3 | 0.02 | DisGeNET |
| SLC26A3 | 0.02 | DisGeNET |
| ABCA1 | 0.02 | DisGeNET |
| S1PR1 | 0.02 | DisGeNET |
| ELF5 | 0.02 | DisGeNET |
| EREG | 0.02 | DisGeNET |
| F2R | 0.02 | DisGeNET |
| ACSL3 | 0.02 | DisGeNET |
| PTK2B | 0.02 | DisGeNET |
| FBLN1 | 0.02 | DisGeNET |
| FCGRT | 0.02 | DisGeNET |
| FGF1 | 0.02 | DisGeNET |
| NLRP1 | 0.02 | DisGeNET |
| FOXM1 | 0.02 | DisGeNET |
| FOXO1 | 0.02 | DisGeNET |
| FOXO3 | 0.02 | DisGeNET |
| CEP68 | 0.02 | DisGeNET |
| TPSD1 | 0.02 | DisGeNET |
| BRD4 | 0.02 | DisGeNET |
| FOSL2 | 0.02 | DisGeNET |
| FPR2 | 0.02 | DisGeNET |
| OPN3 | 0.02 | DisGeNET |
| ALPL | 0.02 | DisGeNET |
| ACKR1 | 0.02 | DisGeNET |
| TAC4 | 0.02 | DisGeNET |
| ARIH1 | 0.02 | DisGeNET |
| ALRH | 0.02 | DisGeNET |
| LRRC32 | 0.02 | DisGeNET |
| GATA1 | 0.02 | DisGeNET |
| FBXO8 | 0.02 | DisGeNET |
| EHF | 0.02 | DisGeNET |
| GJA1 | 0.02 | DisGeNET |
| ACAD8 | 0.02 | DisGeNET |
| LAT | 0.02 | DisGeNET |
| AGO2 | 0.02 | DisGeNET |
| IFNL2 | 0.02 | DisGeNET |
| RGMB | 0.02 | DisGeNET |
| FLVCR1 | 0.02 | DisGeNET |
| ANPEP | 0.02 | DisGeNET |
| CD274 | 0.02 | DisGeNET |
| CXCL1 | 0.02 | DisGeNET |
| ANXA1 | 0.02 | DisGeNET |
| ANXA2 | 0.02 | DisGeNET |
| KCNIP3 | 0.02 | DisGeNET |
| HLA-A | 0.02 | DisGeNET |
| AOAH | 0.02 | DisGeNET |
| HMGCR | 0.02 | DisGeNET |
| FOXA2 | 0.02 | DisGeNET |
| HSPA1B | 0.02 | DisGeNET |
| HSP90AA1 | 0.02 | DisGeNET |
| IGES | 0.02 | DisGeNET |
| APOE | 0.02 | DisGeNET |
| IGFBP3 | 0.02 | DisGeNET |
| IGHG1 | 0.02 | DisGeNET |
| IGHG2 | 0.02 | DisGeNET |
| APP | 0.02 | DisGeNET |
| IL6ST | 0.02 | DisGeNET |
| IL12RB1 | 0.02 | DisGeNET |
| TNFRSF9 | 0.02 | DisGeNET |
| IDO1 | 0.02 | DisGeNET |
| INPP4A | 0.02 | DisGeNET |
| INSRR | 0.02 | DisGeNET |
| IRF7 | 0.02 | DisGeNET |
| JAK1 | 0.02 | DisGeNET |
| KIT | 0.02 | DisGeNET |
| KLK1 | 0.02 | DisGeNET |
| LAMA5 | 0.02 | DisGeNET |
| ARNTL | 0.02 | DisGeNET |
| MIR142 | 0.02 | DisGeNET |
| MIR192 | 0.02 | DisGeNET |
| MIR200A | 0.02 | DisGeNET |
| MIR216A | 0.02 | DisGeNET |
| MIR22 | 0.02 | DisGeNET |
| MARCKS | 0.02 | DisGeNET |
| MCAM | 0.02 | DisGeNET |
| MEFV | 0.02 | DisGeNET |
| MET | 0.02 | DisGeNET |
| CXCL9 | 0.02 | DisGeNET |
| ACHE | 0.02 | DisGeNET |
| CD200 | 0.02 | DisGeNET |
| ABCC1 | 0.02 | DisGeNET |
| COX1 | 0.02 | DisGeNET |
| MUSK | 0.02 | DisGeNET |
| MYC | 0.02 | DisGeNET |
| NFATC2 | 0.02 | DisGeNET |
| NHS | 0.02 | DisGeNET |
| NOTCH1 | 0.02 | DisGeNET |
| NTF4 | 0.02 | DisGeNET |
| TNFRSF11B | 0.02 | DisGeNET |
| OSM | 0.02 | DisGeNET |
| ALDH7A1 | 0.02 | DisGeNET |
| PEBP1 | 0.02 | DisGeNET |
| NOX4 | 0.02 | DisGeNET |
| IL21R | 0.02 | DisGeNET |
| MBL3P | 0.02 | DisGeNET |
| TAS2R14 | 0.02 | DisGeNET |
| PCBD1 | 0.02 | DisGeNET |
| PCNA | 0.02 | DisGeNET |
| IRAK4 | 0.02 | DisGeNET |
| CKLF | 0.02 | DisGeNET |
| CPA4 | 0.02 | DisGeNET |
| BPIFA1 | 0.02 | DisGeNET |
| TLR8 | 0.02 | DisGeNET |
| PDCD1 | 0.02 | DisGeNET |
| PECAM1 | 0.02 | DisGeNET |
| SERPINE2 | 0.02 | DisGeNET |
| PLD1 | 0.02 | DisGeNET |
| PMCH | 0.02 | DisGeNET |
| PNOC | 0.02 | DisGeNET |
| TOLLIP | 0.02 | DisGeNET |
| PARP14 | 0.02 | DisGeNET |
| PPARA | 0.02 | DisGeNET |
| DYM | 0.02 | DisGeNET |
| GIMAP5 | 0.02 | DisGeNET |
| PRG2 | 0.02 | DisGeNET |
| KLK15 | 0.02 | DisGeNET |
| PRKCB | 0.02 | DisGeNET |
| EAF2 | 0.02 | DisGeNET |
| USE1 | 0.02 | DisGeNET |
| GABRQ | 0.02 | DisGeNET |
| PSMB6 | 0.02 | DisGeNET |
| AICDA | 0.02 | DisGeNET |
| MIR499A | 0.02 | DisGeNET |
| PTPN6 | 0.02 | DisGeNET |
| PTPRD | 0.02 | DisGeNET |
| PTPRE | 0.02 | DisGeNET |
| PVT1 | 0.02 | DisGeNET |
| RAPSN | 0.02 | DisGeNET |
| ACE2 | 0.02 | DisGeNET |
| SIGIRR | 0.02 | DisGeNET |
| RBP4 | 0.02 | DisGeNET |
| RGS4 | 0.02 | DisGeNET |
| BCL6 | 0.02 | DisGeNET |
| ROS1 | 0.02 | DisGeNET |
| RPE65 | 0.02 | DisGeNET |
| FIQTL1 | 0.02 | DisGeNET |
| RRBP1 | 0.02 | DisGeNET |
| CLEC11A | 0.02 | DisGeNET |
| CCL1 | 0.02 | DisGeNET |
| CCL3 | 0.02 | DisGeNET |
| CCL3L1 | 0.02 | DisGeNET |
| CXCL11 | 0.02 | DisGeNET |
| XCL1 | 0.02 | DisGeNET |
| SELL | 0.02 | DisGeNET |
| SELP | 0.02 | DisGeNET |
| SEMA4A | 0.02 | DisGeNET |
| ARHGEF28 | 0.02 | DisGeNET |
| CLEC7A | 0.02 | DisGeNET |
| BCL11B | 0.02 | DisGeNET |
| UBE2Z | 0.02 | DisGeNET |
| SLC6A4 | 0.02 | DisGeNET |
| GGTLC5P | 0.02 | DisGeNET |
| SLC8A1 | 0.02 | DisGeNET |
| SMN1 | 0.02 | DisGeNET |
| SMN2 | 0.02 | DisGeNET |
| SNRNP70 | 0.02 | DisGeNET |
| TAC3 | 0.02 | DisGeNET |
| TAP1 | 0.02 | DisGeNET |
| TCF7 | 0.02 | DisGeNET |
| MIR625 | 0.02 | DisGeNET |
| TRBV20OR9-2 | 0.02 | DisGeNET |
| TEK | 0.02 | DisGeNET |
| TFF2 | 0.02 | DisGeNET |
| TGFBR2 | 0.02 | DisGeNET |
| TGM2 | 0.02 | DisGeNET |
| THBD | 0.02 | DisGeNET |
| TLE4 | 0.02 | DisGeNET |
| SERPING1 | 0.02 | DisGeNET |
| TM7SF2 | 0.02 | DisGeNET |
| TP53 | 0.02 | DisGeNET |
| TPSAB1 | 0.02 | DisGeNET |
| TRPC3 | 0.02 | DisGeNET |
| GGTLC3 | 0.02 | DisGeNET |
| GGT2 | 0.02 | DisGeNET |
| TWIST1 | 0.02 | DisGeNET |
| SFTPA2 | 0.02 | DisGeNET |
| GGTLC4P | 0.02 | DisGeNET |
| TYRO3 | 0.02 | DisGeNET |
| UVRAG | 0.02 | DisGeNET |
| EZR | 0.02 | DisGeNET |
| XBP1 | 0.02 | DisGeNET |
| FTO | 0.02 | DisGeNET |
| EOS | 0.02 | DisGeNET |
| FSD1 | 0.02 | DisGeNET |
| GSDMD | 0.02 | DisGeNET |
| NR4A3 | 0.02 | DisGeNET |
| FLAD1 | 0.02 | DisGeNET |
| TET1 | 0.02 | DisGeNET |
| FOSL1 | 0.02 | DisGeNET |
| ADAMTS12 | 0.02 | DisGeNET |
| CRISPLD2 | 0.02 | DisGeNET |
| FSD1L | 0.02 | DisGeNET |
| TAGLN2 | 0.02 | DisGeNET |
| NR0B2 | 0.02 | DisGeNET |
| IL1F10 | 0.02 | DisGeNET |
| RETNLB | 0.02 | DisGeNET |
| ORAI1 | 0.02 | DisGeNET |
| USO1 | 0.02 | DisGeNET |
| SOCS1 | 0.02 | DisGeNET |
| CBL | 0.02 | DisGeNET |
| TNFSF10 | 0.02 | DisGeNET |
| TNFRSF14 | 0.02 | DisGeNET |
| RIPK2 | 0.02 | DisGeNET |
| VNN1 | 0.02 | DisGeNET |
| FCGR2C | 0.02 | DisGeNET |
| MSC | 0.02 | DisGeNET |
| DNER | 0.02 | DisGeNET |
| CD163 | 0.02 | DisGeNET |
| CD27 | 0.02 | DisGeNET |
| TNFRSF8 | 0.02 | DisGeNET |
| CLOCK | 0.02 | DisGeNET |
| CD44 | 0.02 | DisGeNET |
| UBE3C | 0.02 | DisGeNET |
| SEMA3E | 0.02 | DisGeNET |
| HDAC4 | 0.02 | DisGeNET |
| USP38 | - | OMIM |
| GLCCI1 | - | OMIM |
| HSD11B2 | - | OMIM |
| DENND1B | - | OMIM |
| MIR126 | - | OMIM |
| ORMDL3 | - | OMIM |
| SPRED1 | - | OMIM |
| POSTN | - | OMIM |
| NPSRAS1 | - | OMIM |
| NPSR1 | - | OMIM |
| DPP10 | - | OMIM |
| SETDB2 | - | OMIM |
| PHF11 | - | OMIM |
| TNIP1 | - | OMIM |
| BPIFA1 | - | OMIM |
| RASGRP4 | - | OMIM |
| ADAM33 | - | OMIM |
| SCGB3A2 | - | OMIM |
| HAVCR1 | - | OMIM |
| CHIA | - | OMIM |
| NOD1 | - | OMIM |
| SART1 | - | OMIM |
| CYSLTR2 | - | OMIM |
| GAD1 | - | OMIM |
| C3AR1 | - | OMIM |
| HNMT | - | OMIM |
| SPINK5 | - | OMIM |
| TBX21 | - | OMIM |
| PTGDR2 | - | OMIM |
| PTGDR | - | OMIM |
| TNFSF14 | - | OMIM |
| IRAK3 | - | OMIM |
| SOCS3 | - | OMIM |
| CLCA1 | - | OMIM |
| CCL18 | - | OMIM |
| ALOX5AP | - | OMIM |
| TNFSF10 | - | OMIM |
| ADCY9 | - | OMIM |
| CPN1 | - | OMIM |
| TLR4 | - | OMIM |
| FKBP5 | - | OMIM |
| CCL24 | - | OMIM |
| CFTR | - | OMIM |
| DAP3 | - | OMIM |
| IRF4 | - | OMIM |
| VDR | - | OMIM |
| PTEN | - | OMIM |
| PLA2G7 | - | OMIM |
| CHI3L1 | - | OMIM |
| STAT6 | - | OMIM |
| CCR5 | - | OMIM |
| CCL11 | - | OMIM |
| MMP12 | - | OMIM |
| TPT1 | - | OMIM |
| SERPINB4 | - | OMIM |
| SERPINB3 | - | OMIM |
| TYRO3 | - | OMIM |
| TNFRSF4 | - | OMIM |
| GABRB2 | - | OMIM |
| RUNX3 | - | OMIM |
| CYSLTR1 | - | OMIM |
| IL13RA2 | - | OMIM |
| IL13RA1 | - | OMIM |
| IL9R | - | OMIM |
| LTC4S | - | OMIM |
| SCGB1A1 | - | OMIM |
| TNF | - | OMIM |
| TBXA2R | - | OMIM |
| TRBC1 | - | OMIM |
| MARCKS | - | OMIM |
| PRKCA | - | OMIM |
| PTGER2 | - | OMIM |
| SELP | - | OMIM |
| SERPINB2 | - | OMIM |
| NFKBIA | - | OMIM |
| IL12B | - | OMIM |
| MUC7 | - | OMIM |
| LGALS3 | - | OMIM |
| LTA | - | OMIM |
| ALOX5 | - | OMIM |
| IL6R | - | OMIM |
| IL5 | - | OMIM |
| IL4R | - | OMIM |
| IL4 | - | OMIM |
| IL13 | - | OMIM |
| FCER1A | - | OMIM |
| MS4A2 | - | OMIM |
| IL9 | - | OMIM |
| HLA-G | - | OMIM |
| HLA-DRB1 | - | OMIM |
| GAD2 | - | OMIM |
| GABRA2 | - | OMIM |
| FLG | - | OMIM |
| GATA3 | - | OMIM |
| IL10 | - | OMIM |
| C5 | - | OMIM |
| C3 | - | OMIM |
| MMP9 | - | OMIM |
| MMP2 | - | OMIM |
| CMA1 | - | OMIM |
| C5AR1 | - | OMIM |
| ADORA3 | - | DRUGBANK |
| SLCO1B3 | - | DRUGBANK |
| SLC6A2 | - | DRUGBANK |
| SLC6A4 | - | DRUGBANK |
| SLC6A3 | - | DRUGBANK |
| CES1 | - | DRUGBANK |
| S100P | - | DRUGBANK |
| HRH1 | - | DRUGBANK |
| NR1I2 | - | DRUGBANK |
| ABCB11 | - | DRUGBANK |
| CYP17A1 | - | DRUGBANK |
| NOS2 | - | DRUGBANK |
| NR0B1 | - | DRUGBANK |
| CYP4A11 | - | DRUGBANK |
| MAOA | - | DRUGBANK |
| COMT | - | DRUGBANK |
| MAOB | - | DRUGBANK |
| NR3C2 | - | DRUGBANK |
| PLA2G4A | - | DRUGBANK |
| SLCO1B1 | - | DRUGBANK |
| UGT2B7 | - | DRUGBANK |
| UGT2B15 | - | DRUGBANK |
| UGT1A8 | - | DRUGBANK |
| GRIN2D | - | DRUGBANK |
| GRIN3B | - | DRUGBANK |
| GRIN1 | - | DRUGBANK |
| GRIN2A | - | DRUGBANK |
| GRIN2B | - | DRUGBANK |
| GRIN2C | - | DRUGBANK |
| GRIN3A | - | DRUGBANK |
| SHBG | - | DRUGBANK |
| CYP11B1 | - | DRUGBANK |
| CYP11B2 | - | DRUGBANK |
| SRD5A2 | - | DRUGBANK |
| AKR1D1 | - | DRUGBANK |
| SLC22A8 | - | DRUGBANK |
| UGT1A1 | - | DRUGBANK |
| GLB1 | - | DRUGBANK |
| LGALS3 | - | DRUGBANK |
| GLT6D1 | - | DRUGBANK |
| GLTP | - | DRUGBANK |
| ANXA1 | - | DRUGBANK |
| AKR1C4 | - | DRUGBANK |
| AKR1C3 | - | DRUGBANK |
| AKR1C2 | - | DRUGBANK |
| HSD11B2 | - | DRUGBANK |
| AKR1C1 | - | DRUGBANK |
| PGR | - | DRUGBANK |
| SLCO2B1 | - | DRUGBANK |
| HSP90AA1 | - | DRUGBANK |
| FPR1 | - | DRUGBANK |
| PTGDR | - | DRUGBANK |
| CYSLTR2 | - | DRUGBANK |
| TNF | - | DRUGBANK |
| IL5 | - | DRUGBANK |
| RNASE3 | - | DRUGBANK |
| NFKB1 | - | DRUGBANK |
| MUC2 | - | DRUGBANK |
| ABCC2 | - | DRUGBANK |
| ABCB1 | - | DRUGBANK |
| CYP2A6 | - | DRUGBANK |
| CYP2B6 | - | DRUGBANK |
| HSD11B1 | - | DRUGBANK |
| SLCO1A2 | - | DRUGBANK |
| CYP3A43 | - | DRUGBANK |
| ADRA2A | - | DRUGBANK |
| ADRA2B | - | DRUGBANK |
| ADRA2C | - | DRUGBANK |
| ADRA1D | - | DRUGBANK |
| ADRA1A | - | DRUGBANK |
| ADRA1B | - | DRUGBANK |
| SLC22A2 | - | DRUGBANK |
| SLC22A1 | - | DRUGBANK |
| SLC22A3 | - | DRUGBANK |
| ORM1 | - | DRUGBANK |
| ORM2 | - | DRUGBANK |
| SLC25A1 | - | DRUGBANK |
| SLC13A2 | - | DRUGBANK |
| SLC13A5 | - | DRUGBANK |
| SLC25A21 | - | DRUGBANK |
| ADRB1 | - | DRUGBANK |
| ADRB3 | - | DRUGBANK |
| PDE5A | - | DRUGBANK |
| ADA | - | DRUGBANK |
| CYP1A1 | - | DRUGBANK |
| PARP1 | - | DRUGBANK |
| PDE4A | - | DRUGBANK |
| ADORA2A | - | DRUGBANK |
| ADORA2B | - | DRUGBANK |
| ADORA1 | - | DRUGBANK |
| PDE4B | - | DRUGBANK |
| PDE3A | - | DRUGBANK |
| NOMO1 | - | DRUGBANK |
| CYP1B1 | - | DRUGBANK |
| RIC3 | - | DRUGBANK |
| HM13 | - | DRUGBANK |
| HDAC2 | - | DRUGBANK |
| CPNE1 | - | DRUGBANK |
| TTLL3 | - | DRUGBANK |
| SLC22A7 | - | DRUGBANK |
| SLC22A5 | - | DRUGBANK |
| CHRM2 | - | DRUGBANK |
| CHRM4 | - | DRUGBANK |
| CHRM5 | - | DRUGBANK |
| CYP2D6 | - | DRUGBANK |
| CHRM1 | - | DRUGBANK |
| CHRM3 | - | DRUGBANK |
| SLC22A4 | - | DRUGBANK |
| NR3C1 | - | DRUGBANK |
| BCHE | - | DRUGBANK |
| SERPINA6 | - | DRUGBANK |
| CYP3A5 | - | DRUGBANK |
| CYP3A7 | - | DRUGBANK |
| PTGS2 | - | DRUGBANK |
| ADRB2 | - | DRUGBANK |
| CYP2E1 | - | DRUGBANK |
| CYP2C8 | - | DRUGBANK |
| CYP2C19 | - | DRUGBANK |
| ABCG2 | - | DRUGBANK |
| CYSLTR1 | - | DRUGBANK |
| UGT1A9 | - | DRUGBANK |
| ALB | - | DRUGBANK |
| CYP1A2 | - | DRUGBANK |
| CYP3A4 | - | DRUGBANK |
| ALOX5 | - | DRUGBANK |
| CYP2C9 | - | DRUGBANK |
| PTGS1 | - | DRUGBANK |

Supplementary Table 4: One thousand six hundred and sixteen targets related to asthma after removing duplicate items.

| Gene Symbol |
| --- |
| TBX21 |
| IL5 |
| IL13 |
| CCL11 |
| TGFB1 |
| ICAM1 |
| IL4 |
| IL6 |
| MMP9 |
| NOS2 |
| CCL2 |
| CCL5 |
| SCGB3A2 |
| ADRB2 |
| ALOX5 |
| HLA-DQB1 |
| HLA-DRB1 |
| HLA-G |
| GSDMB |
| TNF |
| TSLP |
| IL33 |
| IL1RL1 |
| ORMDL3 |
| HLA-DQA1 |
| IL6R |
| RAD50 |
| PDE4D |
| IKZF3 |
| HNMT |
| CDHR3 |
| KIF3A |
| PYHIN1 |
| MUC7 |
| PLA2G7 |
| DNAH5 |
| TNIP1 |
| WDR36 |
| PTGDR2 |
| CCR3 |
| PARP1 |
| EDN1 |
| GATA3 |
| GSTM1 |
| GSTP1 |
| HLA-DPB1 |
| TNC |
| IL1B |
| IL1RN |
| IL2RA |
| IL4R |
| ARG1 |
| NPSR1 |
| RNASE3 |
| STAT6 |
| VEGFA |
| CAT |
| CD14 |
| ARG2 |
| AREG |
| NQO1 |
| MMP1 |
| ALDH2 |
| CTNNA3 |
| SOD1 |
| BCL2 |
| RUNX3 |
| HMOX1 |
| PLAU |
| TRPA1 |
| IFNL3 |
| NPY |
| PTEN |
| MMP10 |
| ADCY2 |
| ADCYAP1R1 |
| MYB |
| BGLAP |
| SPRR2B |
| TIMP3 |
| CCL26 |
| DNMT1 |
| EGFR |
| NR3C1 |
| HSD11B2 |
| IL10 |
| IL17A |
| KRT19 |
| MUC5AC |
| SERPINE1 |
| IL22 |
| PDE4B |
| SERPINA1 |
| PPP2CA |
| PTGS2 |
| DPP10 |
| CCL17 |
| SCGB1A1 |
| CXCL14 |
| AGER |
| SFTPD |
| STAT4 |
| CCR4 |
| LTA4H |
| CCL22 |
| C5AR1 |
| TLR3 |
| PIN1 |
| C3AR1 |
| CD40 |
| ESR2 |
| PRMT1 |
| FAS |
| PLA2G4A |
| PTGER3 |
| TACR1 |
| C5 |
| VIP |
| CD86 |
| EDNRB |
| RHOA |
| MIF |
| NTRK1 |
| SFTPA1 |
| UTS2 |
| CCR8 |
| AGTR1 |
| AGTR2 |
| ITPR1 |
| PDGFB |
| SFTPB |
| UCN |
| PRMT3 |
| PRMT5 |
| CARM1 |
| CFTR |
| SPINK5 |
| CHAT |
| CSF2RB |
| DCN |
| F7 |
| MS4A2 |
| FGA |
| FLG |
| PRMT2 |
| ITGB6 |
| PIK3CD |
| PRF1 |
| SLC5A7 |
| BDKRB2 |
| SLC18A3 |
| SLC22A1 |
| STAT3 |
| IL18R1 |
| ZPBP2 |
| NOD2 |
| TNFSF4 |
| TLR6 |
| GSDMA |
| TLR10 |
| SMAD3 |
| ATG5 |
| ADA |
| IL1R1 |
| DENND1B |
| FCGR2A |
| ICOS |
| IL2RB |
| TLR1 |
| ADORA1 |
| IL23R |
| JAG1 |
| ICOSLG |
| IL7R |
| IRF4 |
| ITGAX |
| PTGER2 |
| SLC22A5 |
| NKX2-1 |
| COL1A1 |
| ERBB2 |
| MTOR |
| GAB1 |
| DNAI1 |
| DLL1 |
| DNAAF3 |
| ITLN1 |
| EMSY |
| BACH2 |
| RORA |
| CASP8 |
| KIAA1109 |
| GPR65 |
| PGAP3 |
| FADS2 |
| MED24 |
| CDSN |
| PSORS1C1 |
| ETS1 |
| ABCD1 |
| FCER1G |
| FBXL7 |
| CLEC16A |
| NEDD4L |
| PTPN22 |
| GLI2 |
| HLA-B |
| HLA-DPA1 |
| HLA-DRB5 |
| KLK3 |
| ITPR3 |
| KRT7 |
| FADS1 |
| LPP |
| MICB |
| NOTCH4 |
| PBX2 |
| CRIM1 |
| PLEK |
| CCHCR1 |
| ALLC |
| PROC |
| PSMD3 |
| PTCH1 |
| PTPRC |
| RAD51B |
| HAS2-AS1 |
| SLC6A12 |
| TBX1 |
| TNS1 |
| NDFIP1 |
| DOCK8 |
| RUNX1 |
| DNAH11 |
| TNFRSF11A |
| IL18RAP |
| PTGES |
| ZNF432 |
| LRBA |
| TNFSF15 |
| CCNT2-AS1 |
| FAM205C |
| LINC01387 |
| MAP3K14-AS1 |
| RNU4ATAC |
| MIR3681HG |
| MROH7-TTC4 |
| ATP6V1G2-DDX39B |
| RTEL1-TNFRSF6B |
| FMC1-LUC7L2 |
| CDH17 |
| CDK2 |
| LINC01934 |
| TH2LCRR |
| LINC01425 |
| LINC02660 |
| LINC01527 |
| IL12A-AS1 |
| LINC01149 |
| TSBP1-AS1 |
| MMADHC-DT |
| LINC01615 |
| LINC01063 |
| CCNO |
| MRVI1 |
| VAV3 |
| LINC02471 |
| LINC01862 |
| SOCAR |
| LINC02121 |
| MPPED2-AS1 |
| CEL |
| AHSA1 |
| POSTN |
| TSBP1 |
| MTCO2P12 |
| CETP |
| RNU1-79P |
| RNU1-116P |
| LRRK2-DT |
| CYSLTR1 |
| KRT7-AS |
| SLC27A5 |
| TDRKH |
| FGFR1OP |
| CHI3L1 |
| POLI |
| IL17F |
| GLCCI1 |
| ACOT7 |
| CHRNA2 |
| NLRP3 |
| ZNF618 |
| LRRC56 |
| UHRF2 |
| CCDC151 |
| RMI2 |
| TAGAP |
| PARD3B |
| CLCA1 |
| SERPINA3 |
| LRRK2 |
| BTBD11 |
| CCR5 |
| CCR6 |
| DNAAF1 |
| SPATA32 |
| COL5A1 |
| COL5A2 |
| RBM45 |
| ACMSD |
| AP1S3 |
| COMT |
| SLCO6A1 |
| UBE2QL1 |
| PIH1D3 |
| CRK |
| CRP |
| ZBTB46 |
| ADAMTS14 |
| MPP7 |
| MAPK14 |
| CSF2 |
| GLT1D1 |
| LACC1 |
| CARMIL2 |
| CTLA4 |
| CTNNB1 |
| LINC01931 |
| PUS10 |
| LINC02085 |
| JAKMIP1 |
| PLEKHG4B |
| CCDC167 |
| SAXO1 |
| SLC5A12 |
| DGKH |
| AP2A2 |
| DNAAF4 |
| EXD1 |
| ZFPM1 |
| ACE |
| PRICKLE2 |
| PRSS35 |
| SLC30A8 |
| DPH1 |
| DPP6 |
| EDNRA |
| AHR |
| LINC00528 |
| CMYA5 |
| EPHA5 |
| ERBB3 |
| ERBB4 |
| ERCC2 |
| F2RL1 |
| TMEM26 |
| FCER2 |
| JMJD1C |
| RSPH9 |
| JAZF1 |
| FER |
| FGF8 |
| FGFR1 |
| ZNF652 |
| CLSTN1 |
| ZNF365 |
| SBNO2 |
| SEPTIN8 |
| SIK2 |
| CLASP1 |
| SIK3 |
| ZFPM2 |
| CRB1 |
| LRRC6 |
| KCNE4 |
| ALOX5AP |
| GAS2L2 |
| ZBTB38 |
| ZDHHC24 |
| GABPA |
| TNFAIP8 |
| RNF19A |
| DNAH1 |
| COPD |
| POLDIP2 |
| PHF19 |
| RGS22 |
| GAS1 |
| PITPNC1 |
| GAS8 |
| HLA-DPA3 |
| HAVCR1 |
| STK36 |
| CHIA |
| IL37 |
| GLB1 |
| HPGDS |
| GLDC |
| GNAI1 |
| GNAS |
| GP1BA |
| GP1BB |
| GP9 |
| ZNF311 |
| EIF2S2P3 |
| LINC00907 |
| ZNF841 |
| LINC01091 |
| XKR6 |
| ILDR1 |
| GRID1 |
| GSTT1 |
| PDZRN4 |
| HDAC2 |
| HLA-DPB2 |
| HLA-DQA2 |
| HLA-DRB3 |
| HLA-DRB9 |
| HMGB1 |
| UBAC2 |
| FLG-AS1 |
| LINC00299 |
| CCDC39 |
| OTOG |
| IDS |
| IFNA1 |
| DUBR |
| IFNA13 |
| IFNB1 |
| IRGM |
| MCIDAS |
| ACTBL2 |
| IFNG |
| RSPH4A |
| FTCDNL1 |
| IGHE |
| IL1A |
| IL2 |
| IL3 |
| IL5RA |
| CXCL8 |
| IL9 |
| IL12B |
| IL18 |
| CXCL10 |
| ISG20 |
| ITGB8 |
| ITPKA |
| GSTK1 |
| DNAJB13 |
| MROH7 |
| NRROS |
| KNG1 |
| KIF11 |
| CCDC103 |
| KRT81 |
| C4orf50 |
| LEP |
| LIFR |
| LIG4 |
| CASC15 |
| GTF2H5 |
| LTA |
| LTC4S |
| MIR146A |
| MIR155 |
| MANBA |
| HCG18 |
| HCG17 |
| MBL2 |
| ARVCF |
| MLLT3 |
| POTEKP |
| IRF1-AS1 |
| MST1 |
| PLF |
| COX2 |
| MYLK |
| PPP1R12B |
| RERE |
| NFE2L2 |
| NFKB2 |
| NGF |
| NODAL |
| NOS1 |
| NOS3 |
| OAS2 |
| ORM1 |
| OVOL1 |
| ITSN2 |
| CDON |
| PDE11A |
| FOXP3 |
| WDPCP |
| NME8 |
| ZMYND10 |
| PDE4A |
| DDX41 |
| HDAC7 |
| LUC7L2 |
| SUFU |
| SLC26A4 |
| RAPGEF6 |
| RTEL1 |
| PEPD |
| PEX14 |
| PGM3 |
| PIK3CA |
| PIK3CB |
| PIK3CG |
| TLR9 |
| MIR4435-2HG |
| OTULINL |
| MED1 |
| FEV |
| HYDIN |
| BNC2 |
| NSUN2 |
| CDKAL1 |
| DNAAF5 |
| PIGX |
| CCDC40 |
| ATG16L1 |
| ARMC4 |
| DNAAF2 |
| VPS13D |
| KIF16B |
| TENM3 |
| LRP2BP |
| PRKCE |
| PRKCQ |
| PRKG1 |
| MAPK1 |
| MYDGF |
| BTNL2 |
| KCNQ5 |
| CFAP298 |
| ADAMTS9 |
| ACKR3 |
| CYSLTR2 |
| ZMIZ1 |
| PTGDR |
| PTGDS |
| COG6 |
| ARHGAP31 |
| PTPN2 |
| PTPRK |
| BBS1 |
| PEX5 |
| RAB5B |
| EEFSEC |
| RPGR |
| RREB1 |
| BDNF |
| ATXN2 |
| SCN4A |
| CCL24 |
| SDHD |
| POTEM |
| IKZF4 |
| GALNT17 |
| DNAI2 |
| SHH |
| MROH3P |
| IL25 |
| PMEL |
| SIX3 |
| SLC7A2 |
| SPAG1 |
| SRP9 |
| BRCA2 |
| STAT5B |
| BRS3 |
| TAC1 |
| TALDO1 |
| TAP2 |
| TBCD |
| TBXA2R |
| TDGF1 |
| TFRC |
| TGIF1 |
| TLR2 |
| TLR4 |
| ACTG1 |
| ACTG2 |
| D2HGDH |
| HIRA |
| UFD1 |
| VDR |
| TRPV1 |
| MYRF |
| TMEM258 |
| ZIC2 |
| ZBTB16 |
| EVI5 |
| ALMS1 |
| CCDC28B |
| DDX39B |
| MMEL1 |
| EFL1 |
| AIMP2 |
| TBL1XR1 |
| ARHGEF5 |
| PRR5L |
| ZNF614 |
| CCDC33 |
| SPSB1 |
| ADAM33 |
| ASXL3 |
| CAMK4 |
| POLR2M |
| TRIM8 |
| OR5V1 |
| OR12D3 |
| GATD3A |
| LZTR1 |
| ARHGAP24 |
| TTC25 |
| DNAL1 |
| PARP9 |
| RSPH3 |
| TATDN1 |
| CARD11 |
| ATRN |
| COX4I2 |
| OFD1 |
| PHF5A |
| FBXL20 |
| DISP1 |
| CFAP300 |
| ITGA8 |
| AP3B1 |
| CCDC65 |
| CDK13 |
| SERPINB7 |
| TIMELESS |
| HERC2 |
| FOXH1 |
| RSPH1 |
| PPP1R3F |
| NEK9 |
| CD247 |
| SLC16A7 |
| ARRDC1 |
| DRC1 |
| ADAMTSL1 |
| CCDC114 |
| ADIPOQ |
| GRAP2 |
| POM121L2 |
| MAP4K4 |
| FHL5 |
| AKAP6 |
| ADAMTS4 |
| SEC24C |
| SH3PXD2A |
| LPIN2 |
| GREB1 |
| PRORP |
| RIPOR2 |
| TESPA1 |
| ZBTB39 |
| CXCR6 |
| CHIT1 |
| ADRA1A |
| ADRA2B |
| GPR42 |
| IL15 |
| MPO |
| PLG |
| ANO1 |
| SSTR4 |
| TPI1 |
| LPAR2 |
| NAT2 |
| NOD1 |
| LTB4R |
| AKT1 |
| GC |
| HIF1A |
| PCDH1 |
| TLR7 |
| PPARG |
| SLC52A1 |
| PTGS1 |
| VCAM1 |
| HAVCR2 |
| NR1I2 |
| CD28 |
| WDHD1 |
| CHRM3 |
| OXER1 |
| FCGR3A |
| CXCR3 |
| IL16 |
| MIR21 |
| MMP12 |
| ISYNA1 |
| IL23A |
| ACTB |
| CCL20 |
| STAT1 |
| CAMP |
| SOCS3 |
| CD38 |
| CCR1 |
| CCN2 |
| DPP4 |
| AGT |
| EPHB2 |
| ESR1 |
| FCER1A |
| AKR1B1 |
| FN1 |
| FOS |
| FOSB |
| IL27 |
| IL19 |
| HSPA4 |
| IL9R |
| ITGB3 |
| JUN |
| JUNB |
| JUND |
| MIR221 |
| MRC1 |
| MUC1 |
| PHF11 |
| IL17D |
| LTB4R2 |
| RNASE2 |
| CXCL12 |
| SMPD1 |
| TIMP1 |
| CCR2 |
| EPX |
| PLA2G10 |
| CD34 |
| CCL27 |
| CYP2R1 |
| AGXT |
| FCGR3B |
| FKBP5 |
| MMRN1 |
| ALOX15 |
| HDAC1 |
| NRG1 |
| CXCR2 |
| IL13RA1 |
| IRF1 |
| IL31 |
| LGALS3 |
| SMAD2 |
| KITLG |
| IL17RB |
| MAPK8 |
| IL21 |
| S100A8 |
| CCL13 |
| SOD2 |
| SPP1 |
| TGFB2 |
| C3 |
| CD1D |
| IL32 |
| ADAM8 |
| TNFSF13B |
| IRAK3 |
| ADCY9 |
| MRGPRX3 |
| MRGPRX4 |
| CMA1 |
| COL4A5 |
| GPR151 |
| CPA3 |
| CREB1 |
| CRHR1 |
| CSF3 |
| CX3CR1 |
| CYP2C19 |
| CYP24A1 |
| CYP27B1 |
| DEFB1 |
| DUSP1 |
| EGR1 |
| ELANE |
| EPHX1 |
| F3 |
| GPRC6A |
| FGF2 |
| SIRT1 |
| LPAR3 |
| IL17RA |
| MRGPRX1 |
| GCG |
| IFNL1 |
| H19 |
| LAMA1 |
| GSTA1 |
| HLA-DRA |
| HRH1 |
| IL7 |
| IL11 |
| IL13RA2 |
| ITGB4 |
| ITK |
| LEPR |
| LGALS9 |
| MIR126 |
| MIR145 |
| MAS1 |
| VN1R17P |
| GPR166P |
| MTHFR |
| MYD88 |
| ATP5F1A |
| PLA2G2A |
| SERPINF2 |
| POMC |
| SAGE1 |
| MAPK3 |
| TRPV4 |
| LGR6 |
| NPS |
| SERPINB3 |
| SERPINB4 |
| SCD |
| CCL18 |
| CX3CL1 |
| ECSCR |
| ST2 |
| TNFAIP3 |
| TPT1 |
| MUC5B |
| POTEF |
| TNFRSF4 |
| TRPM8 |
| TNFAIP8L2 |
| CD276 |
| SLC2A10 |
| FZD4 |
| CASP1 |
| MBD2 |
| TIMD4 |
| CD19 |
| ENTPD1 |
| FAM13A |
| ADAM10 |
| TUBB4B |
| RACK1 |
| FSTL1 |
| CHRM1 |
| CLC |
| GSTO2 |
| CMD1B |
| CCR7 |
| ADH5 |
| COX8A |
| CPOX |
| CYBA |
| DECR1 |
| EGF |
| FCGR2B |
| FDXR |
| FKBP4 |
| ATF6 |
| FLNC |
| TNFRSF13B |
| GAPDH |
| GGT1 |
| SIGLEC8 |
| ABO |
| ANGPT1 |
| GSTM2 |
| HLX |
| HP |
| APOA1 |
| IGF1 |
| IGF1R |
| LINC01193 |
| IGHG3 |
| APRT |
| IL12A |
| IRAK1 |
| IRF5 |
| ITGAM |
| JAK3 |
| LAG3 |
| LIF |
| LTB |
| MIR152 |
| MIR19A |
| ARSA |
| MFAP1 |
| MMP2 |
| MUC2 |
| NEUROD1 |
| NFKB1 |
| NM |
| P2RX7 |
| SERPINB2 |
| ACP1 |
| PLAUR |
| TREM1 |
| PON1 |
| PTPA |
| PRKCA |
| AXL |
| PTAFR |
| PTGER4 |
| KIDINS220 |
| PTPN11 |
| RAC1 |
| MOK |
| CCND1 |
| RGS2 |
| S100A9 |
| CCL19 |
| CCL21 |
| SELE |
| CRLF2 |
| IFIH1 |
| SMARCA4 |
| SOAT1 |
| TACR2 |
| THBS1 |
| TSPO |
| TRAF6 |
| YY1 |
| CXCR4 |
| CALCA |
| ELP1 |
| TP63 |
| BECN1 |
| MS4A3 |
| COX5A |
| CD48 |
| CD68 |
| CD69 |
| HDAC9 |
| CDC42 |
| CDH1 |
| SIGLEC14 |
| MIR744 |
| HDAC6 |
| LINC01672 |
| ABCB6 |
| PGR-AS1 |
| LRPPRC |
| EBI3 |
| RAMP1 |
| CDKN1B |
| LOC102723996 |
| SEMA3A |
| KAT5 |
| AGR2 |
| STIP1 |
| CKAP4 |
| PRRT2 |
| CHRM2 |
| CHRNA3 |
| SLC26A9 |
| FCRL3 |
| RASGRP4 |
| MRGPRX2 |
| CLU |
| FRMD6 |
| COL4A3 |
| DCBLD2 |
| GNPDA2 |
| ADM |
| PPARGC1B |
| ADORA2A |
| COL26A1 |
| CR1 |
| CREBBP |
| CRHR2 |
| CTSS |
| CYBB |
| CYLD |
| CYP1A2 |
| CYP2B6 |
| DNASE1L3 |
| SLC26A3 |
| ABCA1 |
| S1PR1 |
| ELF5 |
| EREG |
| F2R |
| ACSL3 |
| PTK2B |
| FBLN1 |
| FCGRT |
| FGF1 |
| NLRP1 |
| FOXM1 |
| FOXO1 |
| FOXO3 |
| CEP68 |
| TPSD1 |
| BRD4 |
| FOSL2 |
| FPR2 |
| OPN3 |
| ALPL |
| ACKR1 |
| TAC4 |
| ARIH1 |
| ALRH |
| LRRC32 |
| GATA1 |
| FBXO8 |
| EHF |
| GJA1 |
| ACAD8 |
| LAT |
| AGO2 |
| IFNL2 |
| RGMB |
| FLVCR1 |
| ANPEP |
| CD274 |
| CXCL1 |
| ANXA1 |
| ANXA2 |
| KCNIP3 |
| HLA-A |
| AOAH |
| HMGCR |
| FOXA2 |
| HSPA1B |
| HSP90AA1 |
| IGES |
| APOE |
| IGFBP3 |
| IGHG1 |
| IGHG2 |
| APP |
| IL6ST |
| IL12RB1 |
| TNFRSF9 |
| IDO1 |
| INPP4A |
| INSRR |
| IRF7 |
| JAK1 |
| KIT |
| KLK1 |
| LAMA5 |
| ARNTL |
| MIR142 |
| MIR192 |
| MIR200A |
| MIR216A |
| MIR22 |
| MARCKS |
| MCAM |
| MEFV |
| MET |
| CXCL9 |
| ACHE |
| CD200 |
| ABCC1 |
| COX1 |
| MUSK |
| MYC |
| NFATC2 |
| NHS |
| NOTCH1 |
| NTF4 |
| TNFRSF11B |
| OSM |
| ALDH7A1 |
| PEBP1 |
| NOX4 |
| IL21R |
| MBL3P |
| TAS2R14 |
| PCBD1 |
| PCNA |
| IRAK4 |
| CKLF |
| CPA4 |
| BPIFA1 |
| TLR8 |
| PDCD1 |
| PECAM1 |
| SERPINE2 |
| PLD1 |
| PMCH |
| PNOC |
| TOLLIP |
| PARP14 |
| PPARA |
| DYM |
| GIMAP5 |
| PRG2 |
| KLK15 |
| PRKCB |
| EAF2 |
| USE1 |
| GABRQ |
| PSMB6 |
| AICDA |
| MIR499A |
| PTPN6 |
| PTPRD |
| PTPRE |
| PVT1 |
| RAPSN |
| ACE2 |
| SIGIRR |
| RBP4 |
| RGS4 |
| BCL6 |
| ROS1 |
| RPE65 |
| FIQTL1 |
| RRBP1 |
| CLEC11A |
| CCL1 |
| CCL3 |
| CCL3L1 |
| CXCL11 |
| XCL1 |
| SELL |
| SELP |
| SEMA4A |
| ARHGEF28 |
| CLEC7A |
| BCL11B |
| UBE2Z |
| SLC6A4 |
| GGTLC5P |
| SLC8A1 |
| SMN1 |
| SMN2 |
| SNRNP70 |
| TAC3 |
| TAP1 |
| TCF7 |
| MIR625 |
| TRBV20OR9-2 |
| TEK |
| TFF2 |
| TGFBR2 |
| TGM2 |
| THBD |
| TLE4 |
| SERPING1 |
| TM7SF2 |
| TP53 |
| TPSAB1 |
| TRPC3 |
| GGTLC3 |
| GGT2 |
| TWIST1 |
| SFTPA2 |
| GGTLC4P |
| TYRO3 |
| UVRAG |
| EZR |
| XBP1 |
| FTO |
| EOS |
| FSD1 |
| GSDMD |
| NR4A3 |
| FLAD1 |
| TET1 |
| FOSL1 |
| ADAMTS12 |
| CRISPLD2 |
| FSD1L |
| TAGLN2 |
| NR0B2 |
| IL1F10 |
| RETNLB |
| ORAI1 |
| USO1 |
| SOCS1 |
| CBL |
| TNFSF10 |
| TNFRSF14 |
| RIPK2 |
| VNN1 |
| FCGR2C |
| MSC |
| DNER |
| CD163 |
| CD27 |
| TNFRSF8 |
| CLOCK |
| CD44 |
| UBE3C |
| SEMA3E |
| HDAC4 |
| MIR148A |
| MIR148B |
| ASRT4 |
| ASRT3 |
| ASRT6 |
| ASRT8 |
| NPSR1-AS1 |
| ASOBS |
| CD40LG |
| CCL7 |
| CSF1 |
| BMP6 |
| NPPA |
| CD4 |
| PPBP |
| PF4 |
| JAK2 |
| IL12RB2 |
| CD79A |
| SYK |
| ADRA1B |
| NFKBIA |
| ADORA2B |
| FASLG |
| CXCR1 |
| CYP3A4 |
| CP |
| ITGA4 |
| GSR |
| FGFBP2 |
| CD80 |
| ABCB1 |
| CCL4 |
| CXCL5 |
| HLA-DOA |
| ADCY10 |
| CRH |
| G6PD |
| RAPGEF3 |
| GHRL |
| TF |
| MME |
| ADRB1 |
| VTN |
| CYP2C9 |
| TNFRSF10A |
| P2RY12 |
| THPO |
| GAL |
| LPO |
| OPRM1 |
| ITGA2 |
| SP1 |
| HRH2 |
| F2 |
| INS |
| F2RL3 |
| SST |
| SLC22A2 |
| RYR2 |
| GNRH1 |
| GRK5 |
| ADCYAP1 |
| EIF4E |
| PITX2 |
| SLC22A3 |
| ADORA3 |
| MB |
| HBG2 |
| SERPINA6 |
| CYP3A5 |
| ADRB3 |
| SDHB |
| ATOD1 |
| ITGB2 |
| CLEC1A |
| TGFBR1 |
| WDR46 |
| ATOD3 |
| PLCG2 |
| EP300 |
| POLR1H |
| RIC1 |
| CPN1 |
| ALB |
| PDGFRA |
| ODC1 |
| GRHL2 |
| AFF4 |
| ELOVL4 |
| TRAIP |
| VPS51 |
| TET2 |
| ASXL1 |
| SRSF2 |
| TBCK |
| FBN1 |
| LRP1 |
| USP7 |
| STXBP1 |
| IGF2 |
| CDKN1C |
| LMX1B |
| HMGA2 |
| ARL6 |
| WAC |
| PLAG1 |
| ODAD3 |
| CYFIP2 |
| ERMP1 |
| NSD1 |
| ENPP3 |
| GMPPA |
| ASIC4-AS1 |
| GER |
| GPT |
| HLA-C |
| PLOD1 |
| NEU1 |
| MMP3 |
| ATP12A |
| PAFAH1B1 |
| BBS9 |
| ATP4A |
| SFTA3 |
| STAT5A |
| USB1 |
| CFTR-AS1 |
| ELN |
| IFNGR2 |
| IFNGR1 |
| RANBP6 |
| NTF3 |
| GZMB |
| BBS7 |
| OXA1L |
| LEPQTL1 |
| MPLKIP |
| DEL18Q |
| SLC25A46 |
| BBS12 |
| ITGAL |
| CHUK |
| SLPI |
| HRH4 |
| ANXA5 |
| CTSG |
| CD8A |
| CEP19 |
| FURIN |
| LTF |
| PTX3 |
| C4A |
| NFKBIL1 |
| CTCF |
| IVL |
| PRTN3 |
| BBS2 |
| BBS4 |
| HARS1 |
| C19orf12 |
| EOE1 |
| SERPINB1 |
| FMR1 |
| PRKCZ |
| HTR2A |
| CSN1S1 |
| MLX |
| WDR19 |
| CD209 |
| PI3 |
| SFTPC |
| BBS10 |
| BBS5 |
| TNXB |
| LCN2 |
| PIK3C2A |
| CYP1A1 |
| MMP8 |
| TLR5 |
| TXN |
| PRL |
| GHITM |
| MAVS |
| CD36 |
| TBXT |
| FLT1 |
| PTGER1 |
| SOCS2 |
| IRF3 |
| RETN |
| SUOX |
| CSTA |
| S100A12 |
| HDC |
| IFRD1 |
| NTS |
| KLK7 |
| IFNA2 |
| AOC1 |
| IL10RA |
| MICA |
| CAV1 |
| LORICRIN |
| MIR31 |
| DDX58 |
| FCGR1A |
| SDCCAG8 |
| CLIC2 |
| RAB39B |
| TTLL1 |
| SPATS2L |
| DNAH8 |
| LYN |
| CRCT1 |
| RORC |
| HERC5 |
| RPS27A |
| MIR338 |
| POU2F1 |
| EPRS1 |
| IL21R-AS1 |
| MYH11 |
| GSTO1 |
| ITGB1 |
| PRKAA2 |
| TNFRSF1A |
| TPSB2 |
| PRNP |
| RBM17 |
| LTBP1 |
| STAT2 |
| SLC22A4 |
| AQP5 |
| EPHX2 |
| IGSF3 |
| STX1A |
| ALAD |
| TRIM32 |
| NPHP1 |
| ATRIP |
| TREX1 |
| TGM5 |
| SUZ12 |
| KIF7 |
| TTC8 |
| LZTFL1 |
| MKKS |
| DCTN4 |
| MIA3 |
| CLCA4 |
| NCKAP1L |
| ASTN2 |
| IFT27 |
| ARSL |
| BBIP1 |
| DOP1A |
| DOCK8-AS1 |
| MTOR-AS1 |
| SEPT5-GP1BB |
| LOC105373021 |
| ATRIP-TREX1 |
| GH-LCR |
| LOC107303343 |
| LOC113633877 |
| LOC113664106 |
| LOC111674463 |
| LOC111674472 |
| LOC111674475 |
| LOC111674477 |
| LOC106050102 |
| DEL16P13.3 |
| DUPXQ28 |
| LBR |
| HTR3A |
| CYP27A1 |
| PTGIR |
| NEGR1 |
| PSIP1 |
| FLG2 |
| LY86 |
| GPX4 |
| ZBTB10 |
| GZMA |
| NEAT1 |
| CFL1 |
| XDH |
| EDIL3 |
| ATP2A2 |
| ARRB2 |
| IL18BP |
| DEFB4A |
| LBP |
| KDR |
| ICAM3 |
| MUC22 |
| SETDB2 |
| CEBPB |
| SLC6A11 |
| CCR10 |
| SH2B3 |
| KRT14 |
| HRNR |
| BRD2 |
| ATF6B |
| HCG23 |
| NTRK3 |
| LOC100287329 |
| GPX3 |
| IL20 |
| CPS1 |
| MIR196A2 |
| SELPLG |
| GPX1 |
| INSIG2 |
| CD63 |
| TNFRSF10B |
| UGRP1 |
| TNFA |
| TBET |
| SCYA11 |
| PAFAH |
| MRT51 |
| IRAKM |
| GPR154 |
| GP39 |
| AS1 |
| YKL40 |
| PAFAD |
| GPRA |
| BHR1 |
| ASRT5 |
| ASRT1 |
| VRR1 |
| ASRT7 |
| PGR14 |
| ASRT2 |
| USP38 |
| SPRED1 |
| NPSRAS1 |
| SART1 |
| GAD1 |
| TNFSF14 |
| DAP3 |
| GABRB2 |
| TRBC1 |
| GAD2 |
| GABRA2 |
| SLCO1B3 |
| SLC6A2 |
| SLC6A3 |
| CES1 |
| S100P |
| ABCB11 |
| CYP17A1 |
| NR0B1 |
| CYP4A11 |
| MAOA |
| MAOB |
| NR3C2 |
| SLCO1B1 |
| UGT2B7 |
| UGT2B15 |
| UGT1A8 |
| GRIN2D |
| GRIN3B |
| GRIN1 |
| GRIN2A |
| GRIN2B |
| GRIN2C |
| GRIN3A |
| SHBG |
| CYP11B1 |
| CYP11B2 |
| SRD5A2 |
| AKR1D1 |
| SLC22A8 |
| UGT1A1 |
| GLT6D1 |
| GLTP |
| AKR1C4 |
| AKR1C3 |
| AKR1C2 |
| AKR1C1 |
| PGR |
| SLCO2B1 |
| FPR1 |
| ABCC2 |
| CYP2A6 |
| HSD11B1 |
| SLCO1A2 |
| CYP3A43 |
| ADRA2A |
| ADRA2C |
| ADRA1D |
| ORM2 |
| SLC25A1 |
| SLC13A2 |
| SLC13A5 |
| SLC25A21 |
| PDE5A |
| PDE3A |
| NOMO1 |
| CYP1B1 |
| RIC3 |
| HM13 |
| CPNE1 |
| TTLL3 |
| SLC22A7 |
| CHRM4 |
| CHRM5 |
| CYP2D6 |
| BCHE |
| CYP3A7 |
| CYP2E1 |
| CYP2C8 |
| ABCG2 |
| UGT1A9 |

Supplementary Table 5: Node interactions in the PPI network.

| Node1 | String ID of Node 1 | Node2 | String ID of Node 2 | Co-expression | Experimentally determined interaction | Combined score |
| --- | --- | --- | --- | --- | --- | --- |
| ABCC1 | 9606.ENSP00000382342 | ABCG2 | 9606.ENSP00000237612 | 0.062 | 0.091 | 0.943 |
| ADA | 9606.ENSP00000361965 | ADORA1 | 9606.ENSP00000356205 | 0 | 0.388 | 0.911 |
| ADAM10 | 9606.ENSP00000260408 | PTAFR | 9606.ENSP00000362965 | 0 | 0 | 0.906 |
| ADAM10 | 9606.ENSP00000260408 | PLAU | 9606.ENSP00000361850 | 0.046 | 0 | 0.92 |
| ADAM10 | 9606.ENSP00000260408 | IL6 | 9606.ENSP00000385675 | 0 | 0 | 0.941 |
| ADAM10 | 9606.ENSP00000260408 | APP | 9606.ENSP00000284981 | 0.062 | 0.19 | 0.988 |
| ADORA1 | 9606.ENSP00000356205 | APP | 9606.ENSP00000284981 | 0.139 | 0 | 0.929 |
| ADORA1 | 9606.ENSP00000356205 | CXCR1 | 9606.ENSP00000295683 | 0 | 0 | 0.904 |
| ADORA1 | 9606.ENSP00000356205 | CXCR2 | 9606.ENSP00000319635 | 0 | 0 | 0.905 |
| ADORA1 | 9606.ENSP00000356205 | PTGDR2 | 9606.ENSP00000332812 | 0 | 0 | 0.913 |
| ADORA1 | 9606.ENSP00000356205 | CHRM2 | 9606.ENSP00000399745 | 0.062 | 0 | 0.903 |
| ADORA1 | 9606.ENSP00000356205 | OPRM1 | 9606.ENSP00000394624 | 0 | 0 | 0.92 |
| ADORA1 | 9606.ENSP00000356205 | ADORA3 | 9606.ENSP00000358730 | 0 | 0 | 0.92 |
| ADORA2B | 9606.ENSP00000304501 | PTGER2 | 9606.ENSP00000245457 | 0.061 | 0 | 0.92 |
| ADORA2B | 9606.ENSP00000304501 | PTGIR | 9606.ENSP00000291294 | 0.061 | 0 | 0.916 |
| ADORA2B | 9606.ENSP00000304501 | PTGER4 | 9606.ENSP00000302846 | 0 | 0 | 0.92 |
| ADORA2B | 9606.ENSP00000304501 | PTGDR | 9606.ENSP00000303424 | 0 | 0 | 0.907 |
| ADORA3 | 9606.ENSP00000358730 | APP | 9606.ENSP00000284981 | 0 | 0 | 0.923 |
| ADORA3 | 9606.ENSP00000358730 | CXCR1 | 9606.ENSP00000295683 | 0.125 | 0 | 0.918 |
| ADORA3 | 9606.ENSP00000358730 | CXCR2 | 9606.ENSP00000319635 | 0.065 | 0 | 0.908 |
| ADORA3 | 9606.ENSP00000358730 | PTGDR2 | 9606.ENSP00000332812 | 0 | 0 | 0.913 |
| ADORA3 | 9606.ENSP00000358730 | CHRM2 | 9606.ENSP00000399745 | 0 | 0 | 0.9 |
| ADORA3 | 9606.ENSP00000358730 | OPRM1 | 9606.ENSP00000394624 | 0 | 0 | 0.95 |
| AHR | 9606.ENSP00000242057 | CYP1B1 | 9606.ENSP00000478561 | 0.095 | 0 | 0.943 |
| AHR | 9606.ENSP00000242057 | CYP1A1 | 9606.ENSP00000369050 | 0.061 | 0 | 0.997 |
| AKR1B1 | 9606.ENSP00000285930 | AKR1C3 | 9606.ENSP00000369927 | 0 | 0 | 0.906 |
| AKR1C1 | 9606.ENSP00000370254 | CYP11B1 | 9606.ENSP00000292427 | 0.061 | 0 | 0.907 |
| AKR1C1 | 9606.ENSP00000370254 | CYP11B2 | 9606.ENSP00000325822 | 0.061 | 0 | 0.905 |
| AKR1C1 | 9606.ENSP00000370254 | CYP3A4 | 9606.ENSP00000337915 | 0.062 | 0 | 0.921 |
| AKR1C1 | 9606.ENSP00000370254 | CYP17A1 | 9606.ENSP00000358903 | 0.061 | 0 | 0.938 |
| AKR1C1 | 9606.ENSP00000370254 | CYP1A1 | 9606.ENSP00000369050 | 0.062 | 0 | 0.939 |
| AKR1C1 | 9606.ENSP00000370254 | AKR1C3 | 9606.ENSP00000369927 | 0.214 | 0.943 | 0.995 |
| AKR1C1 | 9606.ENSP00000370254 | AKR1C2 | 9606.ENSP00000370129 | 0.405 | 0.86 | 0.991 |
| AKR1C1 | 9606.ENSP00000370254 | SRD5A2 | 9606.ENSP00000477587 | 0.051 | 0 | 0.952 |
| AKR1C2 | 9606.ENSP00000370129 | AKR1C3 | 9606.ENSP00000369927 | 0.214 | 0.912 | 0.985 |
| AKR1C2 | 9606.ENSP00000370129 | SRD5A2 | 9606.ENSP00000477587 | 0.051 | 0 | 0.959 |
| AKR1C3 | 9606.ENSP00000369927 | CYP11B1 | 9606.ENSP00000292427 | 0.061 | 0 | 0.937 |
| AKR1C3 | 9606.ENSP00000369927 | CYP11B2 | 9606.ENSP00000325822 | 0.061 | 0 | 0.928 |
| AKR1C3 | 9606.ENSP00000369927 | CYP3A4 | 9606.ENSP00000337915 | 0.062 | 0 | 0.925 |
| AKR1C3 | 9606.ENSP00000369927 | CYP1A2 | 9606.ENSP00000342007 | 0.061 | 0 | 0.913 |
| AKR1C3 | 9606.ENSP00000369927 | CYP17A1 | 9606.ENSP00000358903 | 0.061 | 0 | 0.966 |
| AKR1C3 | 9606.ENSP00000369927 | CYP1A1 | 9606.ENSP00000369050 | 0.062 | 0 | 0.928 |
| AKR1C3 | 9606.ENSP00000369927 | AKR1C4 | 9606.ENSP00000369814 | 0.076 | 0.933 | 0.936 |
| AKR1C3 | 9606.ENSP00000369927 | CYP1B1 | 9606.ENSP00000478561 | 0.061 | 0 | 0.933 |
| AKR1C3 | 9606.ENSP00000369927 | SRD5A2 | 9606.ENSP00000477587 | 0.051 | 0 | 0.967 |
| AKR1C4 | 9606.ENSP00000369814 | HSD11B1 | 9606.ENSP00000355995 | 0.062 | 0 | 0.944 |
| AKR1C4 | 9606.ENSP00000369814 | SRD5A2 | 9606.ENSP00000477587 | 0.074 | 0 | 0.944 |
| AKT1 | 9606.ENSP00000451828 | MAPK1 | 9606.ENSP00000215832 | 0.066 | 0.146 | 0.905 |
| AKT1 | 9606.ENSP00000451828 | CCND1 | 9606.ENSP00000227507 | 0.061 | 0 | 0.943 |
| AKT1 | 9606.ENSP00000451828 | GAPDH | 9606.ENSP00000229239 | 0.046 | 0.349 | 0.95 |
| AKT1 | 9606.ENSP00000451828 | MAPK14 | 9606.ENSP00000229795 | 0.082 | 0.715 | 0.931 |
| AKT1 | 9606.ENSP00000451828 | PIK3CA | 9606.ENSP00000263967 | 0.063 | 0.468 | 0.998 |
| AKT1 | 9606.ENSP00000451828 | STAT3 | 9606.ENSP00000264657 | 0 | 0.124 | 0.994 |
| AKT1 | 9606.ENSP00000451828 | PIK3CB | 9606.ENSP00000289153 | 0.063 | 0.464 | 0.986 |
| AKT1 | 9606.ENSP00000451828 | NOS2 | 9606.ENSP00000327251 | 0.063 | 0.065 | 0.909 |
| AKT1 | 9606.ENSP00000451828 | PTPN11 | 9606.ENSP00000340944 | 0.088 | 0.16 | 0.976 |
| AKT1 | 9606.ENSP00000451828 | JAK1 | 9606.ENSP00000343204 | 0.062 | 0 | 0.929 |
| AKT1 | 9606.ENSP00000451828 | PIK3CG | 9606.ENSP00000352121 | 0.063 | 0.174 | 0.979 |
| AKT1 | 9606.ENSP00000451828 | MTOR | 9606.ENSP00000354558 | 0.08 | 0.872 | 0.999 |
| AKT1 | 9606.ENSP00000451828 | PIK3CD | 9606.ENSP00000366563 | 0.063 | 0.179 | 0.945 |
| AKT1 | 9606.ENSP00000451828 | BCL2 | 9606.ENSP00000381185 | 0.061 | 0 | 0.941 |
| AKT1 | 9606.ENSP00000451828 | JAK3 | 9606.ENSP00000391676 | 0.05 | 0 | 0.924 |
| AKT1 | 9606.ENSP00000451828 | ESR1 | 9606.ENSP00000405330 | 0.055 | 0.407 | 0.991 |
| AKT1 | 9606.ENSP00000451828 | LYN | 9606.ENSP00000428924 | 0.05 | 0 | 0.94 |
| ALDH2 | 9606.ENSP00000261733 | MAOB | 9606.ENSP00000367309 | 0.083 | 0 | 0.934 |
| ALDH2 | 9606.ENSP00000261733 | MAOA | 9606.ENSP00000340684 | 0.064 | 0 | 0.942 |
| ALOX15 | 9606.ENSP00000458832 | CYP2C9 | 9606.ENSP00000260682 | 0 | 0 | 0.922 |
| ALOX15 | 9606.ENSP00000458832 | CYP3A4 | 9606.ENSP00000337915 | 0 | 0 | 0.904 |
| ALOX15 | 9606.ENSP00000458832 | CYP1A2 | 9606.ENSP00000342007 | 0 | 0 | 0.901 |
| ALOX15 | 9606.ENSP00000458832 | PTGS1 | 9606.ENSP00000354612 | 0.061 | 0 | 0.958 |
| ALOX15 | 9606.ENSP00000458832 | PTGS2 | 9606.ENSP00000356438 | 0.061 | 0 | 0.959 |
| ALOX15 | 9606.ENSP00000458832 | CYP2C19 | 9606.ENSP00000360372 | 0 | 0 | 0.916 |
| ALOX15 | 9606.ENSP00000458832 | ALOX5 | 9606.ENSP00000363512 | 0.062 | 0 | 0.912 |
| ALOX5 | 9606.ENSP00000363512 | CYP2C9 | 9606.ENSP00000260682 | 0 | 0 | 0.926 |
| ALOX5 | 9606.ENSP00000363512 | PTGS1 | 9606.ENSP00000354612 | 0.095 | 0 | 0.973 |
| ALOX5 | 9606.ENSP00000363512 | PTGS2 | 9606.ENSP00000356438 | 0.08 | 0 | 0.975 |
| ALOX5 | 9606.ENSP00000363512 | CYP2C19 | 9606.ENSP00000360372 | 0 | 0 | 0.917 |
| ALOX5 | 9606.ENSP00000363512 | ALOX5AP | 9606.ENSP00000479870 | 0.32 | 0.521 | 0.997 |
| APP | 9606.ENSP00000284981 | MAPK1 | 9606.ENSP00000215832 | 0.062 | 0.08 | 0.95 |
| APP | 9606.ENSP00000284981 | SERPINE1 | 9606.ENSP00000223095 | 0 | 0 | 0.921 |
| APP | 9606.ENSP00000284981 | GAPDH | 9606.ENSP00000229239 | 0 | 0.379 | 0.966 |
| APP | 9606.ENSP00000284981 | PIK3CA | 9606.ENSP00000263967 | 0 | 0 | 0.908 |
| APP | 9606.ENSP00000284981 | PTGDR2 | 9606.ENSP00000332812 | 0 | 0 | 0.9 |
| APP | 9606.ENSP00000284981 | CXCR1 | 9606.ENSP00000295683 | 0 | 0 | 0.902 |
| APP | 9606.ENSP00000284981 | PTAFR | 9606.ENSP00000362965 | 0 | 0 | 0.903 |
| APP | 9606.ENSP00000284981 | LTB4R | 9606.ENSP00000380008 | 0 | 0 | 0.904 |
| APP | 9606.ENSP00000284981 | CHRM2 | 9606.ENSP00000399745 | 0.062 | 0 | 0.912 |
| APP | 9606.ENSP00000284981 | OPRM1 | 9606.ENSP00000394624 | 0 | 0 | 0.916 |
| APP | 9606.ENSP00000284981 | CXCR2 | 9606.ENSP00000319635 | 0 | 0 | 0.917 |
| APP | 9606.ENSP00000284981 | PLG | 9606.ENSP00000308938 | 0 | 0.132 | 0.941 |
| APP | 9606.ENSP00000284981 | F2 | 9606.ENSP00000308541 | 0 | 0.132 | 0.944 |
| APP | 9606.ENSP00000284981 | IL6 | 9606.ENSP00000385675 | 0 | 0 | 0.957 |
| ARG1 | 9606.ENSP00000349446 | MAPK1 | 9606.ENSP00000215832 | 0 | 0 | 0.918 |
| ARG1 | 9606.ENSP00000349446 | MPO | 9606.ENSP00000225275 | 0.102 | 0 | 0.94 |
| ARG1 | 9606.ENSP00000349446 | ODC1 | 9606.ENSP00000234111 | 0.064 | 0 | 0.975 |
| ARG1 | 9606.ENSP00000349446 | MMP8 | 9606.ENSP00000236826 | 0.142 | 0 | 0.95 |
| ARG1 | 9606.ENSP00000349446 | NOS2 | 9606.ENSP00000327251 | 0.06 | 0.086 | 0.978 |
| ARG1 | 9606.ENSP00000349446 | ELANE | 9606.ENSP00000466090 | 0.108 | 0 | 0.928 |
| ARG1 | 9606.ENSP00000349446 | PTPN6 | 9606.ENSP00000391592 | 0.062 | 0 | 0.942 |
| AXL | 9606.ENSP00000301178 | KDR | 9606.ENSP00000263923 | 0.061 | 0.096 | 0.928 |
| AXL | 9606.ENSP00000301178 | PIK3CA | 9606.ENSP00000263967 | 0.058 | 0.124 | 0.948 |
| AXL | 9606.ENSP00000301178 | PIK3CB | 9606.ENSP00000289153 | 0.058 | 0.124 | 0.913 |
| BCL2 | 9606.ENSP00000381185 | MAPK1 | 9606.ENSP00000215832 | 0 | 0.379 | 0.945 |
| BCL2 | 9606.ENSP00000381185 | STAT3 | 9606.ENSP00000264657 | 0 | 0.186 | 0.931 |
| BCL2 | 9606.ENSP00000381185 | JAK1 | 9606.ENSP00000343204 | 0 | 0 | 0.904 |
| BCL2 | 9606.ENSP00000381185 | JAK3 | 9606.ENSP00000391676 | 0 | 0 | 0.902 |
| BCL2 | 9606.ENSP00000381185 | ESR1 | 9606.ENSP00000405330 | 0.069 | 0.064 | 0.925 |
| BRD2 | 9606.ENSP00000378704 | CCND1 | 9606.ENSP00000227507 | 0.062 | 0 | 0.953 |
| CCND1 | 9606.ENSP00000227507 | EGFR | 9606.ENSP00000275493 | 0.2 | 0.157 | 0.917 |
| CCND1 | 9606.ENSP00000227507 | LYN | 9606.ENSP00000428924 | 0 | 0 | 0.925 |
| CCND1 | 9606.ENSP00000227507 | JAK2 | 9606.ENSP00000371067 | 0 | 0 | 0.96 |
| CCND1 | 9606.ENSP00000227507 | ESR1 | 9606.ENSP00000405330 | 0 | 0.384 | 0.99 |
| CCND1 | 9606.ENSP00000227507 | STAT3 | 9606.ENSP00000264657 | 0.049 | 0.457 | 0.993 |
| CCND1 | 9606.ENSP00000227507 | CDK2 | 9606.ENSP00000266970 | 0.089 | 0.845 | 0.999 |
| CDK2 | 9606.ENSP00000266970 | MAPK1 | 9606.ENSP00000215832 | 0.049 | 0.261 | 0.933 |
| CDK2 | 9606.ENSP00000266970 | PTGDR | 9606.ENSP00000303424 | 0 | 0 | 0.904 |
| CFTR | 9606.ENSP00000003084 | CHRM2 | 9606.ENSP00000399745 | 0 | 0 | 0.902 |
| CFTR | 9606.ENSP00000003084 | F2 | 9606.ENSP00000308541 | 0 | 0 | 0.921 |
| CFTR | 9606.ENSP00000003084 | EGFR | 9606.ENSP00000275493 | 0 | 0.051 | 0.936 |
| CFTR | 9606.ENSP00000003084 | EZR | 9606.ENSP00000356042 | 0 | 0.379 | 0.97 |
| CHRM2 | 9606.ENSP00000399745 | EGFR | 9606.ENSP00000275493 | 0.061 | 0 | 0.905 |
| CHRM2 | 9606.ENSP00000399745 | CXCR1 | 9606.ENSP00000295683 | 0 | 0 | 0.901 |
| CHRM2 | 9606.ENSP00000399745 | CXCR2 | 9606.ENSP00000319635 | 0 | 0 | 0.9 |
| CHRM2 | 9606.ENSP00000399745 | PTGDR2 | 9606.ENSP00000332812 | 0 | 0 | 0.902 |
| CHRM2 | 9606.ENSP00000399745 | OPRM1 | 9606.ENSP00000394624 | 0 | 0 | 0.918 |
| CXCR1 | 9606.ENSP00000295683 | OPRM1 | 9606.ENSP00000394624 | 0 | 0 | 0.904 |
| CXCR1 | 9606.ENSP00000295683 | PTGDR2 | 9606.ENSP00000332812 | 0 | 0 | 0.905 |
| CXCR1 | 9606.ENSP00000295683 | LYN | 9606.ENSP00000428924 | 0.076 | 0.072 | 0.914 |
| CXCR1 | 9606.ENSP00000295683 | CXCR2 | 9606.ENSP00000319635 | 0.669 | 0.379 | 0.978 |
| CXCR2 | 9606.ENSP00000319635 | PTGDR2 | 9606.ENSP00000332812 | 0 | 0 | 0.905 |
| CXCR2 | 9606.ENSP00000319635 | OPRM1 | 9606.ENSP00000394624 | 0 | 0 | 0.908 |
| CYP11B1 | 9606.ENSP00000292427 | SRD5A2 | 9606.ENSP00000477587 | 0 | 0 | 0.936 |
| CYP11B1 | 9606.ENSP00000292427 | CYP11B2 | 9606.ENSP00000325822 | 0.181 | 0.379 | 0.945 |
| CYP11B1 | 9606.ENSP00000292427 | CYP17A1 | 9606.ENSP00000358903 | 0.062 | 0 | 0.95 |
| CYP11B1 | 9606.ENSP00000292427 | HSD11B1 | 9606.ENSP00000355995 | 0 | 0.059 | 0.954 |
| CYP11B1 | 9606.ENSP00000292427 | HSD11B2 | 9606.ENSP00000316786 | 0 | 0.064 | 0.967 |
| CYP11B2 | 9606.ENSP00000325822 | HSD11B2 | 9606.ENSP00000316786 | 0 | 0.064 | 0.969 |
| CYP11B2 | 9606.ENSP00000325822 | SRD5A2 | 9606.ENSP00000477587 | 0 | 0 | 0.926 |
| CYP11B2 | 9606.ENSP00000325822 | HSD11B1 | 9606.ENSP00000355995 | 0 | 0.059 | 0.946 |
| CYP11B2 | 9606.ENSP00000325822 | CYP17A1 | 9606.ENSP00000358903 | 0.062 | 0 | 0.949 |
| CYP17A1 | 9606.ENSP00000358903 | CYP3A4 | 9606.ENSP00000337915 | 0.063 | 0 | 0.935 |
| CYP17A1 | 9606.ENSP00000358903 | CYP1A2 | 9606.ENSP00000342007 | 0.062 | 0 | 0.92 |
| CYP17A1 | 9606.ENSP00000358903 | CYP1A1 | 9606.ENSP00000369050 | 0.063 | 0 | 0.921 |
| CYP17A1 | 9606.ENSP00000358903 | SRD5A2 | 9606.ENSP00000477587 | 0.061 | 0 | 0.973 |
| CYP1A1 | 9606.ENSP00000369050 | CYP2C9 | 9606.ENSP00000260682 | 0.087 | 0 | 0.926 |
| CYP1A1 | 9606.ENSP00000369050 | CYP3A4 | 9606.ENSP00000337915 | 0.129 | 0 | 0.95 |
| CYP1A1 | 9606.ENSP00000369050 | HSD11B1 | 9606.ENSP00000355995 | 0.145 | 0.131 | 0.93 |
| CYP1A1 | 9606.ENSP00000369050 | GSTP1 | 9606.ENSP00000381607 | 0 | 0.077 | 0.908 |
| CYP1A1 | 9606.ENSP00000369050 | IDO1 | 9606.ENSP00000430950 | 0 | 0 | 0.926 |
| CYP1A1 | 9606.ENSP00000369050 | CYP1B1 | 9606.ENSP00000478561 | 0.277 | 0 | 0.935 |
| CYP1A1 | 9606.ENSP00000369050 | PPARA | 9606.ENSP00000385523 | 0.063 | 0.085 | 0.937 |
| CYP1A2 | 9606.ENSP00000342007 | CYP2C9 | 9606.ENSP00000260682 | 0.077 | 0 | 0.931 |
| CYP1A2 | 9606.ENSP00000342007 | CYP3A4 | 9606.ENSP00000337915 | 0.139 | 0 | 0.952 |
| CYP1A2 | 9606.ENSP00000342007 | IDO1 | 9606.ENSP00000430950 | 0 | 0 | 0.904 |
| CYP1A2 | 9606.ENSP00000342007 | XDH | 9606.ENSP00000368727 | 0.061 | 0 | 0.907 |
| CYP1A2 | 9606.ENSP00000342007 | CYP2C19 | 9606.ENSP00000360372 | 0 | 0 | 0.93 |
| CYP1B1 | 9606.ENSP00000478561 | IDO1 | 9606.ENSP00000430950 | 0 | 0 | 0.925 |
| CYP2C19 | 9606.ENSP00000360372 | CYP2C9 | 9606.ENSP00000260682 | 0.322 | 0.685 | 0.977 |
| CYP2C19 | 9606.ENSP00000360372 | CYP3A4 | 9606.ENSP00000337915 | 0.178 | 0 | 0.951 |
| CYP2C19 | 9606.ENSP00000360372 | MAOA | 9606.ENSP00000340684 | 0.063 | 0 | 0.926 |
| CYP2C19 | 9606.ENSP00000360372 | PTGS1 | 9606.ENSP00000354612 | 0.055 | 0 | 0.928 |
| CYP2C19 | 9606.ENSP00000360372 | PTGS2 | 9606.ENSP00000356438 | 0.055 | 0 | 0.926 |
| CYP2C19 | 9606.ENSP00000360372 | MAOB | 9606.ENSP00000367309 | 0.063 | 0 | 0.928 |
| CYP2C9 | 9606.ENSP00000260682 | PTGS1 | 9606.ENSP00000354612 | 0.055 | 0 | 0.932 |
| CYP2C9 | 9606.ENSP00000260682 | PTGS2 | 9606.ENSP00000356438 | 0.055 | 0 | 0.936 |
| CYP2C9 | 9606.ENSP00000260682 | EPHX2 | 9606.ENSP00000430269 | 0.051 | 0.081 | 0.951 |
| CYP2C9 | 9606.ENSP00000260682 | CYP3A4 | 9606.ENSP00000337915 | 0.247 | 0.379 | 0.97 |
| CYP3A4 | 9606.ENSP00000337915 | UGT2B7 | 9606.ENSP00000304811 | 0.142 | 0.379 | 0.969 |
| CYP3A4 | 9606.ENSP00000337915 | MAOB | 9606.ENSP00000367309 | 0.063 | 0 | 0.925 |
| CYP3A4 | 9606.ENSP00000337915 | HSD11B1 | 9606.ENSP00000355995 | 0.113 | 0.059 | 0.929 |
| CYP3A4 | 9606.ENSP00000337915 | MAOA | 9606.ENSP00000340684 | 0.063 | 0 | 0.932 |
| EGFR | 9606.ENSP00000275493 | MAPK1 | 9606.ENSP00000215832 | 0 | 0.472 | 0.965 |
| EGFR | 9606.ENSP00000275493 | PDGFRA | 9606.ENSP00000257290 | 0.107 | 0.418 | 0.923 |
| EGFR | 9606.ENSP00000275493 | PIK3CA | 9606.ENSP00000263967 | 0 | 0.37 | 0.99 |
| EGFR | 9606.ENSP00000275493 | STAT3 | 9606.ENSP00000264657 | 0 | 0.753 | 0.998 |
| EGFR | 9606.ENSP00000275493 | ERBB2 | 9606.ENSP00000269571 | 0.077 | 0.699 | 0.971 |
| EGFR | 9606.ENSP00000275493 | IL6 | 9606.ENSP00000385675 | 0 | 0 | 0.921 |
| EGFR | 9606.ENSP00000275493 | JAK2 | 9606.ENSP00000371067 | 0 | 0.472 | 0.928 |
| EGFR | 9606.ENSP00000275493 | ESR1 | 9606.ENSP00000405330 | 0 | 0.402 | 0.943 |
| EGFR | 9606.ENSP00000275493 | PRKCB | 9606.ENSP00000305355 | 0.049 | 0.403 | 0.945 |
| EGFR | 9606.ENSP00000275493 | PLAU | 9606.ENSP00000361850 | 0.088 | 0 | 0.951 |
| EGFR | 9606.ENSP00000275493 | PIK3CB | 9606.ENSP00000289153 | 0 | 0.204 | 0.954 |
| EGFR | 9606.ENSP00000275493 | LYN | 9606.ENSP00000428924 | 0 | 0.431 | 0.954 |
| EGFR | 9606.ENSP00000275493 | MMP3 | 9606.ENSP00000299855 | 0.076 | 0 | 0.954 |
| EGFR | 9606.ENSP00000275493 | PTK2B | 9606.ENSP00000380638 | 0.063 | 0.467 | 0.957 |
| EGFR | 9606.ENSP00000275493 | PTPN2 | 9606.ENSP00000311857 | 0 | 0.458 | 0.964 |
| EGFR | 9606.ENSP00000275493 | JAK1 | 9606.ENSP00000343204 | 0 | 0.573 | 0.966 |
| EGFR | 9606.ENSP00000275493 | MTOR | 9606.ENSP00000354558 | 0.061 | 0.204 | 0.986 |
| EGFR | 9606.ENSP00000275493 | PTPN11 | 9606.ENSP00000340944 | 0 | 0.8 | 0.996 |
| ELANE | 9606.ENSP00000466090 | MAPK1 | 9606.ENSP00000215832 | 0 | 0.053 | 0.937 |
| ELANE | 9606.ENSP00000466090 | MPO | 9606.ENSP00000225275 | 0.784 | 0.061 | 0.995 |
| ELANE | 9606.ENSP00000466090 | MMP8 | 9606.ENSP00000236826 | 0.175 | 0 | 0.967 |
| ELANE | 9606.ENSP00000466090 | MMP1 | 9606.ENSP00000322788 | 0.052 | 0 | 0.943 |
| ELANE | 9606.ENSP00000466090 | PTPN6 | 9606.ENSP00000391592 | 0.069 | 0 | 0.902 |
| EPHX2 | 9606.ENSP00000430269 | NOS2 | 9606.ENSP00000327251 | 0 | 0 | 0.913 |
| ERBB2 | 9606.ENSP00000269571 | PIK3CA | 9606.ENSP00000263967 | 0 | 0.08 | 0.986 |
| ERBB2 | 9606.ENSP00000269571 | STAT3 | 9606.ENSP00000264657 | 0 | 0.457 | 0.918 |
| ERBB2 | 9606.ENSP00000269571 | PIK3CB | 9606.ENSP00000289153 | 0 | 0.08 | 0.945 |
| ERBB2 | 9606.ENSP00000269571 | ESR1 | 9606.ENSP00000405330 | 0 | 0.32 | 0.947 |
| ERBB2 | 9606.ENSP00000269571 | PGR | 9606.ENSP00000325120 | 0 | 0.36 | 0.954 |
| ERBB2 | 9606.ENSP00000269571 | JAK2 | 9606.ENSP00000371067 | 0 | 0.454 | 0.962 |
| ERBB2 | 9606.ENSP00000269571 | IL6 | 9606.ENSP00000385675 | 0 | 0 | 0.972 |
| ERBB2 | 9606.ENSP00000269571 | PTPN11 | 9606.ENSP00000340944 | 0 | 0.8 | 0.994 |
| ESR1 | 9606.ENSP00000405330 | MAPK1 | 9606.ENSP00000215832 | 0 | 0.399 | 0.971 |
| ESR1 | 9606.ENSP00000405330 | GAPDH | 9606.ENSP00000229239 | 0 | 0.05 | 0.966 |
| ESR1 | 9606.ENSP00000405330 | MAPK14 | 9606.ENSP00000229795 | 0 | 0.399 | 0.962 |
| ESR1 | 9606.ENSP00000405330 | NR3C1 | 9606.ENSP00000231509 | 0 | 0 | 0.925 |
| ESR1 | 9606.ENSP00000405330 | PIK3CA | 9606.ENSP00000263967 | 0 | 0.379 | 0.977 |
| ESR1 | 9606.ENSP00000405330 | IGF1R | 9606.ENSP00000268035 | 0 | 0.407 | 0.989 |
| ESR1 | 9606.ENSP00000405330 | PGR | 9606.ENSP00000325120 | 0.102 | 0.472 | 0.964 |
| ESR1 | 9606.ENSP00000405330 | ESR2 | 9606.ENSP00000343925 | 0.061 | 0.379 | 0.941 |
| ESR2 | 9606.ENSP00000343925 | MAPK1 | 9606.ENSP00000215832 | 0 | 0.399 | 0.915 |
| ESR2 | 9606.ENSP00000343925 | PIK3CA | 9606.ENSP00000263967 | 0 | 0 | 0.913 |
| EZR | 9606.ENSP00000356042 | PRKCQ | 9606.ENSP00000263125 | 0 | 0 | 0.912 |
| EZR | 9606.ENSP00000356042 | F2 | 9606.ENSP00000308541 | 0 | 0 | 0.914 |
| F2 | 9606.ENSP00000308541 | MAPK1 | 9606.ENSP00000215832 | 0 | 0.053 | 0.931 |
| F2 | 9606.ENSP00000308541 | PIK3CA | 9606.ENSP00000263967 | 0 | 0 | 0.903 |
| F2 | 9606.ENSP00000308541 | LTB4R | 9606.ENSP00000380008 | 0 | 0 | 0.907 |
| F2 | 9606.ENSP00000308541 | PPARA | 9606.ENSP00000385523 | 0.063 | 0 | 0.908 |
| F2 | 9606.ENSP00000308541 | PTAFR | 9606.ENSP00000362965 | 0 | 0 | 0.919 |
| F2 | 9606.ENSP00000308541 | PLAU | 9606.ENSP00000361850 | 0 | 0.379 | 0.952 |
| F2 | 9606.ENSP00000308541 | F3 | 9606.ENSP00000334145 | 0 | 0 | 0.993 |
| F3 | 9606.ENSP00000334145 | PLG | 9606.ENSP00000308938 | 0 | 0.379 | 0.913 |
| G6PD | 9606.ENSP00000377192 | GSR | 9606.ENSP00000221130 | 0.179 | 0 | 0.964 |
| G6PD | 9606.ENSP00000377192 | GAPDH | 9606.ENSP00000229239 | 0.098 | 0.05 | 0.962 |
| HMOX1 | 9606.ENSP00000216117 | MAPK14 | 9606.ENSP00000229795 | 0 | 0 | 0.972 |
| HMOX1 | 9606.ENSP00000216117 | NFE2L2 | 9606.ENSP00000380252 | 0 | 0 | 0.996 |
| HSD11B1 | 9606.ENSP00000355995 | PPARG | 9606.ENSP00000287820 | 0.061 | 0 | 0.945 |
| IDO1 | 9606.ENSP00000430950 | MAOA | 9606.ENSP00000340684 | 0 | 0 | 0.917 |
| IDO1 | 9606.ENSP00000430950 | MAOB | 9606.ENSP00000367309 | 0 | 0 | 0.908 |
| IGF1R | 9606.ENSP00000268035 | MAPK1 | 9606.ENSP00000215832 | 0 | 0.146 | 0.934 |
| IGF1R | 9606.ENSP00000268035 | PIK3CA | 9606.ENSP00000263967 | 0.062 | 0.124 | 0.984 |
| IGF1R | 9606.ENSP00000268035 | PIK3CB | 9606.ENSP00000289153 | 0.062 | 0.124 | 0.915 |
| IGF1R | 9606.ENSP00000268035 | PTPN11 | 9606.ENSP00000340944 | 0.062 | 0.456 | 0.967 |
| IL6 | 9606.ENSP00000385675 | MAPK1 | 9606.ENSP00000215832 | 0 | 0 | 0.982 |
| IL6 | 9606.ENSP00000385675 | STAT3 | 9606.ENSP00000264657 | 0 | 0.269 | 0.997 |
| IL6 | 9606.ENSP00000385675 | PTPN11 | 9606.ENSP00000340944 | 0 | 0 | 0.954 |
| IL6 | 9606.ENSP00000385675 | JAK1 | 9606.ENSP00000343204 | 0.061 | 0 | 0.968 |
| IL6 | 9606.ENSP00000385675 | TLR9 | 9606.ENSP00000353874 | 0 | 0 | 0.905 |
| IL6 | 9606.ENSP00000385675 | PTGS2 | 9606.ENSP00000356438 | 0.314 | 0 | 0.945 |
| IL6 | 9606.ENSP00000385675 | MMP9 | 9606.ENSP00000361405 | 0.074 | 0 | 0.923 |
| IL6 | 9606.ENSP00000385675 | TLR4 | 9606.ENSP00000363089 | 0.082 | 0 | 0.959 |
| IL6 | 9606.ENSP00000385675 | JAK2 | 9606.ENSP00000371067 | 0.064 | 0 | 0.975 |
| IL6 | 9606.ENSP00000385675 | JAK3 | 9606.ENSP00000391676 | 0.062 | 0 | 0.959 |
| JAK1 | 9606.ENSP00000343204 | MAPK1 | 9606.ENSP00000215832 | 0.176 | 0.146 | 0.939 |
| JAK1 | 9606.ENSP00000343204 | MAPK14 | 9606.ENSP00000229795 | 0.062 | 0.146 | 0.925 |
| JAK1 | 9606.ENSP00000343204 | PDGFRA | 9606.ENSP00000257290 | 0 | 0.414 | 0.945 |
| JAK1 | 9606.ENSP00000343204 | PIK3CA | 9606.ENSP00000263967 | 0.061 | 0.124 | 0.936 |
| JAK1 | 9606.ENSP00000343204 | STAT3 | 9606.ENSP00000264657 | 0.096 | 0.437 | 0.997 |
| JAK1 | 9606.ENSP00000343204 | PIK3CB | 9606.ENSP00000289153 | 0.058 | 0.124 | 0.92 |
| JAK1 | 9606.ENSP00000343204 | PRKCB | 9606.ENSP00000305355 | 0 | 0.073 | 0.907 |
| JAK1 | 9606.ENSP00000343204 | PRKCE | 9606.ENSP00000306124 | 0.062 | 0.071 | 0.906 |
| JAK1 | 9606.ENSP00000343204 | PTPN2 | 9606.ENSP00000311857 | 0.062 | 0.521 | 0.988 |
| JAK1 | 9606.ENSP00000343204 | PTPN11 | 9606.ENSP00000340944 | 0.062 | 0.523 | 0.988 |
| JAK1 | 9606.ENSP00000343204 | PIK3CD | 9606.ENSP00000366563 | 0.058 | 0.124 | 0.914 |
| JAK1 | 9606.ENSP00000343204 | SYK | 9606.ENSP00000364907 | 0 | 0.321 | 0.941 |
| JAK1 | 9606.ENSP00000343204 | JAK2 | 9606.ENSP00000371067 | 0.085 | 0.379 | 0.943 |
| JAK1 | 9606.ENSP00000343204 | JAK3 | 9606.ENSP00000391676 | 0.061 | 0.379 | 0.943 |
| JAK1 | 9606.ENSP00000343204 | PTK2B | 9606.ENSP00000380638 | 0.061 | 0.433 | 0.949 |
| JAK1 | 9606.ENSP00000343204 | PTPN6 | 9606.ENSP00000391592 | 0.062 | 0.457 | 0.966 |
| JAK2 | 9606.ENSP00000371067 | MAPK1 | 9606.ENSP00000215832 | 0.064 | 0.146 | 0.934 |
| JAK2 | 9606.ENSP00000371067 | PIK3CA | 9606.ENSP00000263967 | 0.088 | 0.124 | 0.958 |
| JAK2 | 9606.ENSP00000371067 | STAT3 | 9606.ENSP00000264657 | 0.055 | 0.472 | 0.998 |
| JAK2 | 9606.ENSP00000371067 | KIT | 9606.ENSP00000288135 | 0 | 0.472 | 0.96 |
| JAK2 | 9606.ENSP00000371067 | PIK3CB | 9606.ENSP00000289153 | 0.062 | 0.124 | 0.925 |
| JAK2 | 9606.ENSP00000371067 | PTPN2 | 9606.ENSP00000311857 | 0.062 | 0.262 | 0.964 |
| JAK2 | 9606.ENSP00000371067 | NOS2 | 9606.ENSP00000327251 | 0 | 0.065 | 0.935 |
| JAK2 | 9606.ENSP00000371067 | PTPN11 | 9606.ENSP00000340944 | 0.062 | 0.525 | 0.992 |
| JAK2 | 9606.ENSP00000371067 | PIK3CG | 9606.ENSP00000352121 | 0.108 | 0.124 | 0.939 |
| JAK2 | 9606.ENSP00000371067 | PIK3CD | 9606.ENSP00000366563 | 0.061 | 0.124 | 0.918 |
| JAK2 | 9606.ENSP00000371067 | JAK3 | 9606.ENSP00000391676 | 0 | 0.328 | 0.933 |
| JAK2 | 9606.ENSP00000371067 | LYN | 9606.ENSP00000428924 | 0.061 | 0.379 | 0.95 |
| JAK2 | 9606.ENSP00000371067 | PTPN6 | 9606.ENSP00000391592 | 0.082 | 0.457 | 0.983 |
| JAK3 | 9606.ENSP00000391676 | MAPK1 | 9606.ENSP00000215832 | 0.064 | 0.146 | 0.929 |
| JAK3 | 9606.ENSP00000391676 | MAPK14 | 9606.ENSP00000229795 | 0.062 | 0.146 | 0.922 |
| JAK3 | 9606.ENSP00000391676 | PIK3CA | 9606.ENSP00000263967 | 0.062 | 0.124 | 0.951 |
| JAK3 | 9606.ENSP00000391676 | STAT3 | 9606.ENSP00000264657 | 0.062 | 0.46 | 0.994 |
| JAK3 | 9606.ENSP00000391676 | PIK3CB | 9606.ENSP00000289153 | 0.061 | 0.124 | 0.918 |
| JAK3 | 9606.ENSP00000391676 | PRKCB | 9606.ENSP00000305355 | 0.077 | 0.073 | 0.908 |
| JAK3 | 9606.ENSP00000391676 | PRKCE | 9606.ENSP00000306124 | 0.062 | 0.071 | 0.906 |
| JAK3 | 9606.ENSP00000391676 | PTPN2 | 9606.ENSP00000311857 | 0.062 | 0.522 | 0.976 |
| JAK3 | 9606.ENSP00000391676 | PTPN11 | 9606.ENSP00000340944 | 0.062 | 0.267 | 0.971 |
| JAK3 | 9606.ENSP00000391676 | SYK | 9606.ENSP00000364907 | 0.088 | 0.064 | 0.924 |
| JAK3 | 9606.ENSP00000391676 | PIK3CD | 9606.ENSP00000366563 | 0.116 | 0.124 | 0.917 |
| JAK3 | 9606.ENSP00000391676 | PTK2B | 9606.ENSP00000380638 | 0.082 | 0.433 | 0.948 |
| JAK3 | 9606.ENSP00000391676 | PTPN6 | 9606.ENSP00000391592 | 0.107 | 0.457 | 0.975 |
| KDR | 9606.ENSP00000263923 | MAPK14 | 9606.ENSP00000229795 | 0 | 0.084 | 0.921 |
| KDR | 9606.ENSP00000263923 | PTK2B | 9606.ENSP00000380638 | 0 | 0.093 | 0.916 |
| KDR | 9606.ENSP00000263923 | PIK3CB | 9606.ENSP00000289153 | 0 | 0.263 | 0.937 |
| KDR | 9606.ENSP00000263923 | PTPN2 | 9606.ENSP00000311857 | 0 | 0.094 | 0.951 |
| KDR | 9606.ENSP00000263923 | PIK3CA | 9606.ENSP00000263967 | 0.055 | 0.263 | 0.966 |
| KDR | 9606.ENSP00000263923 | PTPN11 | 9606.ENSP00000340944 | 0.063 | 0.435 | 0.968 |
| KDR | 9606.ENSP00000263923 | PTPN6 | 9606.ENSP00000391592 | 0.063 | 0.435 | 0.971 |
| KIT | 9606.ENSP00000288135 | PIK3CA | 9606.ENSP00000263967 | 0 | 0.447 | 0.98 |
| KIT | 9606.ENSP00000288135 | STAT3 | 9606.ENSP00000264657 | 0 | 0.096 | 0.959 |
| KIT | 9606.ENSP00000288135 | PTPN6 | 9606.ENSP00000391592 | 0.063 | 0.435 | 0.953 |
| KIT | 9606.ENSP00000288135 | LYN | 9606.ENSP00000428924 | 0.062 | 0.414 | 0.953 |
| KIT | 9606.ENSP00000288135 | PTPN11 | 9606.ENSP00000340944 | 0.063 | 0.435 | 0.974 |
| LTB4R | 9606.ENSP00000380008 | PIK3CA | 9606.ENSP00000263967 | 0 | 0.05 | 0.9 |
| LTB4R | 9606.ENSP00000380008 | PTAFR | 9606.ENSP00000362965 | 0 | 0 | 0.929 |
| LYN | 9606.ENSP00000428924 | MAPK1 | 9606.ENSP00000215832 | 0 | 0.446 | 0.959 |
| LYN | 9606.ENSP00000428924 | MAPK14 | 9606.ENSP00000229795 | 0 | 0.146 | 0.93 |
| LYN | 9606.ENSP00000428924 | PRKCQ | 9606.ENSP00000263125 | 0.062 | 0.398 | 0.942 |
| LYN | 9606.ENSP00000428924 | PIK3CA | 9606.ENSP00000263967 | 0.058 | 0.124 | 0.928 |
| LYN | 9606.ENSP00000428924 | STAT3 | 9606.ENSP00000264657 | 0.061 | 0.404 | 0.969 |
| LYN | 9606.ENSP00000428924 | PIK3CB | 9606.ENSP00000289153 | 0.058 | 0.124 | 0.922 |
| LYN | 9606.ENSP00000428924 | PTPN11 | 9606.ENSP00000340944 | 0.062 | 0.162 | 0.963 |
| LYN | 9606.ENSP00000428924 | PIK3CG | 9606.ENSP00000352121 | 0.106 | 0.432 | 0.957 |
| LYN | 9606.ENSP00000428924 | PLA2G4A | 9606.ENSP00000356436 | 0.061 | 0.131 | 0.925 |
| LYN | 9606.ENSP00000428924 | SYK | 9606.ENSP00000364907 | 0.243 | 0.455 | 0.966 |
| LYN | 9606.ENSP00000428924 | PIK3CD | 9606.ENSP00000366563 | 0.095 | 0.124 | 0.932 |
| LYN | 9606.ENSP00000428924 | PTK2B | 9606.ENSP00000380638 | 0.062 | 0.433 | 0.95 |
| LYN | 9606.ENSP00000428924 | PTPN6 | 9606.ENSP00000391592 | 0.107 | 0.457 | 0.984 |
| MAOA | 9606.ENSP00000340684 | MAOB | 9606.ENSP00000367309 | 0.18 | 0.685 | 0.945 |
| MAPK1 | 9606.ENSP00000215832 | PRKCB | 9606.ENSP00000305355 | 0.056 | 0.158 | 0.927 |
| MAPK1 | 9606.ENSP00000215832 | MYLK | 9606.ENSP00000353452 | 0.058 | 0 | 0.928 |
| MAPK1 | 9606.ENSP00000215832 | MET | 9606.ENSP00000317272 | 0.059 | 0.146 | 0.929 |
| MAPK1 | 9606.ENSP00000215832 | PRKCQ | 9606.ENSP00000263125 | 0.062 | 0.262 | 0.934 |
| MAPK1 | 9606.ENSP00000215832 | MPO | 9606.ENSP00000225275 | 0 | 0.077 | 0.944 |
| MAPK1 | 9606.ENSP00000215832 | PPARA | 9606.ENSP00000385523 | 0 | 0.399 | 0.951 |
| MAPK1 | 9606.ENSP00000215832 | PGR | 9606.ENSP00000325120 | 0 | 0.399 | 0.957 |
| MAPK1 | 9606.ENSP00000215832 | PRKCE | 9606.ENSP00000306124 | 0.062 | 0.522 | 0.959 |
| MAPK1 | 9606.ENSP00000215832 | NR3C1 | 9606.ENSP00000231509 | 0 | 0.399 | 0.961 |
| MAPK1 | 9606.ENSP00000215832 | PLA2G4A | 9606.ENSP00000356436 | 0 | 0.379 | 0.968 |
| MAPK1 | 9606.ENSP00000215832 | PTPN11 | 9606.ENSP00000340944 | 0.099 | 0.26 | 0.987 |
| MAPK1 | 9606.ENSP00000215832 | STAT3 | 9606.ENSP00000264657 | 0.06 | 0.384 | 0.989 |
| MAPK14 | 9606.ENSP00000229795 | TEK | 9606.ENSP00000369375 | 0 | 0.084 | 0.913 |
| MAPK14 | 9606.ENSP00000229795 | PTK2B | 9606.ENSP00000380638 | 0.064 | 0.157 | 0.929 |
| MAPK14 | 9606.ENSP00000229795 | SYK | 9606.ENSP00000364907 | 0.061 | 0.146 | 0.93 |
| MAPK14 | 9606.ENSP00000229795 | NR3C1 | 9606.ENSP00000231509 | 0 | 0.073 | 0.934 |
| MAPK14 | 9606.ENSP00000229795 | PLA2G4A | 9606.ENSP00000356436 | 0 | 0.379 | 0.971 |
| MAPK14 | 9606.ENSP00000229795 | STAT3 | 9606.ENSP00000264657 | 0.06 | 0.05 | 0.971 |
| MET | 9606.ENSP00000317272 | PIK3CA | 9606.ENSP00000263967 | 0.063 | 0.124 | 0.961 |
| MET | 9606.ENSP00000317272 | STAT3 | 9606.ENSP00000264657 | 0.062 | 0.432 | 0.978 |
| MET | 9606.ENSP00000317272 | PTPN2 | 9606.ENSP00000311857 | 0.062 | 0.288 | 0.932 |
| MET | 9606.ENSP00000317272 | PTPN11 | 9606.ENSP00000340944 | 0.062 | 0.472 | 0.971 |
| MMP1 | 9606.ENSP00000322788 | MMP2 | 9606.ENSP00000219070 | 0.154 | 0 | 0.922 |
| MMP1 | 9606.ENSP00000322788 | STAT3 | 9606.ENSP00000264657 | 0 | 0 | 0.966 |
| MMP1 | 9606.ENSP00000322788 | MMP3 | 9606.ENSP00000299855 | 0.529 | 0 | 0.953 |
| MMP1 | 9606.ENSP00000322788 | PLG | 9606.ENSP00000308938 | 0.063 | 0 | 0.96 |
| MMP1 | 9606.ENSP00000322788 | MMP9 | 9606.ENSP00000361405 | 0.518 | 0 | 0.961 |
| MMP12 | 9606.ENSP00000458585 | PLG | 9606.ENSP00000308938 | 0.063 | 0 | 0.952 |
| MMP2 | 9606.ENSP00000219070 | MMP9 | 9606.ENSP00000361405 | 0.061 | 0 | 0.908 |
| MMP2 | 9606.ENSP00000219070 | MMP3 | 9606.ENSP00000299855 | 0.181 | 0 | 0.923 |
| MMP2 | 9606.ENSP00000219070 | TEK | 9606.ENSP00000369375 | 0.142 | 0 | 0.95 |
| MMP2 | 9606.ENSP00000219070 | STAT3 | 9606.ENSP00000264657 | 0 | 0 | 0.979 |
| MMP3 | 9606.ENSP00000299855 | STAT3 | 9606.ENSP00000264657 | 0 | 0.379 | 0.968 |
| MMP3 | 9606.ENSP00000299855 | MMP9 | 9606.ENSP00000361405 | 0.518 | 0 | 0.959 |
| MMP3 | 9606.ENSP00000299855 | PLG | 9606.ENSP00000308938 | 0.063 | 0.379 | 0.979 |
| MMP8 | 9606.ENSP00000236826 | PTPN6 | 9606.ENSP00000391592 | 0 | 0 | 0.901 |
| MMP8 | 9606.ENSP00000236826 | PLG | 9606.ENSP00000308938 | 0.063 | 0 | 0.954 |
| MMP8 | 9606.ENSP00000236826 | MMP9 | 9606.ENSP00000361405 | 0.546 | 0 | 0.964 |
| MMP9 | 9606.ENSP00000361405 | STAT3 | 9606.ENSP00000264657 | 0 | 0 | 0.981 |
| MMP9 | 9606.ENSP00000361405 | PLG | 9606.ENSP00000308938 | 0.063 | 0.379 | 0.991 |
| MMP9 | 9606.ENSP00000361405 | PTPN6 | 9606.ENSP00000391592 | 0.123 | 0 | 0.914 |
| MPO | 9606.ENSP00000225275 | PTGS1 | 9606.ENSP00000354612 | 0.076 | 0 | 0.94 |
| MTOR | 9606.ENSP00000354558 | PIK3CA | 9606.ENSP00000263967 | 0.061 | 0.186 | 0.944 |
| MTOR | 9606.ENSP00000354558 | STAT3 | 9606.ENSP00000264657 | 0.049 | 0.379 | 0.989 |
| MTOR | 9606.ENSP00000354558 | PPARG | 9606.ENSP00000287820 | 0 | 0 | 0.901 |
| NFE2L2 | 9606.ENSP00000380252 | PRKCB | 9606.ENSP00000305355 | 0 | 0 | 0.907 |
| NOS2 | 9606.ENSP00000327251 | STAT3 | 9606.ENSP00000264657 | 0 | 0 | 0.955 |
| NOS2 | 9606.ENSP00000327251 | PTGS2 | 9606.ENSP00000356438 | 0.063 | 0.299 | 0.906 |
| NOS2 | 9606.ENSP00000327251 | PPARA | 9606.ENSP00000385523 | 0 | 0.091 | 0.922 |
| NR3C1 | 9606.ENSP00000231509 | PGR | 9606.ENSP00000325120 | 0 | 0 | 0.912 |
| NR3C1 | 9606.ENSP00000231509 | PIK3CA | 9606.ENSP00000263967 | 0.081 | 0 | 0.919 |
| NR3C1 | 9606.ENSP00000231509 | NR3C2 | 9606.ENSP00000350815 | 0 | 0.379 | 0.943 |
| NR3C2 | 9606.ENSP00000350815 | PGR | 9606.ENSP00000325120 | 0.061 | 0 | 0.914 |
| OPRM1 | 9606.ENSP00000394624 | STAT3 | 9606.ENSP00000264657 | 0 | 0 | 0.918 |
| OPRM1 | 9606.ENSP00000394624 | PTGDR2 | 9606.ENSP00000332812 | 0 | 0 | 0.9 |
| PDGFRA | 9606.ENSP00000257290 | PIK3CB | 9606.ENSP00000289153 | 0 | 0.08 | 0.916 |
| PDGFRA | 9606.ENSP00000257290 | PTPN11 | 9606.ENSP00000340944 | 0.063 | 0.435 | 0.971 |
| PDGFRA | 9606.ENSP00000257290 | PIK3CA | 9606.ENSP00000263967 | 0 | 0.404 | 0.978 |
| PDGFRA | 9606.ENSP00000257290 | STAT3 | 9606.ENSP00000264657 | 0 | 0.712 | 0.981 |
| PGR | 9606.ENSP00000325120 | PIK3CA | 9606.ENSP00000263967 | 0 | 0 | 0.913 |
| PIK3CA | 9606.ENSP00000263967 | PRKCQ | 9606.ENSP00000263125 | 0.064 | 0.165 | 0.918 |
| PIK3CA | 9606.ENSP00000263967 | PTAFR | 9606.ENSP00000362965 | 0 | 0 | 0.901 |
| PIK3CA | 9606.ENSP00000263967 | PIK3CD | 9606.ENSP00000366563 | 0.061 | 0 | 0.908 |
| PIK3CA | 9606.ENSP00000263967 | PIK3CB | 9606.ENSP00000289153 | 0.062 | 0 | 0.911 |
| PIK3CA | 9606.ENSP00000263967 | PTPN6 | 9606.ENSP00000391592 | 0 | 0.061 | 0.92 |
| PIK3CA | 9606.ENSP00000263967 | TEK | 9606.ENSP00000369375 | 0 | 0.08 | 0.921 |
| PIK3CA | 9606.ENSP00000263967 | PIK3CG | 9606.ENSP00000352121 | 0.061 | 0.154 | 0.925 |
| PIK3CA | 9606.ENSP00000263967 | SYK | 9606.ENSP00000364907 | 0.062 | 0.124 | 0.926 |
| PIK3CA | 9606.ENSP00000263967 | STAT3 | 9606.ENSP00000264657 | 0.049 | 0 | 0.943 |
| PIK3CA | 9606.ENSP00000263967 | PTPN11 | 9606.ENSP00000340944 | 0.063 | 0.061 | 0.949 |
| PIK3CB | 9606.ENSP00000289153 | PIK3CD | 9606.ENSP00000366563 | 0.061 | 0 | 0.905 |
| PIK3CB | 9606.ENSP00000289153 | PTPN11 | 9606.ENSP00000340944 | 0.061 | 0.061 | 0.919 |
| PIK3CB | 9606.ENSP00000289153 | TEK | 9606.ENSP00000369375 | 0 | 0.08 | 0.921 |
| PIK3CB | 9606.ENSP00000289153 | SYK | 9606.ENSP00000364907 | 0.062 | 0.124 | 0.923 |
| PIK3CB | 9606.ENSP00000289153 | PIK3CG | 9606.ENSP00000352121 | 0.061 | 0.157 | 0.927 |
| PIK3CD | 9606.ENSP00000366563 | PIK3CG | 9606.ENSP00000352121 | 0.105 | 0.441 | 0.907 |
| PIK3CD | 9606.ENSP00000366563 | SYK | 9606.ENSP00000364907 | 0.096 | 0.124 | 0.936 |
| PIK3CG | 9606.ENSP00000352121 | SYK | 9606.ENSP00000364907 | 0.147 | 0.378 | 0.953 |
| PLA2G4A | 9606.ENSP00000356436 | SYK | 9606.ENSP00000364907 | 0.061 | 0.131 | 0.934 |
| PLA2G4A | 9606.ENSP00000356436 | PTGS2 | 9606.ENSP00000356438 | 0.232 | 0 | 0.946 |
| PLAU | 9606.ENSP00000361850 | SERPINE1 | 9606.ENSP00000223095 | 0.214 | 0.881 | 0.999 |
| PLAU | 9606.ENSP00000361850 | PLG | 9606.ENSP00000308938 | 0 | 0.379 | 0.951 |
| PLAU | 9606.ENSP00000361850 | PTAFR | 9606.ENSP00000362965 | 0.063 | 0 | 0.933 |
| PLG | 9606.ENSP00000308938 | SERPINE1 | 9606.ENSP00000223095 | 0.063 | 0.435 | 0.998 |
| PLG | 9606.ENSP00000308938 | TEK | 9606.ENSP00000369375 | 0.055 | 0 | 0.941 |
| PRKCB | 9606.ENSP00000305355 | PRKCQ | 9606.ENSP00000263125 | 0.09 | 0 | 0.91 |
| PRKCB | 9606.ENSP00000305355 | PRKCE | 9606.ENSP00000306124 | 0.055 | 0 | 0.906 |
| PRKCB | 9606.ENSP00000305355 | PTK2B | 9606.ENSP00000380638 | 0.123 | 0 | 0.914 |
| PRKCE | 9606.ENSP00000306124 | PRKCQ | 9606.ENSP00000263125 | 0 | 0 | 0.904 |
| PRKCE | 9606.ENSP00000306124 | PTK2B | 9606.ENSP00000380638 | 0.076 | 0.133 | 0.918 |
| PRKCQ | 9606.ENSP00000263125 | PTK2B | 9606.ENSP00000380638 | 0 | 0.05 | 0.904 |
| PTAFR | 9606.ENSP00000362965 | STAT3 | 9606.ENSP00000264657 | 0 | 0 | 0.928 |
| PTGDR | 9606.ENSP00000303424 | PTGER2 | 9606.ENSP00000245457 | 0.076 | 0 | 0.908 |
| PTGDR | 9606.ENSP00000303424 | PTGIR | 9606.ENSP00000291294 | 0 | 0 | 0.907 |
| PTGDR | 9606.ENSP00000303424 | PTGER4 | 9606.ENSP00000302846 | 0.063 | 0 | 0.912 |
| PTGER2 | 9606.ENSP00000245457 | PTGIR | 9606.ENSP00000291294 | 0.061 | 0 | 0.907 |
| PTGER2 | 9606.ENSP00000245457 | PTGER4 | 9606.ENSP00000302846 | 0.155 | 0 | 0.927 |
| PTGER4 | 9606.ENSP00000302846 | PTGIR | 9606.ENSP00000291294 | 0.062 | 0 | 0.914 |
| PTGES | 9606.ENSP00000342385 | PTGS1 | 9606.ENSP00000354612 | 0.061 | 0 | 0.97 |
| PTGES | 9606.ENSP00000342385 | PTGS2 | 9606.ENSP00000356438 | 0.063 | 0 | 0.984 |
| PTGS2 | 9606.ENSP00000356438 | STAT3 | 9606.ENSP00000264657 | 0 | 0 | 0.98 |
| PTK2B | 9606.ENSP00000380638 | PTPN11 | 9606.ENSP00000340944 | 0.063 | 0.454 | 0.982 |
| PTK2B | 9606.ENSP00000380638 | SYK | 9606.ENSP00000364907 | 0.1 | 0.373 | 0.952 |
| PTPN11 | 9606.ENSP00000340944 | STAT3 | 9606.ENSP00000264657 | 0.062 | 0.436 | 0.991 |
| PTPN11 | 9606.ENSP00000340944 | TLR4 | 9606.ENSP00000363089 | 0.05 | 0.063 | 0.935 |
| PTPN11 | 9606.ENSP00000340944 | PTPN6 | 9606.ENSP00000391592 | 0 | 0.379 | 0.937 |
| PTPN11 | 9606.ENSP00000340944 | SYK | 9606.ENSP00000364907 | 0.062 | 0.16 | 0.943 |
| PTPN11 | 9606.ENSP00000340944 | TEK | 9606.ENSP00000369375 | 0.063 | 0.4 | 0.955 |
| PTPN2 | 9606.ENSP00000311857 | STAT3 | 9606.ENSP00000264657 | 0.064 | 0.415 | 0.985 |
| PTPN2 | 9606.ENSP00000311857 | PTPN6 | 9606.ENSP00000391592 | 0.062 | 0.904 | 0.918 |
| PTPN6 | 9606.ENSP00000391592 | SYK | 9606.ENSP00000364907 | 0.23 | 0.456 | 0.986 |
| RORA | 9606.ENSP00000261523 | STAT3 | 9606.ENSP00000264657 | 0 | 0.057 | 0.937 |
| RORC | 9606.ENSP00000327025 | STAT3 | 9606.ENSP00000264657 | 0 | 0.085 | 0.975 |
| SYK | 9606.ENSP00000364907 | TLR4 | 9606.ENSP00000363089 | 0.124 | 0.46 | 0.908 |

Supplementary Table 6: Information of key genes in *H. cuspidatus* treatment of asthma in the GEO database.

| Series | Tissue/Cell type | Number of cases | | Gene | *P*-value | t | B | logFoldChange |
| --- | --- | --- | --- | --- | --- | --- | --- | --- |
|  |  | Asthma | Control |  |  |  |  |  |
| GSE104471 | Peripheral blood mononuclear cells (PBMCs) | 12 | 12 | JAK3 | 0.00998 | 2.7900 | -3.3100 | 1.2500 |
|  |  |  |  | AKT1 | 0.01239 | -2.7000 | -3.4000 | -1.0600 |
|  |  |  |  | STAT3 | 0.49038 | -0.7000 | -4.8100 | -0.5000 |
|  | Nasal epithelia | 12 | 12 | MAPK14 | 0.01800 | 2.5400 | -3.2832 | 1.4400 |
|  |  |  |  | EGFR | 0.00141 | -3.6000 | -1.0151 | -0.9930 |
|  |  |  |  | ESR1 | 0.00750 | -2.9100 | -2.5136 | -0.9930 |
|  |  |  |  | CYP3A4 | 0.02260 | -2.4300 | -3.4833 | -0.6310 |
|  |  |  |  | JAK3 | 0.00506 | 3.0800 | -2.1634 | 0.7150 |
|  |  |  |  | PTPN6 | 0.00689 | 2.9500 | -2.4384 | 0.6880 |
|  |  |  |  | IL6 | 0.00109 | 3.7000 | -0.7808 | 0.6730 |
|  |  |  |  | APP | 0.00261 | -3.3500 | -1.5690 | -0.6430 |
|  |  |  |  | STAT3 | 0.00013 | 4.5400 | 1.1791 | 0.6250 |
|  |  |  |  | PIK3CA | 0.00187 | -3.4800 | -1.2717 | -0.6230 |
|  |  |  |  | AKR1C3 | 0.00695 | -2.9500 | -2.4461 | -0.5930 |
|  |  |  |  | LYN | 0.00125 | -3.6500 | -0.9038 | -0.5870 |
|  |  |  |  | AKT1 | 0.00001 | -5.4700 | 3.3391 | -0.5450 |
|  |  |  |  | PTPN11 | 0.00034 | -4.1600 | 0.2820 | -0.5220 |
|  | Bronchial epithelia | 12 | 12 | AKR1C3 | 0.00033 | -4.1748 | 0.2943 | -1.0100 |
|  |  |  |  | ESR1 | 0.00020 | -4.3695 | 0.7575 | -0.9410 |
|  |  |  |  | MAPK14 | 0.25000 | 1.1777 | -5.5293 | 0.8090 |
|  |  |  |  | PIK3CA | 0.00011 | -4.5812 | 1.2625 | -0.7010 |
|  |  |  |  | AKT1 | 0.00228 | -3.4045 | -1.5008 | -0.5850 |
|  |  |  |  | JAK1 | 0.13100 | 1.5638 | -5.0403 | 0.5580 |
|  |  |  |  | PTPN11 | 0.03050 | 2.2958 | -3.8271 | 0.5500 |
|  |  |  |  | JAK2 | 0.00032 | -4.1858 | 0.3204 | -0.5470 |
|  |  |  |  | JAK3 | 0.04550 | 2.1069 | -4.1714 | 0.5350 |
| GSE89809 | Bronchoalveolar lavage | 11 | 15 | PTPN11 | 0.00605 | -2.9707 | -2.3241 | -1.2072 |
|  |  |  |  | LYN | 0.08527 | -1.7841 | -4.1600 | -0.8790 |
|  |  |  |  | PIK3CA | 0.01413 | -2.6179 | -2.9235 | -0.8600 |
|  |  |  |  | JAK1 | 0.01419 | -2.6158 | -2.9270 | -0.7753 |
|  |  |  |  | MAPK13 | 0.05137 | -2.0356 | -3.8197 | -0.7212 |
|  |  |  |  | STAT3 | 0.10055 | -1.6984 | -4.2685 | -0.6242 |
|  |  |  |  | PIK3CB | 0.17886 | -1.3789 | -4.6347 | -0.6043 |
|  | Bronchial epithelia | 11 | 18 | MAPK13 | 0.02633 | 2.3281 | -3.3667 | 0.7650 |
|  |  |  |  | AKT1 | 0.01760 | 2.5020 | -3.0836 | 0.6835 |
|  |  |  |  | STAT3 | 0.10668 | 1.6596 | -4.3187 | 0.6219 |
|  |  |  |  | PIK3CB | 0.13965 | 1.5144 | -4.4923 | 0.5309 |
|  |  |  |  | JAK3 | 0.06714 | 1.8945 | -4.0113 | 0.5305 |
|  | Sputum | 9 | 10 | LYN | 0.00330 | 3.3241 | -1.6759 | 1.9030 |
|  |  |  |  | APP | 0.00166 | 3.6177 | -1.1045 | 1.8894 |
|  |  |  |  | PTPN11 | 0.03418 | 2.2691 | -3.6019 | 1.4076 |
|  |  |  |  | STAT3 | 0.03307 | 2.2851 | -3.5753 | 1.3563 |
|  |  |  |  | MAPK14 | 0.09361 | 1.7582 | -4.3942 | 0.8015 |
|  |  |  |  | SYK | 0.00309 | -3.3519 | -1.6220 | -0.7557 |
|  |  |  |  | PIK3CA | 0.08624 | 1.8021 | -4.3314 | 0.6714 |
|  |  |  |  | JAK2 | 0.07095 | -1.9045 | -4.1806 | -0.6657 |
|  |  |  |  | ESR1 | 0.02373 | -2.4427 | -3.3066 | -0.5419 |
| GSE74986 | Bronchial alveolar lavage cells | 44 | 12 | PIK3CA | 0.00000 | -6.3517 | 9.7897 | -1.1755 |
|  |  |  |  | JAK3 | 0.01230 | 2.5570 | -3.2911 | 1.0998 |
|  |  |  |  | JAK1 | 0.00001 | -4.7945 | 3.5985 | -0.8916 |
|  |  |  |  | PIK3CB | 0.00020 | -3.8803 | 0.4214 | -0.8638 |
|  |  |  |  | JAK2 | 0.00000 | -5.1379 | 4.8920 | -0.8536 |
|  |  |  |  | PTPN11 | 0.00021 | -3.8709 | 0.3908 | -0.7642 |
| GSE74075 | Sputum | 10 | 6 | APP | 0.03340 | -2.3331 | -3.8818 | -1.0066 |
|  |  |  |  | PTPN6 | 0.01210 | -2.8394 | -2.9566 | -0.8496 |
|  |  |  |  | AKT1 | 0.05390 | -2.0849 | -4.3056 | -0.6527 |
|  |  |  |  | IL6 | 0.30900 | 1.0518 | -5.7163 | 0.6107 |
|  |  |  |  | JAK1 | 0.12800 | -1.6083 | -5.0394 | -0.5937 |
|  |  |  |  | SYK | 0.00405 | -3.3650 | -1.9448 | -0.5037 |
| GSE37853 | Airway epithelial cells (AECs) | 4 | 6 | ESR1 | 0.05118 | -2.1931 | -4.1700 | -3.4697 |
|  |  |  |  | IL6 | 0.19837 | -1.3706 | -4.5000 | -3.2833 |
|  |  |  |  | JAK3 | 0.02849 | -2.5273 | -4.0300 | -2.8957 |
|  |  |  |  | SYK | 0.07818 | 1.9461 | -4.2700 | 2.7457 |
|  |  |  |  | EGFR | 0.16384 | -1.4941 | -4.4500 | -2.5419 |
|  |  |  |  | PTPN6 | 0.19976 | -1.3660 | -4.5000 | -2.0318 |
|  |  |  |  | JAK2 | 0.25401 | -1.2050 | -4.5600 | -1.6204 |
|  |  |  |  | APP | 0.53225 | -0.6453 | -4.7200 | -1.0309 |
|  | PBMCs | 5 | 6 | JAK3 | 0.07268 | -1.9959 | -4.3300 | -2.2427 |
|  |  |  |  | MAPK14 | 0.26748 | 1.1714 | -4.5700 | 1.5498 |
|  |  |  |  | JAK2 | 0.30557 | 1.0774 | -4.5900 | 1.2512 |
|  |  |  |  | SYK | 0.04717 | -2.2492 | -4.2600 | -0.6896 |
| GSE27335 | Airway fibroblasts | 8 | 4 | LYN | 0.14996 | 1.5320 | -4.5100 | 1.5373 |
|  |  |  |  | CYP3A4 | 0.04545 | -2.2170 | -4.3600 | -1.5075 |
|  |  |  |  | JAK3 | 0.14070 | 1.5708 | -4.5000 | 1.1027 |
|  |  |  |  | IL6 | 0.15571 | 1.5089 | -4.5100 | 1.0228 |
|  |  |  |  | APP | 0.04021 | 2.2837 | -4.3500 | 0.5504 |
|  |  |  |  | EGFR | 0.08982 | -1.8357 | -4.4400 | -0.5217 |
|  |  |  |  | STAT3 | 0.06915 | 1.9845 | -4.4100 | 0.5020 |
|  | Distal lung fibroblasts | 8 | 4 | ESR1 | 0.01210 | -2.9200 | -4.2200 | -1.4800 |
|  |  |  |  | LYN | 0.44400 | 0.7890 | -4.6300 | 0.5790 |
|  |  |  |  | SYK | 0.28300 | -1.1200 | -4.5800 | -0.5740 |
|  |  |  |  | JAK3 | 0.36100 | 0.9470 | -4.6100 | 0.5510 |
|  |  |  |  | PTPN6 | 0.07990 | 1.9000 | -4.4300 | 0.5460 |

Supplementary Table 7: Searching results of 19 key targets in the TISSUES database.

| Gene | Tissue distribution score in lung | Confidence |
| --- | --- | --- |
| STAT3 | 6.4 | ★★★★☆ |
| PIK3CA | 5.7 | ★★★☆☆ |
| MAPK1 | 4.7 | ★★★☆☆ |
| PTPN11 | 4.7 | ★★★☆☆ |
| EGFR | 7.1 | ★★★★☆ |
| JAK1 | 4.9 | ★★★☆☆ |
| LYN | 4.3 | ★★★☆☆ |
| JAK3 | 4.2 | ★★★☆☆ |
| JAK2 | 5.2 | ★★★☆☆ |
| APP | 4.4 | ★★★☆☆ |
| AKT1 | 7 | ★★★★☆ |
| PIK3CB | 3.9 | ★★☆☆☆ |
| IL6 | 7.4 | ★★★★☆ |
| PTPN6 | 3 | ★★☆☆☆ |
| MAPK14 | 4.8 | ★★★☆☆ |
| ESR1 | 6 | ★★★☆☆ |
| SYK | 4.7 | ★★★☆☆ |
| AKR1C3 | 3.7 | ★★☆☆☆ |
| CYP3A4 | 4.8 | ★★★☆☆ |

Supplementary Table 8: Interactions in the “key target-miRNA” network.

| Gene | miRNA |
| --- | --- |
| AKR1C3 | miR-563 |
| AKR1C3 | miR-937 |
| AKR1C3 | miR-641 |
| AKR1C3 | miR-935 |
| AKR1C3 | miR-592 |
| AKR1C3 | miR-553 |
| AKR1C3 | miR-379 |
| AKR1C3 | miR-377 |
| AKR1C3 | miR-597 |
| AKR1C3 | miR-629 |
| AKR1C3 | miR-598 |
| AKR1C3 | miR-192 |
| AKR1C3 | miR-184 |
| AKR1C3 | miR-511 |
| AKR1C3 | miR-190b |
| AKT1 | miR-625 |
| AKT1 | miR-520b |
| AKT1 | miR-639 |
| AKT1 | miR-520e |
| AKT1 | miR-449b |
| AKT1 | miR-632 |
| AKT1 | miR-449a |
| AKT1 | miR-504 |
| AKT1 | miR-302e |
| AKT1 | miR-802 |
| AKT1 | miR-655 |
| APP | miR-424 |
| APP | miR-376b |
| APP | miR-552 |
| APP | miR-380 |
| APP | miR-382 |
| APP | miR-20a |
| APP | miR-638 |
| APP | miR-634 |
| APP | miR-20b |
| APP | miR-668 |
| APP | miR-93 |
| APP | miR-302e |
| APP | miR-520h |
| APP | miR-17 |
| APP | miR-921 |
| APP | miR-372 |
| APP | miR-346 |
| APP | miR-520g |
| APP | miR-519d |
| APP | miR-143 |
| APP | miR-520b |
| APP | miR-29c |
| APP | miR-592 |
| APP | miR-29a |
| APP | miR-106b |
| APP | miR-520e |
| APP | miR-520f |
| APP | miR-298 |
| APP | miR-106a |
| APP | miR-25 |
| APP | miR-498 |
| APP | let-7i |
| APP | miR-495 |
| APP | let-7g |
| APP | miR-662 |
| APP | miR-660 |
| APP | miR-510 |
| APP | miR-92b |
| APP | miR-645 |
| CYP3A4 | miR-152 |
| CYP3A4 | miR-206 |
| CYP3A4 | miR-934 |
| CYP3A4 | miR-27a |
| CYP3A4 | miR-147b |
| CYP3A4 | miR-608 |
| CYP3A4 | miR-577 |
| CYP3A4 | miR-569 |
| CYP3A4 | miR-660 |
| CYP3A4 | miR-539 |
| CYP3A4 | miR-652 |
| EGFR | miR-452 |
| EGFR | miR-936 |
| EGFR | miR-382 |
| EGFR | miR-200a |
| EGFR | miR-421 |
| EGFR | miR-375 |
| EGFR | miR-891b |
| EGFR | miR-302e |
| EGFR | miR-539 |
| EGFR | miR-196b |
| EGFR | miR-545 |
| EGFR | miR-146a |
| EGFR | miR-141 |
| EGFR | miR-625 |
| EGFR | miR-520b |
| EGFR | miR-137 |
| EGFR | miR-586 |
| EGFR | miR-587 |
| EGFR | miR-520e |
| EGFR | miR-498 |
| EGFR | miR-575 |
| EGFR | miR-133b |
| EGFR | miR-662 |
| EGFR | miR-802 |
| ESR1 | miR-517b |
| ESR1 | miR-517a |
| ESR1 | miR-520b |
| ESR1 | miR-934 |
| ESR1 | miR-551b |
| ESR1 | miR-107 |
| ESR1 | miR-33b |
| ESR1 | miR-520e |
| ESR1 | miR-100 |
| ESR1 | miR-99a |
| ESR1 | miR-33a |
| ESR1 | miR-302e |
| ESR1 | miR-34b |
| ESR1 | miR-18a |
| ESR1 | miR-650 |
| ESR1 | miR-190b |
| ESR1 | miR-517c |
| IL6 | miR-555 |
| IL6 | miR-212 |
| IL6 | miR-376b |
| IL6 | miR-383 |
| IL6 | miR-552 |
| IL6 | miR-643 |
| IL6 | miR-374b |
| IL6 | miR-196b |
| IL6 | miR-760 |
| IL6 | miR-155 |
| IL6 | miR-522 |
| IL6 | miR-149 |
| IL6 | miR-144 |
| IL6 | miR-376c |
| IL6 | miR-137 |
| IL6 | miR-589 |
| IL6 | miR-132 |
| IL6 | miR-588 |
| IL6 | miR-587 |
| IL6 | miR-613 |
| IL6 | miR-498 |
| IL6 | let-7i |
| IL6 | let-7g |
| IL6 | miR-571 |
| IL6 | miR-655 |
| IL6 | miR-568 |
| IL6 | miR-217 |
| JAK1 | miR-934 |
| JAK1 | miR-382 |
| JAK1 | miR-23c |
| JAK1 | miR-635 |
| JAK1 | miR-630 |
| JAK1 | miR-184 |
| JAK1 | miR-373 |
| JAK1 | miR-297 |
| JAK1 | miR-545 |
| JAK1 | miR-520b |
| JAK1 | miR-302a |
| JAK1 | miR-592 |
| JAK1 | miR-587 |
| JAK1 | miR-520e |
| JAK1 | miR-520f |
| JAK1 | miR-583 |
| JAK1 | miR-302c |
| JAK1 | miR-605 |
| JAK1 | miR-302b |
| JAK1 | miR-302d |
| JAK1 | miR-601 |
| JAK1 | miR-597 |
| JAK1 | miR-448 |
| JAK1 | miR-656 |
| JAK1 | miR-214 |
| JAK1 | miR-202 |
| JAK1 | miR-648 |
| JAK1 | miR-567 |
| JAK1 | miR-561 |
| JAK2 | miR-144 |
| JAK2 | miR-621 |
| JAK2 | miR-380 |
| JAK2 | miR-494 |
| JAK2 | miR-300 |
| JAK2 | miR-381 |
| JAK2 | miR-633 |
| JAK2 | miR-155 |
| JAK2 | miR-375 |
| JAK2 | miR-190b |
| JAK3 | miR-21 |
| JAK3 | miR-924 |
| JAK3 | miR-421 |
| JAK3 | miR-198 |
| JAK3 | miR-541 |
| JAK3 | miR-98 |
| JAK3 | miR-668 |
| JAK3 | miR-505 |
| JAK3 | miR-631 |
| JAK3 | miR-95 |
| JAK3 | miR-939 |
| JAK3 | miR-487b |
| JAK3 | miR-182 |
| JAK3 | miR-196b |
| JAK3 | miR-921 |
| JAK3 | miR-367 |
| JAK3 | miR-623 |
| JAK3 | miR-708 |
| JAK3 | miR-130a |
| JAK3 | miR-1193 |
| JAK3 | miR-10a |
| JAK3 | miR-326 |
| JAK3 | miR-609 |
| JAK3 | miR-25 |
| JAK3 | miR-498 |
| JAK3 | miR-604 |
| JAK3 | miR-575 |
| JAK3 | miR-660 |
| JAK3 | miR-432 |
| JAK3 | miR-92B |
| LYN | miR-210 |
| LYN | miR-144 |
| LYN | miR-30a |
| LYN | miR-647 |
| LYN | miR-412 |
| LYN | miR-376c |
| LYN | miR-212 |
| LYN | miR-376b |
| LYN | miR-206 |
| LYN | miR-132 |
| LYN | miR-301a |
| LYN | miR-526b |
| LYN | miR-26b |
| LYN | miR-520f |
| LYN | miR-609 |
| LYN | let-7i |
| LYN | let-7d |
| LYN | miR-185 |
| LYN | miR-221 |
| LYN | miR-564 |
| LYN | miR-30d |
| LYN | miR-30b |
| MAPK1 | miR-384 |
| MAPK1 | miR-543 |
| MAPK1 | miR-206 |
| MAPK1 | miR-383 |
| MAPK1 | miR-936 |
| MAPK1 | miR-585 |
| MAPK1 | miR-1193 |
| MAPK1 | miR-326 |
| MAPK1 | miR-498 |
| MAPK1 | miR-573 |
| MAPK1 | miR-575 |
| MAPK1 | miR-22 |
| MAPK1 | miR-320a |
| MAPK1 | miR-217 |
| MAPK14 | miR-216b |
| MAPK14 | miR-421 |
| PIK3CA | miR-454 |
| PIK3CA | miR-212 |
| PIK3CA | miR-422a |
| PIK3CA | miR-380 |
| PIK3CA | miR-675 |
| PIK3CA | miR-19a |
| PIK3CA | miR-198 |
| PIK3CA | miR-373 |
| PIK3CA | miR-320a |
| PIK3CA | miR-370 |
| PIK3CA | miR-155 |
| PIK3CA | miR-372 |
| PIK3CA | miR-517c |
| PIK3CA | miR-148a |
| PIK3CA | miR-152 |
| PIK3CA | miR-517b |
| PIK3CA | miR-543 |
| PIK3CA | miR-517a |
| PIK3CA | miR-942 |
| PIK3CA | miR-143 |
| PIK3CA | miR-520b |
| PIK3CA | miR-137 |
| PIK3CA | miR-302a |
| PIK3CA | miR-132 |
| PIK3CA | miR-10b |
| PIK3CA | miR-520e |
| PIK3CA | miR-10A |
| PIK3CA | miR-302c |
| PIK3CA | miR-429 |
| PIK3CA | miR-302b |
| PIK3CA | miR-302d |
| PIK3CA | miR-135b |
| PIK3CA | miR-148b |
| PIK3CA | miR-561 |
| PIK3CB | miR-146a |
| PIK3CB | miR-543 |
| PIK3CB | miR-212 |
| PIK3CB | miR-620 |
| PIK3CB | miR-301a |
| PIK3CB | miR-130b |
| PIK3CB | miR-130a |
| PIK3CB | miR-325 |
| PIK3CB | miR-23c |
| PIK3CB | miR-433 |
| PIK3CB | miR-429 |
| PIK3CB | miR-23a |
| PIK3CB | miR-23b |
| PIK3CB | miR-887 |
| PIK3CB | miR-211 |
| PTPN6 | miR-412 |
| PTPN6 | miR-639 |
| PTPN6 | miR-632 |
| PTPN6 | miR-629 |
| PTPN6 | miR-627 |
| PTPN6 | miR-186 |
| PTPN6 | miR-942 |
| PTPN6 | miR-520b |
| PTPN6 | miR-302a |
| PTPN6 | miR-943 |
| PTPN6 | miR-526b |
| PTPN6 | miR-588 |
| PTPN6 | miR-617 |
| PTPN6 | miR-520e |
| PTPN6 | miR-520f |
| PTPN6 | miR-328 |
| PTPN6 | miR-608 |
| PTPN6 | miR-302c |
| PTPN6 | miR-578 |
| PTPN6 | miR-302b |
| PTPN6 | miR-302d |
| PTPN6 | miR-661 |
| PTPN6 | miR-657 |
| PTPN6 | miR-652 |
| PTPN6 | miR-202 |
| PTPN6 | miR-765 |
| PTPN11 | miR-429 |
| PTPN11 | miR-1193 |
| PTPN11 | miR-23c |
| STAT3 | miR-20b |
| STAT3 | miR-99b |
| STAT3 | miR-130a |
| STAT3 | miR-665 |
| STAT3 | miR-106b |
| STAT3 | miR-20a |
| STAT3 | miR-17 |
| STAT3 | miR-106a |
| STAT3 | miR-211 |
| SYK | miR-891a |
| SYK | miR-631 |

Supplementary Table 9: Differentially expressed miRNAs in asthma in published literature over the last three years.

| Differentially expressed miRNAs | References | Title | Detection methods/ model |
| --- | --- | --- | --- |
| miR-196a2 | [1] | A comprehensive analysis of microRNAs as diagnostic biomarkers for asthma | qRT-PCR and microarray |
| miR-92b |
| miR-210 |
| miR-34a |
| miR-155 |
| let-7a |
| miR-378 |
| miR-15a |
| miR-27b-3p |
| miR-21 |
| miR-146a |
| miR-126 |
| miR-1 |
| miR-192 |
| miR-29c |
| miR-149 |
| miR-625-5p |
| miR-513a-5p |
| miR-22-3p |
| miR-125b |
| let-7e |
| miR-98 |
| miR-497 |
| miR-483-3p |
| miR-221 |
| miR-125a |
| miR-21-5p |
| let-7a-5p |
| miR-218-5p |
| miR-17 |
| miR-185-5p |
| miR-320a |
| miR-1246 |
| miR-144-5p |
| miR-10a-5p |
| miR-146a-5p |
| miR-1165-3p |
| miR-199a-5p |
| miR-200b/c |
| miR-1268 |
| miR-663a |
| miR-30a |
| let-7a/b |
| miR-99a |
| miR-27a |
| miR-133a |
| miR-24 |
| miR-221-3p |
| miR-323-3p |
| miR-145 |
| miR-338 |
| miR-15a-5p |
| miR-27a-3p |
| miR-29c-3p |
| miR-223-3p |
| miR-425-5p |
| miR-342-3p |
| miR-371 |
| miR-138 |
| miR-544 |
| miR-214 |
| miR-101 |
| miR-19a |
| miR-34 |
| miR-449 |
| miR-194 |
| miR-375 |
| miR-181b-5p |
| miR-181a-5p |
| miR-31 |
| miR-629-3p |
| miR-142-3p |
| miR-16 |
| miR-223 |
| miR-148 |
| miR-299-5p |
| miR-570 |
| miR-150 |
| miR-224 |
| miR-339-5p |
| miR-382 |
| let-7f |
| miR-18a |
| miR-128 |
| miR-570-3p |
| miR-498 |
| miR-187 |
| miR-87 |
| miR-143 |
| miR-886-2p |
| miR-658 |
| miR-26a |
| miR-200c |
| miR-148b |
| miR-487b |
| miR-181c |
| miR-1248 |
| let-7d |
| miR-34c-5p |
| miR-34b-5p |
| miR-449a |
| miR-449b-5p |
| miR-146b |
| miR-28a-5p |
| miR-140 |
| miR-30b/c/d |
| miR-93 |
| miR-191 |
| miR-320 |
| miR-342 |
| let-7a-5p | [2] | A Network of Sputum MicroRNAs Is Associated with Neutrophilic Airway Inflammation in Asthma | Nanostring nCounter array |
| let-7d-5p |
| let-7g-5p |
| let-7I-5p |
| miR-15b-5p |
| miR-23a-3p |
| miR-25-3p |
| miR-150-5p |
| miR-191-5p |
| miR-181a-5p |
| miR-223-3p |
| miR-342 |
| let-7f | [3] | A Review of Macrophage MicroRNAs' Role in Human Asthma | review the current published data |
| miR-9 |
| miR-18a |
| miR-19a |
| miR-19b-3p |
| miR-26a/b |
| miR-27a/b |
| miR-125b |
| miR-155 |
| let-7a |
| let-7b |
| let-7c |
| let-7e |
| miR-21 |
| miR-34 |
| miR-124a |
| miR-146a |
| miR-223 |
| miR-511 |
| miR-574-5p | [4] | Circulating MicroRNA: Incident Asthma Prediction and Vitamin D Effect Modification | Small RNA sequencing |
| miR-155 | [5] | Circulating microRNAs correlate to clinical parameters in individuals with allergic and non-allergic asthma | qRT-PCR |
| miR-146a |
| miR-223 |
| miR-126 |
| miR-374a |
| miR-145 |
| miR-92a-3p | [6] | Construction of asthma related competing endogenous RNA network revealed novel long non-coding RNAs and potential new drugs | GEO database |
| miR-93-5p |
| miR-126-5p |
| miR-155-5p |
| miR-223-3p |
| miR-48b | [7] | Coordinated DNA Methylation and Gene Expression Data for Identification of the Critical Genes Associated with Childhood Atopic Asthma | GEO database |
| miR-152 |
| miR-148a |
| miR-126 |
| miR-155-5p | [8] | Effects of AntagomiRs on Different Lung Diseases in Human, Cellular, and Animal Models | PubMed database |
| miR-21 |
| miR-1276 | [9] | Eosinophil microRNAs Play a Regulatory Role in Allergic Diseases Included in the Atopic March | RNA sequencing |
| miR-29b2 |
| miR-3175 |
| miR-33b |
| miR-4308 |
| miR-4523 |
| miR-4673 |
| miR-4785 |
| miR-590 |
| miR-638 |
| miR-146a | [10] | Epigenetics in Asthma | review the current published literature |
| miR-152 |
| miR-155 |
| miR-221 |
| miR-26 |
| MIIR133a |
| miR-140 |
| miR-206 |
| miR-221 |
| miR-28-5p |
| miR-146a/b |
| miR-223-3p |
| miR-142-3p |
| miR-629-3p |
| miR-296-5p |
| miR-145 | [11] | Involvement of microRNAs in physiological and pathological processes in asthma | review the current published literature |
| miR-21 |
| miR-138 |
| miR-143-3p |
| miR-20b |
| let-7a |
| miR-1248 |
| miR-146a |
| miR-126 |
| miR-26a |
| let-7d |
| miR-1165-3p |
| miR-1248 |
| miR-21 |
| miR-126 |
| miR-21 |
| let-7c |
| miR-1 |
| miR-16 |
| miR-299-5p |
| miR-146a |
| miR-150 |
| miR-133b |
| miR-1248 |
| miR-29 |
| miR-422 |
| miR-1291 |
| miR-206 |
| miR-328 |
| miR-26a |
| miR-106a |
| miR-155 |
| miR-181b-5p |
| miR-125b |
| let-7b |
| let-7e |
| miR-223 |
| miR-570 |
| miR-148a |
| miR-133a |
| miR-26b |
| miR-330-5p |
| miR-145 |
| miR-144 |
| miR-146a |
| miR-338-3p |
| miR-193 |
| miR-221 |
| miR-155 |
| miR-145 |
| miR-19a |
| miR-323-3p |
| miR-625-5p |
| miR-22-3p |
| miR-513a-5p |
| miR-192 |
| miR-146a |
| miR-146b |
| miR-629-3p |
| miR-223-3p |
| miR-142-3p |
| miR-19a |
| miR-21 |
| miR-126 |
| miR-145 |
| miR-146 |
| miR-221 |
| miR-221 |
| miR-498 |
| miR-187 |
| miR-498 |
| miR-874 |
| miR-143 |
| miR-886-3p |
| miR-21 |
| miR-487b |
| miR-181c |
| let-7f |
| miR-18a |
| miR-19b |
| miR-27b |
| miR-106b |
| miR-128 |
| miR-181b-5p |
| miR-181a-5p |
| let-7a |
| miR-221 |
| miR-34c-5p |
| miR-449a |
| miR-449b-5p |
| miR-18a |
| miR-126 |
| miR-18a |
| miR-126 |
| let-7e |
| miR-224 |
| miR-138 |
| miR-143-3p |
| miR-203 |
| miR-19a |
| miR-192 |
| miR-221 |
| miR-145 |
| miR-146 |
| miR-34c-5p |
| miR-449a |
| miR-449b-5p |
| miR-21 |
| miR-323-3p |
| miR-155-5p | [12] | MicroRNAs as biomarkers in corticosteroid-resistant/neutrophilic asthma: still a long way to go! | Small RNA Sequencing and Profiling |
| miR-532-5p |
| miR-223-3p |
| miR-140-3p | [13] | MicroRNAs as Potential Regulators of Immune Response Networks in Asthma and Chronic Obstructive Pulmonary Disease | review the current published literature |
| miR-145 |
| miR-146a-5p |
| miR-638 |
| miR-708 |
| miR-146a/b |
| miR-48 |
| miR-84 |
| miR-241 |
| miR-126 |
| miR-200 family |
| miR-346 |
| miR-574-5p |
| let-7 |
| miR-24 |
| miR-27 |
| miR-16 |
| miR-125b |
| miR-133b |
| miR-206 |
| miR-144-5p |
| miR-185-5p |
| miR-320a |
| miR-1246 |
| miR-485-5p |
| miR-3162-3p |
| miR-221 |
| miR-21 |
| miR-142-3p |
| miR-223-3p |
| miR-629-3p |
| miR-221-3p |
| miR-146a | [14] | MicroRNAs: future biomarkers and targets of therapy in asthma? | review the current published literature |
| miR-210 |
| miR-34a |
| miR-181 |
| miR-140-3p |
| miR-708 |
| miR-10 |
| miR-142-3p |
| miR-25 |
| miR-133 |
| miR-155 |
| miR-210 |
| miR-181a |
| miR-21 |
| miR-19a |
| miR-221-3p |
| miR-1248 |
| miR-146a |
| let-7 family |
| miR-323-3p |
| miR-181a |
| miR-26a |
| miR-513-5p |
| miR-22-3p |
| miR-625-5p |
| miR-16 | [15] | MicroRNAs: potential biomarkers and targets of therapy in allergic diseases? | review the current published literature |
| miR-21 |
| miR-125b |
| miR-126 |
| miR-145 |
| miR-146a |
| miR-148a |
| miR-221 |
| miR-223 |
| miR-338 |
| miR-485-3p |
| miR-18a |
| miR-126 |
| let-7e |
| miR-155 |
| miR-224 |
| miR-498 |
| miR-187 |
| miR-874 |
| miR-143 |
| miR-886-3p |
| miR-296-5p |
| miR-16-5p |
| miR-203 |
| miR-30d-5p |
| miR-140-3p |
| miR-708 |
| miR-10 |
| miR-142-3p |
| miR-25 |
| miR-133 |
| miR-19a |
| miR-181-5p |
| miR-21 |
| miR-223 |
| miR-199a-5p |
| miR-223-3p |
| miR-142-3p |
| miR-629-3p |
| miR-210 |
| miR-181a |
| miR-19a |
| miR-221-3p |
| miR-1248 |
| miR-146a |
| miR-323-3p |
| let-7 family |
| miR-181a |
| miR-26a |
| miR-513-5p |
| miR-22-3p |
| miR-625-5p |
| miR-744 | [16] | miRNAs in lung development and diseases | review the current published literature |
| miR-943-3p |
| miR-30a |
| let-7 | [17] | Research progress of non-coding RNA in regulating the function of T cells in asthma | review the current published literature |
| let-7f |
| miR-106a |
| miR-323-3p |
| miR-21 |
| miR-146a |
| miR-126 |
| miR-145 |
| miR-29c |
| miR-192 |
| miR-24 |
| miR-27 |
| miR-17 |
| miR-18 |
| miR-19a |
| miR-19b |
| miR-20 |
| miR-92 |
| miR-21 | [18] | Role of microRNA in severe asthma | review the current published literature |
| miR-126 |
| miR-145 |
| miR-629-3p |
| miR-223-3p |
| miR-142-3p |
| miR-200b |
| miR-200c |
| miR-487b-3p |
| miR-221-3p |
| miR-19a |
| miR-145 |
| miR-708-5p |
| miR-140-3p |
| miR-221 |
| miR-150 |
| miR-152 |
| miR-375 |
| miR-27b-3p | [19] | Roles of microRNAs in chronic pediatric diseases and their use as potential biomarkers: A review | review the current published literature |
| miR-143a |
| miR-223a |
| miR-21 |
| miR-221 |
| miR-485-3p |
| miR-21-5p |
| miR-146a-3p |
| miR-155-5p |
| miR-15b | [20] | Spotlight on microRNAs in allergy and asthma | review the current published literature |
| miR-126 |
| miR-139 |
| miR-142 |
| miR-186 |
| miR-191 |
| miR-342 |
| miR-374a |
| miR-409 |
| miR-660 |
| miR-942 |
| miR-1290 |
| miR-16 |
| miR-30d |
| miR-296 |
| miR-146a |
| miR-206 |
| miR-720 |
| miR-16 |
| miR-125b |
| miR-133b |
| miR-206 |
| miR-299 |
| let-7a |
| miR-21 |
| miR-133a |
| miR-155 |
| miR-328 |
| miR-1248 |
| miR-19a |
| miR-21 |
| miR-155 |
| miR-221 |
| miR-185 |
| miR-16 |
| miR-223 |
| miR-513a |
| miR-625 |
| let-7a |
| miR-1248 |
| miR-15a |
| miR-146a |
| miR-210 |
| miR-146a |
| miR-374a |
| miR-223 |
| miR-155 | [21] | The role of epigenetics in the development of childhood asthma | summarize recent epigenetic studies of childhood asthma in humans |
| miR-210 |
| miR-21 |
| miR-19a |
| miR-140-3p |
| miR-708 |
| miR-10 |
| miR-323-3p |
| miR-21 |
| miR-146a |
| miR-181b | [22] | Long non-coding RNA TUG1 promotes airway remodeling and mucus production in asthmatic mice through the microRNA-181b/HMGB1 axis | Animal model |
| miR-106b-5p | [23] | miR‑106b‑5p targeting SIX1 inhibits TGF‑β1‑induced pulmonary fibrosis and epithelial‑mesenchymal transition in asthma through regulation of E2F1 | Cell and animal model |
[truncated: 17,656 more chars]
